# Synthesis, Biological Evaluation, and Molecular Docking Studies of 2*r*,3*t*,4*c*‐Configured *C*‐Furanosidic LpxC Inhibitors

**DOI:** 10.1002/ardp.70270

**Published:** 2026-07-07

**Authors:** André Behnk, Fabian Lüttchens, Frederick Wichter, Katharina Hoff, Elisa Venanzi, Stefan Wimmer, Christopher Vorreiter, Katharina Rox, Wolfgang Sippl, Ralph Holl

**Affiliations:** ^1^ Institute of Organic Chemistry Universität Hamburg Hamburg Germany; ^2^ German Center for Infection Research (DZIF) partner site Hamburg‐Lübeck‐Borstel‐Riems Hamburg Germany; ^3^ Institute of Pharmacy Martin‐Luther‐University of Halle‐Wittenberg Halle (Saale) Germany; ^4^ Department of Chemical Biology Helmholtz Centre for Infection Research (HZI) Braunschweig Germany; ^5^ German Center for Infection Research (DZIF) Partner Site Hannover‐Braunschweig Braunschweig Germany; ^6^ Research group Pharmacokinetics and Pharmacodynamics Unit Helmholtz Centre for Infection Research (HZI) Braunschweig Germany

**Keywords:** antibacterials, *C*‐glycosides, *in vitro* ADMET studies, LpxC inhibitors, molecular docking studies

## Abstract

In chiral pool syntheses starting from d‐mannitol and d‐galactose, a series of four 2*r*,3*t*,4*c*‐configured *C*‐furanosides was accessed. The synthesized stereoisomeric hydroxamic acids were tested for antibacterial activity as well as inhibitory potency toward the bacterial deacetylase LpxC, a promising target for antibiotics selectively targeting Gram‐negative bacteria. The obtained biological data were compared with those of the previously synthesized 12 stereoisomers, and molecular docking as well as molecular dynamics studies were performed to rationalize the observed structure–activity relationships. Initial *in vitro* ADMET studies revealed excellent microsomal metabolic and plasma stability of the investigated *C*‐furanosides.

## Graphical abstract

To provide a full picture of the effects of stereochemistry on the biological activity of *C*‐furanosidic LpxC inhibitors, in chiral pool syntheses starting from d‐mannitol and d‐galactose, a series of four 2*r*,3*t*,4*c*‐configured *C*‐furanosides was synthesized and tested for LpxC inhibitory and antibacterial activity as well as *in vitro* ADMET properties.

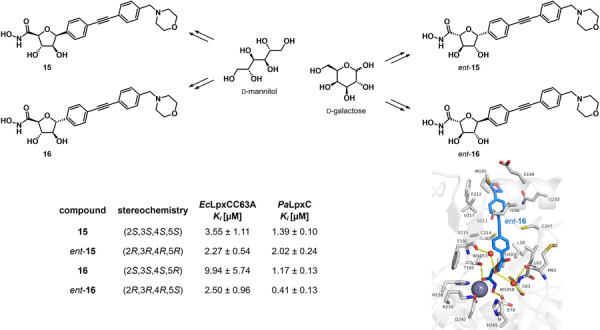

## 1 Introduction

**Figure 1:**
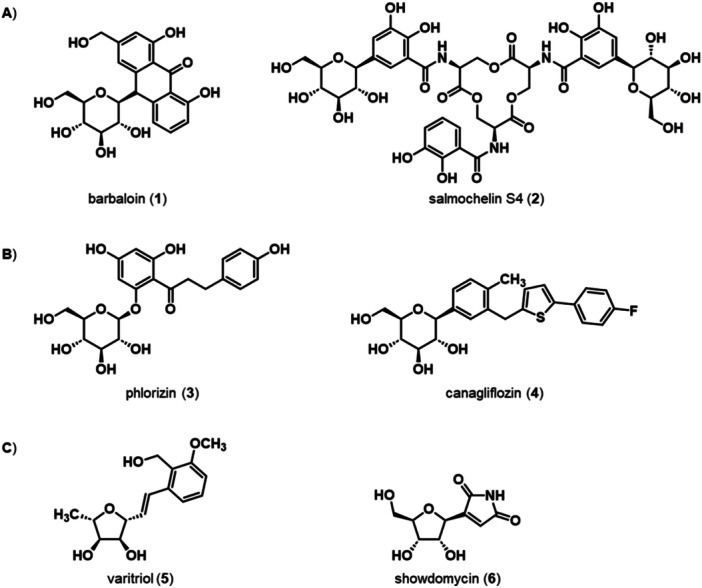
(A) Chemical structures of the naturally occurring *C*‐glycosides barbaloin (**1**) and salmochelin S4 (**2**) with an anthrone‐ and an enterobactin‐derived aglycon, respectively. (B) Chemical structures of phlorizin (**3**), a natural *O*‐glycoside with SGLT2‐inhibitory activity, and of canagliflozin (**4**), a synthetic *C*‐glycosidic SGLT2 inhibitor. (C) Chemical structures of varitriol (**5**) and showdomycin (**6**), two naturally occurring *C*‐furanosides.

*C*‐Glycosides constitute carbohydrate derivatives in which a carbon atom replaces the anomeric oxygen of a glycosidic linkage [1, 2, 3, 4]. These compounds are widely present in nature with a plethora of bioactive molecules such as the plant‐derived anthrone *C*‐glucoside barbaloin (**1**, Figure 1) [5, 6] or salmochelin S4 (**2**) [7], a twofold *C*‐glucosylated analog of the siderophore enterobactin, which is excreted by uropathogenic *Escherichia coli* and *Salmonella enterica*. Compared with *O*‐ and *N*‐glycosides, *C*‐glycosides exhibit higher stability against chemical and enzymatic hydrolysis; thus, these compounds are investigated as carbohydrate mimetics in pharmaceutical research [8, 9, 10]. One such example is the development of SGLT2 inhibitors, where the naturally occurring *O*‐glycoside phlorizin (**3**) was developed into the *C*‐glycoside canagliflozin (**4**) [11].

*C*‐Furanosides represent a sub‐class of the *C*‐glycosides, exhibiting a five‐membered ring comprising one oxygen and four carbon atoms. Examples for naturally occurring *C*‐furanosides are varitriol (**5**) [12], a vinylic *C*‐furanoside found in the marine fungus *Emericella variecolor*, and showdomycin (**6**) [13], a *C*‐furanoside with resemblance to uridine, exhibiting potent antibiotic properties.

Synthetically, *C*‐furanosides can be accessed *via* different synthetic strategies [14, 15, 16, 17], such as the addition of a suitable nucleophilic aglycon donor species to a lactone, followed by the reduction of the resulting lactol [18, 19, 20]. Other methods include sugars bearing an activating group at the anomeric center, which, *via* the formation of an intermediate oxocarbenium cation, can be reacted with a nucleophile to obtain the desired *C*‐furanoside [21, 22, 23]. Alternatively, *C*‐furanosides can be obtained *via* intramolecular cyclization reactions [24, 25, 26, 27].

**Figure 2:**
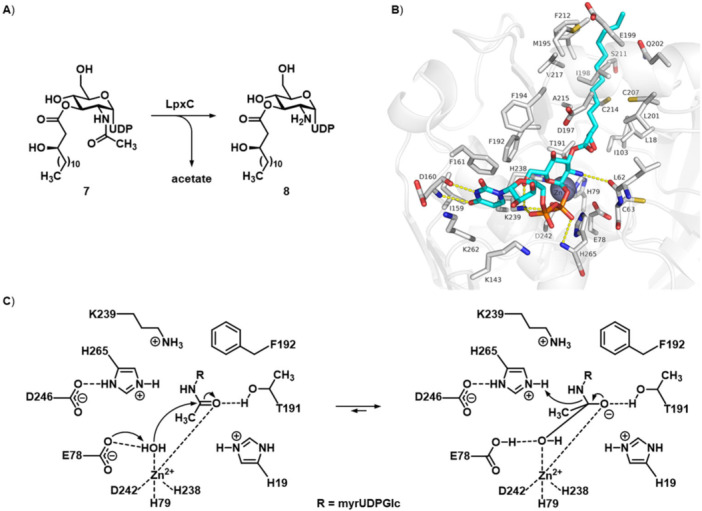
(A) LpxC‐catalyzed deacetylation of UDP‐3‐*O*‐[(*R*)−3‐hydroxymyristoyl]‐*N*‐acetylglucosamine (**7**). (B) Molecular surface of *E. coli* LpxC near the deacetylated natural product **8** (PDB 4MDT) [40]. Carbon atoms of **8** are shown in cyan color. The side chains of amino acid residues are shown in stick representation with white carbon atoms. The surface of the binding pocket is shown in white. The Zn^2+^‐ion is shown as a cyan sphere. Protein–ligand interactions are shown as yellow dashed lines. (C) Proposed catalytic reaction mechanism of the LpxC‐catalyzed deacetylation of **7** [41, 42].

Previously, we reported on the synthesis and biological evaluation of a series of *C*‐furanosides as LpxC inhibitors [28, 29, 30, 31, 32, 33, 34, 35, 36]. LpxC is a Zn^2+^‐dependent metalloamidase, which is present in virtually all Gram‐negative bacteria and highly conserved among them [37, 38, 39]. The enzyme catalyzes the second and committed step of lipid A biosynthesis, being in *E. coli* the irreversible deacetylation of UDP‐3‐*O*‐[(*R*)−3‐hydroxymyristoyl]‐*N*‐acetylglucosamine (**7**, Figure 2A) [43, 44]. Lipid A acts as the hydrophobic membrane anchor of the lipopolysaccharides (LPS) and constitutes the main component of the outer leaflet of the outer membrane of Gram‐negative bacteria, thus being required for the maintenance of an effective outer membrane barrier and in consequence for growth and viability of these germs [38, 45, 46]. Therefore, the inhibition of lipid A biosynthesis is lethal to Gram‐negative bacteria, rendering LpxC a promising target for the development of novel antibiotics [47, 48, 49, 50].

Structural studies revealed that the enzyme comprises two domains with similar topologies [51]. Each domain consists of two α‐helices being packed against a five‐stranded β‐sheet [52]. The assembly of the two domains results in a “β‐α‐α‐β sandwich” fold, with the α‐helices being sandwiched between the β‐sheets. The conical active site cleft is located at one side of the sandwich at the interface of the two domains [53]. Additionally, each domain comprises a unique insert region [51]. Whereas Insert I of Domain I partially shapes the boundary of the active site, Insert II of Domain II largely forms a hydrophobic tunnel leading out of the active site, which binds the fatty acyl chain of the enzyme's natural substrate **7** during catalysis [39, 54]. The catalytic Zn^2+^‐ion of LpxC is located at the bottom of the active site cleft, being complexed by one aspartate and two histidine residues (i.e., H79, H238, and D242, in the case of *E. coli* LpxC) [40].

A crystal structure of *E. coli* LpxC in complex with the deacetylated reaction product **8** provided particular insight into substrate recognition by the deacetylase (Figure 2B) [40]. While the glucosamine moiety interacts with the enzyme *via* a water‐mediated contact to D242 and direct hydrogen bonds between the 6′‐OH group and K239, as well as the 2‐amino group and L62, it binds upon a hydrophobic patch provided by F192 and F194. The pyrophosphate moiety of reaction product **8** is directly recognized by the basic patch residue K239 and undergoes water‐mediated contacts with K143 and D242.

Various active site residues were shown to participate in the catalytic reaction mechanism of LpxC, which has been proposed to proceed *via* a general acid/base mechanism, with E78 and H265 acting as a general base and a general acid, respectively (Figure 2C) [40, 47, 55]. Accordingly, a hydroxide ion, being formed *via* the deprotonation of a Zn^2+^‐bound water molecule by general base E78, attacks the acetamide carbonyl group of substrate **7**, generating a tetrahedral *gem*‐diolate, which is stabilized by T191. Subsequently, the *gem*‐diolate accepts a proton from general acid H265, which leads to the cleavage of the C–N bond and the formation of the deacetylated reaction product **8**.

**Figure 3:**
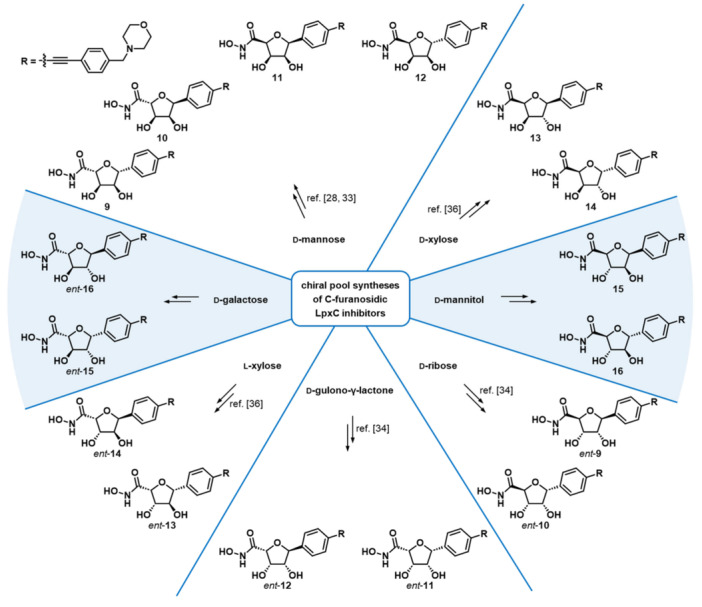
Structures of the *C*‐furanosidic LpxC inhibitor **9** and its 15 possible stereoisomers, which can be obtained in chiral pool syntheses starting from various sugars or sugar derivatives. The stereoisomers described in this paper are highlighted in blue.

Derived from the glycosidic nature of the natural substrate (**7**) of LpxC, we developed a series of *C*‐furanosidic LpxC inhibitors (Figure 3) [28, 33, 34, 36]. These compounds bear a hydroxamate moiety to chelate the catalytic Zn^2+^‐ion of LpxC and a lipophilic side chain to address the hydrophobic tunnel of the enzyme in positions 2 and 5 of their tetrahydrofuran ring, respectively, whereas two hydroxyl groups are located in positions 3 and 4. The *C*‐glycosidic scaffold should lead to a conformational constriction holding the pharmacophoric elements in a defined relative orientation.

To gain insight into the relationship between the stereochemistry and the biological activity of our *C*‐furanosidic LpxC inhibitors, we started to vary the configuration of the four stereocenters of these *C*‐furanosides. Previously, we reported on the chiral pool synthesis and biological evaluation of 12 of the 16 possible stereoisomers. We accomplished the synthesis of all eight 3,4‐*cis*‐configured stereoisomers, of which the (3*R*,4*S*)‐configured dihydroxytetrahydrofuran derivatives **9**, **10**, **11**, and **12** were accessed from d‐mannose [28, 33], whereas their (3*S*,4*R*)‐configured enantiomers *ent*‐**9** and *ent*‐**10**, as well as *ent*‐**11** and *ent*‐**12**, were synthesized from d‐ribose and d‐gluono‐γ‐lactone, respectively [34]. Of the 3,4‐*trans*‐configured dihydroxytetrahydrofuran derivatives, the 2*r*,3*c*,4*t*‐configured stereoisomers **13**, *ent*‐**13**, **14**, and *ent*‐**14** could be obtained in chiral pool syntheses starting from d‐ and l‐xylose [36].

To complete the stereochemical series of our *C*‐furanosidic LpxC inhibitors, in this article, we report on the synthesis of the pending 2*r*,3*t*,4*c*‐configured stereoisomers **15**, *ent*‐**15**, **16**, and *ent*‐**16** exhibiting 2,3‐*trans*‐ as well as 3,4‐*trans*‐configuration. The biological evaluation of these four stereoisomers should clarify the stereochemical structure–activity relationships of this scaffold, which were refined by molecular docking and molecular dynamics simulations.

## 2 Results and Discussion

### 2.1 Chemistry

**Scheme 1:**
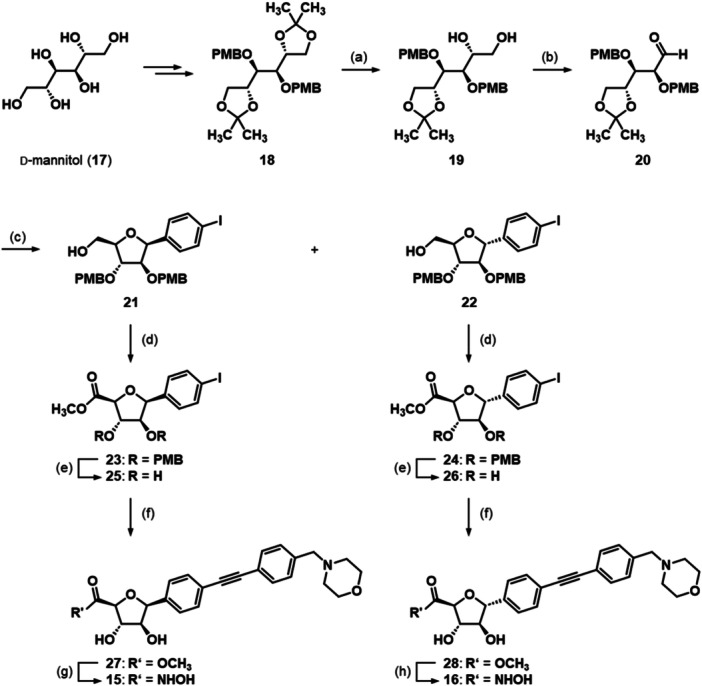
Reagents and conditions: (a) *p*‐TsOH·H_2_O, CHCl_3_/MeOH (10/1), rt, 39%; (b) NaIO_4_, THF/H_2_O (2/1), rt, 91%; (c) 1. 1,4‐diiodobenzene, *n*‐BuLi, THF, –78°C → rt, 2. MsCl, NEt_3_, CH_2_Cl_2_, 0°C, 3. CSA, CH_2_Cl_2_/MeOH (2/1), 0°C → rt, **21** 14%, **22** 16%; (d) 1. NMO·H_2_O, TPAP, CH_2_Cl_2_ (wet), rt, 2. CH_3_I, K_2_CO_3_, acetone, rt, **23** 71%, **24** 65%; (e) DDQ, H_2_O, CH_2_Cl_2_, rt, **25** 90%, **26** 84%; (f) 4‐(morpholinomethyl)phenylacetylene, Pd(PPh_3_)_4_, CuI, NEt_3_, THF, rt, **27** 42%, **28** 52%; (g) aq. NH_2_OH, KCN, THF/H_2_O (1/1), rt, 59%; (h) aq. NH_2_OH, KCN, THF/MeOH (1/1), rt, 75%.

The envisaged (2*S*,3*S*,4*S*)‐configured dihydroxytetrahydrofuran scaffold could be accessed by adapting a reaction sequence, which had been reported for the synthesis of (+)‐2,5‐*epi*‐goniothalesdiol (Scheme 1) [56]. Thus, 1,2:5,6‐di‐*O*‐isopropylidene‐3,4‐di‐*O*‐(4‐methoxybenzyl)‐d‐mannitol (**18**), which had been synthesized from d‐mannitol (**17**) according to reported procedures [57, 58], was subjected to a selective cleavage of one acetonide moiety, yielding diol **19**, which, upon treatment with sodium periodate, gave protected d‐arabinose derivative **20**. Aldehyde **20** was reacted with 4‐iodophenyllithium, yielding an inseparable mixture of diastereomeric alcohols, which were mesylated and subsequently treated with camphorsulfonic acid, resulting in the cleavage of the remaining acetonide moiety and an ensuing cyclization to establish the tetrahydrofuran ring with the desired stereochemistry. The resulting anomeric *C*‐aryl furanosides **21** and **22** could be chromatographically separated, and the hydroxymethyl group of each stereoisomer was transformed into an ester moiety *via* a TPAP‐catalyzed oxidation using NMO·H_2_O as co‐oxidant [59], followed by an alkylation of the intermediate carboxylic acids with methyl iodide and potassium carbonate. Subsequently, the *para*‐methoxybenzyl protecting groups of esters **23** and **24** were cleaved with DDQ, and the resulting diols **25** and **26** were subjected to Sonogashira couplings with 4‐(morpholinomethyl)phenylacetylene [28] to yield diphenylacetylene derivatives **27** and **28**. The desired (2*S*,3*S*,4*S*)‐configured hydroxamic acids **15** and **16** were finally obtained *via* potassium cyanide‐catalyzed aminolyses of esters **27** and **28** with hydroxylamine [53, 60].

**Scheme 2:**
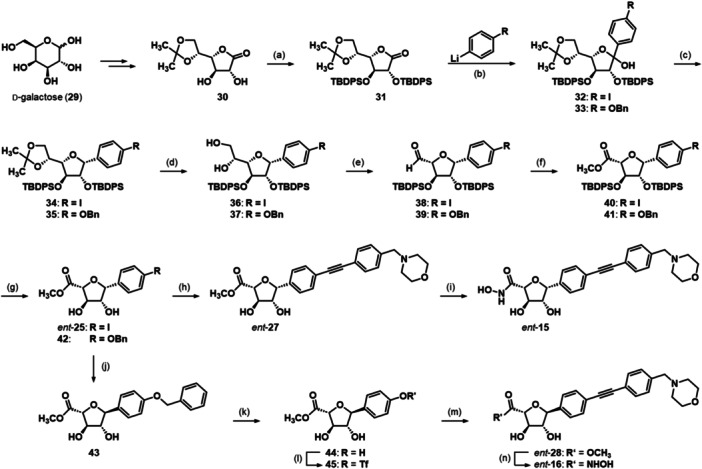
Reagents and conditions: (a) TBDPSCl, imidazole, CH_2_Cl_2_, rt, 96%; (b) THF, –78°C, **32** 100%, **33** 64%; (c) BF_3_·OEt_2_, Et_3_SiH, CH_2_Cl_2_, –40°C, **34** 75%, **35** 85%; (d) *p*‐TsOH·H_2_O, MeOH, rt, **36** 60%, **37** 47%; (e) NaIO_4_, H_2_O, THF, rt, **38** 84%, **39** 94%; (f) 1. NMO·H_2_O, TPAP, CH_2_Cl_2_, rt, 2. CH_3_I, K_2_CO_3_, acetone, rt, **40** 60%, **41** 72%; (g) 1. TBAF·3H_2_O, THF, 60°C, 2. *p*‐TsOH·H_2_O, MeOH, 60°C, *ent*‐**25** 83%, **42** 82%; (h) *ent*‐**25**, 4‐(morpholinomethyl)phenylacetylene, Pd(PPh_3_)_4_, CuI, NEt_3_, THF, rt, 35%; (i) aq. NH_2_OH, KCN, THF/MeOH (1/1), rt, 75%; (j) **42**, Er(OTf)_3_, ACN, 120°C, 20%; (k) Et_3_SiH, Pd/C, MeOH, rt, 68%; (l) phenyl triflimide, NEt_3_, CH_2_Cl_2_, 0°C → rt, 69%; (m) 1. trimethylsilylacetylene, Pd(PPh_3_)_4_, CuI, NEt_3_, ACN, 60°C, 2. TBAF·3H_2_O, CHCl_3_, THF, rt, 3. 4‐(4‐iodobenzyl)morpholine, Pd(PPh_3_)_4_, CuI, NEt_3_, ACN, rt, 14%; (n) aq. NH_2_OH, KCN, DMSO, rt, 40%.

For the synthesis of the respective (2*R*,3*R*,4*R*)‐configured enantiomers *ent*‐**15** and *ent*‐**16**, d‐galactose (**29**) served as chiral starting material, which was converted into 5,6‐*O*‐isopropylidene‐d‐galactono‐1,4‐lactone (**30**) *via* reported procedures (Scheme 2) [61]. After the protection of the hydroxyl groups of diol **30** with *tert*‐butyldiphenylsilyl chloride, the obtained lactone **31** was reacted with 4‐iodophenyllithium and 4‐(benzyloxy)phenyllithium, which were generated *in situ* from 1,4‐diiodobenzene and 4‐benzyloxybromobenzene with *n*‐butyllithium, to yield hemiketals **32** and **33**, respectively. Subsequently, hemiketals **32** and **33** were diastereoselectively reduced with triethylsilane in the presence of boron trifluoride diethyl etherate, yielding *C*‐aryl furanosides **34** and **35**, which exhibit (*R*)‐configuration at the anomeric center. After the cleavage of the acetonide moiety, the resulting diols **36** and **37** were subjected to a glycol cleavage with sodium metaperiodate to give aldehydes **38** and **39**. Aldehydes **38** and **39** were subsequently oxidized with TPAP and NMO, and the resulting carboxylic acids were transformed into esters **40** and **41** with methyl iodide and potassium carbonate. The removal of the *tert*‐butyldiphenylsilyl protective groups with tetrabutylammonium fluoride gave diols *ent*‐**25** and **42**, of which **42** could be crystallized yielding crystals being suitable for X‐ray crystal structure analysis, which confirmed the structure of *C*‐furanoside **42** (space group *P*2_1_ (# 4), *R*_1_ = 4.60%, *wR*_2_ = 11.13%) and particularly the configuration at the anomeric center (Supporting Information: Figure S1) by anomalous dispersion (with Flack and Hooft parameter of 0.00(9) and 0.01(8), respectively).

To obtain the (2*R*,3*R*,4*R*,5*R*)‐configured hydroxamic acid *ent*‐**15**, aryl iodide *ent*‐**25** was subjected to a Sonogashira coupling with 4‐(morpholinomethyl)phenylacetylene and a final aminolysis with hydroxylamine.

The (2*R*,3*R*,4*R*,5*S*)‐configured diastereomer *ent*‐**16** was synthesized from benzyl ether **42**, representing a phenylogous acetal, *via* an erbium(III) trifluoromethanesulfonate‐catalyzed epimerization [33], yielding a mixture of anomers, from which the desired stereoisomer **43** could be isolated. In the next reaction step, the benzyl group of *C*‐furanoside **43** was cleaved *via* a Pd/C‐induced catalytic transfer hydrogenation with triethylsilane [62]. The resulting phenol **44** was reacted with phenyl triflimide to yield triflate **45**. Subsequently, the lipophilic side chain was built up in a stepwise manner. After a Sonogashira coupling with trimethylsilylacetylene, the silyl protective group was removed with tetrabutylammonium fluoride, and the thereby obtained terminal alkyne was coupled with 4‐(4‐iodobenzyl)morpholine. Finally, the obtained diphenylacetylene derivative *ent*‐**28** was subjected to a potassium cyanide‐catalyzed aminolysis with hydroxylamine to yield hydroxamic acid *ent*‐**16**.

### 2.2 LpxC Inhibitory and Antibacterial Activities

To determine the inhibitory activities of the newly synthesized *C*‐furanosides toward *E. coli* LpxC, a fluorescence‐based enzyme assay was performed, using *E. coli* LpxC C63A, which is less susceptible to high Zn^2+^‐concentrations compared with the wild‐type enzyme [37, 41, 63].

All of the newly synthesized *C*‐glycosides were found to exhibit inhibitory activity toward *E. coli* LpxC C63A with *K*_i_ values in the single‐digit micromolar range (Table 1). When comparing the 2*r*,3*t*,4*c*‐configured stereoisomers, the (2*R*,3*R*,4*R*,5*R*)‐configured hydroxamic acid *ent*‐**15** (*K*_i_ = 2.3 µM) was found to be the most potent LpxC inhibitor of this series of compounds. Its enantiomer **15** (*K*_i_ = 3.6 µM) exhibited a less than twofold decreased inhibitory activity. Whereas the inversion of configuration in position 5 of the tetrahydrofuran ring of *ent*‐**15**, leading to compound *ent*‐**16** (*K*_i_ = 2.5 µM), caused only a slight decrease in inhibitory activity, hydroxamic acid **16** (*K*_i_ = 9.9 µM), the (2*S*,3*S*,4*S*,5*R*)‐configured C‐5 epimer of **15**, was found to be the least potent LpxC inhibitor of this series of stereoisomers, being about four times less active than its enantiomer *ent*‐**16**.

**Table 1:** Antibacterial and LpxC inhibitory activities of reported and newly synthesized *C*‐furanosides.

Compound	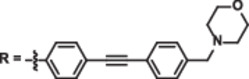		Zone of inhibition [mm]		MIC [µg · mL^−^^1^]		*IC*_50_ [µm]	*K*_i_ [μM]	
			***E. coli*** **BL21(DE3)**	***E. coli*** **D22**	***E. coli*** **BL21(DE3)**	***E. coli*** **D22**	***Ec*** **LpxC C63A**	***Ec*** **LpxC C63A**	***Pa*LpxC**
**9** [34]	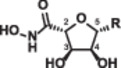	(2*R*,3*R*,4*S*,5*R*)	15.1 ± 1.5	25.7 ± 2.0	> 64	2	28.2 ± 9.0	3.6 ± 1.2	n.d.
*ent‐* **9** [34]	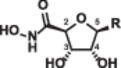	(2*S*,3*S*,4*R*,5*S*)	22.3 ± 1.4	28.3 ± 1.4	8	0.5	3.2 ± 1.0	0.41 ± 0.12	0.095 ± 0.064
**10** [33, 34]	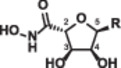	(2*R*,3*R*,4*S*,5*S*)	6.8 ± 1.0	22.8 ± 2.2	> 64	4	10 ± 1.0	1.3 ± 0.2	n.d.
*ent‐* **10** [34, 36]	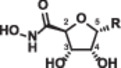	(2*S*,3*S*,4*R*,5*R*)	14.0 ± 1.0	21.0 ± 0.5	> 64	32	127.4 ± 15.6	16.2 ± 1.9	n.d.
**11** [29, 34]	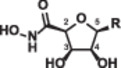	(2*S*,3*R*,4*S*,5*S*)	≤ 6	13.4 ± 1.8	> 64	8	> 200	> 25.4	n.d.
*ent‐* **11** [34]	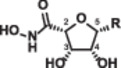	(2*R*,3*S*,4*R*,5*R*)	≤ 6	≤ 6	> 64	> 64	> 200	> 25.4	n.d.
**12** [33, 34]	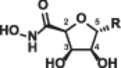	(2*S*,3*R*,4*S*,5*R*)	9.7 ± 0.5	21.4 ± 2.3	> 64	8	34 ± 10	4.3 ± 1.3	n.d.
*ent‐* **12** [34]	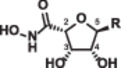	(2*R*,3*S*,4*R*,5*S*)	11.0 ± 0.8	19.7 ± 1.3	> 64	> 64	90 ± 35.6	11.4 ± 4.6	n.d.
**13** [36]	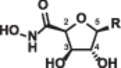	(2*S*,3*R*,4*R*,5*S*)	15.0 ± 1.5	22.3 ± 2.1	> 64	8	38.6 ± 19.4	4.89 ± 2.47	n.d.
*ent‐* **13** [36]	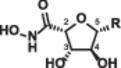	(2*R*,3*S*,4*S*,5*R*)	≤ 6	10.5 ± 1.2	> 64	64	151.0 ± 36.6	19.1 ± 4.65	n.d.
**14** [36]	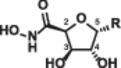	(2*S*,3*R*,4*R*,5*R*)	11.0 ± 2.4	17.5 ± 3.0	> 64	32	149.1 ± 30.7	19.0 ± 3.9	n.d.
*ent‐* **14** [36]	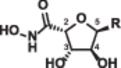	(2*R*,3*S*,4*S*,5*S*)	10.0 ± 1.7	18.8 ± 2.1	> 64	32	185.6 ± 51.5	23.5 ± 6.6	n.d.
**15**	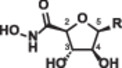	(2*S*,3*S*,4*S*,5*S*)	15.5 ± 1.8	> 30	64	2	28.0 ± 8.74	3.55 ± 1.11	1.39 ± 0.10
*ent‐* **15**	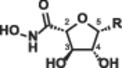	(2*R*,3*R*,4*R*,5*R*)	≤ 6	17.3 ± 1.5	> 64	16	17.9 ± 4.23	2.27 ± 0.54	2.02 ± 0.24
**16**	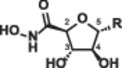	(2*S*,3*S*,4*S*,5*R*)	13.3 ± 1.9	26.7 ± 1.6	64	4	78.4 ± 45.2	9.94 ± 5.74	1.17 ± 0.13
*ent‐* **16**	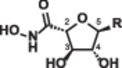	(2*R*,3*R*,4*R*,5*S*)	≤ 6	21.5 ± 0.7	> 64	8	19.7 ± 7.55	2.50 ± 0.96	0.41 ± 0.13

Abbreviation: n.d., not determined.

When comparing the inhibitory activities of the 2*r*,3*t*,4*c*‐configured *C*‐furanosides with those of the previously synthesized stereoisomers, the newly synthesized compounds range among the most potent inhibitors of the entire stereochemical series. Only the 3,4‐*cis*‐configured stereoisomers *ent*‐**9** and **10** outperform the 2*r*,3*t*,4*c*‐configured *C*‐furanosides **15**, *ent*‐**15**, and *ent*‐**16**, whereas **16** is additionally outperformed by *C*‐furanosides **9**, **12**, and **13**. Generally, (2*R*,3*R*)‐configuration, as seen in *ent*‐**15** and *ent*‐**16**, the most active compounds of this work, as well as **9** and **10**, appear to be beneficial for inhibitory activity, with all of these stereoisomers ranking among the most active compounds, being only outperformed by *ent*‐**9**.

Additionally, as *ent*‐**9** had shown antibacterial activity against *P. aeruginosa* [36], this stereoisomer as well as the newly synthesized *C*‐furanosides were tested for inhibitory activity toward LpxC of these bacteria using our previously described LC‐MS/MS‐based *P. aeruginosa* LpxC assay (Table 1) [64]. Even though *E. coli* LpxC had been reported to exhibit promiscuous susceptibility to structurally diverse inhibitors due to its conformational flexibility, enabling the enzyme to alter the size and shape of its active site upon inhibitor binding [65], all investigated *C*‐furanosides were found to inhibit the more rigid *P. aeruginosa* ortholog more potently than *E. coli* LpxC C63A. Whereas this trend was least pronounced in the case of *ent*‐**15** (factor 1.1), the determined *K*_i_ value of **16** against *P. aeruginosa* LpxC was lower by a factor of 8.5 than the one against *E. coli* LpxC C63A, the highest discrepancy of all investigated stereoisomers. Again, the (2*S*,3*S*,4*R*,5*S*)‐configured compound *ent*‐**9** was found to be the most potent LpxC inhibitor (*K*_i_ (*Pa*LpxC) = 95 nM) of all investigated stereoisomers. In case of the 2*r*,3*t*,4*c*‐configured *C*‐furanosides, different trends could be observed in the inhibitory activities against *P. aeruginosa* LpxC compared with the ones against *E. coli* LpxC C63A, with *ent*‐**16** (*K*_i_ (*Pa*LpxC) = 0.41 µM) being the most potent and *ent*‐**15** (*K*_i_ (*Pa*LpxC) = 2.0 µM) being the least potent inhibitor of the ortholog from *P. aeruginosa*.

To evaluate the antibacterial activity of the newly synthesized *C*‐furanosides, disc diffusion assays against *E. coli* BL21(DE3) (*lpxC* ^+^) and *E. coli* D22 (*lpxC101*) [66], a *lpxC* mutant showing reduced LpxC activity, were performed, and the minimum inhibitory concentrations (MICs) of the *C*‐furanosides against the same strains were determined in broth dilution tests (Table 1).

Surprisingly, the superior inhibitory activities of C‐5 epimers *ent*‐**15** and *ent*‐**16** toward *E. coli* LpxC C63A did not translate into higher antibacterial activities against the two *E. coli* strains compared with the respective enantiomers **15** and **16**. Whereas all 2*r*,3*t*,4*c*‐configured *C*‐furanosides showed antibacterial activity against the defective *E. coli* D22 strain (MIC ≤ 16 µg ∙ mL^–1^), only stereoisomers **15** and **16** were able to inhibit the growth of *E. coli* BL21(DE3). With respect to antibacterial activity against *E. coli* BL21(DE3) and *E. coli* D22, the (2*S*,3*S*,4*S*,5*S*)‐configured compound **15**, the antibacterially most potent compound of the series of 2*r*,3*t*,4*c*‐configured *C*‐furanosides, was only outperformed by its C‐4 epimer *ent*‐**9**, which exhibits the highest antibacterial as well as LpxC inhibitory activities of all 16 stereoisomeric *C*‐furanosides.

To elucidate whether efflux pumps account for the observed discrepancy between LpxC inhibition and antibacterial activity among the enantiomeric pairs, the MIC values of compounds **15** and *ent*‐**15** against *E. coli* ATCC 35218 were determined in the absence and the presence of the efflux pump inhibitor phenylalanine‐arginine‐β‐naphthylamide (PAβN) (Table 2). However, for both enantiomers, no change in the MIC value was observed in the presence of PaβN.

**Table 2:** MIC values of **15**, *ent*‐**15**, and *ent*‐**9** against *E. coli* ATCC 35218 and *P. aeruginosa* ATCC 27853 in the absence (–) and presence (+) of the efflux pump inhibitor PAßN (64 µg · mL^–^^1^).

Strain	MIC [µg · mL^–^^1^]		
	15	*ent*‐15	*ent*‐9
*Escherichia coli* ATCC 35218 (‐PAßN)	32	64	8
*E. coli* ATCC 35218 (+PAßN)	32	64	4
*Pseudomonas aeruginosa* ATCC 27853 (‐PAßN)	> 64	> 64	16 [36]
*P. aeruginosa* ATCC 27853 (+PAßN)	32	64	2 [36]

Exhibiting inhibitory activity against *P. aeruginosa* LpxC, enantiomers **15** and *ent*‐**15** were additionally tested for antibacterial activity against *P. aeruginosa* ATCC 27853 (Table 2). In the absence of PAβN, both compounds were unable to inhibit bacterial growth up to a concentration of 64 µg · mL^–1^, whereas the efflux pump inhibitor decreased the MIC values of **15** and *ent*‐**15** to 32 µg · mL^–1^ and 64 µg · mL^–1^, respectively.

These observations are in line with the results obtained for *ent*‐**9**, for which in the presence of PAßN a reduction of the MIC value by only one dilution step was observed in the experiments with *E. coli* ATCC 35218, whereas a reduction by three serial dilution steps was measured in the case of *P. aeruginosa* ATCC 27853 [36].

### 2.3 Molecular Docking

**Figure 4:**
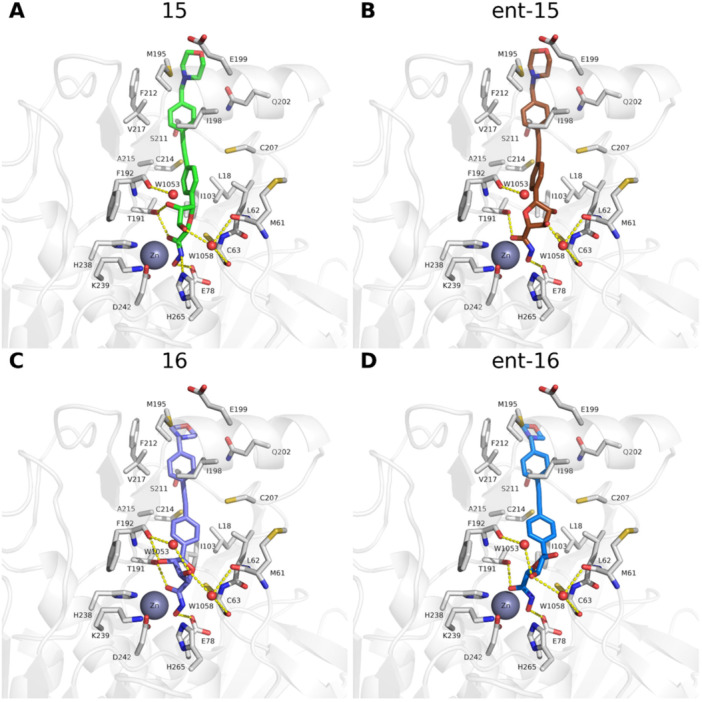
Generated docking poses for **15** (A), *ent‐* **15** (B), **16** (C), and *ent‐* **16** (D). The inhibitors and surrounding pocket residues are shown with sticks, the zinc ion is visualized as gray sphere. Hydrogen bonds are depicted with yellow dashed lines.

To rationalize the observed relationship between the stereochemistry and the inhibitory activity toward *E. coli* LpxC C63A of the synthesized *C*‐glycosidic LpxC inhibitors, the binding modes of all 16 stereoisomeric *C*‐furanosides were predicted by Glide docking to the available crystal structure of *E. coli* LpxC in complex with the inhibitor LPC‐053 (PDB ID 3PS3) [67]. In addition, we investigated whether different ligand–receptor interactions could explain the differences in binding affinity. At first, the docking protocol was validated by extracting LPC‐053 from the complex and re‐docking it into the protein structure, which resulted in a near‐native pose with an RMSD of 0.2 Å. Similar to the co‐crystallized hydroxamic acid‐based inhibitor LPC‐053, a bidentate zinc chelation of the hydroxamic acid group was observable for all *C*‐glycosidic stereoisomers (Figure 4, Supporting Information: Figure S2). Thereby, hydrogen bonds were formed to T191 and E78. The diphenylacetylene moieties were placed inside the hydrophobic tunnel constituted by L18, L62, I198, C207, F212, and V217. The terminal morpholine rings protrude from the binding pocket and appear extensively solvent‐exposed. The studied molecules differ in the absolute configurations of the dihydroxytetrahydrofuran moiety. Regardless of the present configuration, the saturated ring system seems to offer enough flexibility for connecting the tunnel and zinc‐binding moieties without major strains. Potential binding modes for **11**, *ent‐* **11**, **13**, *ent‐* **13**, **14**, *ent‐* **14**, *ent‐* **9**, **10**, and *ent‐* **10** have already been proposed in previous publications [34, 35, 68]. Most of the herein presented binding modes resemble the previously published docking solutions (Supporting Information: Figure S2) with the exceptions of the inactive compound *ent‐* **11**, for which bidentate zinc chelation has not been described so far [34]. The predicted interactions of the newly prepared compounds **15**, *ent‐* **15**, **16**, and *ent‐* **16** are in accordance with the binding models of the previously published active stereoisomers (Figure 4). Besides the above‐described similarities in hydroxamate and hydrophobic interactions, the predicted binding poses among the stereoisomers mainly differ in the placement of the hydroxyl groups. Independent of the present absolute configurations, the hydroxyls of the active compounds mostly adopt solvent‐exposed positions where interactions with the conserved water molecules, M61 and C63 of the Insert I loop, or T191 and F192 of Insert II are possible. Significant deviations in the predicted binding modes are observable for the inactive compounds *ent‐* **11** and **11**. The hydroxyl groups of *ent‐* **11** were found to point toward the hydrophobic region marked by L18 (Supporting Information: Figure S2F). The hydroxyls of **11** appear buried in the opposite side of the binding pocket (Supporting Information: Figure S2E). Both placements lead to unfavorable or even absent interactions, which are potential reasons for the poor binding affinities.

**Figure 5:**
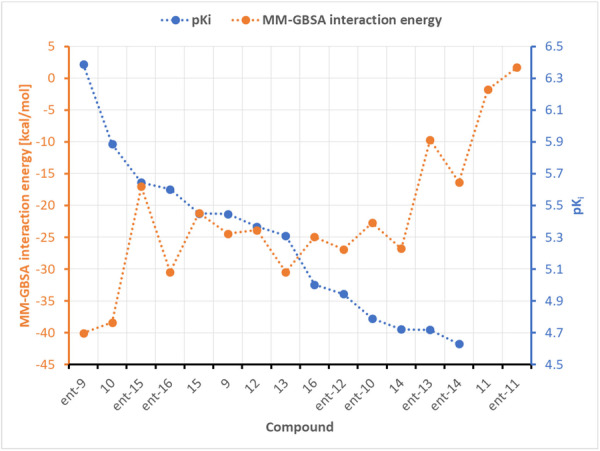
MM‐GBSA interaction energies calculated for all studied compounds, which appear sorted by the experimentally determined p*K*_i_ values.

To further differentiate between the compounds, MM‐GBSA (Molecular Mechanics, Generalized Born Surface Area) interaction energies were calculated using Schrödinger's Prime module. A weak correlation between the calculated interaction energies and the experimentally determined binding affinities was detectable (Figure 5). An exact prediction of binding affinities was not feasible using these calculations; however, a rough classification of isomers as (highly) active or inactive was possible. Notably, the most active compounds, *ent‐* **9** and **10**, show particularly favorable interaction energies (<−35 kcal/mol), while the inactive compounds, **11** and *ent‐* **11**, reveal unfavorable energy values close to 0 kcal/mol (Table 3).

**Table 3:** Interactions between the functional groups of the simulated compounds (green) and the binding site residues (orange). Only reproducible and persistent water‐mediated (blue) and direct hydrogen bond (red) contacts are shown. For comparison, the experimentally determined p*K*_i_ and p*IC*_50_ values as well as the calculated MM‐GBSA interaction energies are listed.

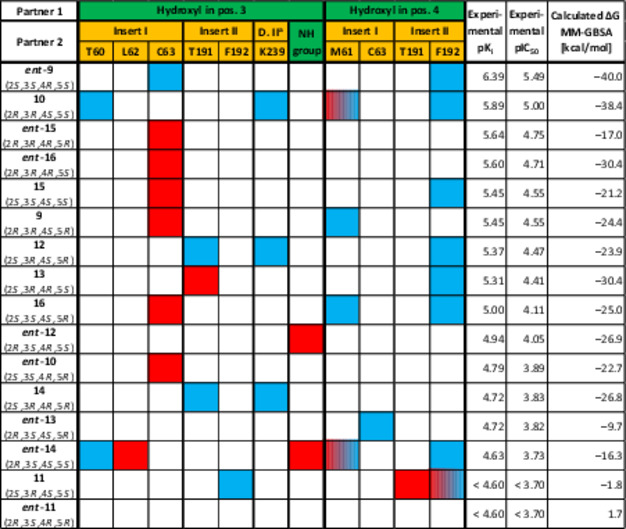

^a^ Domain II.

### 2.4 Molecular Dynamics (MD) Simulations

To evaluate the stability of the generated binding poses and to study the dynamic protein‐ligand interactions, MD simulations were carried out. The co‐crystallized ligand LPC‐053 and the in‐house compounds were subjected to two independent replicas of MD simulation, each of 50 ns length. First of all, ligand RMSF values were calculated (Supporting Information: Figure S3) to identify potential highly fluctuating ligand parts. Elevated fluctuations were only observed for the solvent‐exposed ligand moieties. In addition, the phenyl rings adjacent to the tetrahydrofuran moieties occasionally showed rotational movements, resulting in slightly elevated RMSF values. Due to the high fluctuations of the extensively solvent‐exposed morpholine rings, ligand RMSD calculations were carried out, neglecting the respective atoms. The obtained RMSD values (Supporting Information: Figure S4) converge quickly throughout the MD simulations and do not show significant fluctuations, which confirms the overall stability of the generated poses.

Since the major difference between the binding poses lies within the placement of the hydroxyl groups connected to differently configured stereocenters, we focused on analyzing the interactions formed by these functional groups throughout the generated trajectories. Thereby, only hydrogen bonds and water‐mediated contacts were considered that occurred in both independent MD runs with occupancies > 20%, respectively. In this way, distinct interaction patterns (Table 3) were obtained for all simulated compounds, which were helpful to generate hypotheses explaining the differences in experimentally determined binding affinities. Additionally, the solvent exposure of the inhibitors' hydroxyl groups was quantified throughout the MD simulations to further support these hypotheses (Supporting Information: Figure S5).

The most active compounds, *ent‐* **9** and **10**, maintain water‐mediated contacts with the loop of Insert I and preserve the native water bridge to F192. The active compounds *ent‐* **15**, *ent‐* **16**, **15**, **9**, **16**, and *ent‐* **10** are stabilized by hydrogen bonds to the backbone of C63. Meanwhile, the water bridge to F192 was not maintained by all of the mentioned stereoisomers. Hence, a stable water bridge in addition to the hydrogen bond to C63 does not seem to be beneficial regarding inhibitory activity. The hydroxyl in position 3 of **12** and **14** does not reveal any significant interactions with the loop of Insert I. Instead, water‐mediated contacts are formed with T191 and K239. The increased binding affinity of **12** compared with **14** might be explained by an additional water‐mediated contact between the hydroxyl in position 4 and F192. The hydroxyl in position 3 of *ent‐* **12** and *ent‐* **14** tends to form an intramolecular hydrogen bond to the hydroxamate NH group. Meanwhile, the hydroxyls of *ent‐* **12** do not form any additional intermolecular interactions with the binding pocket. Conversely, *ent‐* **14** interacts with both Insert I and II in multiple ways. However, *ent‐* **14** induced a conformational shift of the Insert I loop, which made the backbone of L62 available for hydrogen bonding. Overall, the interaction pattern does not seem to be favorable regarding the corresponding experimental data. The inactive compounds **11** and *ent‐* **11** deviate in the protein–ligand interaction pattern. The hydroxyls of *ent‐* **11** are unavailable to form any persistent hydrogen bonds or water bridges to the binding site residues. In contrast, the hydroxyls of **11** intensively interact with T191 and F192. However, these interactions require the displacement of the conserved water molecule, which might be unfavorable. **11** was found to uniquely form water‐mediated contacts to F192 *via* the hydroxyl in position 3.

The MD study leads to the conclusion that stereoisomers allowing rather solvent‐exposed hydroxyl positions benefit from favorable interactions with the binding pocket residues. Thereby, water‐mediated contacts between the hydroxyl in position 3 and the loop of Insert I, as well as the maintenance of the conserved water bridge to F192 by the hydroxyl in position 4, seem to be advantageous for achieving favorable binding affinities.

### 2.5 *In Vitro* ADMET Properties

The *in vitro* ADMET profile of the newly synthesized *C*‐furanosides was investigated by evaluating their metabolic stability using murine and human liver microsomes as well as plasma and by analyzing their plasma protein binding (Table 4). Additionally, the *in vitro* ADMET profile of *ent‐* **9**, the most potent LpxC inhibitor among the investigated *C*‐furanosides, as well as several selected stereoisomers, was evaluated. All surveyed *C*‐furanosides showed excellent metabolic stability with both species, mouse and human, with half‐lives > 60 min and in consequence low clearance (CL_int_ < 23 μL/min/mg protein). In plasma, all compounds were found to be very stable (*t*_1/2_ > 240 min), with the exception of the (2*R*,3*R*,4*R*,5*S*)‐configured *C*‐furanoside *ent‐* **16**, which degraded slowly (*t*_1/2_ = 115.5 min) in mouse plasma.

**Table 4:** Microsomal metabolic stability, calculated hepatic clearance, plasma stability, and plasma protein binding (PPB) of selected reported and the newly synthesized *C*‐furanosides.

Compound	Microsomal metabolic stability				Calculated hepatic clearance	Plasma stability		PPB	
	mouse		human		mouse	mouse	human	mouse	human
	*t*_1/2_ [min]	Cl_int_ [µL/min/mg protein]	*t*_1/2_ [min]	Cl_int_ [µL/min/mg protein]	Cl_h,m_ [mL/min/kg]	*t*_1/2_ [min]	*t*_1/2_ [min]	[%]	[%]
**9**	> 60	< 23	> 60	< 23	1.25	> 240	> 240	98.6 ± 1.8	99.1 ± 0.4
*ent‐* **9**	> 60	< 23	> 60	< 23	6.02	> 240	> 240	93.0 ± 6.7	98.1 ± 0.9
*ent‐* **10**	> 60	< 23	> 60	< 23	2.66	> 240	> 240	99.7 ± 0.2	98.4 ± 0.8
*ent‐* **12**	> 60	< 23	> 60	< 23	3.86	> 240	> 240	95.6 ± 1.4	97.2 ± 2.1
**13**	> 60	< 23	> 60	< 23	0.54	> 240	> 240	99.4 ± 0.9	98.4 ± 1.0
**15**	> 60	< 23	> 60	< 23	2.13	> 240	> 240	97.6 ± 1.9	98.4 ± 2.0
*ent‐* **15**	> 60	< 23	> 60	< 23	2.48	> 240	> 240	97.2 ± 2.8	95.8 ± 2.1
**16**	> 60	< 23	> 60	< 23	0.90	> 240	> 240	99.0 ± 1.3	97.3 ± 0.2
*ent‐* **16**	> 60	< 23	> 60	< 23	1.78	115.5	> 240	98.0 ± 2.2	98.2 ± 2.5

Additionally, the plasma protein binding of the compounds was determined, revealing high plasma protein binding for all investigated *C*‐furanosides with unbound fractions ranging from 1% to 2%. Taking into account the high metabolic stability, we calculated the hepatic clearance for the mouse *in vivo*, Cl_h,m_, for the different *C*‐furanosides. Based on the microsomal data in conjunction with the high plasma protein binding, low clearance *via* microsomes *in vivo* is expected. With respect to pharmacokinetic behavior *in vivo*, renal clearance might dominate.

**Figure 6:**
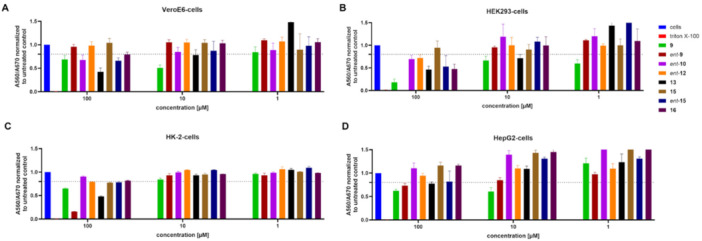
Cytotoxicity profiling of selected *C*‐furanosides in VeroE6 (A), HEK‐293 (B), HK‐2 (C), and HepG2 (D) cells at concentrations 1, 10, and 100 µM.

To differentiate the *C*‐furanosides further, we conducted *in vitro* cytotoxicity tests at three different concentrations, that is, 1, 10, and 100 µM, in four different cell lines. Thereby, we chose VeroE6 cells to assess general toxicity risks, as well as HepG2 cells as a marker for potential liver toxicity, and HEK293 as well as HK‐2 cells as marker cell lines for potential kidney toxicity risks. Apart from **9** and **13**, all other tested compounds did not exhibit cytotoxicity in all four tested cell lines at concentrations of 1 and 10 µM (Figure 6). In VeroE6 cells as a marker for general toxicity risks, only *C*‐furanosides **9**, *ent*‐**10**, **13**, and *ent*‐**15** exhibited toxicity at 100 µM (Figure 6A). However, in HEK293‐cells (Figure 6B), all *C*‐furanosides, except **15**, showed increased toxicity at 100 µM, whereas in the tubular HK‐2 cells, that only applied to *C*‐furanosides **9**, *ent*‐**9**, and **13** (Figure 6C). By contrast, in HepG2‐cells only **9** and *ent*‐**9** exhibited toxicity at high concentrations of 100 µM, suggesting a favorable safety profile for the other *C*‐furanosides with respect to liver toxicity (Figure 6D).

## 3 Summary and Conclusions

To provide a full picture of the effects of stereochemistry on the biological activity of our *C*‐furanosidic LpxC inhibitors, the pending 2*r*,3*t*,4*c*‐configured stereoisomers were synthesized in chiral pool syntheses and tested for LpxC inhibitory and antibacterial activity.

The biological evaluation of the newly synthesized *C*‐furanosides revealed that the compounds range among the most potent stereoisomers of the entire series of 16 stereoisomeric *C*‐furanosides with respect to inhibitory activity toward *E. coli* LpxC C63A. While exhibiting superior inhibitory activities toward *E. coli* LpxC C63A, *C*‐furanosides *ent*‐**15** (*K*_i_ = 2.3 µM) and *ent*‐**16** (*K*_i_ = 2.5 µM) were outperformed by their enantiomers **15** (*K*_i_ = 3.6 µM) and **16** (*K*_i_ = 9.9 µM) with respect to antibacterial activity against *E. coli* BL21(DE3) and *E. coli* D22. This inconsistency might result from differences in permeability, efflux, uptake, medium binding, or intracellular stability. To investigate whether the involvement of efflux pumps accounts for the observed discrepancy between LpxC inhibition and antibacterial activity of the enantiomeric pairs, the MIC values of **15** and *ent*‐**15** against *E. coli* ATCC 35218 were determined in the presence of PAβN, an inhibitor of efflux pumps belonging to the resistance‐nodulation‐division (RND) type (such as AcrAB‐TolC in *E. coli* and MexCD‐OprJ in *P. aeruginosa*). However, no change in the MIC values was observed compared with the experiments without the efflux pump inhibitor, indicating that the efflux pump AcrAB‐TolC has a minor effect on the antibacterial activity of the *C*‐furanosides against *E. coli* ATCC 35218.

Having all 16 stereoisomeric *C*‐furanosides in hand, molecular docking studies were performed to rationalize the obtained structure–activity relationships. These studies indicated that despite similarities in hydroxamate and hydrophobic interactions, the predicted binding poses of the stereoisomers differ in the placement of the hydroxyl groups.

An MD study focusing on the interactions of the hydroxyl groups of the stereoisomeric *C*‐furanosides made evident that a conserved water‐bridge network plays an essential role in inhibitor binding. High biological activity is to some extent correlated with solvent‐exposed hydroxyl groups (particularly position 3 hydroxyl, Supporting Information: Figure S5) and flexible, water‐mediated interactions involving both Insert regions (Table 2). The most favorable interaction pattern includes water‐mediated contacts between the position 3 hydroxyl and Insert I, and between the position 4 hydroxyl and Insert II, while preserving conserved water molecules. In contrast, excessive inter‐ and intramolecular hydrogen bonding tends to reduce activity, which can be traced back to over‐constrained binding conformations or displacement of conserved water molecules. Nevertheless, a stable hydrogen bond between the position 3 hydroxyl and C63 can still result in moderate to high target affinity, as observed for the newly characterized compounds *ent*‐**15**, *ent*‐**16**, **15**, and **16**. Generally, interactions involving the hydroxyl group in position 4 appear more variable and less decisive with regard to biological activity than those involving the hydroxyl group in position 3.

Analysis of stereochemistry shows no universal preference for a single stereocenter configuration. Instead, biological activity depends on the cooperative 3D arrangement of all stereocenters, as they are located in direct proximity to each other within the dihydroxytetrahydrofuran ring. A few configuration patterns are noticeable that correlate with binding affinity. For example, matching absolute configurations at positions 2 and 3 (i.e., (2*S*,3*S*) or (2*R*,3*R*)) generally enhances activity. The resulting 2,3‐*trans* arrangement promotes effective zinc chelation of the hydroxamic acid group while orienting the hydroxyl group in position 3 toward the solvent, where it participates in (water‐mediated) interactions with Insert I. The absolute configuration at position 4 alone is not decisive with regard to biological activity but can influence hydroxyl solvent exposure (e.g., *ent*‐**15** vs. **9**, Supporting Information: Figure S5). The *S*‐configuration at position 5 is slightly preferred, as suggested by the two most active compounds (*ent*‐**9** and **10**), and possibly improves the fit of the diphenylacetylene moiety within the binding tunnel. Overall, the (2*R*,3*R*,5*S*)‐ and (2S,3*S*,5*S*)‐configured compounds are the most promising candidates for further optimization.

Whereas the configuration of the four stereocenters of the *C*‐furanosides exerts a strong influence on the antibacterial and LpxC inhibitory activity of the compounds, little to no influence of stereochemistry on the compounds' metabolic stability was observed. All investigated stereoisomers were found to exhibit good *in vitro* metabolic stability in the presence of mouse and human microsomes and, with the exception of *ent‐* **16**, to be very stable in human and mouse plasma. One can speculate that the different stability behavior observed in mouse and human for *ent*‐**16** might be attributed to different types of enzymes present in the respective plasma. Additionally, the projected hepatic clearance in the mouse *in vivo* based on the microsomal and plasma protein binding data suggests low hepatic clearance. With respect to pharmacokinetic properties, the higher protein binding might also contribute to less renal clearance. However, this has to be determined experimentally and can only be speculated about.

All *C*‐furanosides exhibited a high plasma protein binding with unbound fractions ranging from 1% to 2%, which is in the range of other antibacterials on the market. Only the unbound fraction will exert an effect *in vivo*. It is suspected that under infection conditions, protein binding properties might change slightly, as efficacy has been observed, for example, for cystobactamids despite nearly 100% protein binding [69, 70]. Nevertheless, increasing the unbound fraction further is highly desired for next‐generation *C*‐furanosides.

*In vitro* cytotoxicity tests in four different cell lines, namely VeroE6, HepG2, HEK293, and HK‐2 cells, were conducted, revealing the most favorable profiles for *C*‐furanoside **15**. Thus, the cytotoxicity profiling provided another stratification matrix for further development of our *C*‐furanosides. However, it has to be emphasized that concentrations as high as 100 µM might not be observed *in vivo* during treatment, so that the cytotoxicity observed for the other *C‐*furanosides at this concentration is not a red flag for further development.

Based on the evaluation of all 16 stereoisomeric *C*‐furanosides, the promising LpxC inhibitory and antibacterial activities of some of the stereoisomers, particularly of *ent*‐**9**, in combination with their excellent metabolic stability, render these compounds valuable starting points for further development. However, cytotoxicity needs to be monitored closely during further optimization steps, in particular with respect to the cytotoxicity seen in kidney (HEK293) and kidney tubular cells (HK‐2) for compound *ent*‐**9**.

## 4 Experimental

### 4.1 Chemistry

#### 4.1.1 General Remarks

4.1.1.1

All experiments involving water‐ or air‐sensitive compounds were carried out under anhydrous conditions (N_2_ atmosphere). Reagents were purchased from various suppliers and were used without further purification, unless otherwise noted. Anhydrous solvents were purchased from Acros Organics (extra dry over molecular sieves). Solvents for flash column chromatography were purchased in technical grade and distilled before use. Ultrapure water for reversed‐phase chromatography was purified using a Sartorius arium pro system (Sartopore 0.2 μm, UV). ACN for reversed‐phase chromatography was purchased from VWR (HPLC grade). Flash column chromatography on silica gel was performed using Macherey‐Nagel silica gel 60 M (0.040–0.063 mm). Parentheses include eluent and *R*_*f*_ value. Thin‐layer chromatography was performed on Macherey‐Nagel precoated TLC sheets (ALUGRAM Xtra SIL G/UV_254_). Visualization was achieved by heat‐staining using a cerium molybdate dipping bath [Ce(SO_4_)_2_ (1.8 g), (NH_4_)_6_Mo_7_O_24_·4H_2_O (45 g), conc. H_2_SO_4_ (45 g), H_2_O (900 mL)]. Automatic reversed‐phase flash column chromatography was performed using Biotage SNAP Ultra C18 columns on an Isolera One (Biotage). Product‐containing fractions were combined and lyophilized using a Christ Alpha 2‐4 LDplus freeze‐dryer. Melting points were measured with a Büchi Melting Point M‐565 and are uncorrected. Optical rotation α [deg] was determined with a P8000 polarimeter (A. Krüss Optronic GmbH); path length 1 dm, wavelength 589 nm (sodium D line); the unit of the specific rotation ${\left[\alpha \right]}_{D}^{20}$ [deg · mL · dm^−1^ · g^−1^] is omitted; the concentration of sample c [mg · mL^‐1^] and the solvent used is given in brackets. IR spectra were recorded on a Bruker Alpha FT‐IR Platinum ATR spectro‐photometer. NMR spectra were recorded at ambient temperature on Bruker Avance I 400, DRX 500, and Avance III 600 instruments. High‐resolution mass spectrometry was performed using an Agilent 6224 ESI‐TOF instrument *via* a flow injection analysis in 50:50 water + 0.1% formic acid/acetonitrile + 0.1% formic acid at a flow rate of 0.3 mL/min *via* electrospray ionization. HPLC methods for the determination of product purity: Method 1: VWR Hitachi equipment; UV/VIS detector: 5420; autosampler: 5260; pump: 5160; column: LiChrospher 60 RP‐select B (5 μm); LiChroCART 250‐4 mm cartridge; flow rate: 1.00 mL/min; injection volume: 5.0 µL; detection at λ = 210 nm for 30 min; solvents: A: water with 0.05% (V/V) trifluoroacetic acid, B: acetonitrile with 0.05% (V/V) trifluoroacetic acid; gradient elution: (A%): 0–4 min: 90%, 4–29 min: gradient from 90% to 0%, 29–31 min: 0%, 31–31.5 min: gradient from 0% to 90%, 31.5–40 min: 90%; data were collected and evaluated by Chromaster software. Method 2: VWR Hitachi equipment; UV/VIS detector: 5420; autosampler: 5260; pump: 5160; column: phenomenex Gemini 5 µm C6‐Phenyl 110 Å; LC Column 250 × 4.6 mm; flow rate: 1.00 mL/min; injection volume: 5.0 µL; detection at λ = 254 nm for 20 min; solvents: A: acetonitrile/10 mM ammonium formate = 10/90 with 0.1% formic acid, B: acetonitrile/10 mM ammonium formate = 90/10 with 0.1% formic acid; gradient elution: (A%): 0–5 min: 100%, 5–12 min: gradient from 100% to 0%, 12–20 min: 0%, 20–22 min: gradient from 0% to 100%, 22–30 min: 100%; data were collected and evaluated by Chromaster software. Method 3: VWR Hitachi equipment; UV/VIS detector: 5420; autosampler: 5260; pump: 5160; column: LiChrospher 60 RP‐select B (5 μm); LiChroCART 250‐4 mm cartridge; flow rate: 1.00 mL/min; injection volume: 5.0 µL; detection at λ = 210 nm for 40 min; solvents: A: water with 0.05% (V/V) trifluoroacetic acid, B: acetonitrile with 0.05% (V/V) trifluoroacetic acid: gradient elution: (A%): 0–4 min: 90%, 4–29 min: gradient from 90% to 0%, 29–41 min: 0%, 41–41.5 min: gradient from 0% to 90%, 41.5–50 min: 90%; data were collected and evaluated by Chromaster software. The single crystal X‐ray experiment of **42** was performed using a SuperNova four‐circle diffractometer in Kappa geometry with 50 W Cu microfocus tube using *K*_α_ radiation (λ = 1.54184 Å), an Atlas CCD detector (Rigaku Oxford Diffraction), and a Cryostream 700 Plus cooler (100 K–300 K, Oxford Cryosystems Ltd). Data collection, cell refinement, data reduction, and absorption correction were done using CrysAlis (Rigaku Oxford Diffraction). A suitable crystal (dimension: 0.04 × 0.14 × 0.20 mm^3^) was immersed in PAO 100/100 oil and fixed on the goniometer with a Hampton Research CryoLoop. Then the crystal was centered under a ~ 100 K cold nitrogen flow and measured at a temperature of 99.9(2) K. Using Olex2 [71], the structure was solved with the SHELXT [72] structure solution program using Intrinsic Phasing and refined with the SHELXL [73] refinement package using Least Squares minimization. Missing secondary atom sites were located from the difference Fourier map. Non‐hydrogen atoms were refined using individual, anisotropic temperature factors. Heteroatom‐bound hydrogen atoms were freely refined in their positions. Carbon atom‐bound hydrogen atoms were positioned geometrically and refined riding on their respective parent atoms. *U*_iso_(H) was fixed at 1.5 (OH, CH_3_) or 1.2 (all other H atoms) of the parent atom's *U*_eq_ isotropic displacement parameter. The fully refined data were reviewed using PLATON [74]. Supplementary crystallographic data for compound **42** can be obtained free of charge from the Cambridge Crystallographic Data Center at www.ccdc.cam.ac.uk under the deposition number CCDC 2516138.

#### 4.1.2 Synthesis of (2*R*,3*R*,4*R*)‐4‐[(*R*)‐2,2‐dimethyl‐1,3‐dioxolan‐4‐yl]‐3,4‐bis[(4‐methoxybenzyl)oxy]butane‐1,2‐diol (**19**)

*p*‐Toluenesulfonic acid monohydrate (360 mg, 1.9 mmol) was added to a solution of **18** (19 g, 38 mmol) in a mixture of chloroform and methanol (10/1, 380 mL). After stirring the reaction mixture at ambient temperature for 13 h, a saturated aqueous solution of NaHCO_3_ was added, and the aqueous phase was extracted with dichloromethane (3×). The combined organic layers were dried (Na_2_SO_4_), filtered, and the solvent was removed *in vacuo*. The residue was purified by flash column chromatography (petroleum ether/ethyl acetate = 1/2 → 0/1) to give **19** as a colorless oil (6.8 g, 15 mmol, 39%). TLC: *R*_*f*_ = 0.21 (petroleum ether/ethyl acetate = 1/2); ${\left[\alpha \right]}_{D}^{20}$ = +18.2 (10, chloroform); ^1^H NMR (500 MHz, DMSO‐*d*_*6*_): *δ* [ppm] = 1.28 (s, 3H, C(C*H*_3_)_2_), 1.34 (s, 3H, C(C*H*_3_)_2_), 3.47–3.56 (m, 2H, CHCHCHC*H*_2_OH (1H), CHC*H*CHCH_2_OH), 3.60–3.69 (m, 2H, CHCHCHC*H*_2_OH (1H), CHCHC*H*CH_2_OH), 3.73 (s, 6H, OC*H*_3_), 3.74–3.78 (m, 1H, 5′‐H_1,3‐dioxolan‐4‐yl_), 3.86 (dd, *J* = 6.4/2.1 Hz, 1H, C*H*CHCHCH_2_OH), 3.96 (dd, *J* = 7.9/6.4 Hz, 1H, 5′‐H_1,3‐dioxolan‐4‐yl_), 4.19 (q, *J* = 6.5 Hz, 1H, 4′‐H_1,3‐dioxolan‐4‐yl_), 4.44 (d, *J* = 10.6 Hz, 1H, 3‐OC*H*_2_Ar), 4.49 (d, *J* = 10.6 Hz, 1H, 3‐OC*H*_2_Ar), 4.52–4.56 (m, 2H, 1‐O*H*, 4‐OC*H*_2_Ar (1H)), 4.58 (d, *J* = 10.7 Hz, 1H, 4‐OC*H*_2_Ar), 4.77 (d, *J* = 5.9 Hz, 1H, 2‐O*H*), 6.85–6.91 (m, 4H, 3″‐H_4‐methoxyphenyl_, 5″‐H_4‐methoxyphenyl_), 7.19–7.25 (m, 4H, 2″‐H_4‐methoxyphenyl_, 6″‐H_4‐methoxyphenyl_); ^13^C NMR (126 MHz, DMSO‐*d*_*6*_): *δ* [ppm] = 25.2 (1C, C(*C*H_3_)_2_), 26.5 (1C, C(*C*H_3_)_2_), 55.0 (2C, O*C*H_3_), 62.9 (1C, CHCHCH*C*H_2_OH), 66.2 (1C, C‐5′_1,3‐dioxolan‐4‐yl_), 70.4 (1C, CHCH*C*HCH_2_OH), 73.0 (1C, 3‐O*C*H_2_Ar), 73.5 (1C, 4‐O*C*H_2_Ar), 75.5 (1C, C‐4′_1,3‐dioxolan‐4‐yl_), 78.6 (1C, *C*HCHCHCH_2_OH), 79.0 (1C, CH*C*HCHCH_2_OH), 107.6 (1C, C‐2′_1,3‐dioxolan‐4‐yl_), 113.5 (2C, C‐3″_4__‐methoxyphenyl_, C‐5″_4__‐methoxyphenyl_), 113.6 (2C, C‐3″_4__‐methoxyphenyl_, C‐5″_4__‐methoxyphenyl_), 129.4 (4C, C‐2″_4‐methoxyphenyl_, C‐6″_4‐methoxyphenyl_), 130.7 (1C, C‐1″_4‐methoxyphenyl_), 130.9 (1C, C‐1″_4‐methoxyphenyl_), 158.7 (2C, C‐4″_4‐methoxyphenyl_); IR (neat): $\tilde{\nu }$ [cm^–1^] = 3447, 2985, 2934, 2837, 1612, 1586, 1513, 1463, 1422, 1371, 1345, 1302, 1245, 1174, 1030, 819, 755, 706, 637, 572, 513; HRMS (*m*/*z*): [M+Na]^+^ calcd for C_25_H_34_NaO_8_: 485.2146, found: 485.2161; HPLC (method 1): *t*_R_ = 21.2 min, purity 79.8%.

#### 4.1.3 Synthesis of (2*S*,3*R*)−3‐[(*R*)‐2,2‐dimethyl‐1,3‐dioxolan‐4‐yl]‐2,3‐bis[(4‐methoxybenzyl)oxy]propanal (**20**)

Sodium metaperiodate (6.3 g, 29 mmol) was added to a solution of **19** (6.8 g, 15 mmol) in a mixture of THF and water (2/1, 68 mL) at 0°C. After stirring the reaction mixture at ambient temperature for 24 h, dichloromethane and water were added, and the aqueous phase was extracted with dichloromethane (3×). The combined organic layers were dried (Na_2_SO_4_), filtered, and the solvent was removed *in vacuo*. The residue was purified by flash column chromatography (petroleum ether/ethyl acetate = 2/1) to give **20** as a colorless oil (5.7 g, 13 mmol, 91%). TLC: *R*_*f*_ = 0.29 (petroleum ether/ethyl acetate = 4/1); ${\left[\alpha \right]}_{D}^{20}$ = −14.6 (10, chloroform); ^1^H NMR (400 MHz, CDCl_3_): *δ* [ppm] = 1.32 (s, 3H, C(C*H*_3_)_2_), 1.39 (s, 3H, C(C*H*_3_)_2_), 3.79 (s, 3H, OC*H*_3_), 3.80 (s, 3H, OC*H*_3_), 3.87 (dd, *J* = 8.5/6.3 Hz, 1H, 5′‐H_1,3‐dioxolan‐4‐yl_), 3.91 (dd, *J* = 6.4/3.0 Hz, 1H, C*H*CHCHO), 3.96 (dd, *J* = 3.0/1.4 Hz, 1H, CHC*H*CHO), 4.02 (dd, *J* = 8.5/6.3 Hz, 1H, 5′‐H_1,3‐dioxolan‐4‐yl_), 4.19 (q, *J* = 6.3 Hz, 1H, 4′‐H_1,3‐dioxolan‐4‐yl_), 4.47 (s, 2H, 3‐OC*H*_2_Ar), 4.57 (d, *J* = 11.6 Hz, 1H, 2‐OC*H*_2_Ar), 4.66 (d, *J* = 11.6 Hz, 1H, 2‐OC*H*_2_Ar), 6.82–6.90 (m, 4H, 3''‐H_4‐methoxyphenyl_, 5''‐H_4‐methoxyphenyl_), 7.14–7.20 (m, 2H, 2''‐H_4‐methoxyphenyl_, 6''‐H_4‐methoxyphenyl_), 7.23–7.30 (m, 2H, 2''‐H_4‐methoxyphenyl_, 6''‐H_4‐methoxyphenyl_), 9.63 (d, *J* = 1.4 Hz, 1H, CHCHC*H*O); ^13^C NMR (101 MHz, CDCl_3_): *δ* [ppm] = 25.4 (1C, C(*C*H_3_)_2_), 26.8 (1C, C(*C*H_3_)_2_), 55.39 (1C, O*C*H_3_), 55.40 (1 C, O*C*H_3_), 66.8 (1C, C‐5′_1,3‐dioxolan‐4‐yl_), 73.5 (1C, 2‐O*C*H_2_Ar), 74.2 (1C, 3‐O*C*H_2_Ar), 75.5 (1C, C‐4′_1,3‐dioxolan‐4‐yl_), 79.4 (1C, *C*HCHCHO), 83.8 (1C, CH*C*HCHO), 109.1 (1C, C‐2′_1,3‐dioxolan‐4‐yl_), 113.9 (2C, C‐3''_4‐methoxyphenyl_, C‐5''_4‐methoxyphenyl_), 114.1 (2C, C‐3''_4‐methoxyphenyl_, C‐5''_4‐methoxyphenyl_), 129.2 (1C, C‐1''_4‐methoxyphenyl_), 129.7 (1C, C‐1''_4‐methoxyphenyl_), 129.9 (2C, C‐2''_4‐methoxyphenyl_, C‐6''_4‐methoxyphenyl_), 130.3 (2C, C‐2''_4‐methoxyphenyl_, C6''_4‐methoxyphenyl_), 159.6 (1C, C‐4''_4‐methoxyphenyl_), 159.8 (1C, C‐4''_4‐methoxyphenyl_), 203.6 (1C, CHCH*C*HO); IR (neat): $\tilde{\nu }$ [cm^–1^] = 2986, 2936, 2905, 2837, 1731, 1612, 1586, 1513, 1463, 1371, 1342, 1302, 1244, 1173, 1068, 1030, 936, 819, 759, 575, 514; HRMS (*m*/*z*): [M+Na]^+^ calcd for C_24_H_30_NaO_7_: 453.1884, found: 453.1873.

#### 4.1.4 Synthesis of {(2*R*,3*R*,4*R*,5*S*)‐5‐(4‐iodophenyl)‐3,4‐bis[(4‐methoxybenzyl)oxy]tetrahydrofuran‐2‐yl}methanol (**21**) and {(2*R*,3*R*,4*R*,5*R*)‐5‐(4‐iodophenyl)‐3,4‐bis[(4‐methoxybenzyl)oxy]tetrahydrofuran‐2‐yl}methanol (**22**)

Under N_2_ atmosphere, a solution of 1,4‐diiodobenzene (4.8 g, 15 mmol) in dry THF (74 mL) was cooled to −78°C, and a 1.6 M solution of *n*‐butyllithium in hexanes (5.5 mL, 8.8 mmol) was added dropwise. After stirring the mixture at –78°C for 10 min, a solution of **20** (3.2 g, 7.3 mmol) in dry THF (37 mL) was added dropwise, and the mixture was allowed to slowly warm to ambient temperature over the course of 22.5 h in the thawing cooling bath. Then, a saturated aqueous solution of NH_4_Cl was added, and the aqueous phase was extracted with dichloromethane (3×). The combined organic layers were dried (Na_2_SO_4_), filtered, and the solvent was removed *in vacuo*. The residue was purified by flash column chromatography (petroleum ether/ethyl acetate = 3/1 → 2/1).

The inseparable mixture of diastereomeric alcohols (3.2 g, 5.1 mmol, petroleum ether/ethyl acetate = 3/1, *R*_*f*_ = 0.20) obtained was dissolved in dichloromethane (51 mL). After cooling the solution to 0°C, triethylamine (1.4 mL, 1.0 g, 10 mmol) and methanesulfonyl chloride (0.59 mL, 0.88 g, 7.6 mmol) were added, and the reaction mixture was stirred for 1 h at 0°C. Then, water was added, and the aqueous phase was extracted with dichloromethane (3×). The combined organic layers were washed with brine, dried (Na_2_SO_4_), filtered, and the solvent was removed *in vacuo*.

The obtained crude product was dissolved in a mixture of dichloromethane and methanol (2/1, 51 mL). After cooling the solution to 0°C, camphorsulfonic acid (240 mg, 1.0 mmol) was added, and the mixture was stirred for 18 h in the thawing ice bath. Then, a saturated aqueous solution of NaHCO_3_ was added, and the aqueous phase was extracted with dichloromethane (3×). The combined organic layers were dried (Na_2_SO_4_), filtered, and the solvent was removed *in vacuo*. The residue was purified by flash column chromatography (petroleum ether/ethyl acetate = 2/1 → 1/1) to give **21** (petroleum ether/ethyl acetate = 2/1, *R*_*f*_ = 0.22) as a colorless oil (600 mg, 1.0 mmol, 14%) and **22** (petroleum ether/ethyl acetate = 2/1, *R*_*f*_ = 0.19) as a colorless oil (690 mg, 1.2 mmol, 16%).

Analytical data of **21**: ${\left[\alpha \right]}_{D}^{20}$ = +28.2 (9.5, chloroform); ^1^H NMR (500 MHz, DMSO‐*d*_*6*_): *δ* [ppm] = 3.50 (dt, *J* = 11.1/6.4 Hz, 1H, C*H*_2_OH), 3.54–3.60 (m, 1H, C*H*_2_OH), 3.71 (s, 3H, OC*H*_3_), 3.75 (s, 3H, OC*H*_3_), 3.88 (td, *J* = 6.3/2.7 Hz, 1H, 2‐H), 3.94–3.99 (m, 2H, 3‐H, 4‐OC*H*_2_Ar (1H)), 4.02–4.04 (m, 1H, 4‐H), 4.19 (d, *J* = 11.6 Hz, 1H, 4‐OC*H*_2_Ar), 4.46 (d, *J* = 11.5 Hz, 1H, 3‐OC*H*_2_Ar), 4.49 (d, *J* = 11.5 Hz, 1H, 3‐OC*H*_2_Ar), 4.85 (t, *J* = 5.7 Hz, 1H, CH_2_O*H*), 4.97 (d, *J* = 3.7 Hz, 1H, 5‐H), 6.76–6.82 (m, 2H, 3'''‐H_4‐[(4‐methoxybenzyl)oxy]_, 5'''‐H_4‐[(4‐methoxybenzyl)oxy]_), 6.82–6.88 (m, 2H, 2'''‐H_4‐[(4‐methoxybenzyl)oxy]_, 6'''‐H_4‐[(4‐methoxybenzyl)oxy]_), 6.89–6.95 (m, 2H, 3''‐H_3‐[(4‐methoxybenzyl)oxy]_, 5''‐H_3‐[(4‐methoxybenzyl)oxy]_), 7.16–7.22 (m, 2H, 2′‐H_4‐iodophenyl_, 6′‐H_4‐iodophenyl_), 7.24–7.30 (m, 2H, 2''‐H_3‐[(4‐methoxybenzyl)oxy]_, 6''‐H_3‐[(4‐methoxybenzyl)oxy]_), 7.65–7.71 (m, 2H, 3′‐H_4‐iodophenyl_, 5′‐H_4‐iodophenyl_); ^13^C NMR (126 MHz, DMSO‐*d*_*6*_): *δ* [ppm] = 55.0 (1C, O*C*H_3_), 55.1 (1C, O*C*H_3_), 61.8 (1C, *C*H_2_OH), 70.3 (1C, 3‐O*C*H_2_Ar), 70.4 (1C, 4‐O*C*H_2_Ar), 81.7 (1C, C‐5), 83.1 (1C, C‐4), 83.8 (1C, C‐3), 84.3 (1C, C‐2), 93.0 (1C, C‐4′_4‐iodophenyl_), 113.4 (2C, C‐3'''4_‐[(4‐methoxybenzyl)oxy]_, C‐5'''_4__‐[(4‐methoxybenzyl)oxy]_), 113.6 (2C, C‐3''_3__‐[(4‐methoxybenzyl)oxy]_, C‐5''_3__‐[(4‐methoxybenzyl)oxy]_), 128.8 (2C, C‐2'''_4__‐[(4‐methoxybenzyl)oxy]_, C‐6'''_4__‐[(4‐methoxybenzyl)oxy]_), 129.3 (2C, C‐2''_3__‐[(4‐methoxybenzyl)oxy]_, C‐6''_3__‐[(4‐methoxybenzyl)oxy]_), 129.69 (2C, C‐2′_4‐iodophenyl_, C‐6′_4‐iodophenyl_), 129.73 (1C, C‐1'''_4__‐[(4‐methoxybenzyl)oxy]_), 130.0 (1C, C‐1''_3__‐[(4‐methoxybenzyl)oxy]_), 136.2 (2C, C‐3′_4‐iodophenyl_, C‐5′_4‐iodophenyl_), 137.3 (1C, C‐1′_4‐iodophenyl_), 158.6 (1C, C‐4'''_4__‐[(4‐methoxybenzyl)oxy]_), 158.8 (1C, C‐4''_3__‐[(4‐methoxybenzyl)oxy]_); IR (neat): $\tilde{\nu }$ [cm^–1^] = 3450, 2999, 2908, 2865, 2835, 1611, 1586, 1512, 1485, 1463, 1441, 1421, 1397, 1360, 1302, 1246, 1174, 1059, 1033, 1006, 817, 755, 735, 704, 576, 514; HRMS (*m*/*z*): [M+Na]^+^ calcd for C_27_H_29_INaO_6_: 599.0901, found: 599.0905; HPLC (method 1): *t*_R_ = 25.7 min, purity 96.4%.

Analytical data of **22**: ${\left[\alpha \right]}_{D}^{20}$ = +49.7 (10, chloroform); ^1^H NMR (500 MHz, DMSO‐*d*_*6*_): *δ* [ppm] = 3.49–3.60 (m, 2H, C*H*_2_OH), 3.74 (s, 6H, OC*H*_3_), 3.96 (dd, *J* = 4.8/2.9 Hz, 1H, 4‐H), 4.06 (t, *J* = 3.0 Hz, 1H, 3‐H), 4.09–4.14 (m, 1H, 2‐H), 4.37 (d, *J* = 11.4 Hz, 1H, 3‐OC*H*_2_Ar), 4.40–4.46 (m, 3H, 3‐OC*H*_2_Ar (1H), 4‐OC*H*_2_Ar), 4.89 (d, *J* = 4.9 Hz, 1H, 5‐H), 4.93 (t, *J* = 5.7 Hz, 1H, CH_2_O*H*), 6.84–6.90 (m, 4H, 3''‐H_4‐methoxyphenyl_, 5''‐H_4‐methoxyphenyl_), 7.08 –7.13 (m, 2H, 2''‐H_4‐methoxyphenyl_, 6''‐H_4‐methoxyphenyl_), 7.14– 7.19 (m, 4H, 2′‐H_4‐iodophenyl_, 6′‐H_4‐iodophenyl_, 2''‐H_4‐methoxyphenyl_, 6''‐H_4‐methoxyphenyl_), 7.67–7.71 (m, 2H, 3′‐H_4‐iodophenyl_, 5′‐H_4‐iodophenyl_); ^13^C NMR (126 MHz, DMSO‐*d*_*6*_): *δ* [ppm] = 55.02 (1C, O*C*H_3_), 55.04 (1C, O*C*H_3_), 61.5 (1C, *C*H_2_OH), 70.3 (1C, 3‐O*C*H_2_Ar), 70.7 (1C, 4‐O*C*H_2_Ar), 82.6 (1C, C‐5), 84.0 (1C, C‐2), 84.2 (1C, C‐3), 89.1 (1C, C‐4), 93.2 (1C, C‐4′_4‐iodophenyl_), 113.5 (2C, C‐3''_4__‐methoxyphenyl_, C‐5''_4__‐methoxyphenyl_), 113.6 (2C, C‐3''_4__‐methoxyphenyl_, C‐5''_4__‐methoxyphenyl_), 128.4 (2C, C‐2′_4‐iodophenyl_, C‐6′_4‐iodophenyl_), 129.2 (2C, C‐2''_4__‐methoxyphenyl_, C‐6''_4__‐methoxyphenyl_), 129.3 (2C, C‐2''_4__‐methoxyphenyl_, C‐6″_4‐methoxyphenyl_), 129.7 (1C, C‐1''_4__‐methoxyphenyl_), 129.9 (1C, C‐1''_4__‐methoxyphenyl_), 136.9 (2C, C‐3′_4‐iodophenyl_, C‐5′_4‐iodophenyl_), 140.8 (1C, C‐1′_4‐iodophenyl_), 158.7 (1C, C‐4''_4__‐methoxyphenyl_), 158.8 (1C, C‐4''_4__‐methoxyphenyl_); IR (neat): $\tilde{\nu }$ [cm^–1^] = 3435, 2998, 2923, 2853, 1728, 1612, 1586, 1513, 1484, 1463, 1394, 1361, 1302, 1248, 1174, 1069, 1034, 1006, 847, 818, 761, 583, 517; HRMS (*m*/*z*): [M+Na]^+^ calcd for C_27_H_29_INaO_6_: 599.0901, found: 599.0912; HPLC (method 1): *t*_R_ = 25.6 min, purity 96.9%.

#### 4.1.5 Synthesis of methyl (2*S*,3*S*,4*R*,5*S*)‐5‐(4‐iodophenyl)‐3,4‐bis[(4‐methoxybenzyl)oxy]tetrahydrofuran‐2‐carboxylate (**23**)

**21** (180 mg, 0.31 mmol) was dissolved in dichloromethane (3.1 mL), which had been saturated with water. *N*‐Methylmorpholine *N*‐oxide monohydrate (420 mg, 3.1 mmol) and tetrapropylammonium perruthenate (11 mg, 0.031 mmol) were successively added, and the reaction mixture was stirred at ambient temperature for 3 d. Then, the mixture was filtered through Celite, and the solvent was removed *in vacuo*. The residue was dissolved in acetone (3.1 mL), and K_2_CO_3_ (210 mg, 1.5 mmol) and iodomethane (0.096 mL, 220 mg, 1.5 mmol) were successively added. After stirring the reaction mixture at ambient temperature for 18 h, it was filtered through Celite, and the solvent was removed *in vacuo*. The residue was dissolved in ethyl acetate, and the organic solvent was washed with water, dried (Na_2_SO_4_), filtered, and removed *in vacuo*. The residue was purified by flash column chromatography (petroleum ether/ethyl acetate = 4/1, *R*_*f*_ = 0.44) to give **23** as a colorless oil (130 mg, 0.22 mmol, 71%). ${\left[\alpha \right]}_{D}^{20}$ = +2.1 (4.2, chloroform); ^1^H NMR (400 MHz, CDCl_3_): *δ* [ppm] = 3.73 (s, 3H, CO_2_C*H*_3_), 3.79 (s, 3H, ArOC*H*_3_), 3.80–3.84 (m, 4H, 4‐H, ArOC*H*_3_ (3H)), 3.96 (d, *J* = 11.8 Hz, 1H, OC*H*_2_Ar), 4.11 (d, *J* = 11.8 Hz, 1H, OC*H*_2_Ar), 4.41 (m, 1H, 3‐H), 4.51 (d, *J* = 11.5 Hz, 1H, OC*H*_2_Ar), 4.61–4.66 (m, 2H, 2‐H, OC*H*_2_Ar (1H)), 5.17 (d, *J* = 3.2 Hz, 1H, 5‐H), 6.72–6.81 (m, 4H, 2''‐H_4‐methoxyphenyl_, 3''‐H_4‐methoxyphenyl_, 5''‐H_4‐methoxyphenyl_, 6''‐H_4‐methoxyphenyl_), 6.88–6.94 (m, 2H, 3''‐H_4‐methoxyphenyl_, 5''‐H_4‐methoxyphenyl_), 7.21–7.31 (m, 4H, 2′‐H_4‐iodophenyl_, 6′‐H_4‐iodophenyl_, 2″‐H_4‐methoxyphenyl_, 6″‐H_4‐methoxyphenyl_), 7.62–7.68 (m, 2H, 3′‐H_4‐iodophenyl_, 5′‐H_4‐iodophenyl_); ^13^C NMR (101 MHz, CDCl_3_): *δ* [ppm] = 52.4 (1C, CO_2_ *C*H_3_), 55.4 (1C, ArO*C*H_3_), 55.5 (1C, ArO*C*H_3_), 71.5 (1C, O*C*H_2_Ar), 71.7 (1C, O*C*H_2_Ar), 81.7 (2C, C‐2, C‐4), 84.3 (1C, C‐5), 86.0 (1C, C‐3), 93.3 (1C, C‐4′_4‐iodophenyl_), 113.7 (2C, C‐3''_4__‐methoxyphenyl_, C‐5''_4__‐methoxyphenyl_), 114.1 (2C, C‐3''_4__‐methoxyphenyl_, C‐5''_4__‐methoxyphenyl_), 129.1 (2C, C‐2''_4__‐methoxyphenyl_, C‐6''_4__‐methoxyphenyl_), 129.36 (1C, C‐1''_4__‐methoxyphenyl_), 129.45 (1C, C‐1''_4__‐methoxyphenyl_), 129.7 (2C, C‐2''_4__‐methoxyphenyl_, C‐6''_4__‐methoxyphenyl_), 129.9 (2C, C‐2′_4‐iodophenyl_, C‐6′_4‐iodophenyl_), 136.4 (1C, C‐1′_4‐iodophenyl_), 137.0 (2C, C‐3′_4‐iodophenyl_, C‐5′_4‐iodophenyl_), 159.3 (1C, C‐4''_4__‐methoxyphenyl_), 159.7 (1C, C‐4''_4__‐methoxyphenyl_), 171.1 (1C, *C*O_2_CH_3_); IR (neat): $\tilde{\nu }$ [cm^–1^] = 2954, 2923, 2854, 1758, 1729, 1652, 1612, 1586, 1513, 1485, 1463, 1440, 1395, 1379, 1359, 1301, 1247, 1212, 1175, 1095, 1035, 1007, 949, 820, 767, 593, 516, 438; HRMS (*m*/*z*): [M+Na]^+^ calcd for C_28_H_29_INaO_7_: 627.0850, found: 627.0823; HPLC (method 1): *t*_R_ = 27.3 min, purity 95.2%.

#### 4.1.6 Synthesis of methyl (2*S*,3*S*,4*R*,5*R*)‐5‐(4‐iodophenyl)‐3,4‐bis[(4‐methoxybenzyl)oxy]tetrahydrofuran‐2‐carboxylate (**24**)

**22** (660 mg, 1.2 mmol) was dissolved in dichloromethane (12 mL), which had been saturated with water. *N*‐Methylmorpholine *N*‐oxide monohydrate (1.6 g, 12 mmol) and tetrapropylammonium perruthenate (40 mg, 0.12 mmol) were successively added, and the reaction mixture was stirred at ambient temperature for 15.5 h. Then, the mixture was filtered through Celite, and the solvent was removed *in vacuo*. The residue was dissolved in acetone (12 mL), and K_2_CO_3_ (800 mg, 5.8 mmol) and iodomethane (0.36 mL, 820 mg, 5.8 mmol) were successively added. After stirring the reaction mixture at ambient temperature for 12.5 h, it was filtered through Celite, and the solvent was removed *in vacuo*. The residue was dissolved in ethyl acetate, and the organic solvent was washed with water, dried (Na_2_SO_4_), filtered, and removed *in vacuo*. The residue was purified by flash column chromatography (petroleum ether/ethyl acetate = 4/1, *R*_*f*_ = 0.43) to give **24** as a colorless oil (450 mg, 0.75 mmol, 65%). ${\left[\alpha \right]}_{D}^{20}$ = +42.2 (5.0, chloroform); ^1^H NMR (400 MHz, CDCl_3_): *δ* [ppm] = 3.78 (s, 3H, CO_2_C*H*_3_), 3.80 (s, 3H, ArOC*H*_3_), 3.82 (s, 3H, ArOC*H*_3_), 3.92 (dd, *J* = 5.3/2.6 Hz, 1H, 4‐H), 4.33–4.41 (m, 3H, 3‐H, OC*H*_2_Ar (2H)), 4.44 (d, *J* = 11.4 Hz, 1H, OC*H*_2_Ar), 4.59 (d, *J* = 11.4 Hz, 1H, OC*H*_2_Ar), 4.80 (d, *J* = 2.1 Hz, 1H, 2‐H), 5.05 (d, *J* = 5.3 Hz, 1H, 5‐H), 6.80–6.85 (m, 2H, 3''‐H_4‐methoxyphenyl_, 5''‐H_4‐methoxyphenyl_), 6.85–6.91 (m, 2H, 3''‐H_4‐methoxyphenyl_, 5''‐H_4‐methoxyphenyl_), 7.06–7.21 (m, 6H, 2′‐H_4‐iodophenyl_, 6′‐H_4‐iodophenyl_, 2''‐H_4‐methoxyphenyl_ (2H), 6''‐H_4‐methoxyphenyl_ (2H)), 7.61–7.68 (m, 2H, 3′‐H_4‐iodophenyl_, 5′‐H_4‐iodophenyl_); ^13^C NMR (101 MHz, CDCl_3_): *δ* [ppm] = 52.5 (1C, CO_2_ *C*H_3_), 55.4 (2C, ArO*C*H_3_), 71.7 (1C, O*C*H_2_Ar), 72.0 (1C, O*C*H_2_Ar), 81.8 (1C, C‐2), 84.6 (1C, C‐5), 86.3 (1C, C‐3), 88.7 (1C, C‐4), 93.4 (1C, C‐4′_4‐iodophenyl_), 113.9 (2C, C‐3''_4__‐methoxyphenyl_, C‐5''_4__‐methoxyphenyl_), 114.0 (2C, C‐3''_4__‐methoxyphenyl_, C‐5''_4__‐methoxyphenyl_), 128.4 (2C, C‐2′_4‐iodophenyl_, C‐6′_4‐iodophenyl_), 129.3 (1C, C‐1''_4__‐methoxyphenyl_), 129.4 (1C, C‐1''_4__‐methoxyphenyl_), 129.5 (2C, C‐2''_4__‐methoxyphenyl_, C‐6''_4__‐methoxyphenyl_), 129.7 (2C, C‐2''_4__‐methoxyphenyl_, C‐6''_4__‐methoxyphenyl_), 137.5 (2C, C‐3′_4‐iodophenyl_, C‐5′_4‐iodophenyl_), 139.3 (1C, C‐1′_4‐iodophenyl_), 159.5 (1C, C‐4''_4__‐methoxyphenyl_), 159.6 (1C, C‐4''_4__‐methoxyphenyl_), 171.5 (1C, *C*O_2_CH_3_); IR (neat): $\tilde{\nu }$ [cm^–1^] = 2999, 2951, 2932, 2836, 1753, 1731, 1611, 1586, 1512, 1484, 1462, 1439, 1394, 1359, 1301, 1245, 1211, 1173, 1081, 1030, 1005, 847, 814, 755, 713, 636, 585, 516; HRMS (*m*/*z*): [M+Na]^+^ calcd for C_28_H_29_INaO_7_: 627.0850, found: 627.0816; HPLC (method 1): *t*_R_ = 27.4 min, purity 97.8%.

#### 4.1.7 Synthesis of methyl (2*S*,3*S*,4*S*,5*S*)‐3,4‐dihydroxy‐5‐(4‐iodophenyl)tetrahydrofuran‐2‐carboxylate (**25**)

At 0°C, a solution of **23** (390 mg, 0.65 mmol) in dichloromethane (12 mL) was added to a solution of DDQ (440 mg, 1.9 mmol) in water (1.3 mL). After stirring the reaction mixture at ambient temperature for 19.5 h, a saturated aqueous solution of NaHCO_3_ was added, and the aqueous phase was extracted with dichloromethane (3×). The combined organic layers were dried (Na_2_SO_4_), filtered, and the solvent was removed *in vacuo*. The residue was purified by flash column chromatography (dichloromethane/methanol = 9/1) to give **25** as a colorless solid (210 mg, 0.59 mmol, 90%). m.p. = 154°C; TLC: *R*_*f*_ = 0.45 (dichloromethane/methanol = 19/1); ${\left[\alpha \right]}_{D}^{20}$ = +40.0 (5.0, dichloromethane); HPLC (method 1): *t*_R_ = 17.4 min, purity 97.4%.

#### 4.1.8 Synthesis of methyl (2*R*,3*R*,4*R*,5*R*)‐3,4‐dihydroxy‐5‐(4‐iodophenyl)tetrahydrofuran‐2‐carboxylate (*ent*‐**25**)

A 1.0 M solution of tetrabutylammonium fluoride in THF (9.6 mL, 9.6 mmol) was added to a solution of **40** (2.2 g, 2.7 mmol) in dry THF (27 mL). After stirring the reaction mixture at 60°C for 16.5 h, a saturated aqueous solution of NH_4_Cl was added, and the mixture was extracted with dichloromethane (3×). The combined organic layers were dried (Na_2_SO_4_), filtered, and the solvent was removed *in vacuo*. The residue was dissolved in methanol (27 mL), and *p*‐toluenesulfonic acid monohydrate (100 mg, 0.53 mmol) was added. After stirring the mixture at 60°C for 17.5 h, it was cooled to ambient temperature. A saturated aqueous solution of NaHCO_3_ was added, and the mixture was extracted with dichloromethane (3×). The combined organic layers were dried (Na_2_SO_4_), filtered, and the solvent was removed *in vacuo*. The residue was purified by flash column chromatography (dichloromethane/methanol = 19/1, *R*_*f*_ = 0.45) to give *ent*‐**25** as a colorless solid (800 mg, 2.2 mmol, 83%). m.p. = 158°C; ${\left[\alpha \right]}_{D}^{20}$ = ‐85.6 (5.0, dichloromethane); HPLC (method 1): *t*_R_ = 17.4 min, purity 90.2%.

Spectroscopic data of **25** and *ent*‐**25**: ^1^H NMR (400 MHz, DMSO‐*d*_*6*_): *δ* [ppm] = 3.67 (s, 3H, CO_2_C*H*_3_), 3.84–3.88 (m, 1H, 4‐H), 4.34–4.38 (m, 2H, 2‐H, 3‐H), 4.87 (d, *J* = 3.9 Hz, 1H, 4‐O*H*), 5.09 (d, *J* = 3.0 Hz, 1H, 5‐H), 5.78 (d, *J* = 3.8 Hz, 1H, 3‐O*H*), 7.31–7.35 (m, 2H, 2′‐H_4‐iodophenyl_, 6′‐H_4‐iodophenyl_), 7.64–7.69 (m, 2H, 3′‐H_4‐iodophenyl_, 5′‐H_4‐iodophenyl_); ^13^C NMR (101 MHz, DMSO‐*d*_*6*_): *δ* [ppm] = 51.6 (1C, CO_2_ *C*H_3_), 76.5 (1C, C‐4), 80.9 (1C, C‐3), 83.2 (1C, C‐2), 83.8 (1C, C‐5), 93.0 (1C, C‐4′_4‐iodophenyl_), 130.3 (2C, C‐2′_4‐iodophenyl_, C‐6′_4‐iodophenyl_), 136.1 (2C, C‐3′_4‐iodophenyl_, C‐5′_4‐iodophenyl_), 137.8 (1C, C‐1′_4‐iodophenyl_), 171.0 (1C, *C*O_2_CH_3_); IR (neat): $\tilde{\nu }$ [cm^–1^] = 3417, 3352, 2954, 2922, 2851, 1748, 1485, 1438, 1412, 1312, 1229, 1085, 1060, 1040, 1007, 944, 838, 822, 790, 772, 762; HRMS (*m*/*z*): [M+Na]^+^ calcd for C_12_H_13_INaO_5_: 386.9700, found: 386.9667.

#### 4.1.9 Synthesis of methyl (2*S*,3*S*,4*S*,5*R*)‐3,4‐dihydroxy‐5‐(4‐iodophenyl)tetrahydrofuran‐2‐carboxylate (**26**)

At 0°C, a solution of **24** (240 mg, 0.40 mmol) in dichloromethane (9 mL) was added to a solution of DDQ (270 mg, 1.2 mmol) in water (1 mL). After stirring the reaction mixture at ambient temperature for 19 h, a saturated aqueous solution of NaHCO_3_ was added, and the aqueous phase was extracted with dichloromethane (3×). The combined organic layers were dried (Na_2_SO_4_), filtered, and the solvent was removed *in vacuo*. The residue was purified by flash column chromatography (dichloromethane/methanol = 19/1 → 4/1) to give **26** as a colorless solid (120 mg, 0.34 mmol, 84%). m.p. = 173°C; TLC: *R*_*f*_ = 0.39 (dichloromethane/methanol = 19/1); ${\left[\alpha \right]}_{D}^{20}$ = +26.4 (5.0, methanol); ^1^H NMR (400 MHz, DMSO‐*d*_*6*_): *δ* [ppm] = 3.68 (s, 3H, CO_2_C*H*_3_), 3.71–3.75 (m, 1H, 4‐H), 4.15–4.20 (m, 1H, 3‐H), 4.48 (d, *J* = 3.8 Hz, 1H, 2‐H), 4.71 (d, *J* = 5.8 Hz, 1H, 5‐H), 5.54 (d, *J* = 4.9 Hz, 1H, 4‐O*H*), 5.66 (d, *J* = 5.0 Hz, 1H, 3‐O*H*), 7.17–7.22 (m, 2H, 2′‐H_4‐iodophenyl_, 6′‐H_4‐iodophenyl_), 7.68–7.73 (m, 2H, 3′‐H_4‐iodophenyl_, 5′‐H_4‐iodophenyl_); ^13^C NMR (101 MHz, DMSO‐*d*_*6*_): *δ* [ppm] = 51.7 (1C, CO_2_ *C*H_3_), 80.6 (1C, C‐3), 82.4 (1C, C‐2), 83.1 (1C, C‐4), 85.3 (1C, C‐5), 93.3 (1C, C‐4′_4‐iodophenyl_), 128.5 (2C, C‐2′_4‐iodophenyl_, C‐6′_4‐iodophenyl_), 136.9 (2C, C‐3′_4‐iodophenyl_, C‐5′_4‐iodophenyl_), 140.3 (1C, C‐1′_4‐iodophenyl_), 171.5 (1C, *C*O_2_CH_3_); IR (neat): $\tilde{\nu }$ [cm^–1^] = 3284, 1737, 1273, 1101, 1088, 1047, 1004, 934, 859, 795, 751, 496; HRMS (*m*/*z*): [M+Na]^+^ calcd for C_12_H_13_INaO_5_: 386.9700, found: 386.9699; HPLC (method 1): *t*_R_ = 17.2 min, purity 98.1%.

#### 4.1.10 Synthesis of methyl (2*S*,3*S*,4*S*,5*S*)‐3,4‐dihydroxy‐5‐(4‐{[4‐(morpholinomethyl)phenyl]ethynyl}phenyl)tetrahydrofuran‐2‐carboxylate (**27**)

Under N_2_ atmosphere, **25** (180 mg, 0.50 mmol), tetrakis(triphenylphosphine)palladium(0) (58 mg, 0.050 mmol), and copper(I) iodide (19 mg, 0.10 mmol) were suspended in dry THF (5.0 mL), and a solution of 4‐(4‐ethynylbenzyl)morpholine (110 mg, 0.55 mmol) in dry THF (5.5 mL) was added dropwise. After the addition of triethylamine (0.07 mL, 51 mg, 0.50 mmol), the reaction mixture was stirred at ambient temperature for 20 h. Then, the mixture was filtered through Celite, and the volatiles were removed *in vacuo*. The residue was purified by flash column chromatography (dichloromethane/methanol = 19/1, *R*_*f*_ = 0.39) to give **27** as a colorless solid (92 mg, 0.21 mmol, 42%). m.p. = 184°C; ${\left[\alpha \right]}_{D}^{20}$ = +22.8 (5.0, methanol); HPLC (method 1): *t*_R_ = 14.7 min, purity 99.0%.

#### 4.1.11 Synthesis of methyl (2*R*,3*R*,4*R*,5*R*)‐3,4‐dihydroxy‐5‐(4‐{[4‐(morpholinomethyl)phenyl]ethynyl}phenyl)tetrahydrofuran‐2‐carboxylate (*ent*‐**27**)

Under N_2_ atmosphere, tetrakis(triphenylphosphine)palladium(0) (74 mg, 0.064 mmol), copper(I) iodide (24 mg, 0.13 mmol), and triethylamine (0.09 mL, 65 mg, 0.64 mmol) were added to a solution of *ent*‐**25** (230 mg, 0.64 mmol) in dry THF (6.4 mL). After the dropwise addition of a solution of 4‐(4‐ethynylbenzyl)morpholine (140 mg, 0.70 mmol) in dry THF (7.0 mL), the reaction mixture was stirred at ambient temperature for 20 h. Then, the volatiles were removed *in vacuo*, and the residue was purified by flash column chromatography (dichloromethane/methanol = 19/1, *R*_*f*_ = 0.39) to give *ent*‐**27** as a colorless solid (99 mg, 0.23 mmol, 35%). m.p. = 178°C; ${\left[\alpha \right]}_{D}^{20}$ = ‐29.3 (7.5, dichloromethane); HPLC (method 1): *t*_R_ = 14.8 min, purity 92.2%.

Spectroscopic data of **27** and *ent*‐**27**: ^1^H NMR (500 MHz, DMSO‐*d*_*6*_): *δ* [ppm] = 2.32–2.38 (m, 4H, N(C*H*_2_CH_2_)_2_O), 3.49 (s, 2H, NC*H*_2_Ar), 3.56–3.60 (m, 4H, N(CH_2_C*H*_2_)_2_O), 3.68 (s, 3H, CO_2_C*H*_3_), 3.89–3.92 (m, 1H, 4‐H), 4.36–4.39 (m, 2H, 2‐H, 3‐H), 4.88 (d, *J* = 3.9 Hz, 1H, 4‐O*H*), 5.16 (d, *J* = 3.1 Hz, 1H, 5‐H), 5.79 (d, *J* = 3.8 Hz, 1H, 3‐O*H*), 7.34–7.38 (m, 2H, 3''‐H_4‐(morpholinomethyl)phenyl_, 5''‐H_4‐(morpholinomethyl)phenyl_), 7.46–7.53 (m, 4H, 3′‐H_4‐{[4‐(morpholinomethyl)phenyl]ethynyl}phenyl_, 5′‐H_4‐{[4‐(morpholinomethyl)phenyl]ethynyl}phenyl_, 2''‐H_4‐(morpholinomethyl)phenyl_, 6''‐H_4‐(morpholinomethyl)phenyl_), 7.54–7.58 (m, 2H, 2′‐H_4‐{[4‐(morpholinomethyl)phenyl]ethynyl}phenyl_, 6′‐H_4‐{[4‐(morpholinomethyl)phenyl]ethynyl}phenyl_); ^13^C NMR (126 MHz, DMSO‐*d*_*6*_): *δ* [ppm] = 51.5 (1C, CO_2_ *C*H_3_), 53.1 (2C, N(*C*H_2_CH_2_)_2_O), 62.0 (1C, Ar*C*H_2_N), 66.2 (2C, N(CH_2_ *C*H_2_)_2_O), 76.6 (1C, C‐4), 81.0 (1C, C‐3), 83.2 (1C, C‐2), 84.0 (1C, C‐5), 88.8 (1C, *C*≡C), 89.5 (1C, *C*≡C), 120.8 (1C, C‐4′_4‐{[4‐(morpholinomethyl)phenyl]ethynyl}phenyl_), 121.0 (1C, C‐1''_4__‐(morpholinomethyl)phenyl_), 128.2 (2C, C‐2′_4‐{[4‐(morpholinomethyl)phenyl]ethynyl}phenyl_, C‐6′_4‐{[4‐(morpholinomethyl)phenyl]ethynyl}phenyl_), 129.2 (2C, C‐3''_4__‐(morpholinomethyl)phenyl_, C‐5''_4__‐(morpholinomethyl)phenyl_), 130.4 (2C, C‐3′_4‐{[4‐(morpholinomethyl)phenyl]ethynyl}phenyl_, C‐5′_4‐{[4‐(morpholinomethyl)phenyl]ethynyl}phenyl_), 131.2 (2C, C‐2''_4__‐(morpholinomethyl)phenyl_, C‐6''_4__‐(morpholinomethyl)phenyl_), 138.71 (1C, C_arom._), 138.75 (1C, C_arom._), 171.0 (1C, *C*O_2_CH_3_); IR (neat): $\tilde{\nu }$ [cm^–1^] = 3374, 3035, 2921, 2893, 2851, 2697, 1755, 1519, 1457, 1435, 1411, 1378, 1351, 1335, 1320, 1296, 1263, 1208, 1112, 1099, 1073, 1031, 1000, 957, 920, 866, 845, 788, 761, 718, 582, 528, 451, 444; HRMS (*m*/*z*): [M + H]^+^ calcd for C_25_H_28_NO_6_: 438.1911, found: 438.1934.

#### 4.1.12 Synthesis of methyl (2*S*,3*S*,4*S*,5*R*)‐3,4‐dihydroxy‐5‐(4‐{[4‐(morpholinomethyl)phenyl]ethynyl}phenyl)tetrahydrofuran‐2‐carboxylate (**28**)

Under N_2_ atmosphere, **26** (190 mg, 0.52 mmol), tetrakis(triphenylphosphine)palladium(0) (60 mg, 0.052 mmol), and copper(I) iodide (20 mg, 0.10 mmol) were suspended in dry THF (5.2 mL). After the addition of triethylamine (0.07 mL, 53 mg, 0.52 mmol), a solution of 4‐(4‐ethynylbenzyl)morpholine (110 mg, 0.57 mmol) in dry THF (5.7 mL) was added dropwise. After stirring the reaction mixture at ambient temperature for 18.5 h, the mixture was filtered through Celite, and the volatiles were removed *in vacuo*. The residue was purified by flash column chromatography (dichloromethane/methanol = 19/1, *R*_*f*_ = 0.32) to give **28** as a colorless solid (120 mg, 0.27 mmol, 52%). m.p. = 153°C; ${\left[\alpha \right]}_{D}^{20}$ = +8.6 (5.0, methanol); HPLC (method 1): *t*_R_ = 14.7 min, purity 100%.

#### 4.1.13 Synthesis of methyl (2*R*,3*R*,4*R*,5*S*)‐3,4‐dihydroxy‐5‐(4‐{[4‐(morpholinomethyl)phenyl]ethynyl}phenyl)tetrahydrofuran‐2‐carboxylate (*ent*‐**28**)

Under N_2_ atmosphere, tetrakis(triphenylphosphine)palladium(0) (9.6 mg, 0.0083 mmol) and copper(I) iodide (3.0 mg, 0.016 mmol) were added to a solution of **45** (32 mg, 0.083 mmol) in acetonitrile (1.0 mL). After the addition of triethylamine (35 µL, 25 mg, 0.25 mmol), the mixture was heated to 60°C, and trimethylsilylacetylene (35 µL, 24 mg, 0.25 mmol) was added. After stirring the reaction mixture at 60°C overnight, the volatiles were removed *in vacuo*, and the residue was purified by flash column chromatography (dichloromethane/methanol = 19/1). Fractions containing the desired product were combined, and the solvent was removed *in vacuo*.

The residue (27 mg, 0.081 mmol) was dissolved in a mixture of chloroform (0.6 mL) and THF (0.5 mL). After the addition of tetrabutylammonium fluoride trihydrate (31 mg, 0.10 mmol), the mixture was stirred at ambient temperature for 1 h. Then, water (10 mL) was added, and the mixture was extracted with ethyl acetate (3× 15 mL). The combined organic layers were dried (Na_2_SO_4_), filtered, and the solvent was removed *in vacuo*. The residue was purified by flash column chromatography (dichloromethane/methanol = 19/1). Fractions containing the desired product were combined, and the solvent was removed *in vacuo*.

Under N_2_ atmosphere, tetrakis(triphenylphosphine)palladium(0) (5.3 mg, 0.0046 mmol), copper(I) iodide (1.8 mg, 0.0092 mmol), and triethylamine (19 µL, 14 mg, 0.14 mmol) were added to a solution of 4‐(4‐iodobenzyl)morpholine (35 mg, 0.12 mmol) in acetonitrile (0.3 mL). Then, a solution of the previously obtained residue (12 mg, 0.046 mmol) in acetonitrile (0.4 mL) was added dropwise. After stirring the reaction mixture at ambient temperature for 1 h, the volatiles were removed *in vacuo* and the residue was purified by flash column chromatography (dichloromethane/methanol = 19/1, *R*_*f*_ = 0.32) to give *ent*‐**28** as a colorless solid (5.0 mg, 0.011 mmol, 14%).

m.p. = 153°C; ${\left[\alpha \right]}_{D}^{20}$ = +6.1 (1.8, dichloromethane); HPLC (method 1): *t*_R_ = 14.7 min, purity 99.1%.

Spectroscopic data of **28** and *ent*‐**28**: ^1^H NMR (500 MHz, DMSO‐*d*_*6*_): *δ* [ppm] = 2.32–2.38 (m, 4H, N(C*H*_2_CH_2_)_2_O), 3.49 (s, 2H, NC*H*_2_Ar), 3.55–3.60 (m, 4H, N(CH_2_C*H*_2_)_2_O), 3.69 (s, 3H, CO_2_C*H*_3_), 3.75–3.80 (m, 1H, 4‐H), 4.18–4.22 (m, 1H, 3‐H), 4.51 (d, *J* = 3.8 Hz, 1H, 2‐H), 4.78 (d, *J* = 5.9 Hz, 1H, 5‐H), 5.59 (d, *J* = 4.0 Hz, 1H, 4‐O*H*), 5.70 (d, *J* = 3.5 Hz, 1H, 3‐O*H*), 7.34–7.38 (m, 2H, 3''‐H_4‐(morpholinomethyl)phenyl_, 5''‐H_4‐(morpholinomethyl)phenyl_), 7.41–7.45 (m, 2H, 2′‐H_4‐{[4‐(morpholinomethyl)phenyl]ethynyl}phenyl_, 6′‐H_4‐{[4‐(morpholinomethyl)phenyl]ethynyl}phenyl_), 7.49–7.54 (m, 4H, 3′‐H_4‐{[4‐(morpholinomethyl)phenyl]ethynyl}phenyl_, 5′‐H_4‐{[4‐(morpholinomethyl)phenyl]ethynyl}phenyl_, 2''‐H_4‐(morpholinomethyl)phenyl_, 6''‐H_4‐(morpholinomethyl)phenyl_); ^13^C NMR (126 MHz, DMSO‐*d*_*6*_): *δ* [ppm] = 51.7 (1C, CO_2_ *C*H_3_), 53.1 (2C, N(*C*H_2_CH_2_)_2_O), 62.0 (1C, Ar*C*H_2_N), 66.2 (2C, N(CH_2_ *C*H_2_)_2_O), 80.7 (1C, C‐3), 82.4 (1C, C‐2), 83.2 (1C, C‐4), 85.4 (1C, C‐5), 89.1 (1C, *C*≡C), 89.2 (1C, *C*≡C), 120.9 (1C, C‐1''_4__‐(morpholinomethyl)phenyl_), 121.3 (1C, C‐4′_4‐{[4‐(morpholinomethyl)phenyl]ethynyl}phenyl_), 126.5 (2C, C‐2′_4‐{[4‐(morpholinomethyl)phenyl]ethynyl}phenyl_, C‐6′_4‐{[4‐(morpholinomethyl)phenyl]ethynyl}phenyl_), 129.2 (2C, C‐3''_4__‐(morpholinomethyl)phenyl_, C‐5''_4__‐(morpholinomethyl)phenyl_), 131.1 (2C, C_arom._), 131.2 (2C, C_arom._), 138.8 (1C, C‐4''_4__‐(morpholinomethyl)phenyl_), 141.1 (1C, C‐1′_4‐{[4‐(morpholinomethyl)phenyl]ethynyl}phenyl_), 171.5 (1C, *C*O_2_CH_3_); IR (neat): $\tilde{\nu }$ [cm^–1^] = 3396, 2953, 2924, 2854, 1736, 1518, 1455, 1438, 1411, 1350, 1333, 1291, 1263, 1211, 1112, 1070, 1006, 967, 915, 865, 834, 795, 732, 701, 561, 530; HRMS (*m*/*z*): [M + H]^+^ calcd for C_25_H_28_NO_6_: 438.1911, found: 438.1933.

#### 4.1.14 Synthesis of (2*S*,3*S*,4*S*,5*S*)‐*N*,3,4‐trihydroxy‐5‐(4‐{[4‐(morpholinomethyl)phenyl]ethynyl}phenyl)tetrahydrofuran‐2‐carboxamide (**15**)

Potassium cyanide (13 mg, 0.21 mmol) and an aqueous solution of hydroxylamine (50 wt%, 5.6 mL) were added to a solution of **27** (90 mg, 0.21 mmol) in a mixture of THF and water (1/1, 2.0 mL). After stirring the reaction mixture at ambient temperature for 24 h, the volatiles were removed *in vacuo*, and the residue was purified by automatic flash column chromatography using a Biotage Isolera One system (10% → 100% ACN in H_2_O, Biotage SNAP KP‐C18‐HS 12 g). Fractions containing the desired product were combined and subjected to lyophilization to give **15** as a colorless solid (53 mg, 0.12 mmol, 59%). m.p. = 185°C; TLC: *R*_*f*_ = 0.26 (dichloromethane/methanol = 9/1); ${\left[\alpha \right]}_{D}^{20}$ = +129.3 (0.75, methanol); HPLC (method 2): *t*_R_ = 12.0 min, purity 99.7%.

#### 4.1.15 Synthesis of (2*R*,3*R*,4*R*,5*R*)‐*N*,3,4‐trihydroxy‐5‐(4‐{[4‐(morpholinomethyl)phenyl]ethynyl}phenyl)tetrahydrofuran‐2‐carboxamide (*ent*‐**15**)

Potassium cyanide (12 mg, 0.18 mmol) and an aqueous solution of hydroxylamine (50 wt%, 4.9 mL) were added to a solution of *ent*‐**27** (78 mg, 0.18 mmol) in a mixture of THF and methanol (1/1, 1.8 mL). After stirring the reaction mixture at ambient temperature for 24 h, the volatiles were removed *in vacuo*, and the residue was purified by automatic flash column chromatography using a Biotage Isolera One system (5% → 100% ACN in H_2_O, Biotage SNAP KP‐C18‐HS 12 g). Fractions containing the desired product were combined and subjected to lyophilization to give *ent*‐**15** as a colorless solid (59 mg, 0.13 mmol, 75%). m.p. = 183°C; TLC: *R*_*f*_ = 0.26 (dichloromethane/methanol = 9/1); ${\left[\alpha \right]}_{D}^{20}$ = ‐82.4 (5.0, methanol); HPLC (method 2): *t*_R_ = 12.1 min, purity 99.7%.

Spectroscopic data of **15** and *ent*‐**15**: ^1^H NMR (500 MHz, DMSO‐*d*_*6*_): *δ* [ppm] = 2.31–2.40 (m, 4H, N(C*H*_2_CH_2_)_2_O), 3.49 (s, 2H, NC*H*_2_Ar), 3.56–3.60 (m, 4H, N(CH_2_C*H*_2_)_2_O), 3.88–3.93 (m, 1H, 4‐H), 4.15 (d, *J* = 1.4 Hz, 1H, 2‐H), 4.21–4.23 (m, 1H, 3‐H), 5.10 (d, *J* = 3.1 Hz, 1H, 5‐H), 5.35 (d, *J* = 7.7 Hz, 1H, 4‐O*H*), 5.78 (d, *J* = 4.1 Hz, 1H, 3‐O*H*), 7.34–7.38 (m, 2H, 3''‐H_4‐(morpholinomethyl)phenyl_, 5''‐H_4‐(morpholinomethyl)phenyl_), 7.45–7.53 (m, 6H, 2′‐H_4‐{[4‐(morpholinomethyl)phenyl]ethynyl}phenyl_, 3′‐H_4‐{[4‐(morpholinomethyl)phenyl]ethynyl}phenyl_, 5′‐H_4‐{[4‐(morpholinomethyl)phenyl]ethynyl}phenyl_, 6′‐H_4‐{[4‐(morpholinomethyl)phenyl]ethynyl}phenyl_, 2''‐H_4‐(morpholinomethyl)phenyl_, 6''‐H_4‐(morpholinomethyl)phenyl_), 9.17 (s br, 1H, CON*H*OH), 10.79 (s br, 1H, CONHO*H*); ^13^C NMR (126 MHz, DMSO‐*d*_*6*_): *δ* [ppm] = 53.1 (2C, N(*C*H_2_CH_2_)_2_O), 62.0 (1C, Ar*C*H_2_N), 66.2 (2C, N(CH_2_ *C*H_2_)_2_O), 78.2 (1C, C‐4), 79.9 (1C, C‐3), 83.7 (1C, C‐2), 83.9 (1C, C‐5), 89.0 (1C, *C*≡C), 89.3 (1C, *C*≡C), 120.87 (1C, C_arom._), 120.95 (1C, C_arom._), 127.7 (2C, C‐2′_4‐{[4‐(morpholinomethyl)phenyl]ethynyl}phenyl_, C‐6′_4‐{[4‐(morpholinomethyl)phenyl]ethynyl}phenyl_), 129.2 (2C, C‐3''_4__‐(morpholinomethyl)phenyl_, C‐5''_4__‐(morpholinomethyl)phenyl_), 130.5 (2C, C‐3′_4‐{[4‐(morpholinomethyl)phenyl]ethynyl}phenyl_, C‐5′_4‐{[4‐(morpholinomethyl)phenyl]ethynyl}phenyl_), 131.2 (2C, C‐2''_4__‐(morpholinomethyl)phenyl_, C‐6''_4__‐(morpholinomethyl)phenyl_), 138.3 (1C, C‐1′_4‐{[4‐(morpholinomethyl)phenyl]ethynyl}phenyl_), 138.7 (1C, C‐4''_4__‐(morpholinomethyl)phenyl_), 167.4 (1C, *C*ONHOH); IR (neat): $\tilde{\nu }$ [cm^–^^1^] = 3431, 3387, 2954, 2930, 2857, 2807, 2761, 1664, 1541, 1519, 1451, 1409, 1396, 1348, 1313, 1301, 1289, 1261, 1232, 1213, 1161, 1115, 1087, 1059, 1035, 1008, 948, 914, 867, 844, 829, 784, 733, 713, 684, 624, 615, 586, 545, 528, 502, 491, 477, 466, 457, 430; HRMS (*m*/*z*): [M + H]^+^ calcd for C_24_H_27_N_2_O_6_: 439.1864, found: 439.1869.

#### 4.1.16 Synthesis of (2*S*,3*S*,4*S*,5*R*)‐*N*,3,4‐trihydroxy‐5‐(4‐{[4‐(morpholinomethyl)phenyl]ethynyl}phenyl)tetrahydrofuran‐2‐carboxamide (**16**)

Potassium cyanide (17 mg, 0.27 mmol) and an aqueous solution of hydroxylamine (50 wt%, 7.3 mL) were added to a solution of **28** (120 mg, 0.27 mmol) in a mixture of THF and methanol (1/1, 2.7 mL). After stirring the reaction mixture at ambient temperature for 24 h, the volatiles were removed *in vacuo*, and the residue was purified by automatic flash column chromatography using a Biotage Isolera One system (10% → 100% ACN in H_2_O, Biotage SNAP KP‐C18‐HS 12 g). Fractions containing the desired product were combined and subjected to lyophilization to give **16** as a colorless solid (88 mg, 0.20 mmol, 75%). m.p. = 137°C; TLC: *R*_*f*_ = 0.20 (dichloromethane/methanol = 9/1); ${\left[\alpha \right]}_{D}^{20}$ = +11.2 (1.3, methanol); HPLC (method 2): *t*_R_ = 11.8 min, purity 100%.

#### 4.1.17 Synthesis of (2*R*,3*R*,4*R*,5*S*)‐*N*,3,4‐trihydroxy‐5‐(4‐{[4‐(morpholinomethyl)phenyl]ethynyl}phenyl)tetrahydrofuran‐2‐carboxamide (*ent*‐**16**)

Potassium cyanide (0.10 mg, 0.0015 mmol) and an aqueous solution of hydroxylamine (50 wt%, 0.17 mL) were added to a solution of *ent*‐**28** (5.0 mg, 0.011 mmol) in DMSO (1.0 mL). After stirring the reaction mixture at ambient temperature overnight, it was purified by automatic flash column chromatography using a Biotage Isolera One system (10% → 100% ACN in H_2_O, Biotage SNAP KP‐C18‐HS 12 g). Fractions containing the desired product were combined and subjected to lyophilization to give *ent*‐**16** as a colorless solid (2.0 mg, 0.0046 mmol, 40%). m.p. = 143°C (decomposition); TLC: *R*_*f*_ = 0.20 (dichloromethane/methanol = 9/1); ${\left[\alpha \right]}_{D}^{20}$ = ‐3.3 (0.3, methanol); HPLC (method 2): *t*_R_ = 11.8 min, purity 100%.

Spectroscopic data of **16** and *ent*‐**16**: ^1^H NMR (500 MHz, DMSO‐*d*_*6*_): *δ* [ppm] = 2.33–2.38 (m, 4H, N(C*H*_2_CH_2_)_2_O), 3.49 (s, 2H, NC*H*_2_Ar), 3.55–3.60 (m, 4H, N(CH_2_C*H*_2_)_2_O), 3.74–3.80 (m, 1H, 4‐H), 4.20–4.25 (m, 2H, 2‐H, 3‐H), 4.81 (d, *J* = 6.5 Hz, 1H, 5‐H), 5.50–5.60 (m, 2H, 3‐O*H*, 4‐O*H*), 7.33–7.39 (m, 2H, 3''‐H_4‐(morpholinomethyl)phenyl_, 5''‐H_4‐(morpholinomethyl)phenyl_), 7.39–7.44 (m, 2H, 2′‐H_4‐{[4‐(morpholinomethyl)phenyl]ethynyl}phenyl_, 6′‐H_4‐{[4‐(morpholinomethyl)phenyl]ethynyl}phenyl_), 7.48–7.55 (m, 4H, 3′‐H_4‐{[4‐(morpholinomethyl)phenyl]ethynyl}phenyl_, 5′‐H_4‐{[4‐(morpholinomethyl)phenyl]ethynyl}phenyl_, 2''‐H_4‐(morpholinomethyl)phenyl_, 6''‐H_4‐(morpholinomethyl)phenyl_), 8.88 (s br, 1H, CON*H*OH), 10.73 (s br, 1H, CONHO*H*); ^13^C NMR (126 MHz, DMSO‐*d*_*6*_): *δ* [ppm] = 53.1 (2C, N(*C*H_2_CH_2_)_2_O), 62.0 (1C, Ar*C*H_2_N), 66.2 (2C, N(CH_2_ *C*H_2_)_2_O), 79.8 (1C, C‐3), 82.1 (1C, C‐2), 83.3 (1C, C‐4), 84.8 (1C, C‐5), 89.1 (1C, *C*≡C), 89.2 (1C, *C*≡C), 120.9 (1C, C‐1''_4__‐(morpholinomethyl)phenyl_), 121.2 (1C, C‐4′_4‐{[4‐(morpholinomethyl)phenyl]ethynyl}phenyl_), 126.4 (2C, C‐2′_4‐{[4‐(morpholinomethyl)phenyl]ethynyl}phenyl_, C‐6′_4‐{[4‐(morpholinomethyl)phenyl]ethynyl}phenyl_), 129.2 (2C, C‐3''_4__‐(morpholinomethyl)phenyl_, C‐5''_4__‐(morpholinomethyl)phenyl_), 131.1 (2C, C_arom._), 131.2 (2C, C_arom._), 138.8 (1C, C‐4''_4__‐(morpholinomethyl)phenyl_), 141.6 (1C, C‐1′_4‐{[4‐(morpholinomethyl)phenyl]ethynyl}phenyl_), 167.1 (1C, *C*ONHOH); IR (neat): $\tilde{\nu }$ [cm^–1^] = 3217, 2905, 2814, 1670, 1516, 1454, 1411, 1352, 1335, 1293, 1262, 1210, 1107, 1067, 1055, 1017, 1005, 952, 916, 863, 827, 792, 746, 710, 679, 652, 626, 553, 537, 502, 486, 467; HRMS (*m*/*z*): [M + H]^+^ calcd for C_24_H_27_N_2_O_6_: 439.1864, found: 439.1880.

#### 4.1.18 Synthesis of (3*R*,4*S*,5*S*)‐3,4‐bis[(*tert*‐butyldiphenylsilyl)oxy]‐5‐[(*R*)‐2,2‐dimethyl‐1,3‐dioxolan‐4‐yl]dihydrofuran‐2(3*H*)‐one (**31**)

*tert*‐Butyldiphenylsilyl chloride (12 mL, 13 g, 22 mmol) was added to a solution of **30** (4.7 g, 22 mmol) and imidazole (4.8 g, 71 mmol) in dichloromethane (110 mL). After stirring the reaction mixture at ambient temperature overnight, a saturated aqueous solution of NaHCO_3_ (20 mL) was added, and the aqueous phase was extracted with dichloromethane (3×). The combined organic layers were dried (Na_2_SO_4_), filtered, and the solvent was removed *in vacuo*. The residue was purified by flash column chromatography (petroleum ether/ethyl acetate = 15/1) to give **31** as a colorless solid (14 g, 21 mmol, 96%). m.p. = 140°C; TLC: *R*_*f*_ = 0.38 (petroleum ether/ethyl acetate = 10/1); ${\left[\alpha \right]}_{D}^{20}$ = ‐52.0 (4.5, dichloromethane); ^1^H NMR (500 MHz, CDCl_3_): *δ* [ppm] = 0.90 (s, 9H, SiC(C*H*_3_)_3_), 1.04 (s, 9H, SiC(C*H*_3_)_3_), 1.24 (s, 3H, C(C*H*_3_)_2_), 1.28 (s, 3H, C(C*H*_3_)_2_), 2.84 (t, *J* = 8.0 Hz, 1H, OC*H*_2_CHCHCHCHC═O), 3.45 (dd, *J* = 8.3/6.7 Hz, 1H, OC*H*_2_CHCHCHCHC═O), 3.84–3.90 (m, 1H, OCH_2_C*H*CHCHCHC═O), 4.03–4.09 (m, 2H, OCH_2_CHC*H*C*H*CHC═O), 4.39 (d, *J* = 2.3 Hz, 1H, OCH_2_CHCHCHC*H*C═O), 7.27–7.49 (m, 14H, 2′‐H_diphenylsilyl_ (1H), 3′‐H_diphenylsilyl_ (4H), 4′‐H_diphenylsilyl_ (4H), 5′‐H_diphenylsilyl_ (4H), 6′‐H_diphenylsilyl_ (1H)), 7.49 – 7.55 (m, 2H, 2′‐H_diphenylsilyl_, 6′‐H_diphenylsilyl_), 7.60–7.66 (m, 2H, 2′‐H_diphenylsilyl_, 6′‐H_diphenylsilyl_), 7.68–7.73 (m, 2H, 2′‐H_diphenylsilyl_, 6′‐H_diphenylsilyl_); ^13^C NMR (126 MHz, CDCl_3_): *δ* [ppm] = 19.1 (1C, Si*C*(CH_3_)_3_), 19.4 (1C, Si*C*(CH_3_)_3_), 25.6 (1C, C(*C*H_3_)_2_), 26.3 (1C, C(*C*H_3_)_2_), 26.8 (3C, SiC(*C*H_3_)_3_), 26.9 (3C, SiC(*C*H_3_)_3_), 64.7 (1C, O*C*H_2_CHCHCHCHC═O), 74.8 (1C, OCH_2_ *C*HCHCHCHC═O), 75.9 (1C, OCH_2_CHCHCH*C*HC═O), 76.3 (1C, OCH_2_CHCH*C*HCHC═O), 85.7 (1C, OCH_2_CH*C*HCHCHC═O), 110.1 (1C, *C*(CH_3_)_2_), 127.9 (2C, C‐3′_diphenylsilyl_, C‐5′_diphenylsilyl_), 128.0 (2C, C‐3′_diphenylsilyl_, C‐5′_diphenylsilyl_), 128.1 (2C, C‐3′_diphenylsilyl_, C‐5′_diphenylsilyl_), 128.2 (2C, C‐3′_diphenylsilyl_, C‐5′_diphenylsilyl_), 130.1 (1C, C‐4′_diphenylsilyl_), 130.2 (1C, C‐4′_diphenylsilyl_), 130.38 (1C, C‐4′_diphenylsilyl_), 130.44 (1C, C‐4′_diphenylsilyl_), 131.7 (1C, C‐1′_diphenylsilyl_), 132.1 (1C, C‐1′_diphenylsilyl_), 132.7 (1C, C‐1′_diphenylsilyl_), 132.8 (1C, C‐1′_diphenylsilyl_), 135.77 (2C, C‐2′_diphenylsilyl_, C‐6′_diphenylsilyl_), 135.78 (2C, C‐2′_diphenylsilyl_, C‐6′_diphenylsilyl_), 135.85 (2C, C‐2′_diphenylsilyl_, C‐6′_diphenylsilyl_), 136.1 (2C, C‐2′_diphenylsilyl_, C‐6′_diphenylsilyl_), 173.5 (1C, OCH_2_CHCHCHCH*C* = O); IR (neat): $\tilde{\nu }$ [cm^–1^] = 3072, 3046, 2954, 2932, 2895, 2858, 1809, 1791, 1588, 1471, 1428, 1392, 1371, 1335, 1318, 1298, 1246, 1220, 1163, 1109, 1059, 1036, 997, 967, 946, 924, 902, 861, 847, 821, 798, 784, 766, 741, 702, 674, 609, 531, 509, 496, 485, 471, 438; HRMS (*m*/*z*): [M + H]^+^ calcd for C_41_H_50_NaO_6_Si_2_: 717.3038, found: 717.3022; HPLC (method 1): *t*_R_ = 25.4 min, purity 100%.

#### 4.1.19 Synthesis of (3*R*,4*S*,5*S*)‐3,4‐bis[(*tert*‐butyldiphenylsilyl)oxy]‐5‐[(*R*)‐2,2‐dimethyl‐1,3‐dioxolan‐4‐yl]‐2‐(4‐iodophenyl)tetrahydrofuran‐2‐ol (**32**)

Under N_2_ atmosphere, a solution of 1,4‐diiodobenzene (17 g, 52 mmol) in dry THF (210 mL) was cooled to –78°C, and a 1.6 M solution of *n*‐butyllithium in hexanes (20 mL, 31 mmol) was added dropwise. After stirring the mixture at –78°C for 15 min, a solution of **31** (18 g, 26 mmol) in dry THF (65 mL) was added dropwise and the mixture was stirred for an additional 30 min at –78°C. Then, a saturated aqueous solution of NH_4_Cl was added, and the mixture was warmed to ambient temperature. After the addition of water, the mixture was extracted with dichloromethane (3×). The combined organic layers were dried (Na_2_SO_4_), filtered, and the solvent was removed *in vacuo*. The residue was purified by flash column chromatography (petroleum ether/ethyl acetate = 9/1, *R*_*f*_ = 0.40) to give **32** as a yellowish solid (23 g, 26 mmol, quant.). m.p. = 118°C; ^1^H NMR (400 MHz, DMSO‐*d*_*6*_): *δ* [ppm] = 0.56 (s, 9H, SiC(C*H*_3_)_3_), 0.79 (s, 9H, SiC(C*H*_3_)_3_), 1.17 (s, 3H, C(C*H*_3_)_2_), 1.23 (s, 3H, C(C*H*_3_)_2_), 2.83 (t, *J* = 8.0 Hz, 1H, OC*H*_2_CHCHCHCH), 3.18–3.23 (m, 1H, OC*H*_2_CHCHCHCH), 3.46–3.54 (m, 1H, OCH_2_C*H*CHCHCH), 3.94 (dd, *J* = 5.7/2.1 Hz, 1H, OCH_2_CHC*H*CHCH), 4.12 (d, *J* = 2.1 Hz, 1H, OCH_2_CHCHC*H*CH), 4.35 (s, 1H, OCH_2_CHCHCHC*H*), 6.47 (s, 1H, O*H*), 7.21–7.59 (m, 22H, H_arom._), 7.63–7.68 (m, 2H, 3′‐H_4‐iodophenyl_, 5′‐H_4‐iodophenyl_), two epimers exist in the ratio 4:1, the signals of the major epimer are given; ^13^C NMR (101 MHz, DMSO‐*d*_*6*_): *δ* [ppm] = 18.3 (1C, Si*C*(CH_3_)_3_), 18.7 (1C, Si*C*(CH_3_)_3_), 25.3 (1C, C(*C*H_3_)_2_), 26.1 (3C, SiC(*C*H_3_)_3_), 26.38 (3C, SiC(*C*H_3_)_3_), 26.42 (1C, C(*C*H_3_)_2_), 64.4 (1C, O*C*H_2_CHCHCHCH), 75.0 (1C, OCH_2_ *C*HCHCHCH), 80.7 (1C, OCH_2_CHCH*C*HCH), 84.8 (1C, OCH_2_CHCHCH*C*H), 85.4 (1C, OCH_2_CH*C*HCHCH), 94.1 (1C, C‐4′_4‐iodophenyl_), 107.6 (1C, OCH_2_CHCHCHCH*C*OH), 108.2 (1C, *C*(CH_3_)_2_), 127.5 (2C, C‐3''_d__iphenylsilyl_, C‐5''_d__iphenylsilyl_), 127.6 (2C, C‐3''_d__iphenylsilyl_, C‐5''_d__iphenylsilyl_), 127.73 (2C, C‐3''_d__iphenylsilyl_, C‐5''_d__iphenylsilyl_), 127.75 (2C, C‐3''_d__iphenylsilyl_, C‐5''_d__iphenylsilyl_), 129.7 (1C, C‐4''_d__iphenylsilyl_), 129.8 (1C, C‐4''_d__iphenylsilyl_), 129.9 (4C, C‐2′_4‐iodophenyl_, C‐6′_4‐iodophenyl_, 2 C‐4''_d__iphenylsilyl_), 131.3 (1C, C‐1''_d__iphenylsilyl_), 132.6 (1C, C‐1''_d__iphenylsilyl_), 132.8 (1C, C‐1''_d__iphenylsilyl_), 132.9 (1C, C‐1''_d__iphenylsilyl_), 135.2 (2C, C‐2''_d__iphenylsilyl_, C‐6''_d__iphenylsilyl_), 135.4 (4C, C‐2''_d__iphenylsilyl_, C‐6''_d__iphenylsilyl_), 135.5 (2C, C‐2''_d__iphenylsilyl_, C‐6''_d__iphenylsilyl_), 136.0 (2C, C‐3′_4‐iodophenyl_, C‐5′_4‐iodophenyl_), 141.3 (1C, C‐1′_4‐iodophenyl_), the signals of the major epimer are given; IR (neat): $\tilde{\nu }$ [cm^–1^] = 3485, 3071, 3048, 2931, 2890, 2857, 1793, 1590, 1486, 1471, 1427, 1390, 1369, 1259, 1212, 1104, 1051, 999, 977, 938, 895, 859, 820, 795, 739, 699, 610, 502, 486; HRMS (*m*/*z*): [M+Na]^+^ calcd for C_47_H_55_INaO_6_Si_2_: 921.2474, found: 921.2516.

#### 4.1.20 Synthesis of (3*R*,4*S*,5*S*)‐2‐[4‐(benzyloxy)phenyl]‐3,4‐bis[(*tert*‐butyldiphenylsilyl)oxy]‐5‐[(*R*)‐2,2‐dimethyl‐1,3‐dioxolan‐4‐yl]tetrahydrofuran‐2‐ol (**33**)

Under N_2_ atmosphere, a solution of 4‐benzyloxybromobenzene (5.7 g, 22 mmol) in dry THF (75 mL) was cooled to –78°C, and a 1.6 M solution of *n*‐butyllithium in hexanes (10 mL, 16 mmol) was added dropwise. After stirring the mixture at –78°C for 30 min, a solution of **31** (8.7 g, 13 mmol) in dry THF (40 mL) was added dropwise, and the mixture was stirred for an additional 45 min at –78°C. Then, a saturated aqueous solution of NH_4_Cl (20 mL) was added, and the mixture was warmed to ambient temperature. After the addition of water (30 mL), the mixture was extracted with dichloromethane (3× 150 mL). The combined organic layers were dried (Na_2_SO_4_), filtered, and the solvent was removed *in vacuo*. The residue was purified by flash column chromatography (petroleum ether/ethyl acetate = 9/1, *R*_*f*_ = 0.24) to give **33** as a colorless solid (7.0 g, 8.0 mmol, 64%). m.p. = 53°C; ^1^H NMR (500 MHz, DMSO‐*d*_*6*_): *δ* [ppm] = 0.55 (s, 9H, SiC(C*H*_3_)_3_), 0.79 (s, 9H, SiC(C*H*_3_)_3_), 1.17 (s, 3H, C(C*H*_3_)_2_), 1.22 (s, 3H, C(C*H*_3_)_2_), 2.74 (t, *J* = 8.0 Hz, 1H, OC*H*_2_CHCHCHCH), 3.15–3.19 (m, 1H, OC*H*_2_CHCHCHCH), 3.52 –3.58 (m, 1H, OCH_2_C*H*CHCHCH), 3.90 (dd, *J* = 5.9/2.0 Hz, 1H, OCH_2_CHC*H*CHCH), 4.07 (d, *J* = 2.0 Hz, 1H, OCH_2_CHCHC*H*CH), 4.31 (s, 1H, OCH_2_CHCHCHC*H*), 5.09 (d, *J* = 12.6 Hz, 1H, OC*H*_*2*_Ph), 5.12 (d, *J* = 12.6 Hz, 1H, OC*H*_2_Ph), 6.23 (s, 1H, O*H*), 6.91–6.96 (m, 2H, 3′‐H_4‐(benzyloxy)phenyl_, 5′‐H_4‐(benzyloxy)phenyl_), 7.20–7.50 (m, 25H, H_arom._), 7.51–7.56 (m, 2H, 2'''‐H_diphenylsilyl_, 6'''‐H_diphenylsilyl_), two epimers exist in the ratio 4:1, the signals of the major epimer are given; ^13^C NMR (126 MHz, DMSO‐*d*_*6*_): *δ* [ppm] = 18.4 (1C, Si*C*(CH_3_)_3_), 18.7 (1C, Si*C*(CH_3_)_3_), 25.4 (1C, C(*C*H_3_)_2_), 26.2 (3C, SiC(*C*H_3_)_3_), 26.40 (3C, SiC(*C*H_3_)_3_), 26.45 (1C, C(*C*H_3_)_2_), 64.3 (1C, O*C*H_2_CHCHCHCH), 69.1 (1C, O*C*H_2_Ph), 75.1 (1C, OCH_2_ *C*HCHCHCH), 80.7 (1C, OCH_2_CHCH*C*HCH), 84.5 (1C, OCH_2_CHCHCH*C*H), 85.2 (1C, OCH_2_CH*C*HCHCH), 107.9 (1C, OCH_2_CHCHCHCH*C*OH), 108.2 (1C, *C*(CH_3_)_2_), 113.5 (2C, C‐3′_4‐(benzyloxy)phenyl_, C‐5′_4‐(benzyloxy)phenyl_), 127.4 (2C, C‐2''_b__enzyl_, C‐6''_b__enzyl_), 127.5 (4C, C‐3'''_d__iphenylsilyl_, C‐5'''_d__iphenylsilyl_), 127.70 (2C, C‐3'''_d__iphenylsilyl_, C‐5'''_d__iphenylsilyl_), 127.73 (2C, C‐3'''_d__iphenylsilyl_, C‐5'''_d__iphenylsilyl_), 128.4 (2C, C‐3''_b__enzyl_, C‐5''_b__enzyl_), 128.9 (2C, C‐2′_4‐(benzyloxy)phenyl_, C‐6′_4‐(benzyloxy)phenyl_), 129.68 (1C, C_arom._), 129.71 (1C, C_arom._), 129.88 (2C, C_arom._), 129.93 (1C, C_arom._), 131.4 (1C, C‐1'''_d__iphenylsilyl_), 132.6 (1C, C‐1'''_d__iphenylsilyl_), 132.9 (1C, C‐1'''_d__iphenylsilyl_), 133.0 (1C, C‐1'''_d__iphenylsilyl_), 133.7 (1C, C‐1′_4‐(benzyloxy)phenyl_), 135.2 (2C, C‐2'''_d__iphenylsilyl_, C‐6'''_d__iphenylsilyl_), 135.4 (4C, C‐2'''_d__iphenylsilyl_, C‐6'''_d__iphenylsilyl_), 135.6 (2C, C‐2'''_d__iphenylsilyl_, C‐6'''_d__iphenylsilyl_), 137.2 (1C, C‐1''_b__enzyl_), 158.0 (1C, C‐4′_4‐(benzyloxy)phenyl_), the signals of the major epimer are given; IR (neat): $\tilde{\nu }$ [cm^‐1^] = 3489, 3071, 3048, 2930, 2857, 1739, 1611, 1589, 1512, 1471, 1427, 1379, 1369, 1309, 1241, 1221, 1172, 1105, 1047, 1005, 940, 915, 861, 822, 787, 739, 699, 610, 502, 486; HRMS (*m*/*z*): [M+Na]^+^ calcd for C_54_H_62_NaO_7_Si_2_: 901.3926, found: 901.3929; HPLC (method 3): *t*_R_ = 34.3 min, purity 93.0%.

#### 4.1.21 Synthesis of ({(2*S*,3*S*,4*S*,5*R*)‐2‐[(*R*)‐2,2‐dimethyl‐1,3‐dioxolan‐4‐yl]‐5‐(4‐iodophenyl)tetrahydrofuran‐3,4‐diyl}bis(oxy))bis(*tert*‐butyldiphenylsilane) (**34**)

Under N_2_ atmosphere, triethylsilane (6.3 mL, 4.6 g, 39 mmol) and boron trifluoride diethyl etherate (4.8 mL, 5.6 g, 39 mmol) were successively added to a solution of **32** (18 g, 20 mmol) in dry dichloromethane (98 mL) at –40°C. After stirring the reaction mixture at –40°C for 2 h, a saturated aqueous solution of K_2_CO_3_ (20 mL) was added, and the mixture was warmed to ambient temperature. After the addition of water (20 mL), the mixture was extracted with dichloromethane (3×). The combined organic layers were dried (Na_2_SO_4_), filtered, and the solvent was removed *in vacuo*. The residue was purified by flash column chromatography (petroleum ether/ethyl acetate = 9/1, *R*_*f*_ = 0.48) to give **34** as a colorless solid (13 g, 15 mmol, 75%). m.p. = 94°C; ${\left[\alpha \right]}_{D}^{20}$ = −3.6 (4.5, dichloromethane); ^1^H NMR (400 MHz, CDCl_3_): *δ* [ppm] = 0.76 (s, 9H, SiC(C*H*_3_)_3_), 0.96 (s, 9H, SiC(C*H*_3_)_3_), 1.28 (s, 3H, C(C*H*_3_)_2_), 1.31 (s, 3H, C(C*H*_3_)_2_), 2.14 (t, *J* = 8.3 Hz, 1H, OC*H*_2_CHCHCHCHCH), 3.08 (dd, *J* = 8.1/6.4 Hz, 1H, OC*H*_2_CHCHCHCHCH), 3.67 (s, 1H, OCH_2_CHCHC*H*CHCH), 3.80 (d, *J* = 8.9 Hz, 1H, OCH_2_CHC*H*CHCHCH), 4.24–4.31 (m, 1H, OCH_2_C*H*CHCHCHCH), 4.34–4.36 (m, 1H, OCH_2_CHCHCHC*H*CH), 5.31 (d, *J* = 2.6 Hz, 1H, OCH_2_CHCHCHCHC*H*), 6.99–7.05 (m, 2H, 2''‐H_diphenylsilyl_, 6''‐H_diphenylsilyl_), 7.11–7.20 (m, 4H, 2′‐H_4‐iodophenyl_, 6′‐H_4‐iodophenyl_, 3''‐H_diphenylsilyl_, 5''‐H_diphenylsilyl_), 7.20– 7.28 (m, 4H, 3''‐H_diphenylsilyl_ (2H), 5''‐H_diphenylsilyl_ (2H)), 7.29–7.48 (m, 12H, 2''‐H_diphenylsilyl_ (3H), 3''‐H_diphenylsilyl_, 4''‐H_diphenylsilyl_ (4H), 5''‐H_diphenylsilyl_, 6''‐H_diphenylsilyl_ (3H)), 7.58–7.64 (m, 2H, 3′‐H_4‐iodophenyl_, 5′‐H_4‐iodophenyl_); ^13^C NMR (101 MHz, CDCl_3_): *δ* [ppm] = 19.0 (1C, Si*C*(CH_3_)_3_), 19.2 (1C, Si*C*(CH_3_)_3_), 25.8 (1C, C(*C*H_3_)_2_), 26.8 (1C, C(*C*H_3_)_2_), 26.9 (6C, SiC(*C*H_3_)_3_), 64.9 (1C, O*C*H_2_CHCHCHCHCH), 76.5 (1C, OCH_2_ *C*HCHCHCHCH), 80.1 (1C, OCH_2_CHCH*C*HCHCH), 80.3 (1C, OCH_2_CHCHCH*C*HCH), 84.1 (1C, OCH_2_CHCHCHCH*C*H), 88.8 (1C, OCH_2_CH*C*HCHCHCH), 93.0 (1C, C‐4′_4‐iodophenyl_), 109.4 (1C, *C*(CH_3_)_2_), 127.6 (2C, C‐3''_d__iphenylsilyl_, C‐5''_d__iphenylsilyl_), 127.87 (2C, C‐3''_d__iphenylsilyl_, C‐5''_d__iphenylsilyl_), 127.93 (2C, C‐3''_d__iphenylsilyl_, C‐5''_d__iphenylsilyl_), 128.1 (2C, C‐3''_d__iphenylsilyl_, C‐5''_d__iphenylsilyl_), 129.8 (1C, C‐4''_d__iphenylsilyl_), 129.95 (1C, C‐4''_d__iphenylsilyl_), 130.00 (2C, C‐2′_4‐iodophenyl_, C‐6′_4‐iodophenyl_), 130.2 (1C, C‐4''_d__iphenylsilyl_), 130.3 (1C, C‐4''_d__iphenylsilyl_), 132.0 (1C, C‐1''_d__iphenylsilyl_), 132.8 (1C, C‐1''_d__iphenylsilyl_), 133.1 (1C, C‐1''_d__iphenylsilyl_), 133.4 (1C, C‐1''_d__iphenylsilyl_), 135.8 (2C, C‐2''_d__iphenylsilyl_, C‐6''_d__iphenylsilyl_), 135.9 (2C, C‐2''_d__iphenylsilyl_, C‐6''_d__iphenylsilyl_), 135.97 (2C, C‐2''_d__iphenylsilyl_, C‐6''_d__iphenylsilyl_), 136.02 (2C, C‐2''_d__iphenylsilyl_, C‐6''_d__iphenylsilyl_), 136.9 (2C, C‐3′_4‐iodophenyl_, C‐5′_4‐iodophenyl_), 137.0 (1C, C‐1′_4‐iodophenyl_); IR (neat): $\tilde{\nu }$ [cm^–1^] = 3071, 3048, 2931, 2890, 2857, 1966, 1904, 1735, 1590, 1485, 1471, 1427, 1391, 1368, 1259, 1212, 1106, 1059, 1006, 932, 898, 844, 820, 795, 740, 700, 610, 503, 487, 420; HRMS (*m*/*z*): [M+Na]^+^ calcd for C_47_H_55_INaO_5_Si_2_: 905.2525, found: 905.2512.

#### 4.1.22 Synthesis of ({(2*R*,3*S*,4*S*,5*S*)‐2‐[4‐(benzyloxy)phenyl]‐5‐[(*R*)‐2,2‐dimethyl‐1,3‐dioxolan‐4‐yl]tetrahydrofuran‐3,4‐diyl}bis(oxy))bis(*tert*‐butyldiphenylsilane) (**35**)

Under N_2_ atmosphere, triethylsilane (2.5 mL, 1.8 g, 16 mmol) and boron trifluoride diethyl etherate (1.8 mL, 2.1 g, 15 mmol) were successively added to a solution of **33** (6.9 g, 7.8 mmol) in dry dichloromethane (40 mL) at –40°C. After stirring the reaction mixture at –40°C for 80 min, a saturated aqueous solution of K_2_CO_3_ (13 mL) was added, and the mixture was warmed to ambient temperature. After the addition of water (30 mL), the mixture was extracted with dichloromethane (3×). The combined organic layers were dried (Na_2_SO_4_), filtered, and the solvent was removed *in vacuo*. The residue was purified by flash column chromatography (petroleum ether/ethyl acetate = 9/1, *R*_*f*_ = 0.33) to give **35** as a colorless solid (5.7 g, 6.6 mmol, 85%). m.p. = 46°C; ${\left[\alpha \right]}_{D}^{20}$ = ‐1.8 (6.6, dichloromethane); ^1^H NMR (500 MHz, CDCl_3_): *δ* [ppm] = 0.78 (s, 9H, SiC(C*H*_3_)_3_), 0.95 (s, 9H, SiC(C*H*_3_)_3_), 1.25 (s, 3H, C(C*H*_3_)_2_), 1.30 (s, 3H, C(C*H*_3_)_2_), 2.01 (t, *J* = 8.3 Hz, 1H, OC*H*_2_CHCHCHCHCH), 3.06 (dd, *J* = 8.1/6.4 Hz, 1H, OC*H*_2_CHCHCHCHCH), 3.55 (s, 1H, OCH_2_CHCHC*H*CHCH), 3.75 (d, *J* = 8.9 Hz, 1H, OCH_2_CHC*H*CHCHCH), 4.27–4.33 (m, 2H, OCH_2_C*H*CHCHC*H*CH), 5.06–5.12 (m, 2H, OC*H*_2_Ph), 5.36 (d, *J* = 2.5 Hz, 1H, OCH_2_CHCHCHCHC*H*), 6.93–6.97 (m, 2H, 3′‐H_4‐(benzyloxy)phenyl_, 5′‐H_4‐(benzyloxy)phenyl_), 6.97–7.01 (m, 2H, 2'''‐H_diphenylsilyl_, 6'''‐H_diphenylsilyl_), 7.11–7.16 (m, 2H, 3'''‐H_diphenylsilyl_, 5'''‐H_diphenylsilyl_), 7.16–7.20 (m, 2H, 3'''‐H_diphenylsilyl_, 5'''‐H_diphenylsilyl_), 7.20–7.25 (m, 2H, 3'''‐H_diphenylsilyl_, 5'''‐H_diphenylsilyl_), 7.28–7.46 (m, 19H, H_arom._); ^13^C NMR (126 MHz, CDCl_3_): *δ* [ppm] = 19.0 (1C, Si*C*(CH_3_)_3_), 19.2 (1C, Si*C*(CH_3_)_3_), 25.9 (1C, C(*C*H_3_)_2_), 26.8 (1C, C(*C*H_3_)_2_), 26.9 (3C, SiC(*C*H_3_)_3_), 27.0 (3C, SiC(*C*H_3_)_3_), 64.8 (1C, O*C*H_2_CHCHCHCHCH), 70.2 (1C, O*C*H_2_Ph), 76.6 (1C, OCH_2_ *C*HCHCHCHCH), 80.0 (1C, OCH_2_CHCH*C*HCHCH), 80.3 (1C, OCH_2_CHCHCH*C*HCH), 84.3 (1C, OCH_2_CHCHCHCH*C*H), 88.4 (1C, OCH_2_CH*C*HCHCHCH), 109.3 (1C, *C*(CH_3_)_2_), 114.5 (2C, C‐3′_4‐(benzyloxy)phenyl_, C‐5′_4‐(benzyloxy)phenyl_), 127.5 (4C, C‐2''_b__enzyl_, C‐6''_b__enzyl_, C‐3'''_d__iphenylsilyl_, C‐5'''_d__iphenylsilyl_), 127.8 (2C, C‐3'''_d__iphenylsilyl_, C‐5'''_d__iphenylsilyl_), 127.9 (3C, C‐4''_b__enzyl_, C‐3'''_d__iphenylsilyl_, C‐5'''_d__iphenylsilyl_), 128.0 (2C, C‐3'''_d__iphenylsilyl_, C‐5'''_d__iphenylsilyl_), 128.7 (2C, C‐3''_b__enzyl_, C‐5''_b__enzyl_), 129.4 (2C, C‐2′_4‐(benzyloxy)phenyl_, C‐6′_4‐(benzyloxy)phenyl_), 129.5 (1C, C‐1′_4‐(benzyloxy)phenyl_), 129.6 (1C, C‐4'''_d__iphenylsilyl_), 129.9 (1C, C‐4'''_d__iphenylsilyl_), 130.1 (1C, C‐4'''_d__iphenylsilyl_), 130.2 (1C, C‐4'''_d__iphenylsilyl_), 132.1 (1C, C‐1'''_d__iphenylsilyl_), 132.9 (1C, C‐1'''_d__iphenylsilyl_), 133.2 (1C, C‐1'''_d__iphenylsilyl_), 133.6 (1C, C1'''_d__iphenylsilyl_), 135.86 (2C, C‐2'''_d__iphenylsilyl_, C‐6'''_d__iphenylsilyl_), 135.91 (2C, C‐2'''_d__iphenylsilyl_, C‐6'''_d__iphenylsilyl_), 136.0 (2C, C‐2'''_d__iphenylsilyl_, C‐6'''_d__iphenylsilyl_), 136.2 (2C, C‐2'''_d__iphenylsilyl_, C‐6'''_d__iphenylsilyl_), 137.4 (1C, C‐1''_b__enzyl_), 158.5 (1C, C‐4′_4‐(benzyloxy)phenyl_); IR (neat): $\tilde{\nu }$ [cm^–1^] = 3070, 2931, 2891, 2857, 1738, 1613, 1588, 1512, 1487, 1471, 1427, 1369, 1302, 1240, 1220, 1172, 1104, 1053, 1008, 945, 898, 859, 822, 799, 739, 698, 641, 610, 502, 486; HRMS (*m*/*z*): [M+Na]^+^ calcd for C_54_H_62_NaO_6_Si_2_: 885.3977, found: 885.3962; HPLC (method 3): *t*_R_ = 34.9 min, purity 98.9%.

#### 4.1.23 Synthesis of (*R*)‐1‐{(2*S*,3*S*,4*S*,5*R*)‐3,4‐bis[(*tert*‐butyldiphenylsilyl)oxy]‐5‐(4‐iodophenyl)tetrahydrofuran‐2‐yl}ethane‐1,2‐diol (**36**)

*p*‐Toluenesulfonic acid monohydrate (69 mg, 0.36 mmol) was added to a solution of **34** (7.4 g, 8.3 mmol) in methanol (200 mL), and the mixture was stirred at ambient temperature for 12 h. After the addition of a saturated aqueous solution of NaHCO_3_ (10 mL) and water (100 mL), the mixture was extracted with dichloromethane (3×). The combined organic layers were dried (Na_2_SO_4_), filtered, and the solvent was removed *in vacuo*. The residue was purified by flash column chromatography (petroleum ether/ethyl acetate = 4/1, *R*_*f*_ = 0.28) to give **36** as a colorless solid (4.2 g, 5.0 mmol, 60%). m.p. = 151°C; ${\left[\alpha \right]}_{D}^{20}$ = +14.9 (3.8, dichloromethane); ^1^H NMR (400 MHz, DMSO‐*d*_*6*_): *δ* [ppm] = 0.63 (s, 9H, SiC(C*H*_3_)_3_), 0.87 (s, 9H, SiC(C*H*_3_)_3_), 2.87– 2.95 (m, 1H, HOC*H*_2_CHOH), 3.00–3.08 (m, 1H, HOC*H*_2_CHOH), 3.51–3.59 (m, 1H, HOCH_2_C*H*OH), 3.83 (d, *J* = 6.8 Hz, 1H, 2‐H), 4.16 (d, *J* = 2.8 Hz, 1H, 4‐H), 4.24 (s, 1H, 3‐H), 4.34–4.39 (m, 2H, *H*OCH_2_CHO*H*), 5.08 (d, *J* = 2.6 Hz, 1H, 5‐H), 6.91–6.96 (m, 2H, 2''‐H_diphenylsilyl_, 6''‐H_diphenylsilyl_), 7.07–7.18 (m, 4H, 2′‐H_4‐iodophenyl_, 6′‐H_4‐iodophenyl_, 3''‐H_diphenylsilyl_, 5''‐H_diphenylsilyl_), 7.18–7.31 (m, 6H, 2''‐H_diphenylsilyl_, 3''‐H_diphenylsilyl_ (2H), 5''‐H_diphenylsilyl_ (2H), 6''‐H_diphenylsilyl_), 7.32–7.50 (m, 10H, 2''‐H_diphenylsilyl_ (2H), 3''‐H_diphenylsilyl_, 4''‐H_diphenylsilyl_ (4H), 5''‐H_diphenylsilyl_, 6''‐H_diphenylsilyl_ (2H)), 7.50–7.56 (m, 2H, 3′‐H_4‐iodophenyl_, 5′‐H_4‐iodophenyl_); ^13^C NMR (101 MHz, DMSO‐*d*_*6*_): *δ* [ppm] = 18.3 (1C, Si*C*(CH_3_)_3_), 18.6 (1C, Si*C*(CH_3_)_3_), 26.5 (3C, SiC(*C*H_3_)_3_), 26.6 (3C, SiC(*C*H_3_)_3_), 62.6 (1C, HO*C*H_2_CHOH), 71.5 (1C, HOCH_2_ *C*HOH), 79.7 (1C, C‐4), 79.8 (1C, C‐3), 82.2 (1C, C‐5), 88.0 (1C, C‐2), 93.1 (1‐C, C‐4′_4‐iodophenyl_), 127.4 (2C, C‐3''_d__iphenylsilyl_, C‐5''_d__iphenylsilyl_), 127.5 (2C, C‐3''_d__iphenylsilyl_, C‐5''_d__iphenylsilyl_), 127.7 (2C, C‐3''_d__iphenylsilyl_, C‐5''_d__iphenylsilyl_), 127.8 (2C, C‐3''_d__iphenylsilyl_, C‐5''_d__iphenylsilyl_), 129.6 (1C, C‐4''_d__iphenylsilyl_), 129.7 (2C, C‐2′_4‐iodophenyl_, C‐6′_4‐iodophenyl_), 129.8 (1C, C‐4''_d__iphenylsilyl_), 129.9 (1C, C‐4''_d__iphenylsilyl_), 130.0 (1C, C‐4''_d__iphenylsilyl_), 131.3 (1C, C‐1''_d__iphenylsilyl_), 132.30 (1C, C‐1''_d__iphenylsilyl_), 132.35 (1C, C‐1''_d__iphenylsilyl_), 132.6 (1C, C‐1''_d__iphenylsilyl_), 135.2 (2C, C‐2''_d__iphenylsilyl_, C‐6''_d__iphenylsilyl_), 135.3 (6C, C‐2''_d__iphenylsilyl_, C‐6''_d__iphenylsilyl_), 136.2 (2C, C‐3′_4‐iodophenyl_, C‐5′_4‐iodophenyl_), 136.8 (1C, C‐1′_4‐iodophenyl_); IR (neat): $\tilde{\nu }$ [cm^–1^] = 3440, 3072, 3049, 2932, 2914, 2888, 2857, 1589, 1486, 1471, 1445, 1427, 1391, 1362, 1330, 1254, 1104, 1059, 1005, 971, 934, 897, 871, 856, 818, 794, 740, 698, 609, 501, 484, 437; HRMS (*m*/*z*): [M+Na]^+^ calcd for C_44_H_51_INaO_5_Si_2_: 865.2212, found: 865.2233.

#### 4.1.24 Synthesis of (*R*)‐1‐{(2*S*,3*S*,4*S*,5*R*)‐5‐[4‐(benzyloxy)phenyl]‐3,4‐bis[(*tert*‐butyldiphenylsilyl)oxy]tetrahydrofuran‐2‐yl}ethane‐1,2‐diol (**37**)

*p*‐Toluenesulfonic acid monohydrate (120 mg, 0.64 mmol) was added to a solution of **35** (13 g, 15 mmol) in methanol (310 mL), and the mixture was stirred at ambient temperature for 5 h. After the addition of a saturated aqueous solution of NaHCO_3_ (35 mL) and water (200 mL), the mixture was extracted with dichloromethane (3× 150 mL). The combined organic layers were dried (Na_2_SO_4_), filtered, and the solvent was removed *in vacuo*. The residue was purified by flash column chromatography (petroleum ether/ethyl acetate = 4/1 → 2/1) to give **37** as a colorless solid (5.8 g, 7.0 mmol, 47%). m.p. = 67°C; TLC: *R*_*f*_ = 0.22 (petroleum ether/ethyl acetate = 4/1); ${\left[\alpha \right]}_{D}^{20}$ = +9.3 (1.5, dichloromethane); ^1^H NMR (400 MHz, DMSO‐*d*_*6*_): *δ* [ppm] = 0.62 (s, 9H, SiC(C*H*_3_)_3_), 0.85 (s, 9H, SiC(C*H*_3_)_3_), 2.81 – 2.89 (m, 1H, HOC*H*_2_CHOH), 2.97–3.05 (m, 1H, HOC*H*_2_CHOH), 3.47–3.55 (m, 1H, HOCH_2_C*H*OH), 3.78 (d, *J* = 6.8 Hz, 1H, 2‐H), 4.08 (d, *J* = 2.5 Hz, 1H, 4‐H), 4.15 (s, 1H, 3‐H), 4.30 (d, *J* = 5.0 Hz, 1H, HOCH_2_CHO*H*), 4.35 (t, *J* = 5.5 Hz, 1H, *H*OCH_2_CHOH), 5.07 (d, *J* = 2.6 Hz, 1H, 5‐H), 5.10 (s, 2H, OC*H*_2_Ph), 6.83–6.89 (m, 2H, 3′‐H_4‐(benzyloxy)phenyl_, 5′‐H_4‐(benzyloxy)phenyl_), 6.89–6.94 (m, 2H, 2'''‐H_diphenylsilyl_, 6'''‐H_diphenylsilyl_), 7.09–7.50 (m, 25H, H_arom._); ^13^C NMR (101 MHz, DMSO‐*d*_*6*_): *δ* [ppm] = 18.3 (1C, Si*C*(CH_3_)_3_), 18.6 (1C, Si*C*(CH_3_)_3_), 26.58 (3C, SiC(*C*H_3_)_3_), 26.61 (3C, SiC(*C*H_3_)_3_), 62.5 (1C, HO*C*H_2_CHOH), 69.1 (1C, O*C*H_2_Ph), 71.6 (1C, HOCH_2_ *C*HOH), 79.8 (1C, C‐4), 79.9 (1C, C‐3), 82.4 (1C, C‐5), 87.5 (1C, C‐2), 114.0 (2C, C‐3′_4‐(benzyloxy)phenyl_, C‐5′_4‐(benzyloxy)phenyl_), 127.3 (2C, C‐3'''_d__iphenylsilyl_, C‐5'''_d__iphenylsilyl_), 127.4 (2C, C‐2''_b__enzyl_, C‐6''_b__enzyl_), 127.5 (2C, C‐3'''_d__iphenylsilyl_, C‐5'''_d__iphenylsilyl_), 127.68 (1C, C‐4''_b__enzyl_), 127.71 (2C, C‐3'''_d__iphenylsilyl_, C‐5'''_d__iphenylsilyl_), 127.8 (2C, C‐3'''_d__iphenylsilyl_, C‐5'''_d__iphenylsilyl_), 128.4 (2C, C‐3''_b__enzyl_, C‐5''_b__enzyl_), 128.8 (2C, C‐2′_4‐(benzyloxy)phenyl_, C‐6′_4‐(benzyloxy)phenyl_), 128.9 (1C, C‐1′_4‐(benzyloxy)phenyl_), 129.6 (1C, C‐4'''_d__iphenylsilyl_), 129.8 (1C, C‐4'''_d__iphenylsilyl_), 129.93 (1C, C‐4'''_d__iphenylsilyl_), 129.95 (1C, C‐4'''_d__iphenylsilyl_), 131.2 (1C, C‐1'''d_iphenylsilyl_), 132.5 (2C, C‐1'''_d__iphenylsilyl_), 132.6 (1C, C‐1'''_d__iphenylsilyl_), 135.1 (2C, C‐2'''_d__iphenylsilyl_, C‐6'''_d__iphenylsilyl_), 135.3 (2C, C‐2'''_d__iphenylsilyl_, C‐6'''_d__iphenylsilyl_), 135.4 (2C, C‐2'''_d__iphenylsilyl_, C‐6'''_d__iphenylsilyl_), 135.5 (2C, C‐2'''_d__iphenylsilyl_, C‐6'''_d__iphenylsilyl_), 137.3 (1C, C‐1''_b__enzyl_), 157.6 (1C, C‐4′_4‐(benzyloxy)phenyl_); IR (neat): $\tilde{\nu }$ [cm^‐1^] = 3423, 3070, 2930, 2890, 2857, 1613, 1588, 1512, 1471, 1427, 1390, 1363, 1303, 1240, 1223, 1173, 1105, 1050, 1024, 940, 898, 861, 822, 800, 739, 698, 609, 502, 486; HRMS (*m*/*z*): [M+Na]^+^ calcd for C_51_H_58_NaO_6_Si_2_: 845.3664, found: 845.3624; HPLC (method 3): *t*_R_ = 33.0 min, purity 97.8%.

#### 4.1.25 Synthesis of (2*R*,3*S*,4*S*,5*R*)‐3,4‐bis[(*tert*‐butyldiphenylsilyl)oxy]‐5‐(4‐iodophenyl)tetrahydrofuran‐2‐carbaldehyde (**38**)

Water (23 mL) and sodium metaperiodate (2.0 g, 9.4 mmol) were added to a solution of **36** (3.9 g, 4.7 mmol) in THF (47 mL). After stirring the reaction mixture at ambient temperature overnight, water was added, and the mixture was extracted with dichloromethane (3×). The combined organic layers were dried (Na_2_SO_4_), filtered, and the solvent was removed *in vacuo*. The residue was purified by flash column chromatography (petroleum ether/ethyl acetate = 9/1, *R*_*f*_ = 0.82) to give **38** as a colorless solid (3.2 g, 3.9 mmol, 84%). m.p. = 105°C; ${\left[\alpha \right]}_{D}^{20}$ = ‐23.8 (5.0, dichloromethane); ^1^H NMR (500 MHz, CDCl_3_): *δ* [ppm] = 0.72 (s, 9H, SiC(C*H*_3_)_3_), 0.99 (s, 9H, SiC(C*H*_3_)_3_), 4.08 (dd, *J* = 2.4/1.3 Hz, 1H, 4‐H), 4.33 (s, 1H, 2‐H), 4.51–4.53 (m, 1H, 3‐H), 5.33 (d, *J* = 2.2 Hz, 1H, 5‐H), 6.85–6.89 (m, 2H, 2''‐H_diphenylsilyl_, 6''‐H_diphenylsilyl_), 6.99–7.03 (m, 2H, 2′‐H_4‐iodophenyl_, 6′‐H_4‐iodophenyl_), 7.09–7.14 (m, 2H, 3''‐H_diphenylsilyl_, 5''‐H_diphenylsilyl_), 7.16–7.22 (m, 4H, 2''‐H_diphenylsilyl_, 3''‐H_diphenylsilyl_, 5''‐H_diphenylsilyl_, 6''‐H_diphenylsilyl_), 7.24–7.28 (m, 2H, 3''‐H_diphenylsilyl_, 5''‐H_diphenylsilyl_), 7.28–7.33 (m, 1H, 4''‐H_diphenylsilyl_), 7.33–7.38 (m, 3H, 3''‐H_diphenylsilyl_, 4''‐H_diphenylsilyl_, 5''‐H_diphenylsilyl_), 7.38–7.48 (m, 4H, 2''‐H_diphenylsilyl_, 4''‐H_diphenylsilyl_ (2H), 6''‐H_diphenylsilyl_), 7.48–7.54 (m, 4H, 3′‐H_4‐iodophenyl_, 5′‐H_4‐iodophenyl_, 2''‐H_diphenylsilyl_, 6''‐H_diphenylsilyl_), 9.72–9.74 (m, 1H, C*H*═O); ^13^C NMR (126 MHz, CDCl_3_): *δ* [ppm] = 18.8 (1C, Si*C*(CH_3_)_3_), 19.2 (1C, Si*C*(CH_3_)_3_), 26.91 (3C, SiC(*C*H_3_)_3_), 26.92 (3C, SiC(*C*H_3_)_3_), 78.2 (1C, C‐4), 82.6 (1C, C‐3), 84.7 (1C, C‐5), 89.8 (1C, C‐2), 93.2 (1C, C‐4′_4‐iodophenyl_), 127.5 (2C, C‐3''_d__iphenylsilyl_, C‐5''_d__iphenylsilyl_), 127.7 (2C, C‐3''_d__iphenylsilyl_, C‐5''_d__iphenylsilyl_), 127.95 (2C, C‐3''_d__iphenylsilyl_, C‐5''_d__iphenylsilyl_), 128.01 (2C, C‐3''_d__iphenylsilyl_, C‐5''_d__iphenylsilyl_), 129.5 (2C, C‐2′_4‐iodophenyl_, C‐6′_4‐iodophenyl_), 129.7 (1C, C‐4''_d__iphenylsilyl_), 130.0 (1C, C‐4''_d__iphenylsilyl_), 130.1 (1C, C‐4''_d__iphenylsilyl_), 130.2 (1C, C‐4''_d__iphenylsilyl_), 131.8 (1C, C‐1''_d__iphenylsilyl_), 132.5 (1C, C‐1''_d__iphenylsilyl_), 132.6 (1C, C‐1''_d__iphenylsilyl_), 132.8 (1C, C‐1''_d__iphenylsilyl_), 135.7 (2C, C‐2''_d__iphenylsilyl_, C‐6''_d__iphenylsilyl_), 135.8 (2C, C‐2''_d__iphenylsilyl_, C‐6''_d__iphenylsilyl_), 135.95 (2C, C‐2''_d__iphenylsilyl_, C‐6''_d__iphenylsilyl_), 136.04 (2C, C‐2''_d__iphenylsilyl_, C‐6''_d__iphenylsilyl_), 136.2 (1C, C‐1′_4‐iodophenyl_), 137.1 (2C, C‐3′_4‐iodophenyl_, C‐5′_4‐iodophenyl_), 201.7 (1C, *C*H═O); IR (neat): $\tilde{\nu }$ [cm^–1^] = 3071, 3046, 2930, 2893, 2857, 1731, 1589, 1485, 1471, 1427, 1392, 1363, 1242, 1188, 1104, 1042, 1005, 960, 930, 896, 817, 794, 739, 698, 609, 501, 485; HRMS (*m*/*z*): [M+Na]^+^ calcd for C_43_H_47_INaO_4_Si_2_: 833.1950, found: 833.1965.

#### 4.1.26 Synthesis of (2*R*,3*S*,4*S*,5*R*)‐5‐[4‐(benzyloxy)phenyl]‐3,4‐bis[(*tert*‐butyldiphenylsilyl)oxy]tetrahydrofuran‐2‐carbaldehyde (**39**)

Water (12 mL) and sodium metaperiodate (0.99 g, 4.6 mmol) were added to a solution of **37** (1.9 g, 2.2 mmol) in THF (24 mL). After stirring the reaction mixture at ambient temperature overnight, water was added, and the mixture was extracted with dichloromethane (3×). The combined organic layers were dried (Na_2_SO_4_), filtered, and the solvent was removed *in vacuo*. The residue was purified by flash column chromatography (petroleum ether/ethyl acetate = 9/1, *R*_*f*_ = 0.75) to give **39** as a colorless solid (1.7 g, 2.1 mmol, 94%). m.p. = 122°C; ${\left[\alpha \right]}_{D}^{20}$ = ‐25.3 (4.7, dichloromethane); ^1^H NMR (500 MHz, CDCl_3_): *δ* [ppm] = 0.72 (s, 9H, SiC(C*H*_3_)_3_), 0.97 (s, 9H, SiC(C*H*_3_)_3_), 4.06 (dd, *J* = 2.4/1.2 Hz, 1H, 4‐H), 4.26 (s, 1H, 2‐H), 4.35–4.37 (m, 1H, 3‐H), 5.09 (s, 2H, OC*H*_2_Ph), 5.40 (d, *J* = 2.2 Hz, 1H, 5‐H), 6.83–6.91 (m, 4H, 3′‐H_4‐(benzyloxy)phenyl_, 5′‐H_4‐(benzyloxy)phenyl_, 2'''‐H_diphenylsilyl_, 6'''‐H_diphenylsilyl_), 7.08–7.14 (m, 4H, H_arom._), 7.22–7.50 (m, 21H, H_arom._), 9.74– 9.75 (m, 1H, C*H* = O); ^13^C NMR (126 MHz, CDCl_3_): *δ* [ppm] = 18.8 (1C, Si*C*(CH_3_)_3_), 19.2 (1C, Si*C*(CH_3_)_3_), 26.9 (3C, SiC(*C*H_3_)_3_), 27.0 (3C, SiC(*C*H_3_)_3_), 70.1 (1C, O*C*H_2_Ph), 78.3 (1C, C‐4), 82.7 (1C, C‐3), 84.9 (1C, C‐5), 89.5 (1C, C‐2), 114.6 (2C, C‐3′_4‐(benzyloxy)phenyl_, C‐5′_4‐(benzyloxy)phenyl_), 127.4 (2C, C‐3'''_d__iphenylsilyl_, C‐5'''_d__iphenylsilyl_), 127.5 (2C, C‐2''_b__enzyl_, C‐6''b_enzyl_), 127.7 (2C, C‐3'''_d__iphenylsilyl_, C‐5'''_d__iphenylsilyl_), 127.87 (2C, C‐3'''_d__iphenylsilyl_, C‐5'''_d__iphenylsilyl_), 127.94 (2C, C‐3'''_d__iphenylsilyl_, C‐5'''_d__iphenylsilyl_), 128.0 (1C, C‐4''_b__enzyl_), 128.73 (2C, C‐3''_b__enzyl_, C‐5''_b__enzyl_), 128.75 (1C, C‐1′_4‐(benzyloxy)phenyl_), 129.1 (2C, C‐2′_4‐(benzyloxy)phenyl_, C‐6′_4‐(benzyloxy)phenyl_), 129.6 (1C, C‐4'''_d__iphenylsilyl_), 129.9 (1C, C‐4'''_d__iphenylsilyl_), 130.0 (1C, C‐4'''_d__iphenylsilyl_), 130.1 (1C, C‐4'''_d__iphenylsilyl_), 131.9 (1C, C‐1'''_d__iphenylsilyl_), 132.7 (1C, C‐1'''_d__iphenylsilyl_), 132.86 (1C, C‐1'''_d__iphenylsilyl_), 132.88 (1C, C‐1'''_d__iphenylsilyl_), 135.76 (2C, C‐2'''_d__iphenylsilyl_, C‐6'''_d__iphenylsilyl_), 135.77 (2C, C‐2'''_d__iphenylsilyl_, C‐6'''_d__iphenylsilyl_), 136.0 (2C, C‐2'''_d__iphenylsilyl_, C‐6'''_d__iphenylsilyl_), 136.1 (2C, C‐2'''_d__iphenylsilyl_, C‐6'''_d__iphenylsilyl_), 137.3 (1C, C‐1''_b__enzyl_), 158.6 (1C, C‐4′_4‐(benzyloxy)phenyl_), 202.1 (1C, *C*H═O); IR (neat): $\tilde{\nu }$ [cm^‐1^] = 3071, 3048, 2930, 2889, 2857, 1971, 1863, 1731, 1614, 1588, 1512, 1487, 1471, 1427, 1391, 1363, 1302, 1240, 1223, 1189, 1173, 1104, 1081, 1040, 1026, 998, 958, 938, 896, 864, 821, 804, 738, 697, 610, 502, 485; HRMS (*m*/*z*): [M+Na]^+^ calcd for C_50_H_54_NaO_5_Si_2_: 813.3402, found: 813.3387.

#### 4.1.27 Synthesis of methyl (2*R*,3*S*,4*S*,5*R*)‐3,4‐bis[(*tert*‐butyldiphenylsilyl)oxy]‐5‐(4‐iodophenyl)tetrahydrofuran‐2‐carboxylate (**40**)

*N*‐Methylmorpholine *N*‐oxide monohydrate (4.1 g, 30 mmol) and tetrapropylammonium perruthenate (110 mg, 0.30 mmol) were successively added to a solution of **38** (2.4 g, 3.0 mmol) in dichloromethane (30 mL). After stirring the mixture at ambient temperature overnight, it was filtered through Celite and the solvent was removed *in vacuo*. The residue was dissolved in acetone (60 mL), and K_2_CO_3_ (2.1 g, 15 mmol) and iodomethane (0.95 mL, 2.2 g, 15 mmol) were successively added. After stirring the mixture at ambient temperature overnight, it was filtered through Celite, and the solvent was removed *in vacuo*. The residue was purified by flash column chromatography (petroleum ether/ethyl acetate = 15/1, *R*_*f*_ = 0.18) to give **40** as a colorless solid (1.5 g, 1.8 mmol, 60%). m.p. = 164°C; ${\left[\alpha \right]}_{D}^{20}$ = −87.6 (2.5, dichloromethane); ^1^H NMR (500 MHz, CDCl_3_): *δ* [ppm] = 0.70 (s, 9H, SiC(C*H*_3_)_3_), 0.98 (s, 9H, SiC(C*H*_3_)_3_), 3.58 (s, 3H, CO_2_C*H*_3_), 4.10 (dd, *J* = 2.8/1.1 Hz, 1H, 4‐H), 4.48 (s, 1H, 2‐H), 4.69–4.71 (m, 1H, 3‐H), 5.29 (d, *J* = 2.7 Hz, 1H, 5‐H), 6.92–6.95 (m, 2H, 2''‐H_diphenylsilyl_, 6''‐H_diphenylsilyl_), 7.10–7.14 (m, 2H, 3''‐H_diphenylsilyl_, 5''‐H_diphenylsilyl_), 7.14–7.23 (m, 6H, 2′‐H_4‐iodophenyl_, 6′‐H_4‐iodophenyl_, 2''‐H_diphenylsilyl_, 3''‐H_diphenylsilyl_, 5''‐H_diphenylsilyl_, 6''‐H_diphenylsilyl_), 7.25–7.37 (m, 6H, 3''‐H_diphenylsilyl_ (2H), 4''‐H_diphenylsilyl_ (2H), 5''‐H_diphenylsilyl_ (2H)), 7.39–7.46 (m, 4H, 2''‐H_diphenylsilyl_, 4''‐H_diphenylsilyl_ (2H), 6''‐H_diphenylsilyl_), 7.47–7.51 (m, 2H, 3′‐H_4‐iodophenyl_, 5′‐H_4‐iodophenyl_), 7.52–7.56 (m, 2H, 2''‐H_diphenylsilyl_, 6''‐H_diphenylsilyl_); ^13^C NMR (126 MHz, CDCl_3_): *δ* [ppm] = 18.9 (1C, Si*C*(CH_3_)_3_), 19.4 (1C, Si*C*(CH_3_)_3_), 26.8 (3C, SiC(*C*H_3_)_3_), 26.9 (3C, SiC(*C*H_3_)_3_), 52.0 (1C, CO_2_ *C*H_3_), 78.6 (1C, C‐4), 82.8 (1C, C‐3), 84.0 (1C, C‐2), 85.3 (1C, C‐5), 93.2 (1C, C‐4′_4‐iodophenyl_), 127.5 (2C, C‐3''_d__iphenylsilyl_, C‐5''_d__iphenylsilyl_), 127.6 (2C, C‐3''_d__iphenylsilyl_, C‐5''_d__iphenylsilyl_), 127.8 (2C, C‐3''_d__iphenylsilyl_, C‐5''_d__iphenylsilyl_), 127.9 (2C, C‐3''_d__iphenylsilyl_, C‐5''_d__iphenylsilyl_), 129.6 (1C, C‐4''_d__iphenylsilyl_), 129.8 (1C, C‐4''_d__iphenylsilyl_), 129.98 (1C, C‐4''_d__iphenylsilyl_), 130.02 (1C, C‐4''_d__iphenylsilyl_), 130.3 (2C, C‐2′_4‐iodophenyl_, C‐6′_4‐iodophenyl_), 132.1 (1C, C‐1''_d__iphenylsilyl_), 132.9 (3C, C‐1''_d__iphenylsilyl_), 135.89 (2C, C‐2''_d__iphenylsilyl_, C‐6''_d__iphenylsilyl_), 135.92 (2C, C‐2''_d__iphenylsilyl_, C‐6''_d__iphenylsilyl_), 135.95 (2C, C‐2''_d__iphenylsilyl_, C‐6''_d__iphenylsilyl_), 136.00 (2C, C‐2''_d__iphenylsilyl_, C‐6''_d__iphenylsilyl_), 136.4 (1C, C‐1′_4‐iodophenyl_), 136.9 (2C, C‐3′_4‐iodophenyl_, C‐5′_4‐iodophenyl_), 170.5 (1C, *C*O_2_CH_3_); IR (neat): $\tilde{\nu }$ [cm^‐1^] = 3072, 3047, 2954, 2930, 2891, 2855, 1759, 1734, 1589, 1484, 1470, 1439, 1426, 1403, 1390, 1376, 1362, 1334, 1302, 1270, 1212, 1180, 1103, 1043, 1005, 974, 956, 933, 923, 904, 890, 854, 843, 824, 806, 773, 739, 698, 665, 610, 513, 502, 482, 437; HRMS (*m*/*z*): [M+Na]^+^ calcd for C_44_H_49_INaO_5_Si_2_: 863.2055, found: 863.2050.

#### 4.1.28 Synthesis of methyl (2*R*,3*S*,4*S*,5*R*)‐5‐[4‐(benzyloxy)phenyl]‐3,4‐bis[(*tert*‐butyldiphenylsilyl)oxy]tetrahydrofuran‐2‐carboxylate (**41**)

Method 1: *N*‐Methylmorpholine *N*‐oxide monohydrate (2.7 g, 20 mmol) and tetrapropylammonium perruthenate (70 mg, 0.20 mmol) were successively added to a solution of **39** (1.6 g, 2.0 mmol) in dichloromethane (20 mL). After stirring the mixture at ambient temperature overnight, it was filtered through Celite and the solvent was removed *in vacuo*. The residue was dissolved in acetone (40 mL), and K_2_CO_3_ (1.4 g, 10 mmol) and iodomethane (0.62 mL, 1.4 g, 10 mmol) were added. After stirring the mixture at ambient temperature overnight, it was filtered through Celite, and the solvent was removed *in vacuo*. The residue was purified by flash column chromatography (petroleum ether/ethyl acetate = 12/1, *R*_*f*_ = 0.17) to give **41** as a colorless, sticky solid (1.2 g, 1.4 mmol, 72%).

Method 2: Orthoperiodic acid (5.0 g, 22 mmol) was added to a solution of **37** (4.0 g, 4.9 mmol) in acetonitrile (20 mL) at 0°C. After stirring the reaction mixture for 1 h, pyridinium chlorochromate (21 mg, 0.097 mmol) was added at 0°C. After stirring the resulting mixture for 1 h at ambient temperature, water (50 mL) was added, and the mixture was extracted with ethyl acetate (3×). The combined organic layers were dried (Na_2_SO_4_), filtered, and the solvent was removed *in vacuo*. The residue was dissolved in acetone (40 mL), and K_2_CO_3_ (3.4 g, 24 mmol) and iodomethane (1.5 mL, 3.5 g, 24 mmol) were successively added. After stirring the mixture at ambient temperature overnight, it was filtered through Celite and the solvent was removed *in vacuo*. The residue was purified by flash column chromatography (petroleum ether/ethyl acetate = 8/1, *R*_*f*_ = 0.50) to give **41** as a colorless, sticky solid (3.4 g, 4.2 mmol, 86%).

${\left[\alpha \right]}_{D}^{20}$ = ‐58.0 (4.5, dichloromethane); ^1^H NMR (500 MHz, CDCl_3_): *δ* [ppm] = 0.70 (s, 9H, SiC(C*H*_3_)_3_), 0.97 (s, 9H, SiC(C*H*_3_)_3_), 3.56 (s, 3H, CO_2_C*H*_3_), 4.08 (dd, *J* = 2.8/1.1 Hz, 1H, 4‐H), 4.43 (s, 1H, 2‐H), 4.56–4.58 (m, 1H, 3‐H), 5.04–5.10 (m, 2H, OC*H*_2_Ph), 5.36 (d, *J* = 2.6 Hz, 1H, 5‐H), 6.85–6.89 (m, 2H, 3′‐H_4‐(benzyloxy)phenyl_, 5′‐H_4‐(benzyloxy)phenyl_), 6.92–6.95 (m, 2H, 2'''‐H_diphenylsilyl_, 6'''‐H_diphenylsilyl_), 7.09–7.14 (m, 4H, H_arom._), 7.24–7.45 (m, 19H, H_arom._), 7.50–7.53 (m, 2H, 2'''‐H_diphenylsilyl_, 6'''‐H_diphenylsilyl_); ^13^C NMR (126 MHz, CDCl_3_): *δ* [ppm] = 18.9 (1C, Si*C*(CH_3_)_3_), 19.2 (1C, Si*C*(CH_3_)_3_), 26.85 (3C, SiC(*C*H_3_)_3_), 26.89 (3C, SiC(*C*H_3_)_3_), 51.8 (1C, CO_2_ *C*H_3_), 70.1 (1C, O*C*H_2_Ph), 78.7 (1C, C‐4), 82.7 (1C, C‐3), 83.7 (1C, C‐2), 85.5 (1C, C‐5), 114.5 (2C, C‐3′_4‐(benzyloxy)phenyl_, C‐5′_4‐(benzyloxy)phenyl_), 127.3 (2C, C‐3'''_d__iphenylsilyl_, C‐5'''_d__iphenylsilyl_), 127.5 (2C, C‐2''_b__enzyl_, C‐6''_b__enzyl_), 127.6 (2C, C‐3'''_d__iphenylsilyl_, C‐5'''_d__iphenylsilyl_), 127.75 (2C, C‐3'''_d__iphenylsilyl_, C‐5'''_d__iphenylsilyl_), 127.82 (2C, C‐3'''_d__iphenylsilyl_, C‐5'''_d__iphenylsilyl_), 128.0 (1C, C‐4''_b__enzyl_), 128.7 (2C, C‐3''_b__enzyl_, C‐5''_b__enzyl_), 129.0 (1C, C‐1′_4‐(benzyloxy)phenyl_), 129.5 (1C, C‐4'''_d__iphenylsilyl_), 129.7 (1C, C‐4'''_d__iphenylsilyl_), 129.8 (2C, C‐2′_4‐(benzyloxy)phenyl_, C‐6′_4‐(benzyloxy)phenyl_), 129.89 (1C, C‐4'''_d__iphenylsilyl_), 129.91 (1C, C‐4'''_d__iphenylsilyl_), 132.2 (1C, C‐1'''_d__iphenylsilyl_), 133.0 (1C, C‐1'''_d__iphenylsilyl_), 133.12 (1C, C‐1'''_d__iphenylsilyl_), 133.14 (1C, C‐1'''_d__iphenylsilyl_), 135.89 (2C, C‐2'''_d__iphenylsilyl_, C‐6'''_d__iphenylsilyl_), 135.91 (2C, C‐2'''_d__iphenylsilyl_, C‐6'''_d__iphenylsilyl_), 136.0 (2C, C2'''_d__iphenylsilyl_, C‐6'''_d__iphenylsilyl_), 136.1 (2C, C‐2'''_d__iphenylsilyl_, C‐6'''_d__iphenylsilyl_), 137.4 (1C, C‐1''_b__enzyl_), 158.5 (1C, C‐4′_4‐(benzyloxy)phenyl_), 170.7 (1C, *C*O_2_CH_3_); IR (neat): $\tilde{\nu }$ [cm^–1^] = 3071, 3047, 2931, 2891, 2857, 1759, 1733, 1613, 1588, 1512, 1471, 1427, 1391, 1372, 1330, 1304, 1239, 1214, 1174, 1097, 1044, 1008, 956, 934, 891, 822, 807, 738, 698, 610, 501, 486; HRMS (*m*/*z*): [M+Na]^+^ calcd for C_51_H_56_NaO_6_Si_2_: 843.3508, found: 843.3518; HPLC (method 3): *t*_R_ = 34.3 min, purity 98.6%.

#### 4.1.29 Synthesis of methyl (2*R*,3*R*,4*R*,5*R*)‐5‐[4‐(benzyloxy)phenyl]‐3,4‐dihydroxytetrahydrofuran‐2‐carboxylate (**42**)

Tetrabutylammonium fluoride trihydrate (7.6 g, 24 mmol) was added to a solution of **41** (5.0 g, 6.0 mmol) in dry THF (60 mL). After stirring the reaction mixture at 60°C overnight, a saturated aqueous solution of NH_4_Cl (40 mL) was added, and the mixture was extracted with dichloromethane (3×). The combined organic layers were dried (Na_2_SO_4_), filtered, and the solvent was removed *in vacuo*. The residue was dissolved in methanol (60 mL), and *p*‐toluenesulfonic acid monohydrate (460 mg, 2.4 mmol) was added. After stirring the mixture at 60°C overnight, it was cooled to ambient temperature and triethylamine (1.0 mL) was added. The volatiles were removed *in vacuo*, and the residue was purified by flash column chromatography (dichloromethane/methanol = 19/1, *R*_*f*_ = 0.39) to give **42** as a colorless solid (1.7 g, 5.0 mmol, 82%). m.p. = 153°C; ${\left[\alpha \right]}_{D}^{20}$ = ‐52.4 (1.4, methanol); ^1^H NMR (400 MHz, DMSO‐*d*_*6*_): *δ* [ppm] = 3.67 (s, 3H, CO_2_C*H*_3_), 3.77–3.82 (m, 1H, 4‐H), 4.30–4.33 (m, 1H, 2‐H), 4.33–4.36 (m, 1H, 3‐H), 4.75 (d, *J* = 4.0 Hz, 1H, 4‐O*H*), 5.05 (d, *J* = 3.0 Hz, 1H, 5‐H), 5.09 (s, 2H, OC*H*_2_Ph), 5.73 (d, *J* = 3.9 Hz, 1H, 3‐O*H*), 6.90–6.98 (m, 2H, 3′‐H_4‐(benzyloxy)phenyl_, 5′‐H_4‐(benzyloxy)phenyl_), 7.29–7.48 (m, 7H, 2′‐H_4‐(benzyloxy)phenyl_, 6′‐H_4‐(benzyloxy)phenyl_, 2''‐H_benzyl_, 3''‐H_benzyl_, 4''‐H_benzyl_, 5''‐H_benzyl_, 6''‐H_benzyl_); ^13^C NMR (101 MHz, DMSO‐*d*_*6*_): *δ* [ppm] = 51.5 (1C, CO_2_ *C*H_3_), 69.1 (1C, O*C*H_2_Ph), 76.5 (1C, C‐4), 81.1 (1C, C‐3), 83.0 (1C, C‐2), 83.9 (1C, C‐5), 113.7 (2C, C‐3′_4‐(benzyloxy)phenyl_, C‐5′_4‐(benzyloxy)phenyl_), 127.6 (2C, C‐2''_b__enzyl_, C‐6''_b__enzyl_), 127.8 (1C, C‐4''_b__enzyl_), 128.5 (2C, C‐3''_b__enzyl_, C‐5''_b__enzyl_), 129.3 (2C, C‐2′_4‐(benzyloxy)phenyl_, C‐6′_4‐(benzyloxy)phenyl_), 129.8 (1C, C‐1′_4‐(benzyloxy)phenyl_), 137.3 (1C, C‐1''_b__enzyl_), 157.6 (1C, C‐4′_4‐(benzyloxy)phenyl_), 171.2 (1C, *C*O_2_CH_3_); IR (neat): $\tilde{\nu }$ [cm^‐1^] = 3344, 2959, 2923, 2896, 2855, 1747, 1610, 1584, 1512, 1454, 1433, 1382, 1346, 1299, 1230, 1172, 1114, 1078, 1054, 1033, 980, 951, 939, 927, 905, 858, 832, 791, 772, 762, 742, 724, 699, 634, 567, 547, 500, 426; HRMS (*m*/*z*): [M+Na]^+^ calcd for C_19_H_20_NaO_6_: 367.1152, found: 367.1123; HPLC (method 1): *t*_R_ = 19.2 min, purity 98.0%;

X‐ray crystal structure analysis: structural data can be found on https://www.ccdc.cam.ac.uk under the deposition number CCDC 2516138.

#### 4.1.30 Synthesis of methyl (2*R*,3*R*,4*R*,5*S*)‐5‐[4‐(benzyloxy)phenyl]‐3,4‐dihydroxytetrahydrofuran‐2‐carboxylate (**43**)

Under N_2_ atmosphere, erbium(III) trifluoromethanesulfonate (54 mg, 0.087 mmol) was added to a solution of **42** (600 mg, 1.7 mmol) in acetonitrile (5 mL). After heating the reaction mixture in a sealed pressure tube to 120°C overnight, the solvent was removed *in vacuo*, and the residue was purified by flash column chromatography (dichloromethane/methanol = 40/1) to give **43** as a yellowish solid (120 mg, 0.34 mmol, 20%). m.p. = 89°C; TLC: *R*_*f*_ = 0.38 (dichloromethane/methanol = 19/1); ${\left[\alpha \right]}_{D}^{20}$ = ‐26.0 (1.9, methanol); ^1^H NMR (600 MHz, DMSO‐*d*_*6*_): *δ* [ppm] = 3.68 (s, 3H, CO_2_C*H*_3_), 3.73–3.77 (m, 1H, 4‐H), 4.14–4.18 (m, 1H, 3‐H), 4.43 (d, *J* = 4.1 Hz, 1H, 2‐H), 4.66 (d, *J* = 6.3 Hz, 1H, 5‐H), 5.10 (s, 2H, OC*H*_2_Ph), 5.44 (d, *J* = 5.2 Hz, 1H, 4‐O*H*), 5.67 (d, *J* = 5.1 Hz, 1H, 3‐O*H*), 6.96–7.01 (m, 2H, 3′‐H_4‐(benzyloxy)phenyl_, 5′‐H_4‐(benzyloxy)phenyl_), 7.28–7.34 (m, 3H, 2′‐H_4‐(benzyloxy)phenyl_, 6′‐H_4‐(benzyloxy)phenyl_, 4''‐H_benzyl_), 7.37–7.41 (m, 2H, 3''‐H_benzyl_, 5''‐H_benzyl_), 7.43–7.46 (m, 2H, 2''‐H_benzyl_, 6''‐H_benzyl_); ^13^C NMR (151 MHz, DMSO‐*d*_*6*_): *δ* [ppm] = 51.7 (1C, CO_2_ *C*H_3_), 69.2 (1C, O*C*H_2_Ph), 80.7 (1C, C‐3), 82.0 (1C, C‐2), 83.1 (1C, C‐4), 85.4 (1C, C‐5), 114.4 (2C, C‐3′_4‐(benzyloxy)phenyl_, C‐5′_4‐(benzyloxy)phenyl_), 127.6 (2C, C‐2''_b__enzyl_, C‐6''_b__enzyl_), 127.8 (3C, C‐2′_4‐(benzyloxy)phenyl_, C‐6′_4‐(benzyloxy)phenyl_, C‐4''_b__enzyl_), 128.4 (2C, C‐3''_b__enzyl_, C‐5''_b__enzyl_), 132.5 (1C, C‐1′_4‐(benzyloxy)phenyl_), 137.1 (1C, C‐1''_b__enzyl_), 157.8 (1C, C‐4′_4‐(benzyloxy)phenyl_), 171.8 (1C, *C*O_2_CH_3_); IR (neat): $\tilde{\nu }$ [cm^–1^] = 3409, 3034, 2952, 1733, 1612, 1585, 1512, 1454, 1438, 1381, 1299, 1237, 1175, 1100, 1078, 1010, 962, 918, 870, 829, 733, 697, 639, 546, 510, 475; HRMS (*m*/*z*): [M+Na]^+^ calcd for C_19_H_20_NaO_6_: 367.1152, found: 367.1146; HPLC (method 1): *t*_R_ = 18.9 min, purity 95.1%.

#### 4.1.31 Synthesis of methyl (2*R*,3*R*,4*R*,5*S*)‐3,4‐dihydroxy‐5‐(4‐hydroxyphenyl)tetrahydrofuran‐2‐carboxylate (**44**)

Under N_2_ atmosphere, palladium on activated charcoal (10% Pd, 62 mg) and triethylsilane (0.46 mL, 330 mg, 2.9 mmol) were added to a solution of **43** (100 mg, 0.29 mmol) in methanol (4 mL) and the reaction mixture was stirred for 1 h at ambient temperature. Additional triethylsilane (0.25 mL, 180 mg, 1.5 mmol) was added, and the mixture was stirred for 30 min before further triethylsilane (0.25 mL, 180 mg, 0.93 mmol) was added. After stirring the mixture for 30 min at ambient temperature, additional triethylsilane (0.25 mL, 180 mg, 1.5 mmol) was added, and the mixture was stirred for 30 min. After the addition of further triethylsilane (0.25 mL, 180 mg, 1.5 mmol), the mixture was stirred for an additional 30 min before it was filtered through Celite, and the solvent was removed *in vacuo*. The residue was purified by flash column chromatography (dichloromethane/methanol = 9/1) to give **44** as a yellowish solid (50 mg, 0.20 mmol, 68%). m.p. = 107°C; TLC: *R*_*f*_ = 0.21 (dichloromethane/methanol = 19/1); ${\left[\alpha \right]}_{D}^{20}$ = ‐50.5 (1.4, methanol); ^1^H NMR (400 MHz, DMSO‐*d*_*6*_): *δ* [ppm] = 3.68 (s, 3H, CO_2_C*H*_3_), 3.71–3.76 (m, 1H, 4‐H), 4.12–4.16 (m, 1H, 3‐H), 4.39 (d, *J* = 4.2 Hz, 1H, 2‐H), 4.59 (d, *J* = 6.5 Hz, 1H, 5‐H), 5.40 (d, *J* = 5.2 Hz, 1H, 4‐O*H*), 5.66 (d, *J* = 5.2 Hz, 1H, 3‐O*H*), 6.96–7.01 (m, 2H, 3′‐H_4‐hydroxyphenyl_, 5′‐H_4‐hydroxyphenyl_), 7.15–7.20 (m, 2H, 2′‐H_4‐hydroxyphenyl_, 6′‐H_4‐hydroxyphenyl_), 9.35 (s, 1H, ArOH); ^13^C NMR (101 MHz, DMSO‐*d*_*6*_): *δ* [ppm] = 51.7 (1 C, CO_2_ *C*H_3_), 80.7 (1C, C‐3), 81.9 (1C, C‐2), 83.1 (1C, C‐4), 85.5 (1C, C‐5), 114.8 (2C, C‐3′_4‐hydroxyphenyl_, C‐5′_4‐hydroxyphenyl_), 127.9 (2C, C‐2′_4‐hydroxyphenyl_, C‐6′_4‐hydroxyphenyl_), 130.4 (1C, C‐1′_4‐hydroxyphenyl_), 156.9 (1C, C‐4′_4‐hydroxyphenyl_), 171.9 (1C, *C*O_2_CH_3_); IR (neat): $\tilde{\nu }$ [cm^–^^1^] = 3381, 3265, 3164, 3057, 2952, 2926, 2893, 2854, 1740, 1617, 1600, 1515, 1436, 1361, 1334, 1275, 1262, 1237, 1213, 1183, 1168, 1105, 1092, 1043, 1004, 972, 946, 866, 824, 813, 800, 758, 730, 709, 630, 594, 529, 489, 432, 418; HRMS (*m*/*z*): [M+Na]^+^ calcd for C_12_H_14_NaO_6_: 277.0683, found: 277.0689; HPLC (method 1): *t*_R_ = 7.5 min, purity 99.2%.

#### 4.1.32 Synthesis of methyl (2*R*,3*R*,4*R*,5*S*)‐3,4‐dihydroxy‐5‐(4‐{[(trifluoromethyl)sulfonyl]oxy}phenyl)tetrahydrofuran‐2‐carboxylate (**45**)

Under N_2_ atmosphere, triethylamine (0.11 mL, 79 mg, 0.78 mmol) and bis(trifluoromethanesulfonyl)aniline (150 mg, 0.43 mmol) were added to a solution of **44** (99 mg, 0.39 mmol) in dichloromethane (3.9 mL) at 0°C. After stirring the reaction mixture for 15 min at 0°C, stirring was continued for 3 h at ambient temperature. Then, water (10 mL) was added, and the aqueous layer was extracted with dichloromethane (3× 25 mL). The combined organic layers were dried (Na_2_SO_4_), filtered, and the solvent was removed *in vacuo*. The residue was purified by flash column chromatography (dichloromethane/methanol = 29/1) to give **45** as a colorless solid (100 mg, 0.27 mmol, 69%). m.p. = 91°C; TLC: *R*_*f*_ = 0.26 (dichloromethane/methanol = 19/1); ${\left[\alpha \right]}_{D}^{20}$ = ‐43.9 (1.1, methanol); ^1^H NMR (400 MHz, DMSO‐*d*_*6*_): *δ* [ppm] = 3.69 (s, 3H, CO_2_C*H*_3_), 3.74–3.80 (m, 1H, 4‐H), 4.17–4.22 (m, 1H, 3‐H), 4.53 (d, *J* = 3.6 Hz, 1H, 2‐H), 4.82 (d, *J* = 5.6 Hz, 1H, 5‐H), 5.63 (d, *J* = 4.8 Hz, 1H, 4‐O*H*), 5.72 (d, *J* = 4.8 Hz, 1H, 3‐O*H*), 7.45–7.51 (m, 2H, 3′‐H_phenyl_, 5′‐H_phenyl_), 7.54–7.59 (m, 2H, 2′‐H_phenyl_, 6′‐H_phenyl_); ^13^C NMR (101 MHz, DMSO‐*d*_*6*_): *δ* [ppm] = 51.8 (1C, CO_2_ *C*H_3_), 80.6 (1C, C‐3), 82.5 (1C, C‐2), 83.1 (1C, C‐4), 85.1 (1C, C‐5), 118.3 (q, *J* = 321 Hz, 1C, *C*F_3_), 121.2 (2C, C‐3′_phenyl_, C‐5′_phenyl_), 128.4 (2C, C‐2′_phenyl_, C‐6′_phenyl_), 141.5 (1C, C‐1′_phenyl_), 148.4 (1C, C‐4′_phenyl_), 171.4 (1C, *C*O_2_CH_3_); IR (neat): $\tilde{\nu }$ [cm^–1^] = 3550, 2961, 2929, 1740, 1601, 1504, 1423, 1345, 1249, 1203, 1135, 1093, 1055, 1042, 1018, 997, 979, 957, 889, 834, 777, 764, 747, 724, 705, 633, 602, 573, 534, 510, 471, 426; HRMS (*m*/*z*): [M+Na]^+^ calcd for C_13_H_13_F_3_NaO_8_S: 409.0175, found: 409.0182; HPLC (method 1): *t*_R_ = 19.1 min, purity 96.4%.

### 4.2 Computational Methods

#### 4.2.1 Protein Preparation

The protein structure of *E. coli* LpxC cocrystallized with LPC‐053 was retrieved from the Protein Data Bank (PDB ID 3PS3) [75]. The following steps were executed using the Schrödinger software suite; the respective tools were accessed via the graphical user interface Maestro [76]. Structure preparation was carried out using the Protein Preparation Wizard [77, 78]. In the first preprocessing step, bond orders were assigned, missing hydrogens were added, zero‐order bonds to metals were created, and the protein's termini were capped. Meanwhile, the ligand protonation states at pH 7.0 ± 2.0 were generated. All water and buffer molecules were deleted except for the conserved water molecules W1053 and W1058. Subsequently, the hydrogen bond network was optimized at pH 7.0. In the final step, the structure underwent a restrained minimization procedure while applying the OPLS3e force field [79].

#### 4.2.2 Ligand Preparation

Schrödinger's LigPrep [80] was used to prepare all ligands for molecular docking. The OPLS3e force field [79] was chosen for initial 3D structure generation. Possible protonation states at pH 7.0 ± 2.0 were generated by Epik [81, 82, 83]. Meanwhile, metal binding states were added, and specified chiralities were retained. Schrödinger's ConGen [84] was used afterward to generate 64 conformers per prepared ligand for docking input.

#### 4.2.3 Molecular Docking

Initially, a receptor grid was generated using the prepared complex of LpxC and LPC‐053 (PDB ID 3PS3). An inner box side length of 10 Å was chosen; the side length of the outer box featured additional 15 Å of side length for ligand placement. Molecular docking was carried out using Glide [85, 86, 87] in standard precision (SP) mode within the OPLS3e force field [79]. Flexible ligand sampling and the option to preserve input conformations during conformer generation were enabled. The process of binding pose generation was restricted to the reference position defined by the cocrystallized inhibitor LPC‐053. More precisely, the position of the hydroxamic acid group had to be matched with a tolerance of 1.00 Å. Five binding poses per input conformer were subjected to post‐docking minimization, and one pose per input conformer was reported in the final output. The best docking pose was chosen based on the Emodel descriptor. The docking protocol was previously validated by re‐docking co‐crystallized inhibitors (details can be found in [35, 64, 88, 89]). This is also shown by the results for the protein structure used in the current study: the best‐scored starting position of the ligand in 3PS3 showed an RMSD of 0.2 Å, which underlines the very good performance of the docking settings. MM‐GBSA interaction energies were calculated for all obtained docking poses using Prime MM‐GBSA [90]. Thereby, the VSGB solvation model and the OPLS3e force field [79] were used. During the calculations, only the ligand was defined as a flexible residue.

#### 4.2.4 Molecular Dynamics (MD) Simulations

MD simulations were carried out using Desmond [91, 92]. The protein–ligand complexes were initially solvated in orthorhombic boxes of TIP3P water molecules using the System Builder panel. The box volume was automatically minimized while maintaining a buffer distance of 10 Å between the solute surface and box edges. Additionally, sodium ions were added for system neutralization and placed at a distance > 10 Å from the ligand. Each solvated periodic system was simulated in two independent 50 ns runs. Snapshots were saved every 100 ps to the output trajectory. The residual MD parameters were left at standard settings as predefined by the MD panel: all simulations were performed within the NPT ensemble at 300 K, maintained by the Nosé‐Hoover chain thermostat with a relaxation time of 1 ps, and at 1.01325 bar, maintained by the Martyna‐Tobias‐Klein barostat with a relaxation time of 2 ps. A cutoff radius of 9 Å was chosen for Coulombic interactions. Initial random velocity assignment was ensured by randomly generated seeds. Prior to each MD simulation, the system was relaxed according to the default Desmond relaxation protocol. Trajectory analysis was carried out using the Simulation Interaction Diagram panel as well as the Simulation Event Analysis panel. Quantification of solvent exposures was performed using Schrödinger's Binding Surface Area Analysis tool.

#### 4.2.5 Molecular Visualization and Plot Generation

3D structures of the protein‐ligand complexes were generated using PyMol [93]. The plots were compiled by Microsoft Excel, Matplotlib [94], and Seaborn [95].

### 4.3 Biological Evaluation

#### 4.3.1 Antibacterial Activity

The disk diffusion assays as well as the determination of the MIC values against *E. coli* BL21(DE3) and *E. coli* D22 were conducted as described previously [64].

To determine the MIC against *E. coli* ATCC 35218 and *P. aeruginosa* ATCC 27853 in the presence/absence of the efflux pump inhibitor PAßN, the bacteria were grown overnight on an LB‐agar plate. The next day, one single colony was diluted with sterile saline to yield a suspension visually equivalent to a 0.5 McFarland standard. This initial inoculum was consecutively diluted 1:10 with MHB twice. 25 µL of a twofold dilution series of the compounds in MHB (ranging from 512 µg · mL^−^^1^ to 1 µg · mL^−1^) was dispensed to each well of a 96‐well plate. Then, 25 µL MHB or 25 µL PAßN (256 µg · mL^−1^ in MHB) was added, respectively. Finally, 50 µL of the prepared inoculum was added, resulting in 5 · 10^5^ cfu · mL^−1^, a final concentration range of the test compounds between 128 µg · mL^−1^ and 0.25 µg · mL^−1^, and, in case the efflux pump inhibitor was present, a PAßN concentration of 64 µg · mL^−1^. The plates were incubated for 18 h at 37°C. The MIC was defined as the lowest concentration of the compounds that prevented visible growth after incubation. Each assay was performed at least three times on separate days.

#### 4.3.2 LpxC Enzyme Assays

Expression and purification of *E. coli* LpxC C63A and *P. aeruginosa* LpxC, as well as the performance of the respective enzyme inhibition assays, were conducted as described previously [64]. Each assay was performed at least three times on separate days. The determined *IC*_50_ values were converted into *K*_i_ values using the Cheng–Prusoff equation, thereby employing our experimentally determined *K*_M_ values of *E. coli* LpxC C63A (3.6 µM) and *P. aeruginosa* LpxC (4.7 µM) for the deacetylation of **7** [64, 96].

#### 4.3.3 ADME *In Vitro* Studies

The plasma stability assay, the metabolic stability assay, and the plasma protein binding assay were conducted as described previously [97].

#### 4.3.4 HPLC‐MS/MS Analysis

Samples were analyzed using an Agilent 1290 Infinity II HPLC system coupled to an AB Sciex QTrap 6500plus mass spectrometer using the same LC conditions as described previously [64]. Mass transitions for controls and compounds are depicted in Supporting Information: Table S1.

#### 4.3.5 Cytotoxicity Tests

Cytotoxicity tests were conducted as described previously [98]. In brief, HepG2 (ATCC HB‐8065TM), VeroE6 (ATCC CRL‐1586), and HEK293 (ATCC CRL‐1573) cells were each cultivated in Dulbecco's modified Eagle's medium (DMEM) with 10% heat‐inactivated fetal calf serum (FCS) at 37°C and 5% CO_2_. HK‐2 (ATCC CRL‐2190) cells were cultivated in keratinocyte‐serum‐free medium supplemented with 0.05 mg/mL bovine pituitary extract and 5 ng/mL human recombinant epidermal growth factor. Compounds **9**, *ent*‐**9**, *ent*‐**10**, *ent*‐**12**, **13**, **15**, *ent*‐**15**, and **16** were assessed at final concentrations of 1, 10, and 100 µM. Assays were conducted in biological triplicates. Results were visualized using GraphPad Prism 10.5.

## Conflicts of Interest

The authors declare no conflicts of interest.

## Supporting information

Supporting File 1

Supporting File 2

## Data Availability

The data that support the findings of this study are available from the corresponding author upon reasonable request.
