# Supplementary material for: Synthesis, Biological Evaluation, and Molecular Docking Studies of 2r,3t,4c‐Configured C‐Furanosidic LpxC Inhibitors
Source: Arch Pharm (Weinheim). 2026 Jul 7;359(6):e70270. doi: 10.1002/ardp.70270 (PMC13342928; doi:10.1002/ardp.70270)
Supplement: Supplementary file 1 — Supporting File 1 [file ARDP-359-e70270-s002.pdf]

Supporting Information

Synthesis, biological evaluation, and molecular docking studies of 2*r*,3*t*,4*c*-configured *C*-furanosidic LpxC inhibitors

André Behnk,[a,b] Fabian Lüttchens,[a,b] Frederick Wichter,[a,b] Katharina Hoff,[a,b] Elisa Venanzi,[a] Stefan Wimmer,[a] Christopher Vorreiter,[c] Katharina Rox,[d,e,f] Wolfgang Sippl,[c] and Ralph Holl[a,b,*]

[a]Institute of Organic Chemistry, Universität Hamburg, Martin-Luther-King-Platz 6, 20146 Hamburg, Germany

[b]German Center for Infection Research (DZIF), partner site Hamburg-Lübeck-Borstel-Riems

[c]Institute of Pharmacy, Martin-Luther-University of Halle-Wittenberg, Kurt-Mothes-Str. 3, 06120 Halle (Saale), Germany

[d]Department of Chemical Biology, Helmholtz Centre for Infection Research (HZI), Inhoffenstr. 7, 38124 Braunschweig, Germany

[e]German Center for Infection Research (DZIF), partner site Hannover-Braunschweig, Inhoffenstr. 7, 38124 Braunschweig, Germany

[f]Research group Pharmacokinetics and Pharmacodynamics Unit, Helmholtz Centre for Infection Research (HZI), Inhoffenstr. 7, 38124 Braunschweig, Germany

*To whom correspondence should be addressed. Tel.: +49-40-2395-22825; E-mail: ralph.holl@uni-hamburg.de

Contents

Figure S1	S3
Figure S2	S4
Figure S3	S5 – S6
Figure S4	S7 – S8
Figure S5	S9
Table S1	S10
^{1}H and ^{13}C NMR spectra of representative compounds	S11 – S37
HPLC traces of the synthesized compounds	S38 – S50
HRMS spectra of the synthesized compounds	S51 – S67

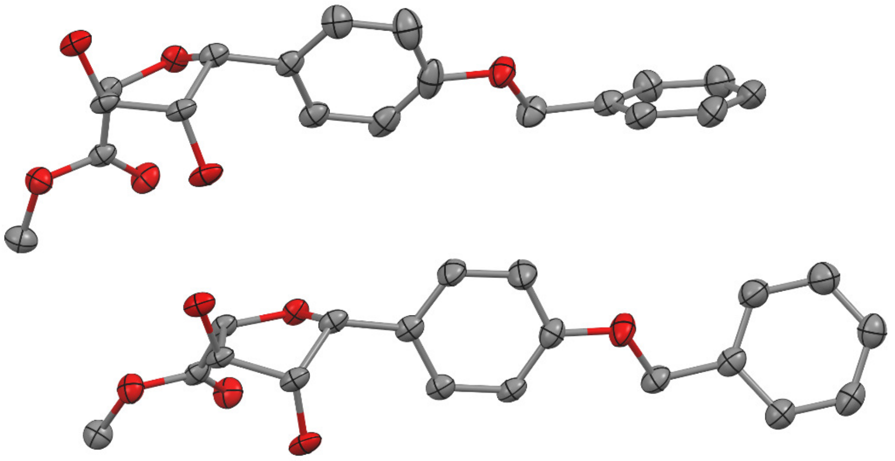

Figure S1: ORTEP-like picture of the X-ray crystal structure of *C*-furanoside **42** (ellipsoids are at a probability level of 50%, H atoms are not shown for clarity reasons). Note that the asymmetric unit comprises two molecules that have different conformations of the benzyloxy moiety. The slight positional disorder, which is present at the benzyloxy moiety in the upper molecule, has been removed for this image.

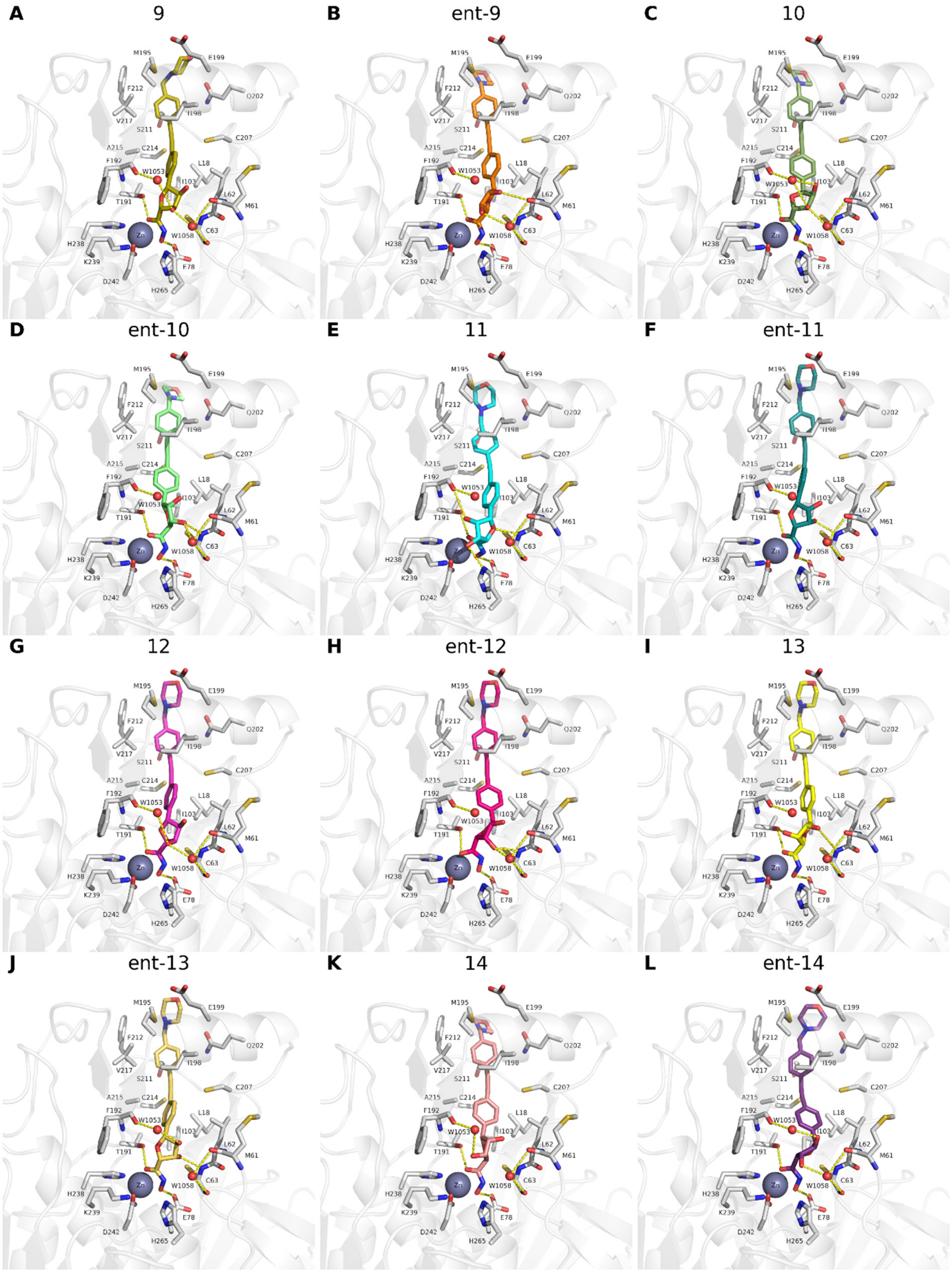

Figure S2: Generated docking poses for the residual compounds. The inhibitors and surrounding pocket residues are shown with sticks, the zinc ion is visualized as grey sphere. Hydrogen bonds are depicted with yellow dashed lines.

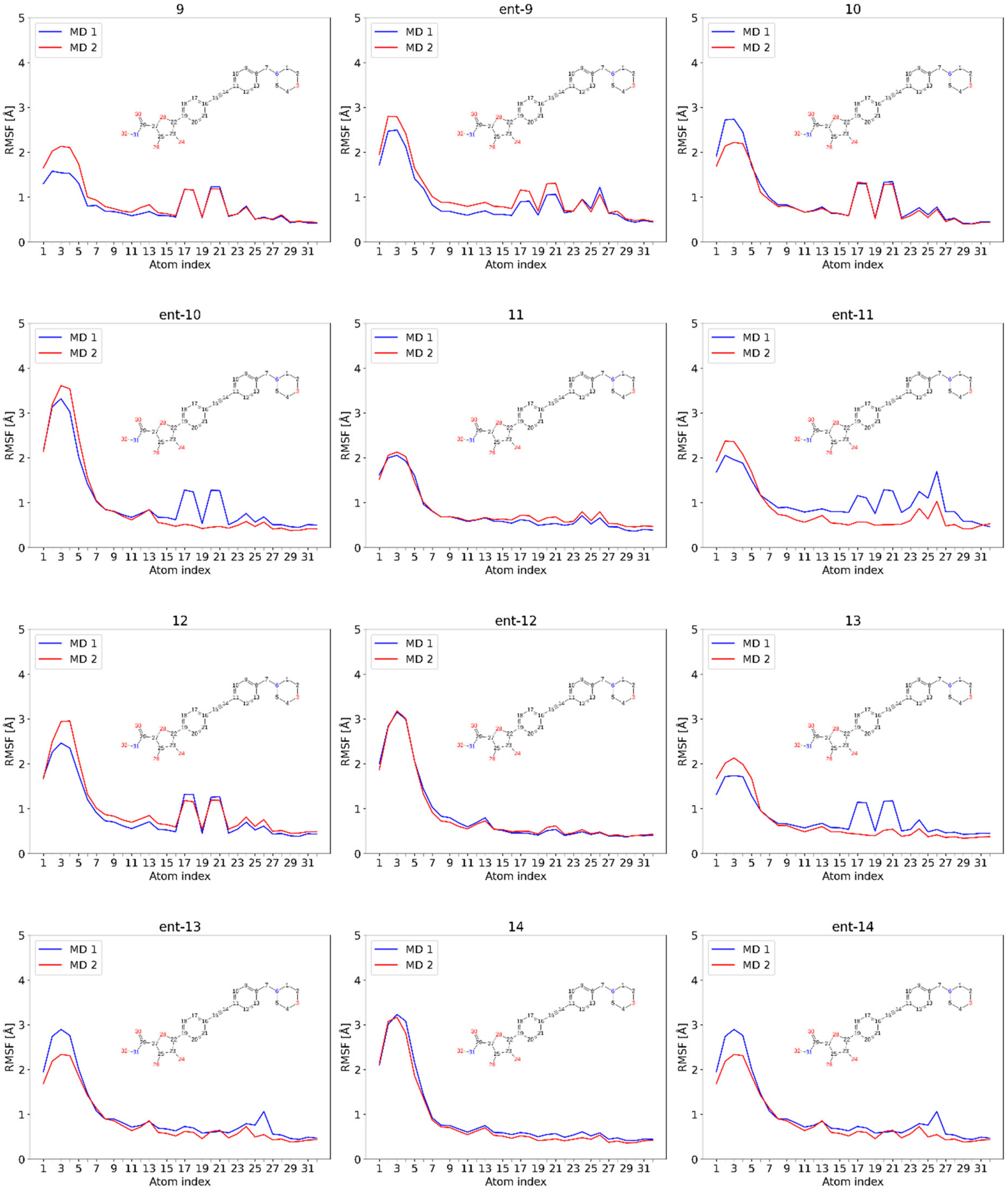
9
ent-9
10
ent-10
11
ent-11
12
ent-12
13
ent-13
14
ent-14
MD 1
MD 2
RMSF [Å]
Atom index

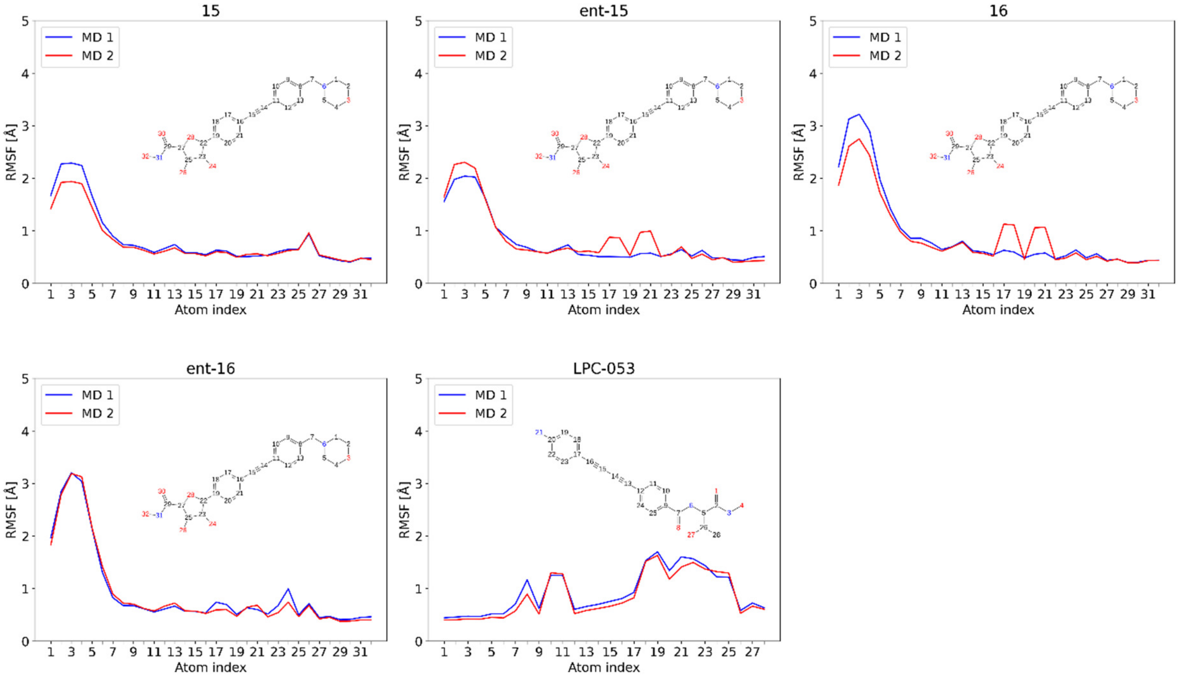

Figure S3: Ligand atom fluctuations (RMSF) calculated for all performed MD simulations.

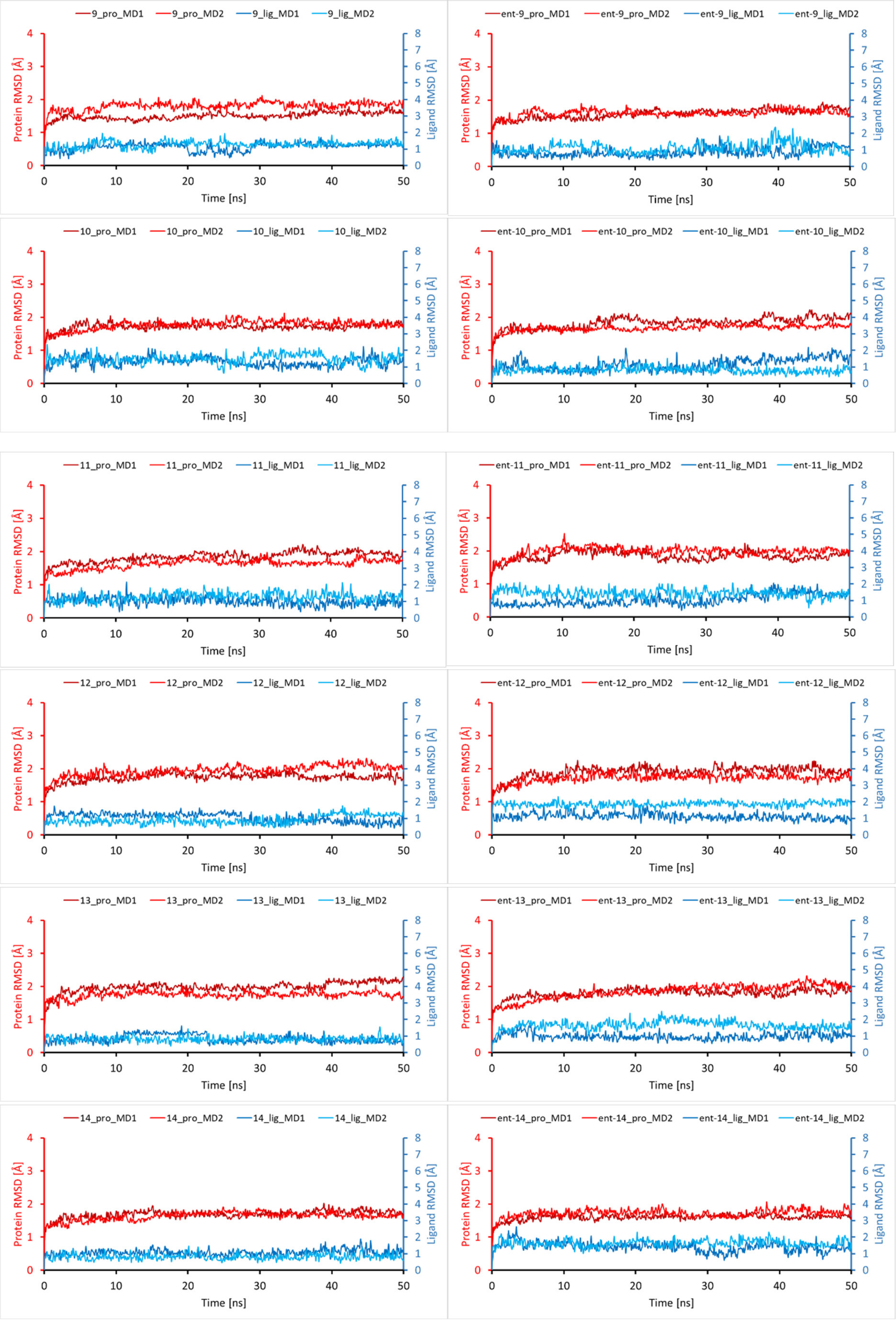
9_pro_MD1 9_pro_MD2 9_lig_MD1 9_lig_MD2
ent-9_pro_MD1 ent-9_pro_MD2 ent-9_lig_MD1 ent-9_lig_MD2
10_pro_MD1 10_pro_MD2 10_lig_MD1 10_lig_MD2
ent-10_pro_MD1 ent-10_pro_MD2 ent-10_lig_MD1 ent-10_lig_MD2
11_pro_MD1 11_pro_MD2 11_lig_MD1 11_lig_MD2
ent-11_pro_MD1 ent-11_pro_MD2 ent-11_lig_MD1 ent-11_lig_MD2
12_pro_MD1 12_pro_MD2 12_lig_MD1 12_lig_MD2
ent-12_pro_MD1 ent-12_pro_MD2 ent-12_lig_MD1 ent-12_lig_MD2
13_pro_MD1 13_pro_MD2 13_lig_MD1 13_lig_MD2
ent-13_pro_MD1 ent-13_pro_MD2 ent-13_lig_MD1 ent-13_lig_MD2
14_pro_MD1 14_pro_MD2 14_lig_MD1 14_lig_MD2
ent-14_pro_MD1 ent-14_pro_MD2 ent-14_lig_MD1 ent-14_lig_MD2
Protein RMSD [Å]
Ligand RMSD [Å]
Time [ns]

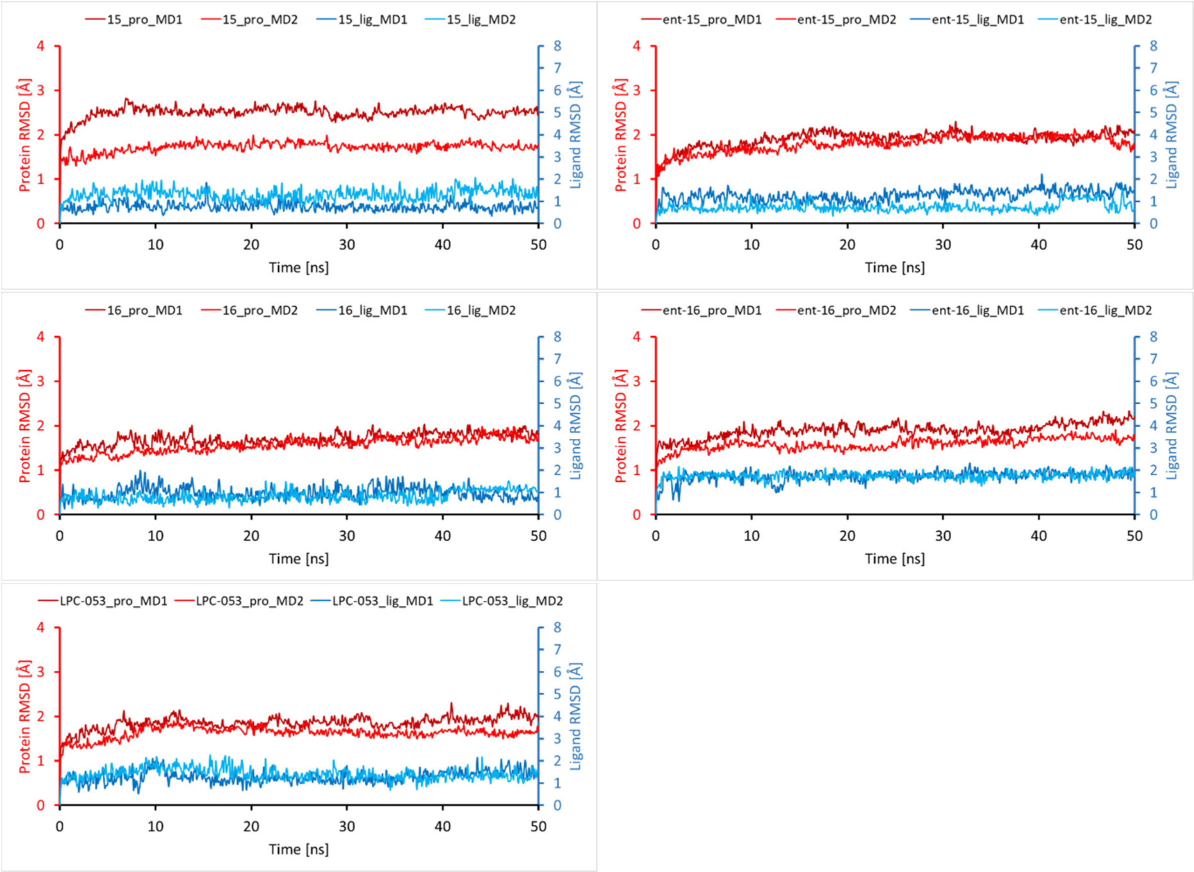

Figure S4: Protein and ligand heavy atom RMSD values calculated for all performed MD simulations.

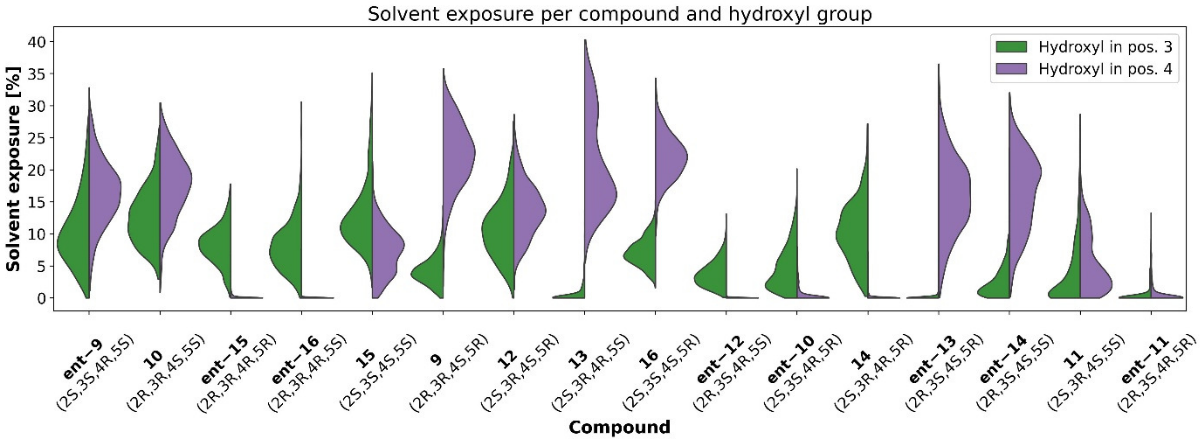

Figure S5: Distribution of hydroxyl solvent exposures calculated for all *C*-glycoside compounds across the performed MD simulations.

Table S1: Mass transitions of the internal standard (caffeine) and the *C*-furanosides.

ID	***Q1* [Da]**	***Q3* [Da]**	***DP* [V]**	***CE* [V]**	***CXP* [V]**
caffeine	195.024	138.0<br>110.0	130.0<br>130.0	25.0<br>31.0	14.0<br>18.0
stereoisomeric *C*-furanosides	439.285	352.228<br>334.207<br>189.157	130.0<br>130.0<br>130.0	24.0<br>38.0<br>104.0	26.0<br>24.0<br>20.0

DP: declustering potential; CE: collision energy; CXP: collision cell exit potential.

^{1}H and ^{13}C NMR spectra of representative compounds

Compound **15**

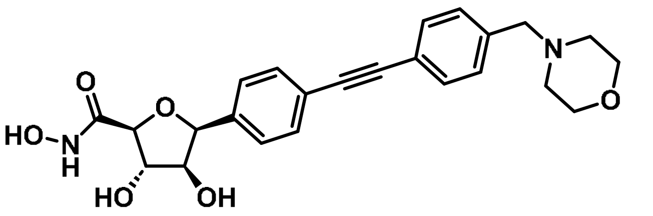

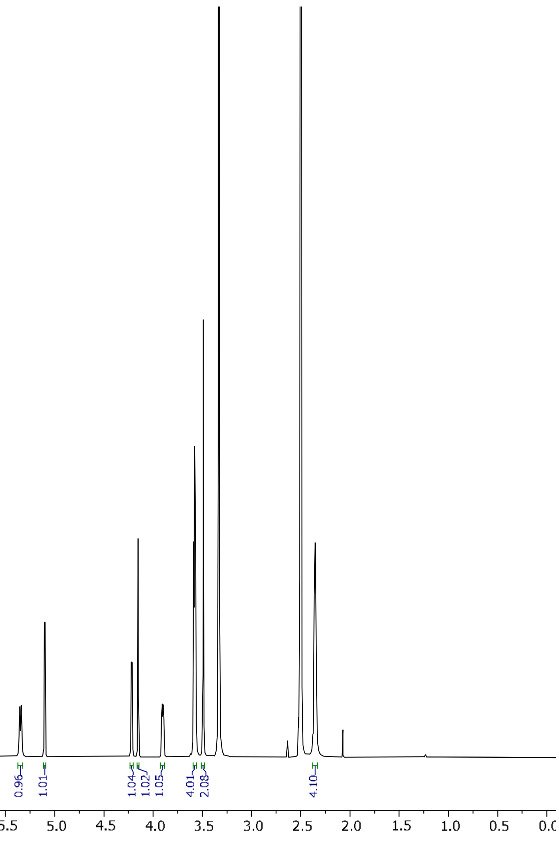

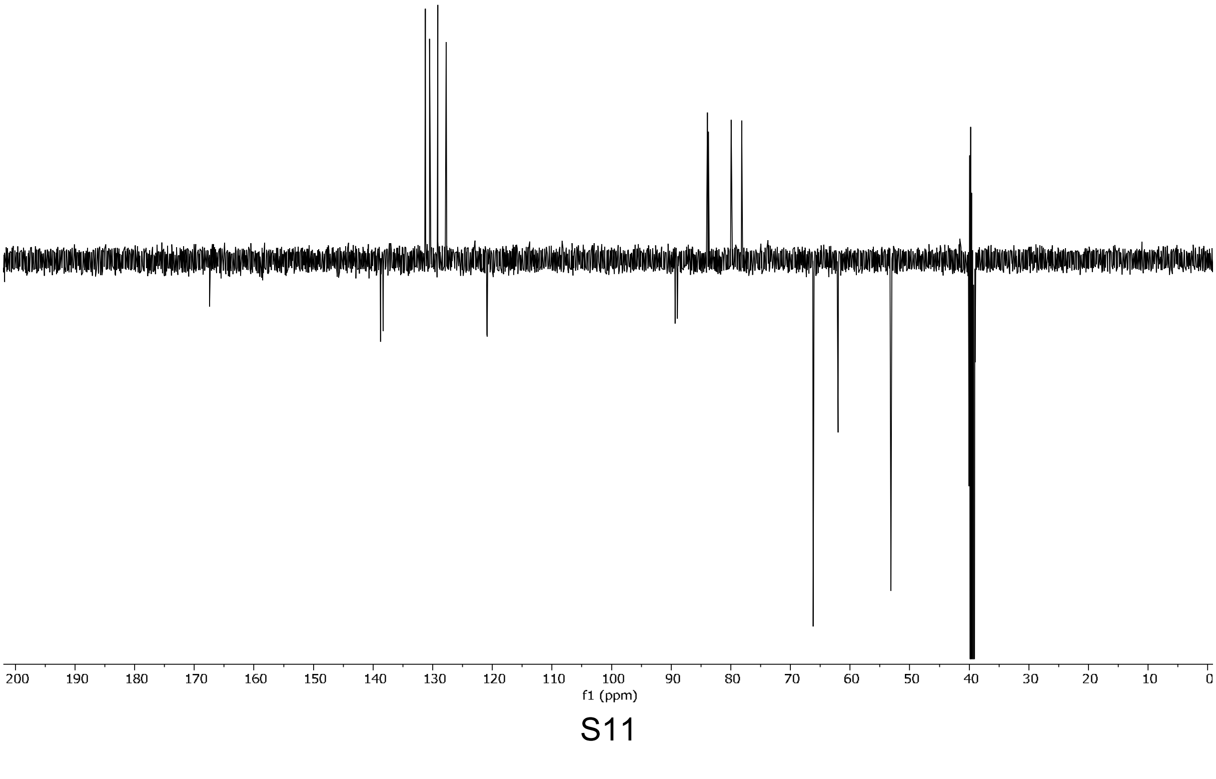

Compound **16**

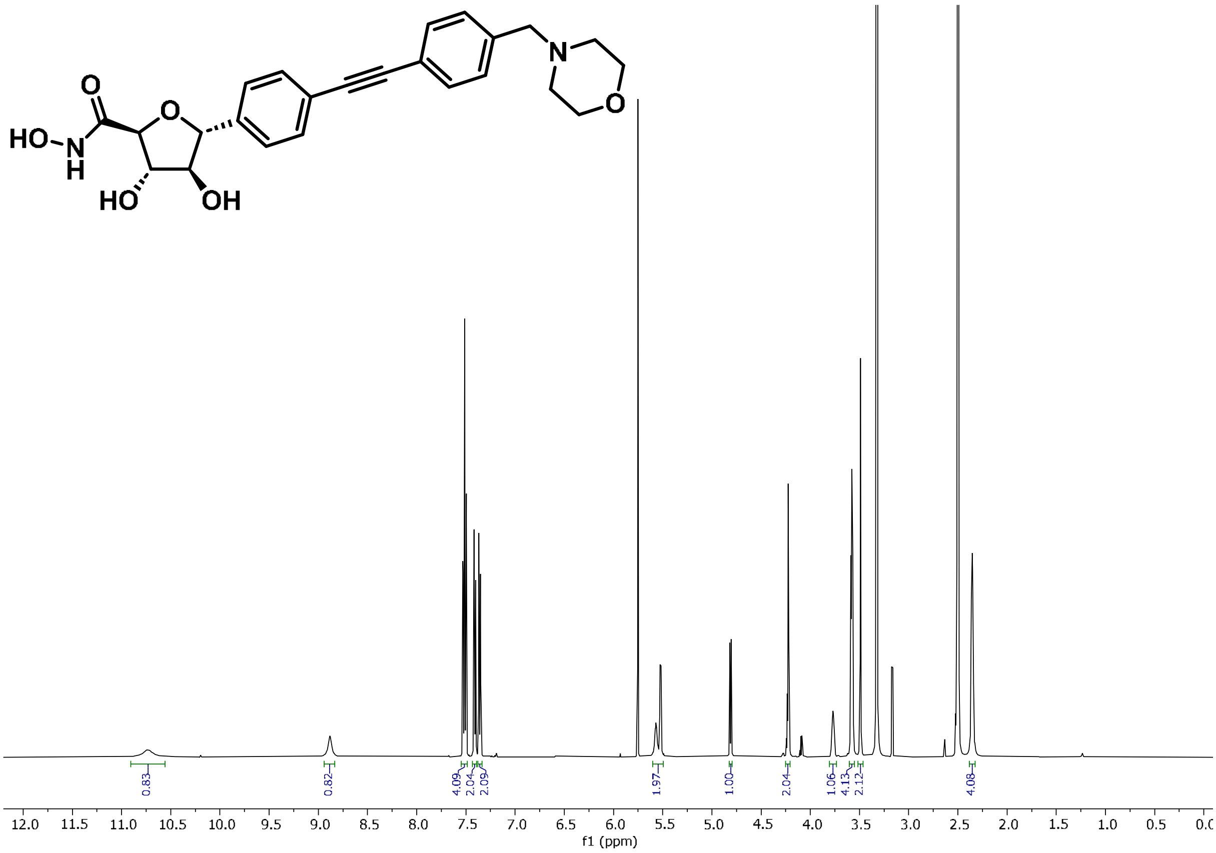

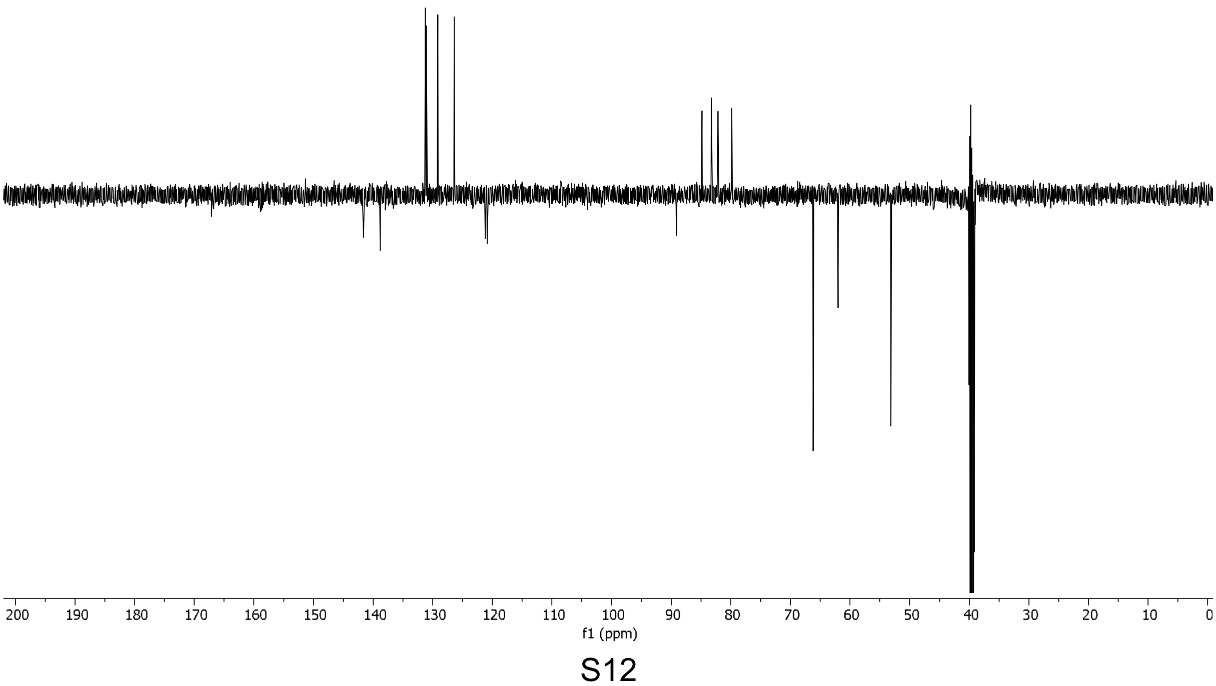

Compound **19**

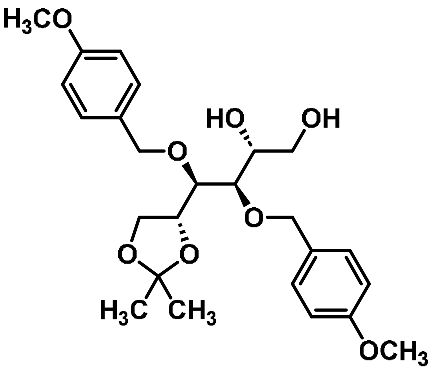

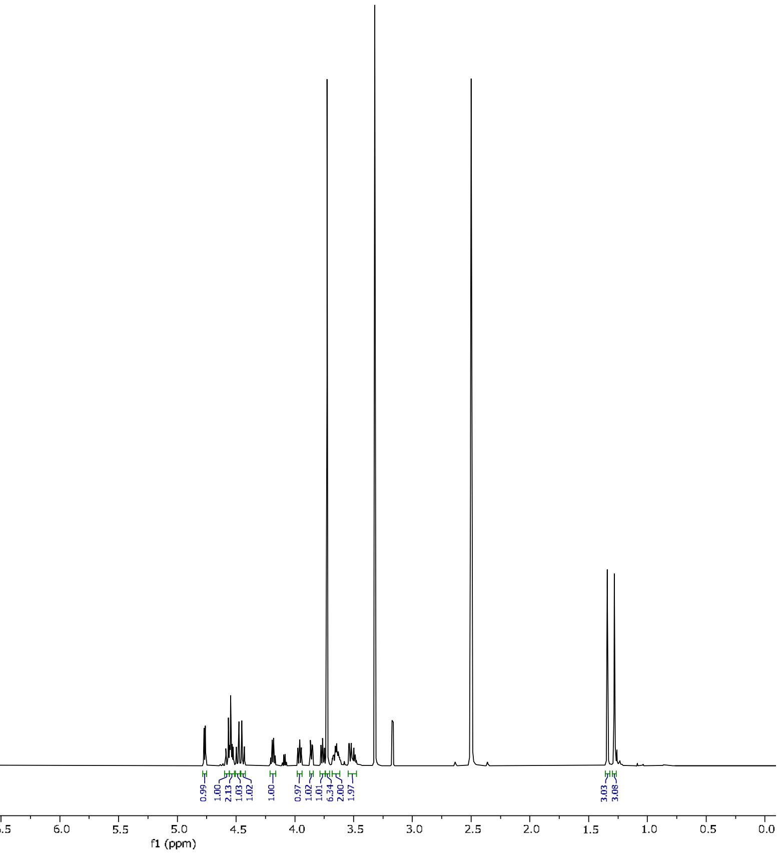

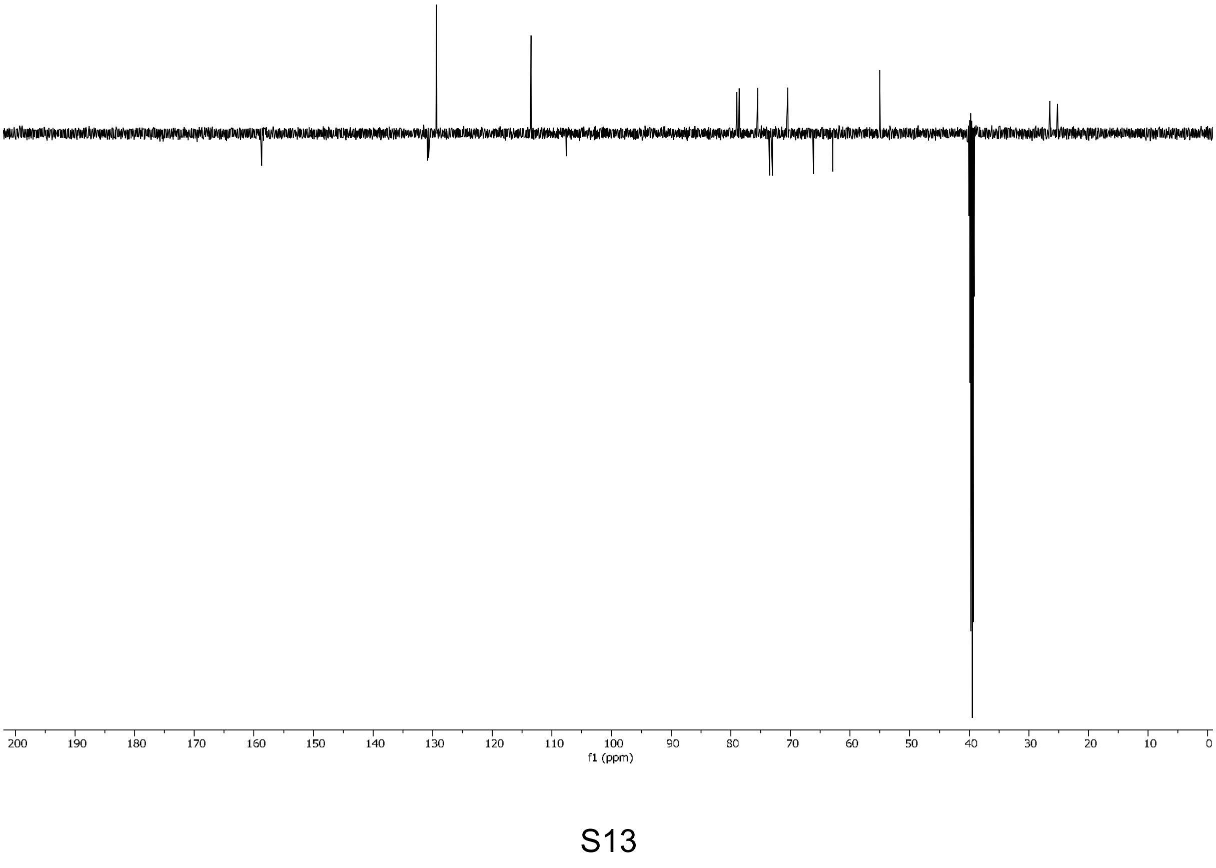

Compound **20**

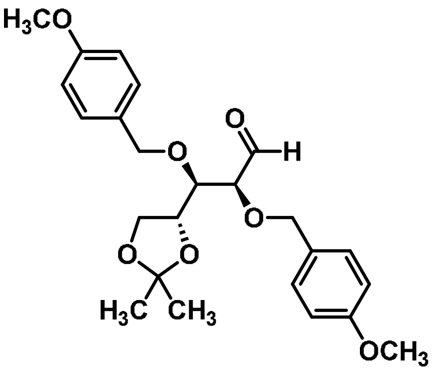
H3CO
O
H
O
O
O
O
H3C
CH3
OCH3

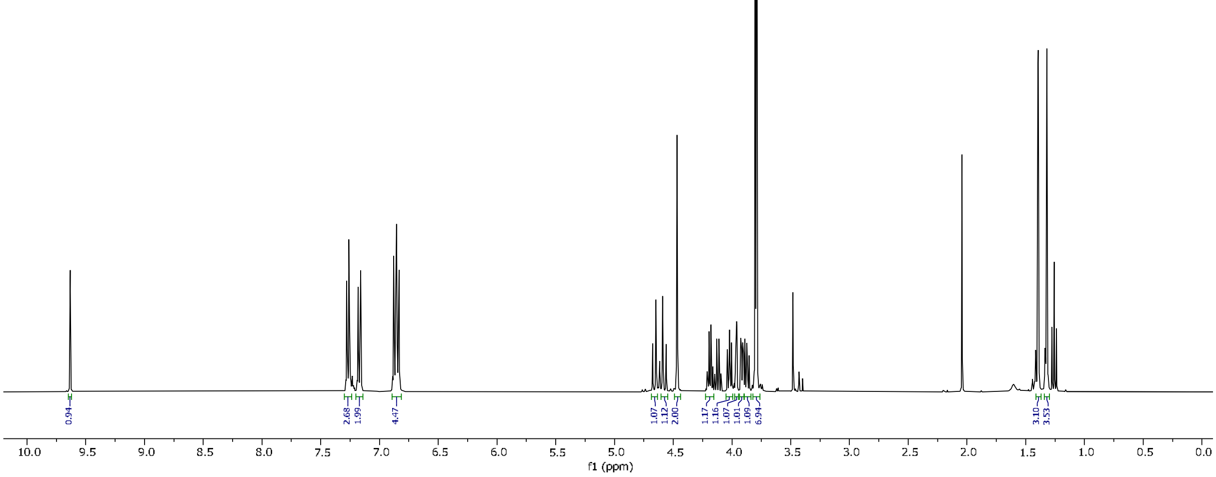
0.94
2.68
1.99
4.47
1.07
1.12
2.00
1.17
1.16
1.07
1.01
1.09
6.94
3.10
3.53
10.0
9.5
9.0
8.5
8.0
7.5
7.0
6.5
6.0
5.5
5.0
4.5
4.0
3.5
3.0
2.5
2.0
1.5
1.0
0.5
0.0
f1 (ppm)

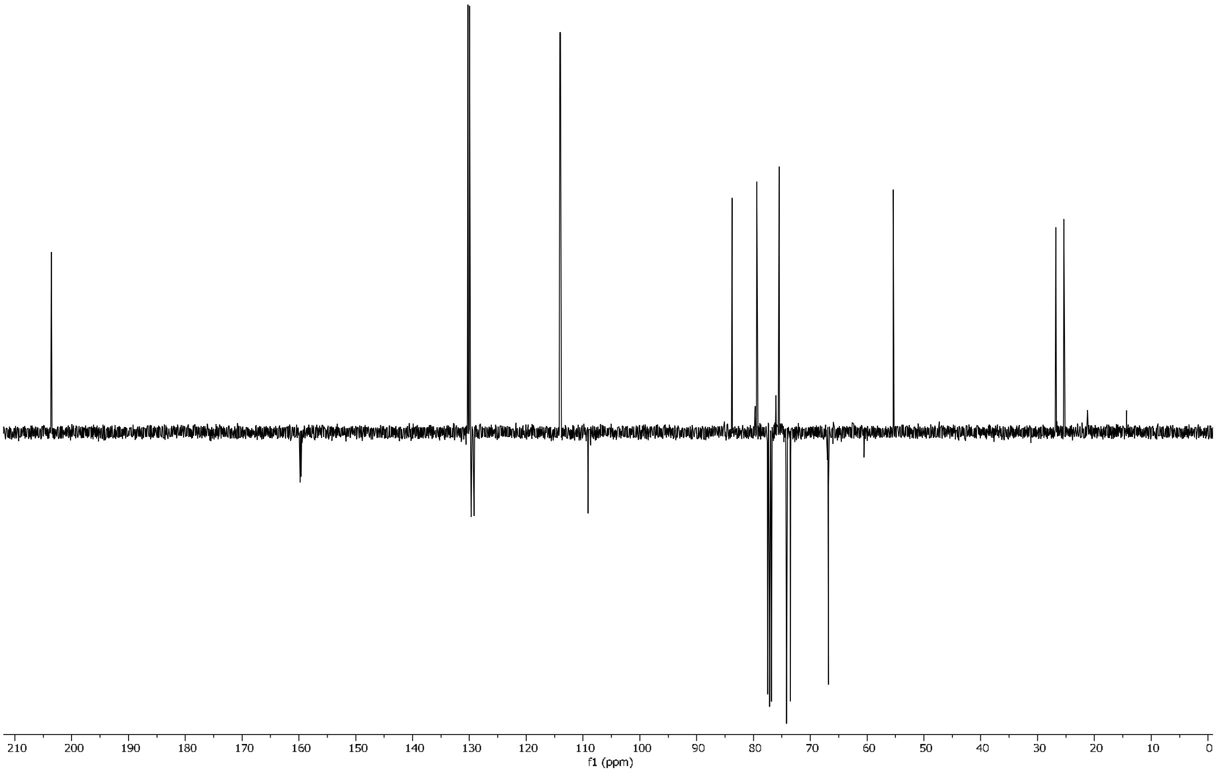
210
200
190
180
170
160
150
140
130
120
110
100
90
80
70
60
50
40
30
20
10
0
f1 (ppm)

Compound **21**

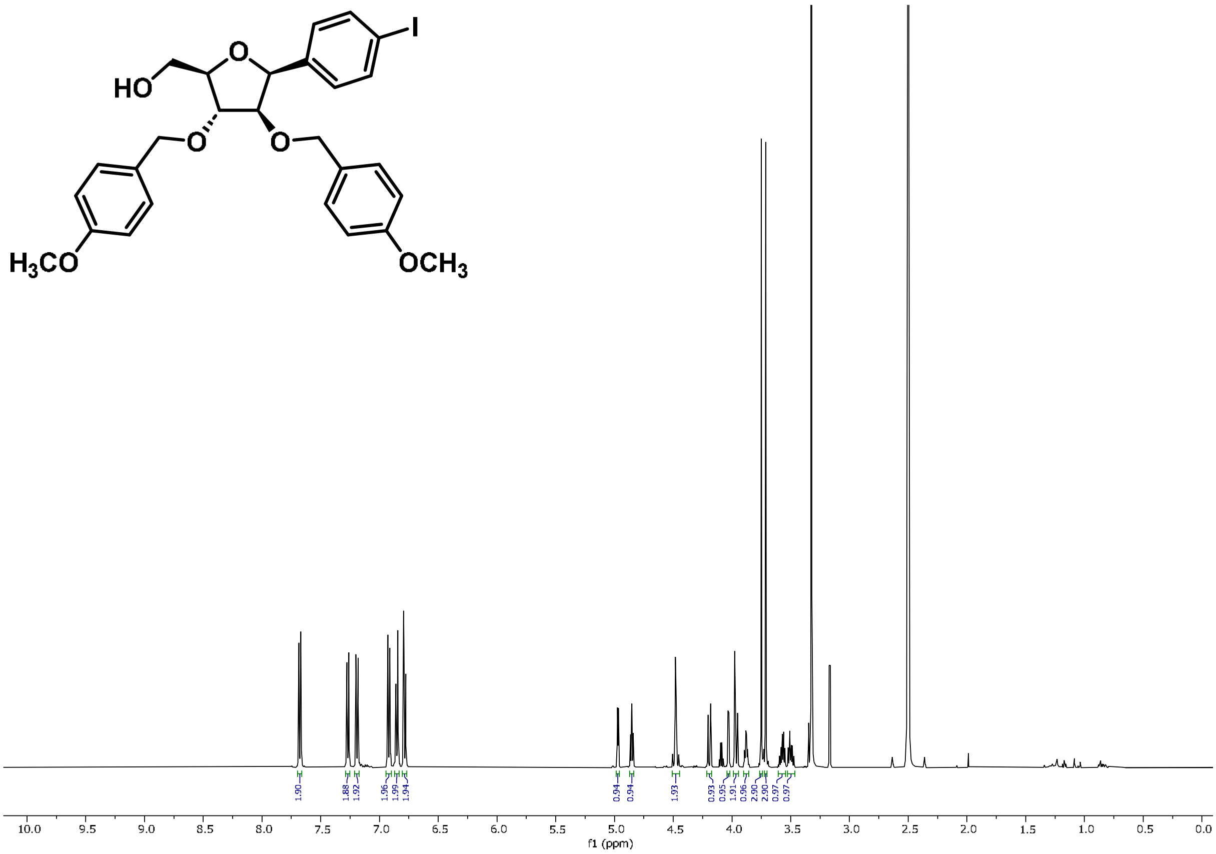
HO
O
I
O
O
H3CO
OCH3
10.0 9.5 9.0 8.5 8.0 7.5 7.0 6.5 6.0 5.5 5.0 4.5 4.0 3.5 3.0 2.5 2.0 1.5 1.0 0.5 0.0
f1 (ppm)

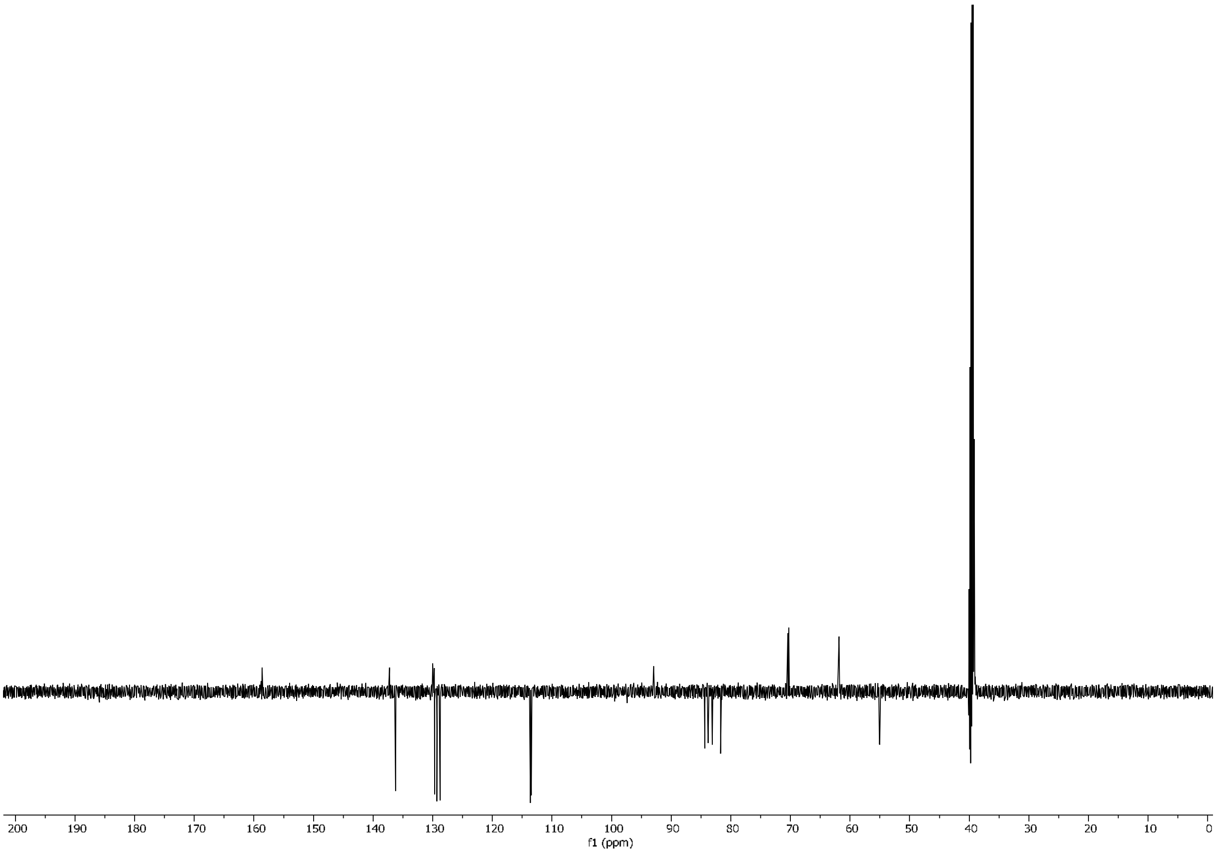
200 190 180 170 160 150 140 130 120 110 100 90 80 70 60 50 40 30 20 10 0
f1 (ppm)

Compound **22**

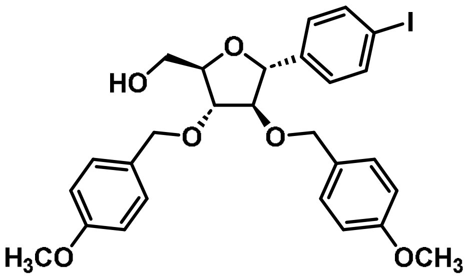

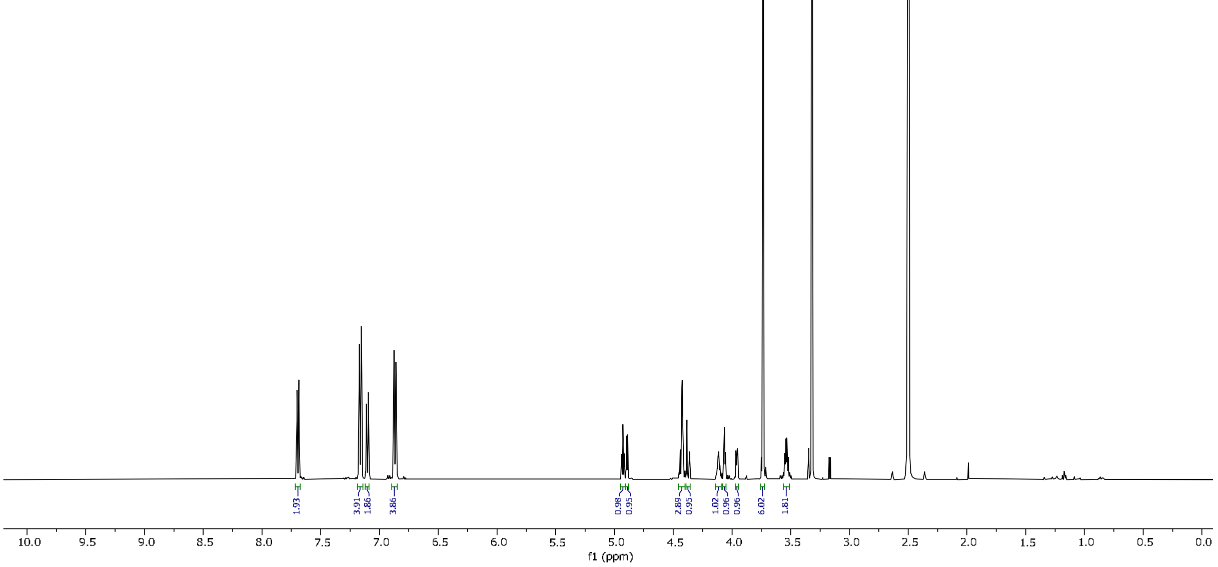

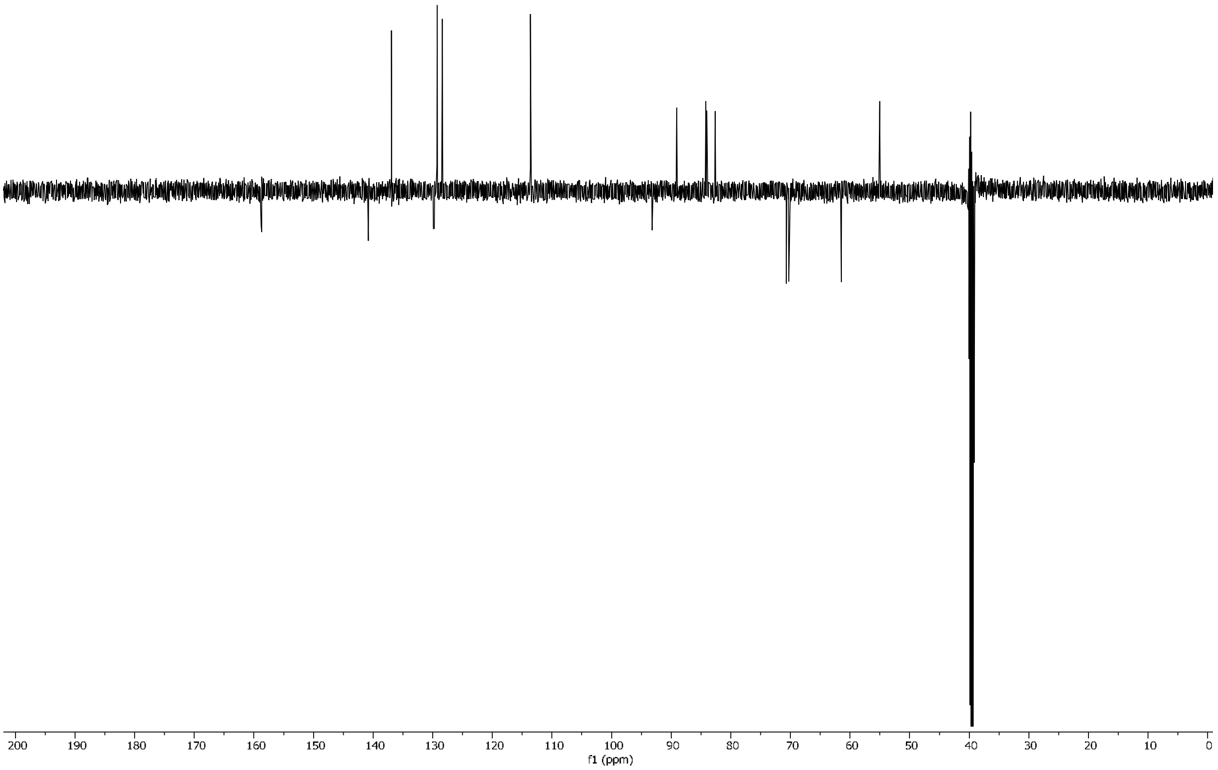

Compound **23**

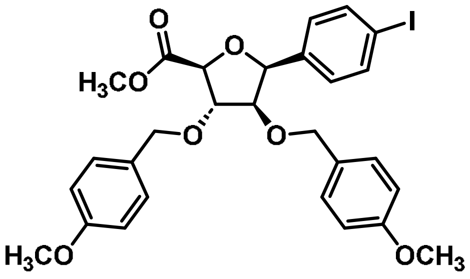

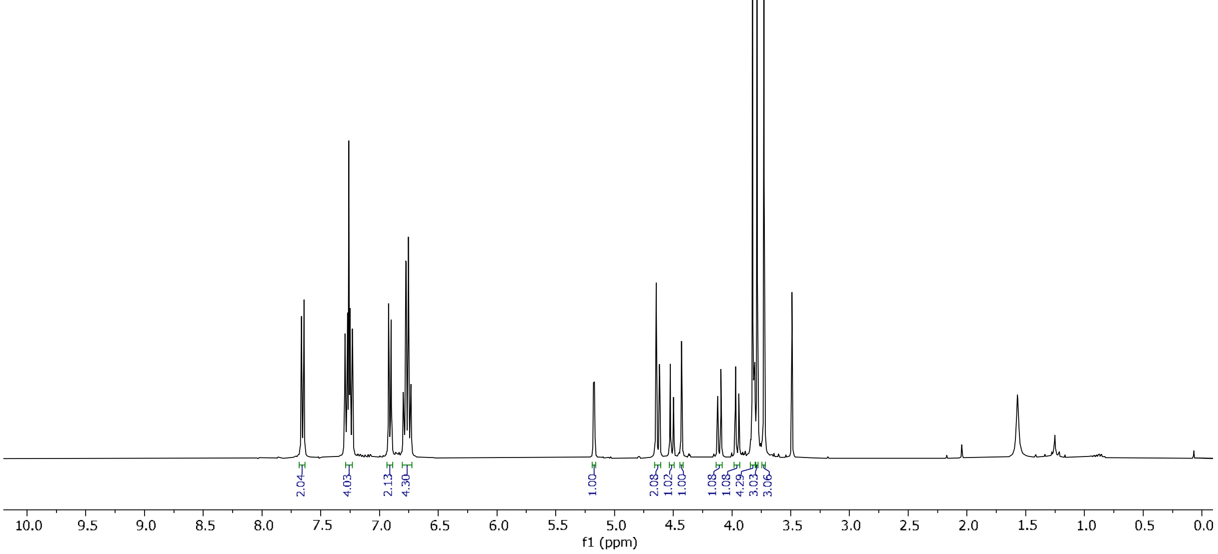

2.04
4.03
2.13
4.30
1.00
2.08
1.02
1.00
1.08
1.08
4.29
3.03
3.06
10.0
9.5
9.0
8.5
8.0
7.5
7.0
6.5
6.0
5.5
5.0
4.5
4.0
3.5
3.0
2.5
2.0
1.5
1.0
0.5
0.0
f1 (ppm)

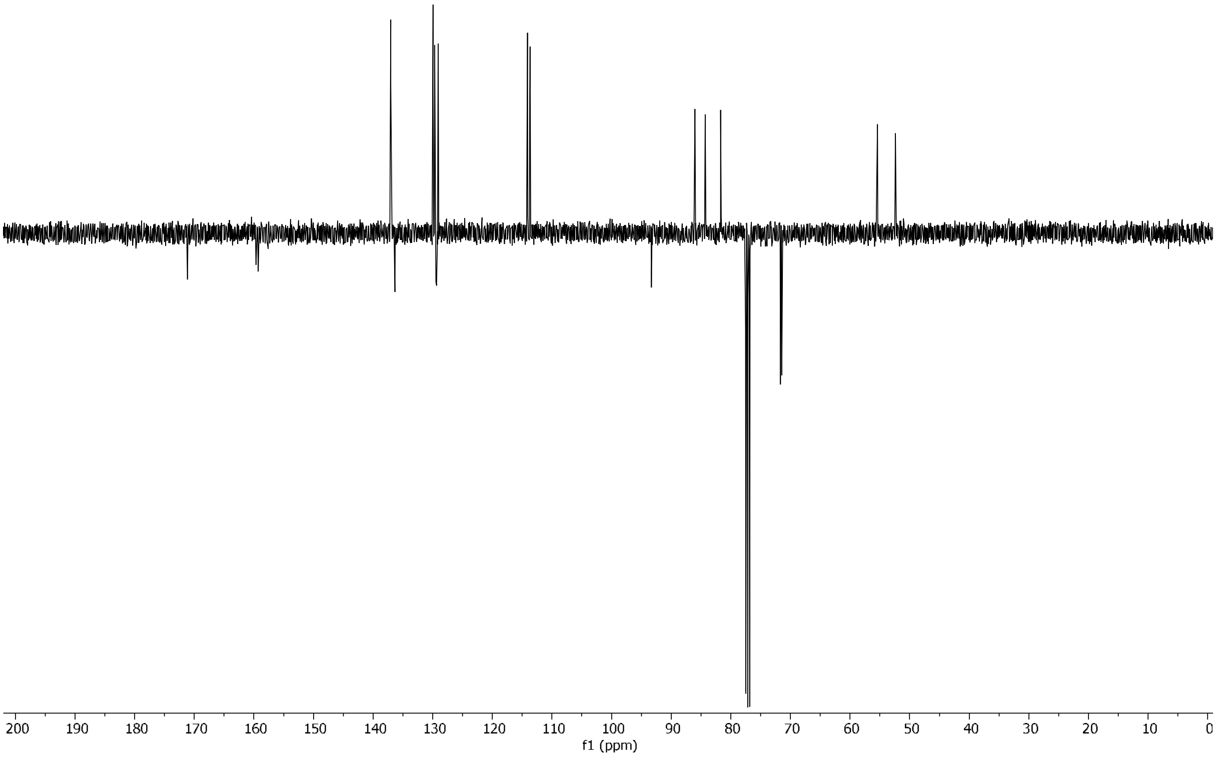

200
190
180
170
160
150
140
130
120
110
100
90
80
70
60
50
40
30
20
10
0
f1 (ppm)

Compound **24**

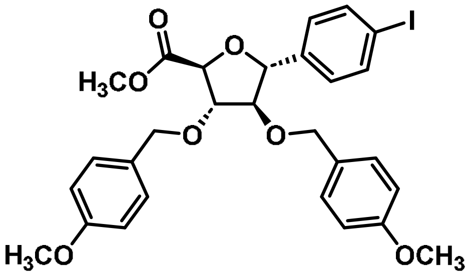
O
O
I
H3CO
O
O
H3CO
OCH3

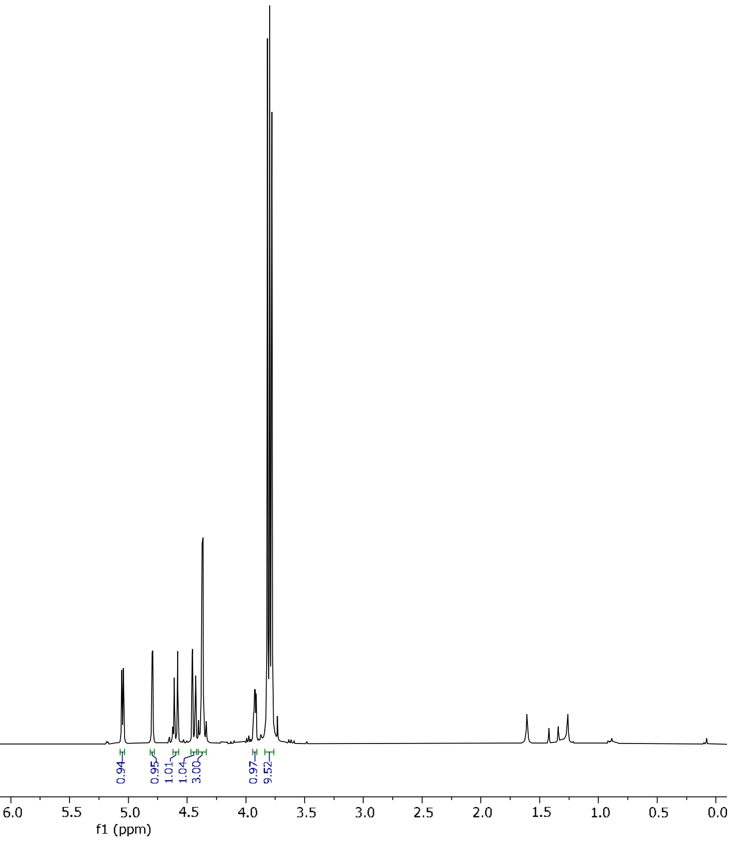
1.97
6.06
2.01
2.02
0.94
0.95
1.01
1.04
3.00
0.97
9.52
10.0 9.5 9.0 8.5 8.0 7.5 7.0 6.5 6.0 5.5 5.0 4.5 4.0 3.5 3.0 2.5 2.0 1.5 1.0 0.5 0.0
f1 (ppm)

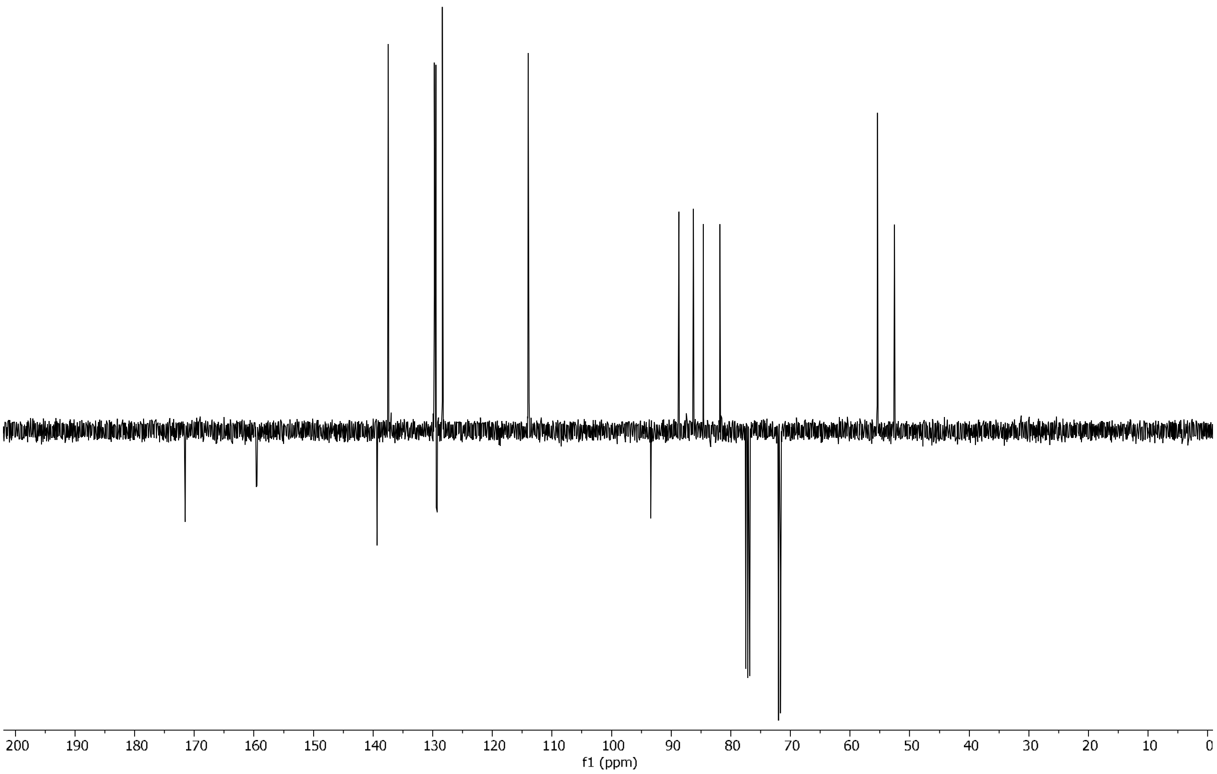
200 190 180 170 160 150 140 130 120 110 100 90 80 70 60 50 40 30 20 10 0
f1 (ppm)

Compound **25**

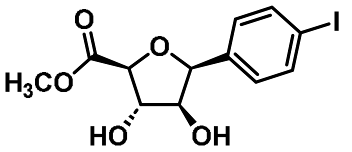
O
O
H_3CO
HO
OH
I

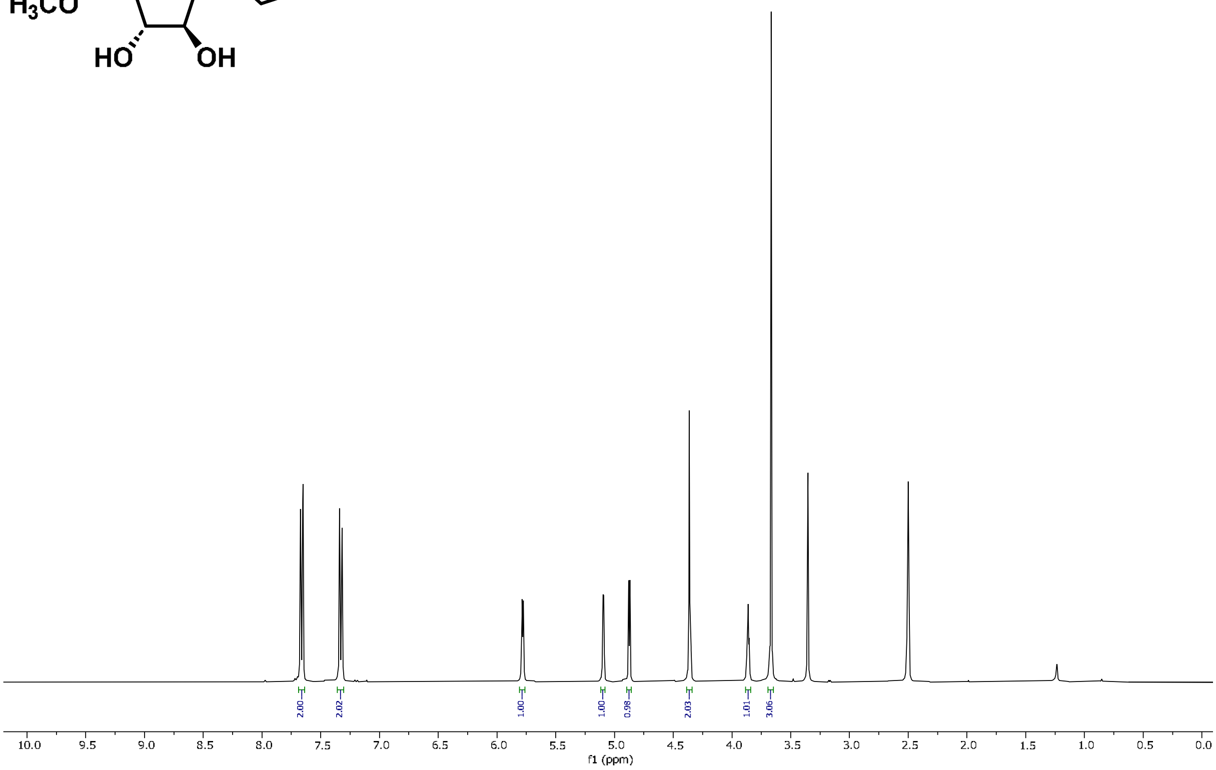
2.00
2.02
1.00
1.00
0.98
2.03
1.01
3.06
10.0 9.5 9.0 8.5 8.0 7.5 7.0 6.5 6.0 5.5 5.0 4.5 4.0 3.5 3.0 2.5 2.0 1.5 1.0 0.5 0.0
f1 (ppm)

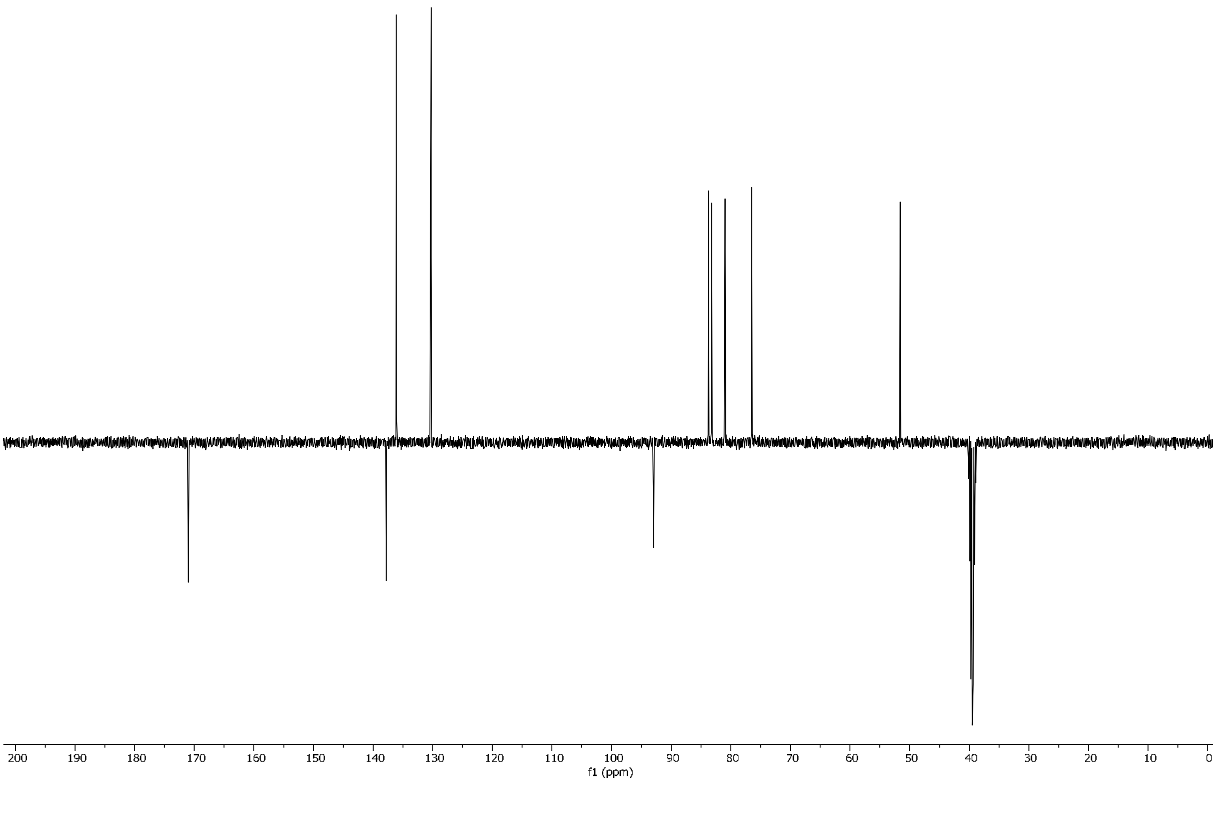
200 190 180 170 160 150 140 130 120 110 100 90 80 70 60 50 40 30 20 10 0
f1 (ppm)

Compound **26**

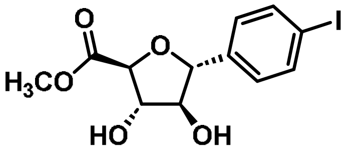
O
O
H3CO
I
HO
OH

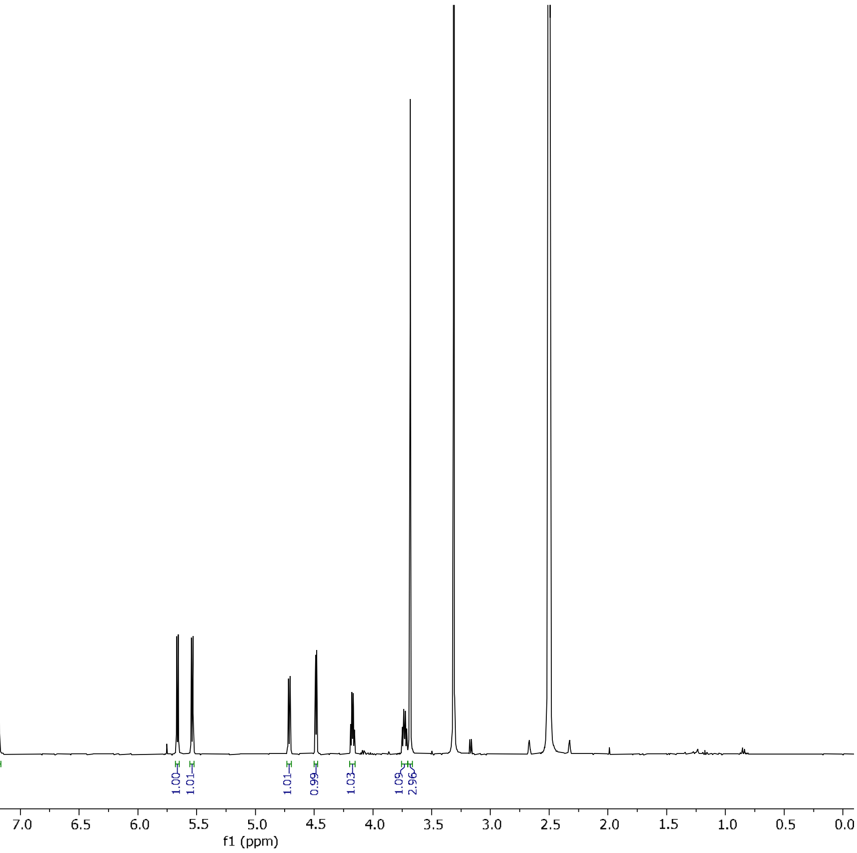
2.00
2.05
1.00
1.01
1.01
0.99
1.03
1.09
2.96
10.0 9.5 9.0 8.5 8.0 7.5 7.0 6.5 6.0 5.5 5.0 4.5 4.0 3.5 3.0 2.5 2.0 1.5 1.0 0.5 0.0
f1 (ppm)

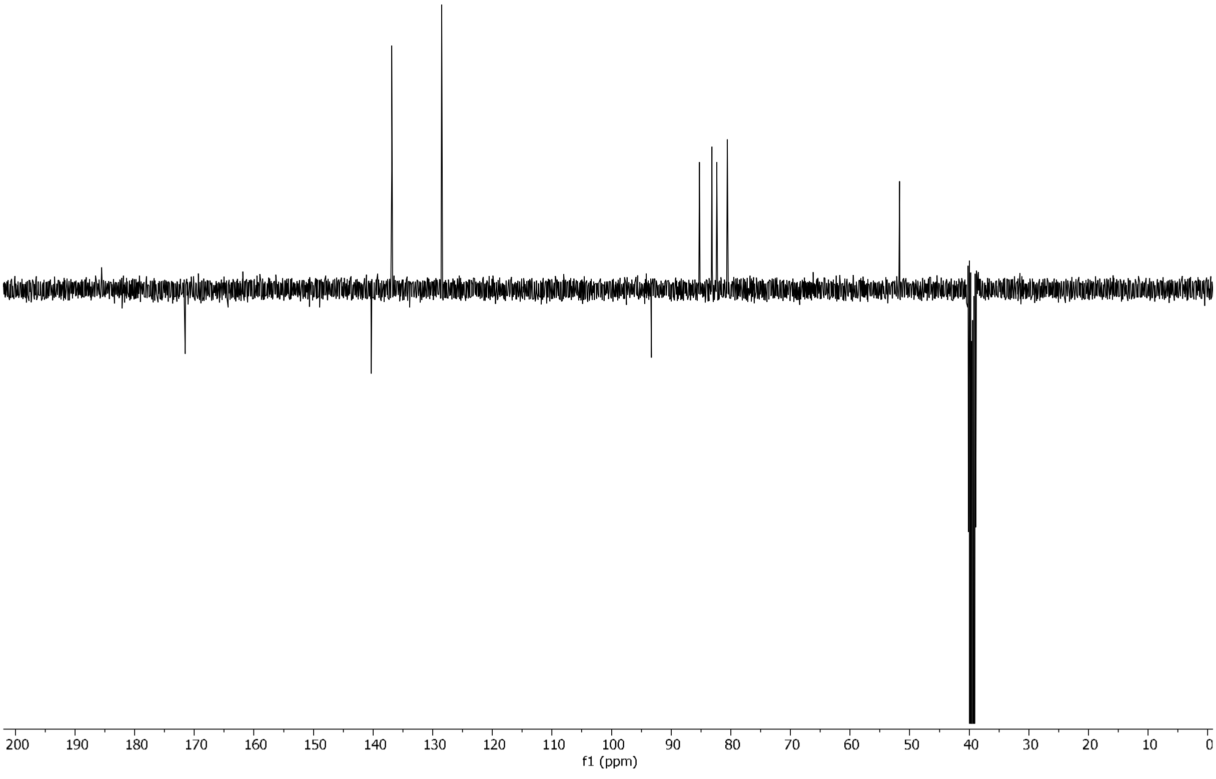
200 190 180 170 160 150 140 130 120 110 100 90 80 70 60 50 40 30 20 10 0
f1 (ppm)

Compound **27**

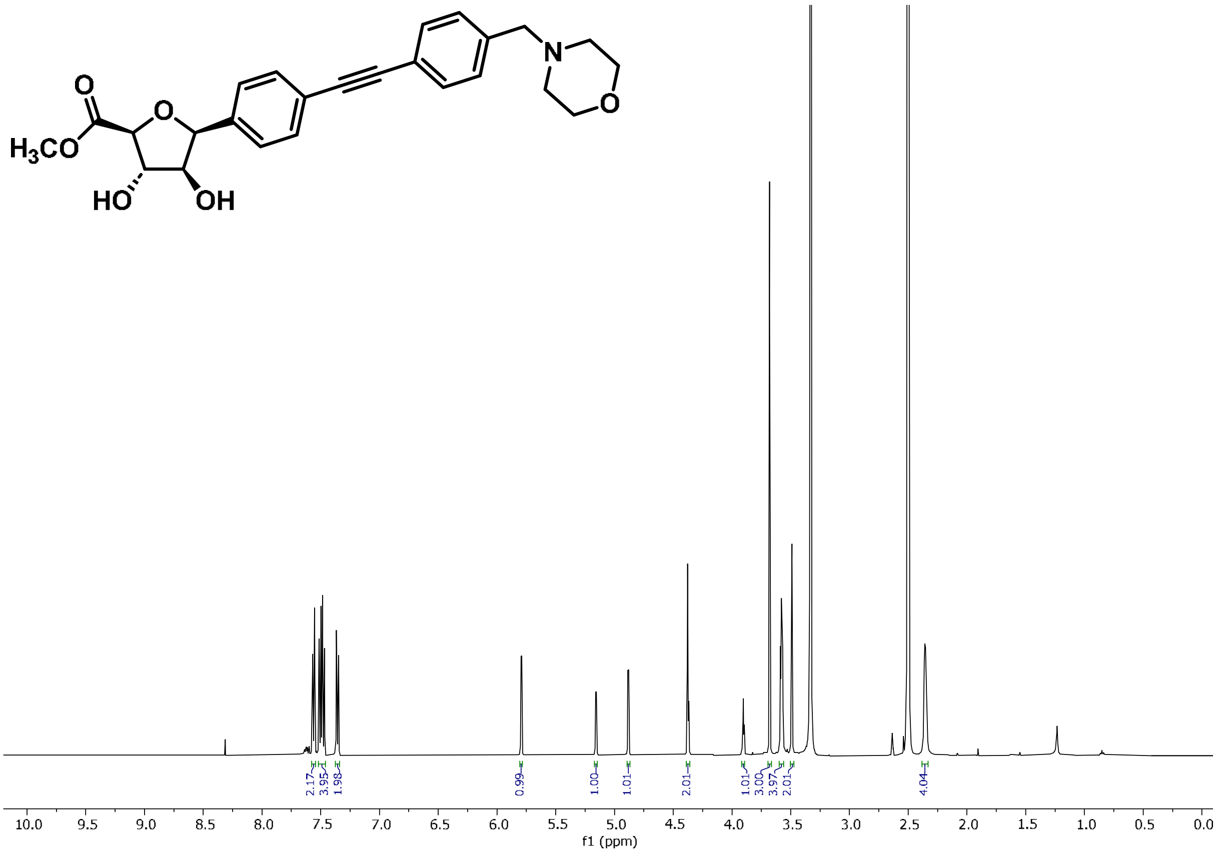

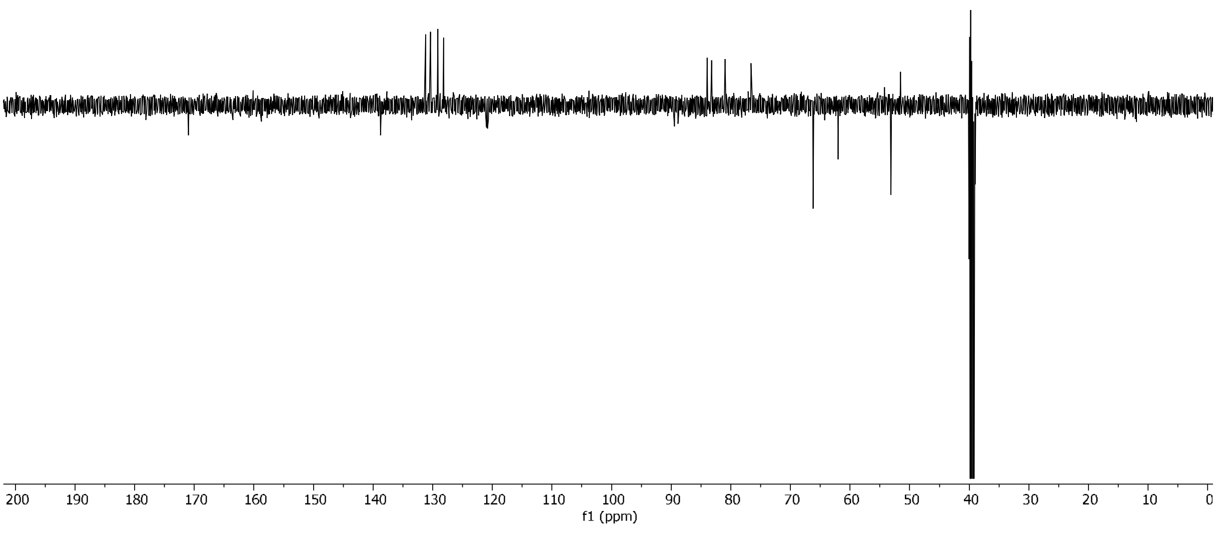

Compound **28**

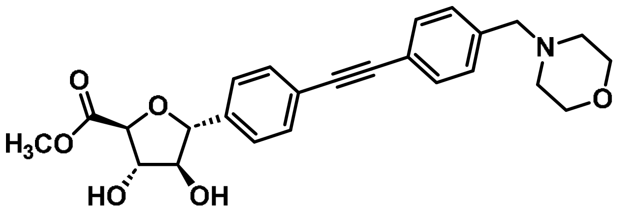

O
O
H3CO
HO
OH
N
O

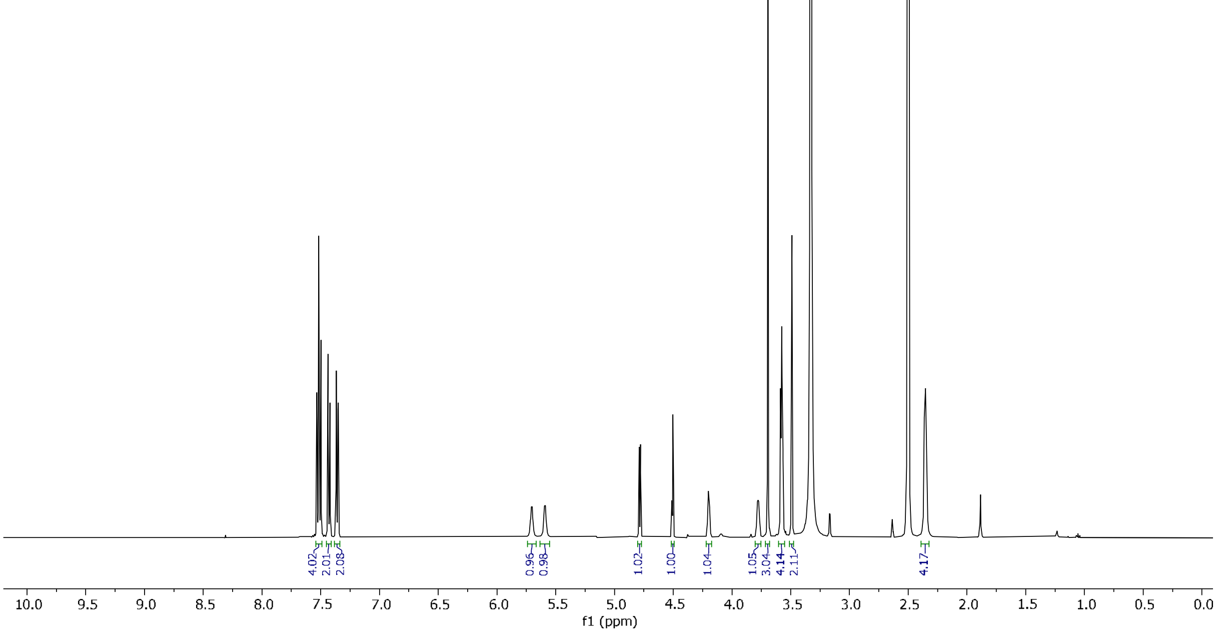

4.02
2.01
2.08
0.96
0.98
1.02
1.00
1.04
1.05
3.04
4.14
2.11
4.17
10.0
9.5
9.0
8.5
8.0
7.5
7.0
6.5
6.0
5.5
5.0
4.5
4.0
3.5
3.0
2.5
2.0
1.5
1.0
0.5
0.0
f1 (ppm)

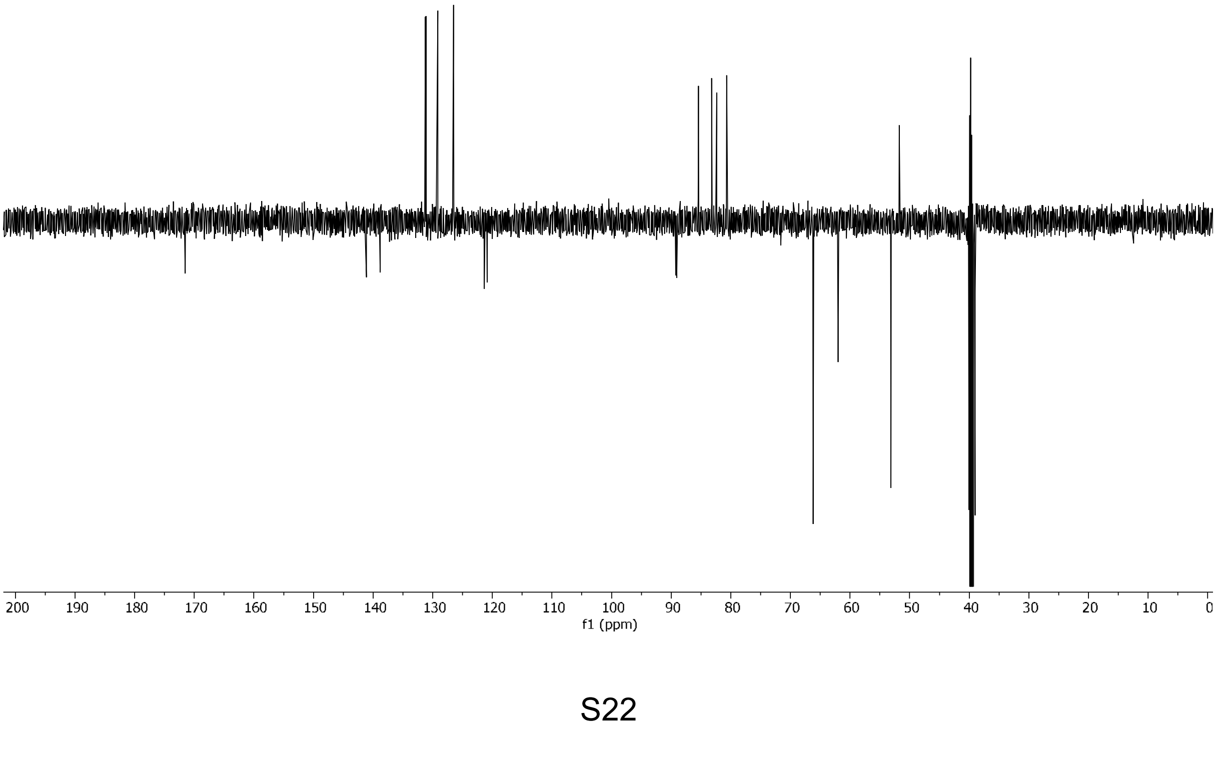

200
190
180
170
160
150
140
130
120
110
100
90
80
70
60
50
40
30
20
10
0
f1 (ppm)

Compound **31**

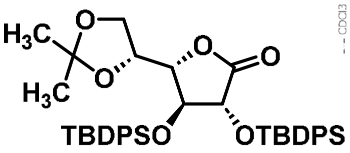
H3C
H3C
O
O
O
O
TBDPSO
OTBDPS

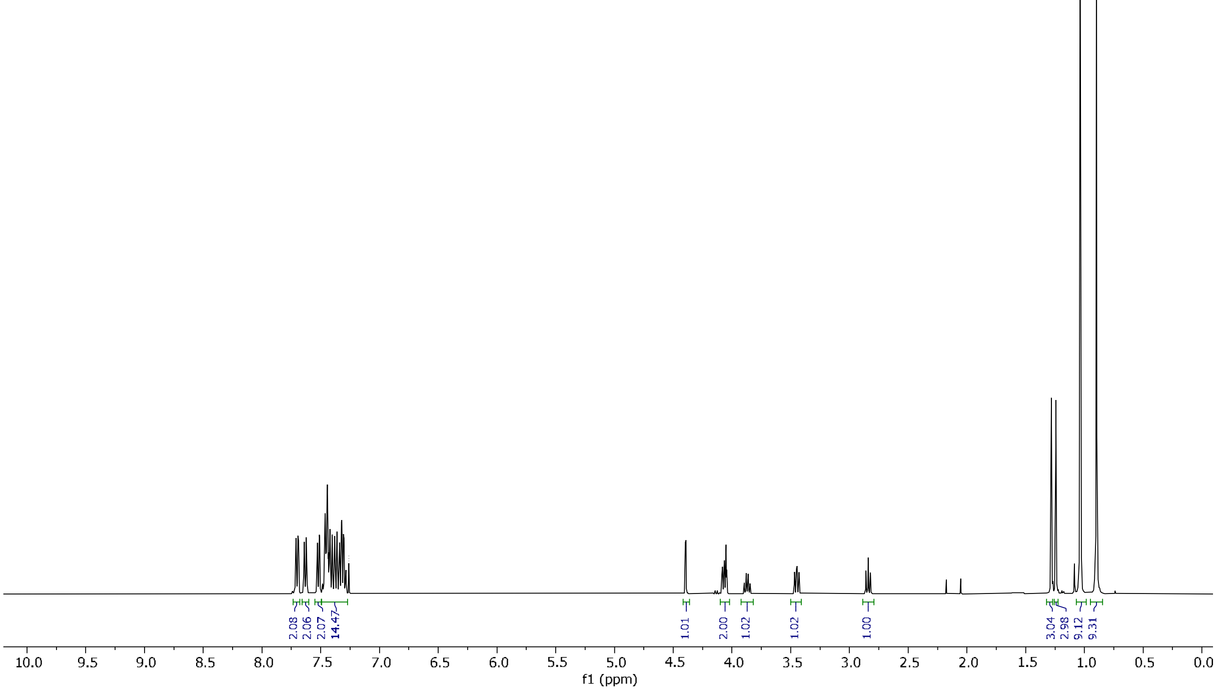
CDCl3
2.08
2.06
2.07
14.47
1.01
2.00
1.02
1.02
1.00
3.04
2.98
9.12
9.31
f1 (ppm)

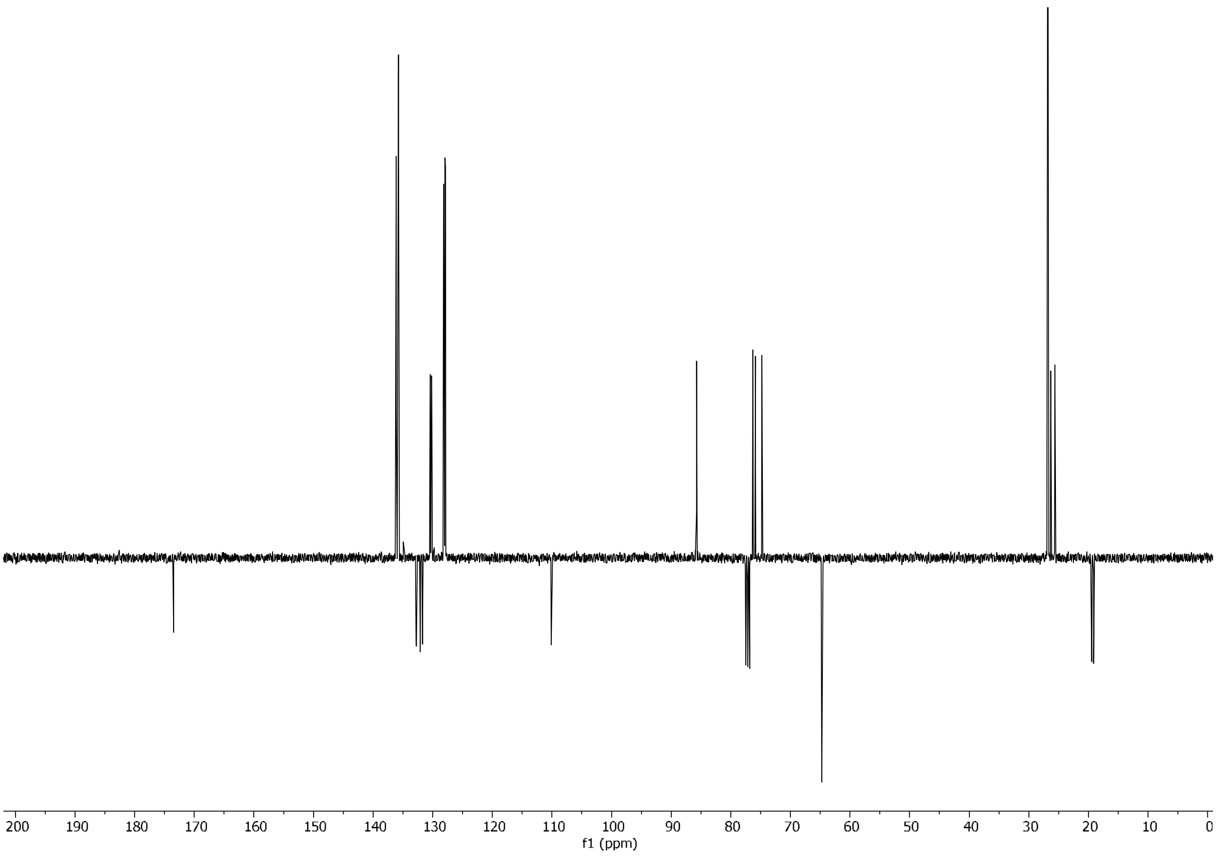
f1 (ppm)

Compound **32**

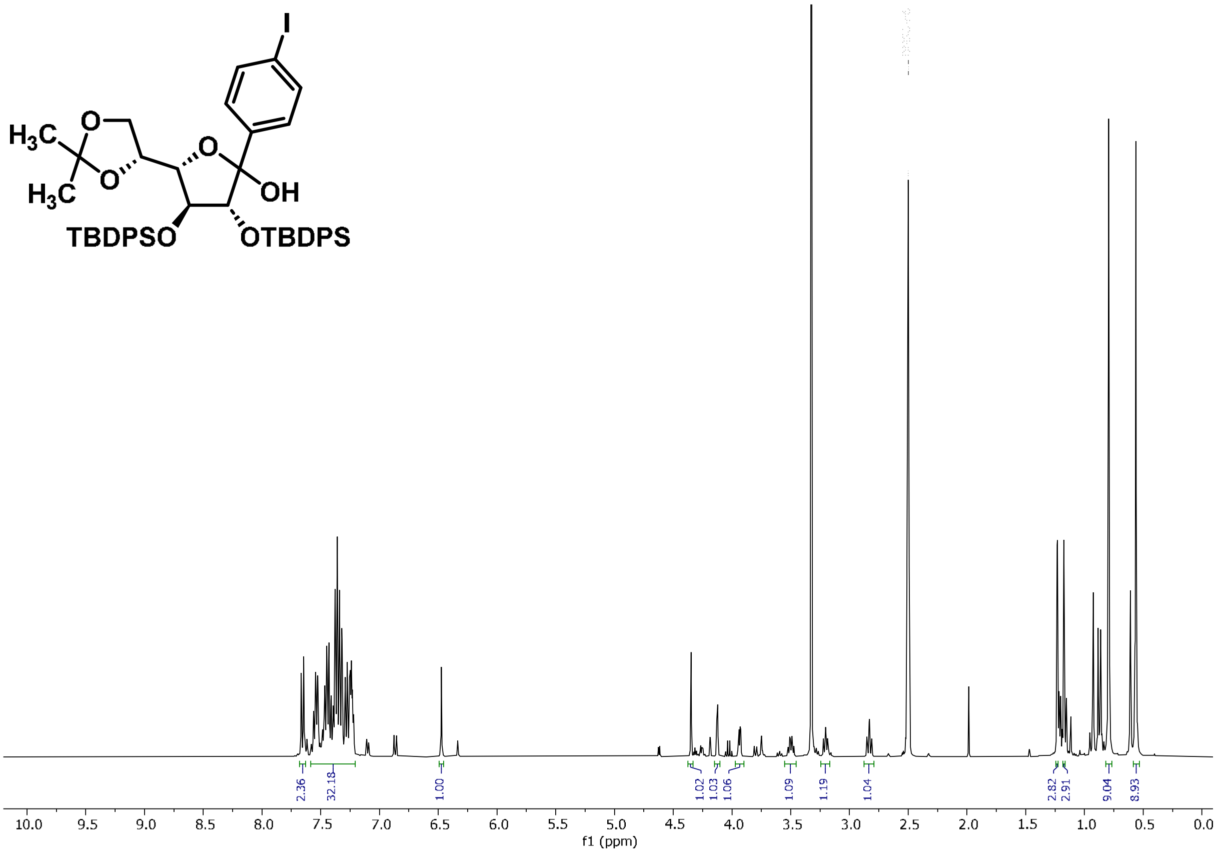

I
H3C
O
O
O
OH
H3C
TBDPSO
OTBDPS
2.36
32.18
1.00
1.02
1.03
1.06
1.09
1.19
1.04
2.82
2.91
9.04
8.93
10.0 9.5 9.0 8.5 8.0 7.5 7.0 6.5 6.0 5.5 5.0 4.5 4.0 3.5 3.0 2.5 2.0 1.5 1.0 0.5 0.0
f1 (ppm)

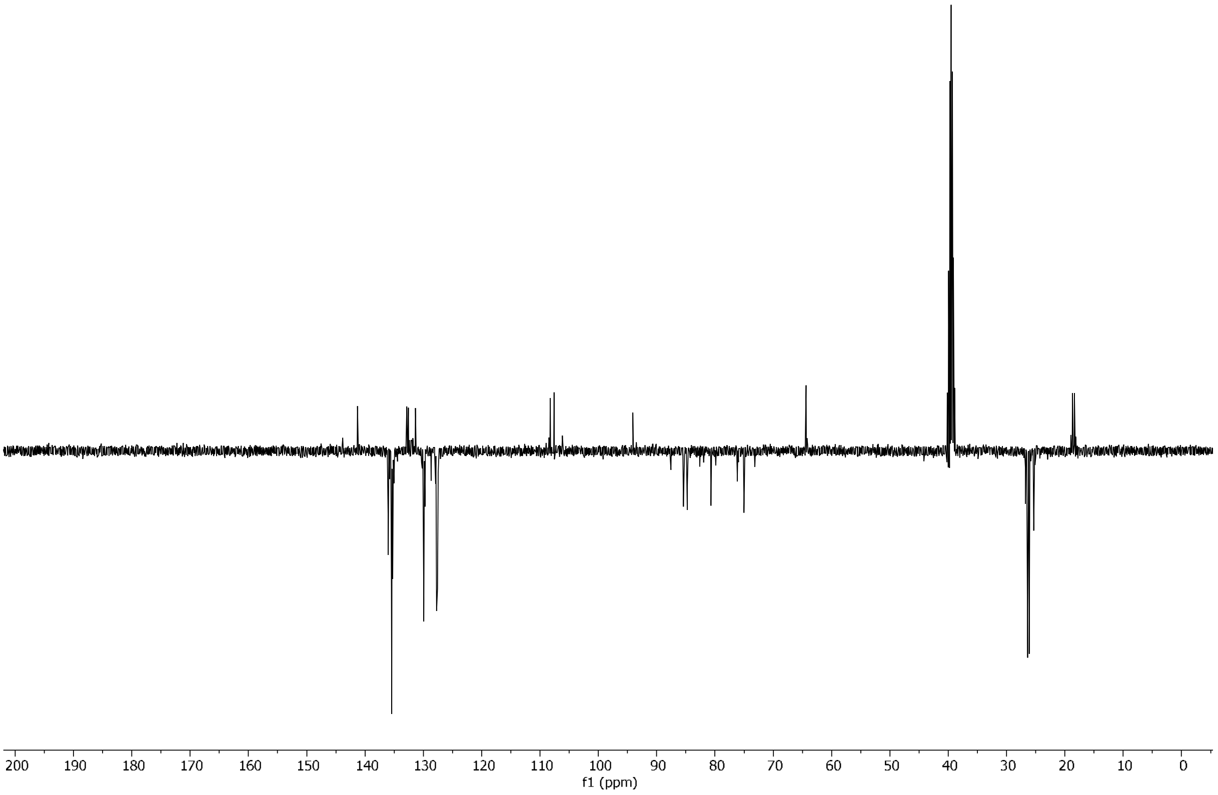

200 190 180 170 160 150 140 130 120 110 100 90 80 70 60 50 40 30 20 10 0
f1 (ppm)

Compound **33**

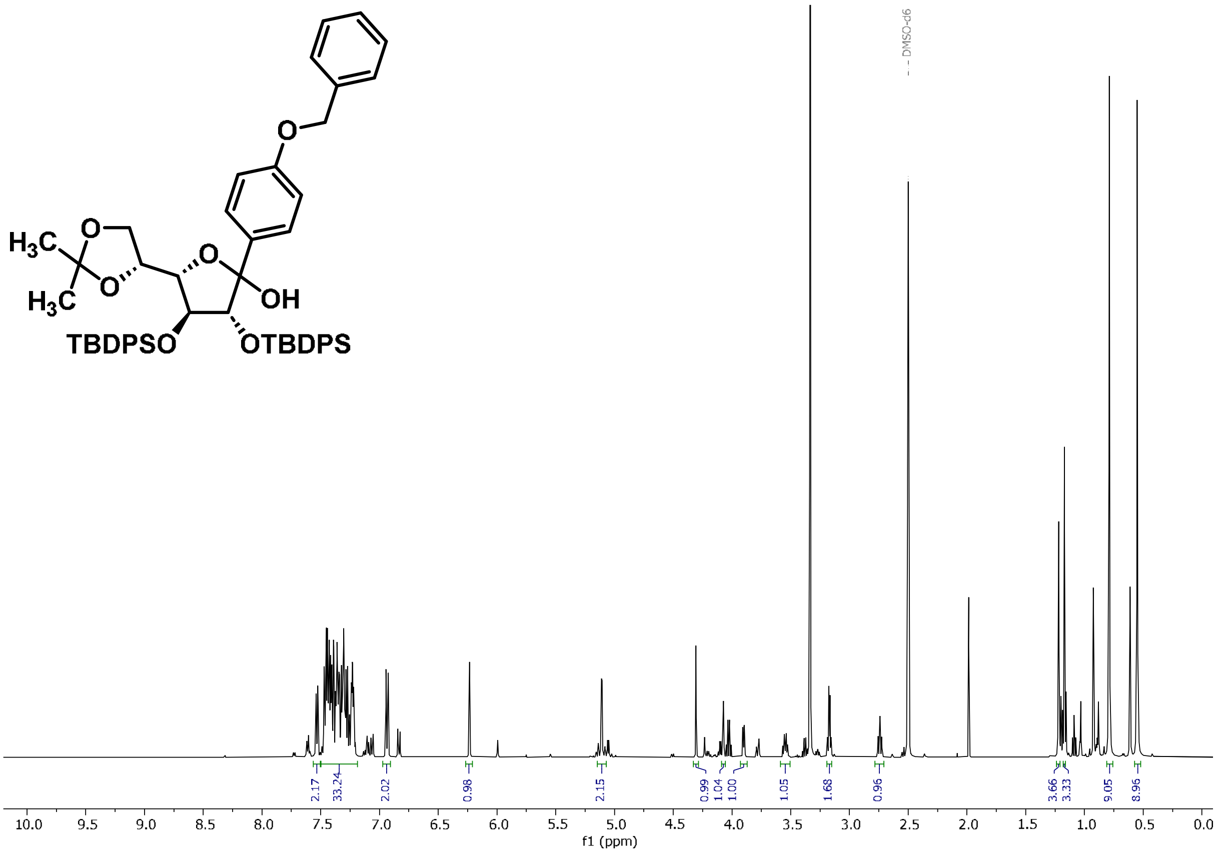
H3C
H3C
O
O
O
OH
O
TBDPSO
OTBDPS
DMSO-d6
2.17
33.24
2.02
0.98
2.15
0.99
1.04
1.00
1.05
1.68
0.96
3.66
3.33
9.05
8.96
10.0 9.5 9.0 8.5 8.0 7.5 7.0 6.5 6.0 5.5 5.0 4.5 4.0 3.5 3.0 2.5 2.0 1.5 1.0 0.5 0.0
f1 (ppm)

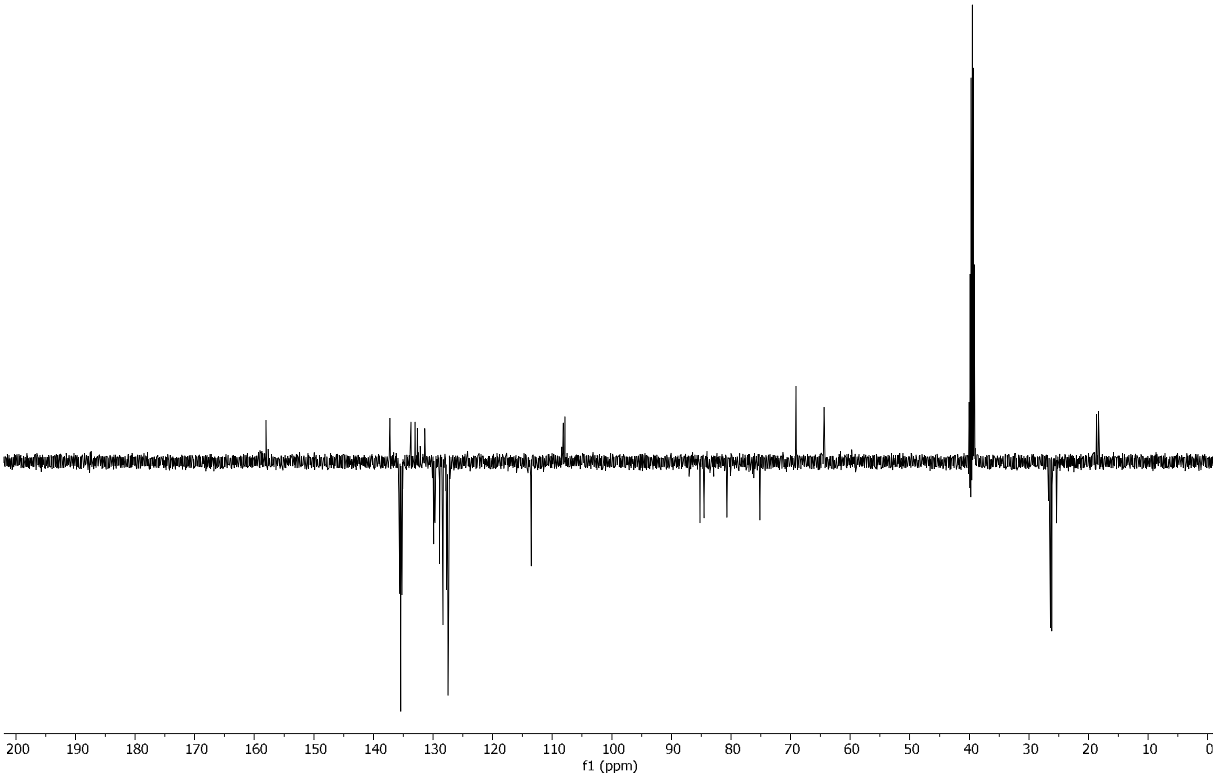
200 190 180 170 160 150 140 130 120 110 100 90 80 70 60 50 40 30 20 10 0
f1 (ppm)

Compound **34**

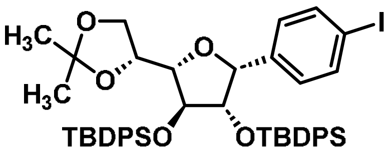

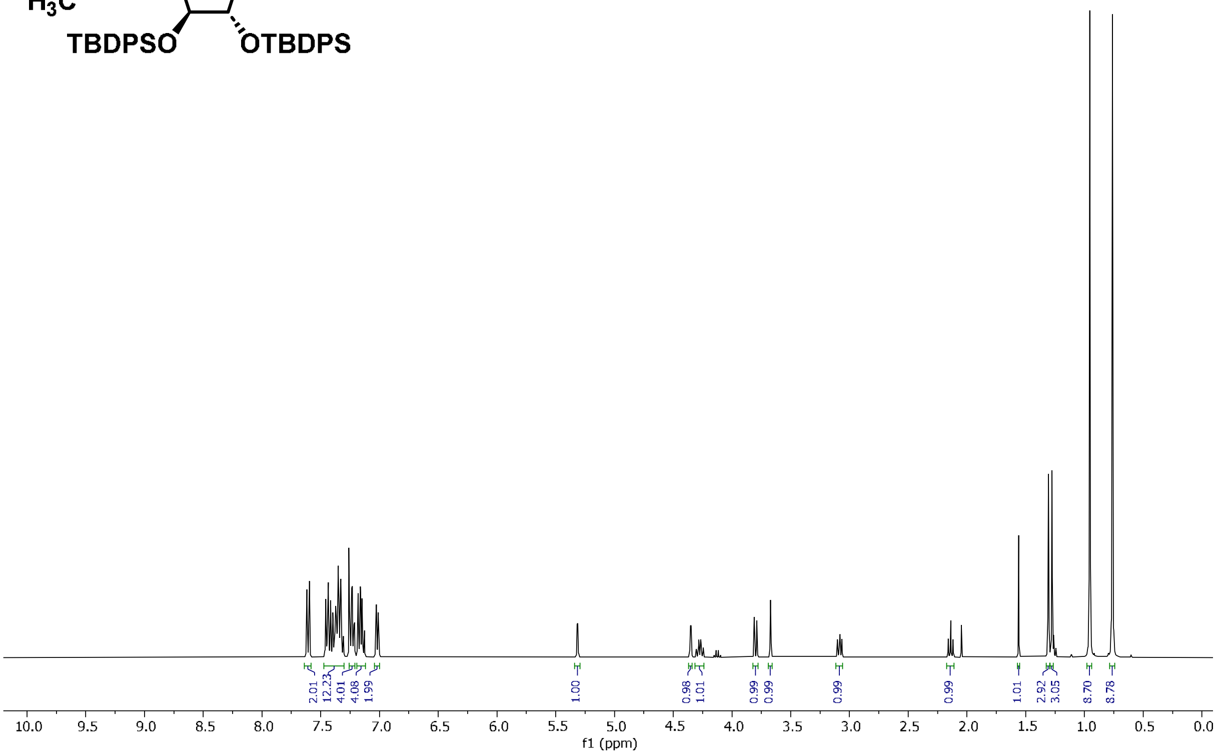
2.01
12.23
4.01
4.08
1.99
1.00
0.98
1.01
0.99
0.99
0.99
0.99
1.01
2.92
3.05
8.70
8.78
10.0
9.5
9.0
8.5
8.0
7.5
7.0
6.5
6.0
5.5
5.0
4.5
4.0
3.5
3.0
2.5
2.0
1.5
1.0
0.5
0.0
f1 (ppm)

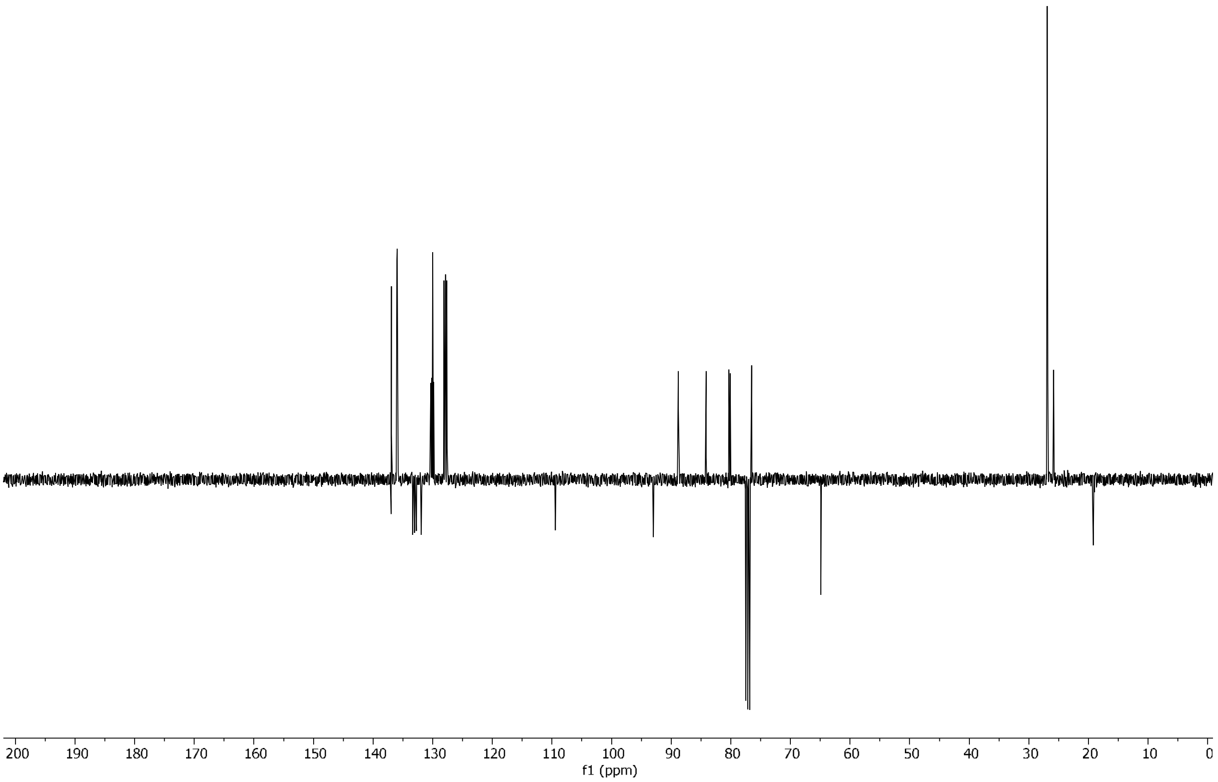
200
190
180
170
160
150
140
130
120
110
100
90
80
70
60
50
40
30
20
10
0
f1 (ppm)

Compound **35**

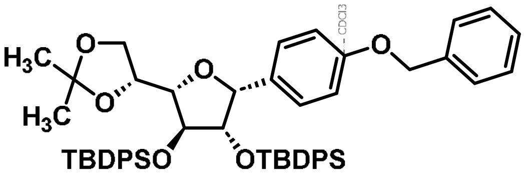
H3C
H3C
O
O
O
O
TBDPSO
OTBDPS

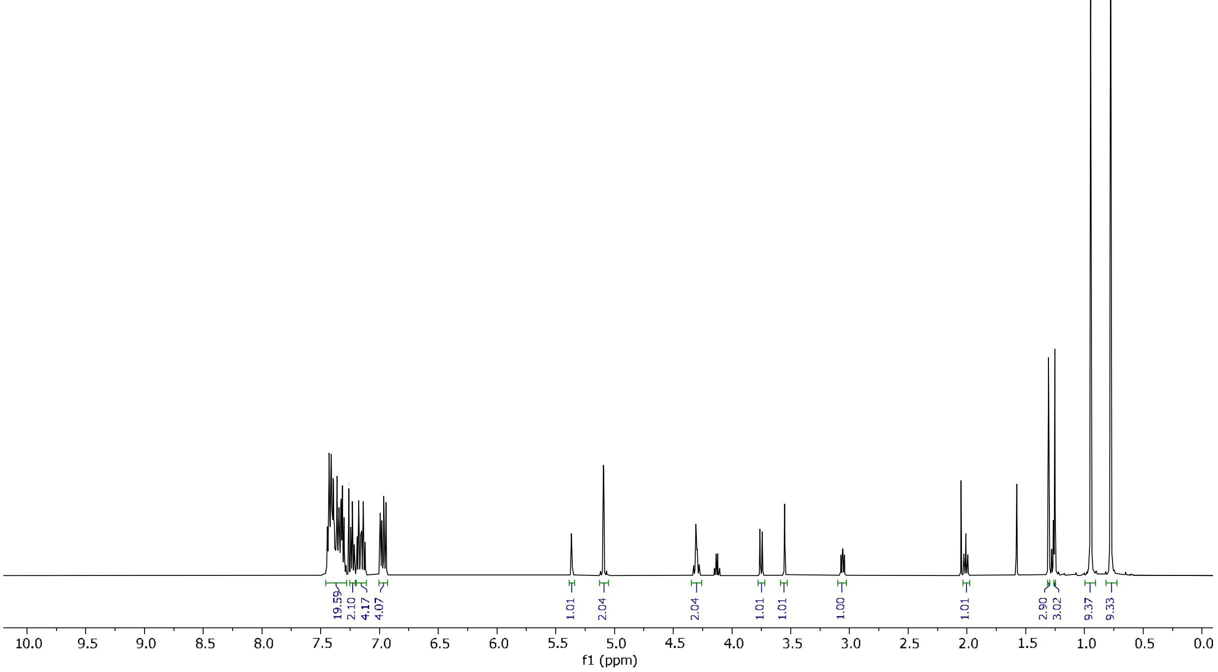
CDCl3
19.59
2.10
4.17
4.07
1.01
2.04
2.04
1.01
1.01
1.00
1.01
2.90
3.02
9.37
9.33
10.0 9.5 9.0 8.5 8.0 7.5 7.0 6.5 6.0 5.5 5.0 4.5 4.0 3.5 3.0 2.5 2.0 1.5 1.0 0.5 0.0
f1 (ppm)

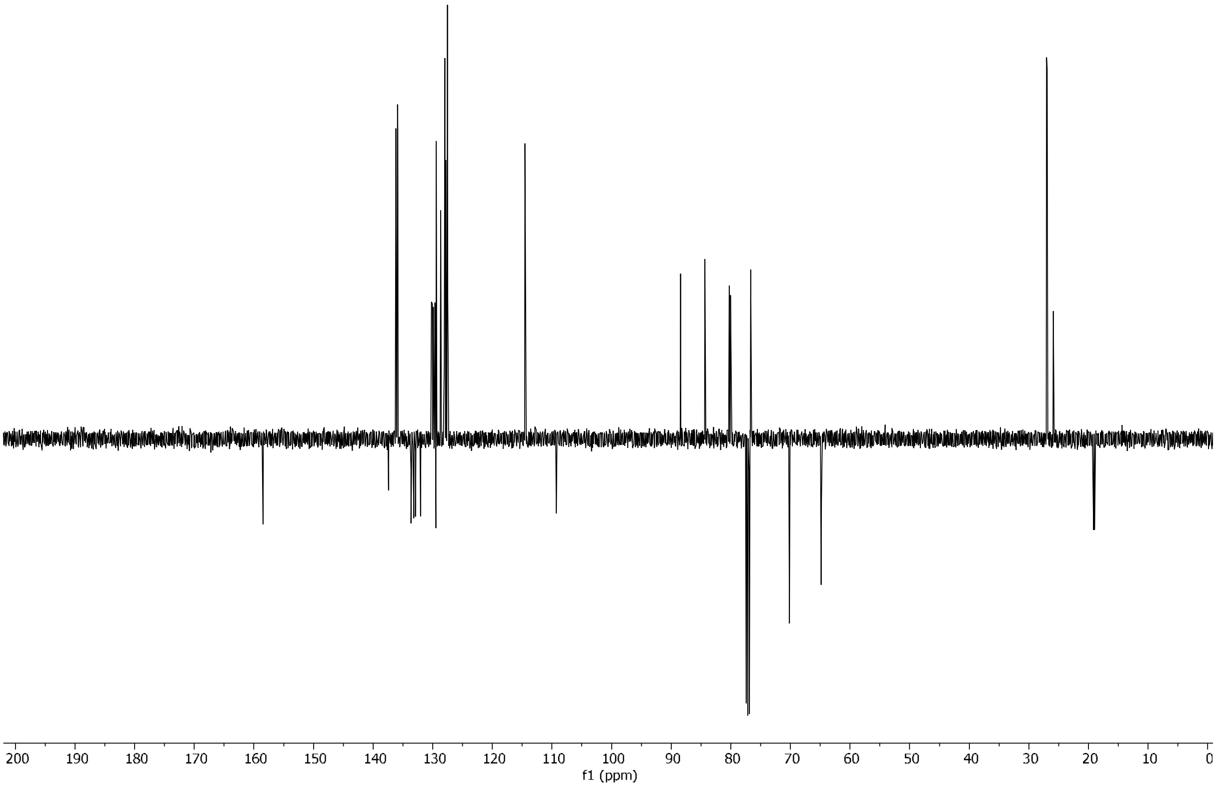
200 190 180 170 160 150 140 130 120 110 100 90 80 70 60 50 40 30 20 10 0
f1 (ppm)

Compound **36**

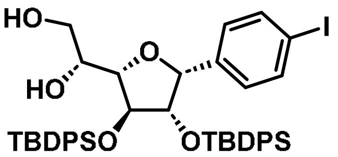

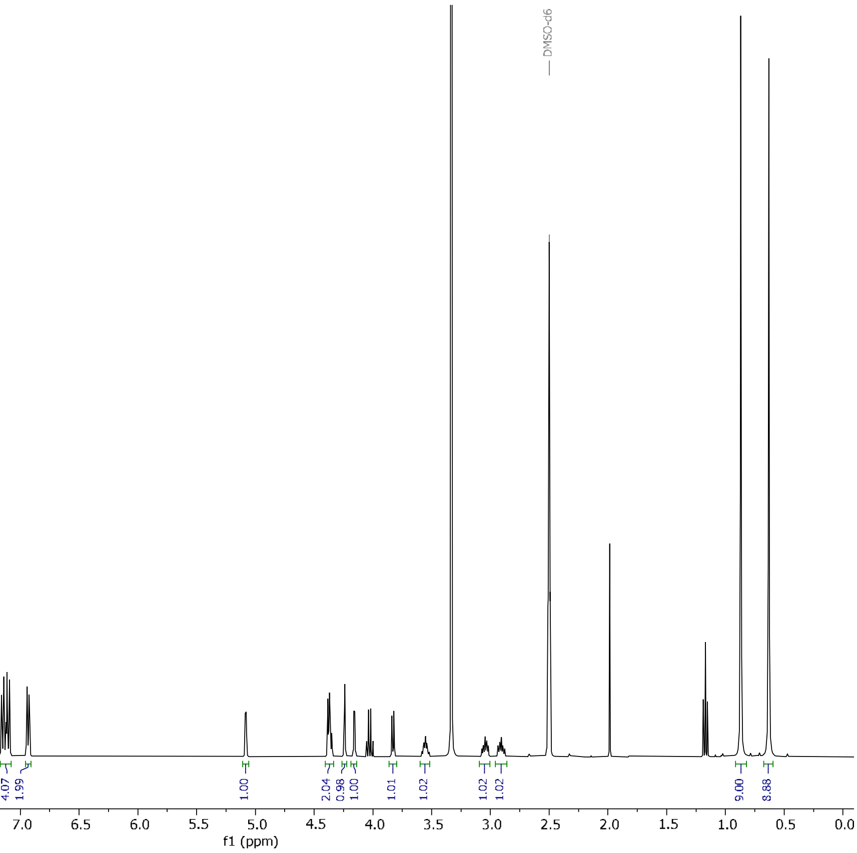

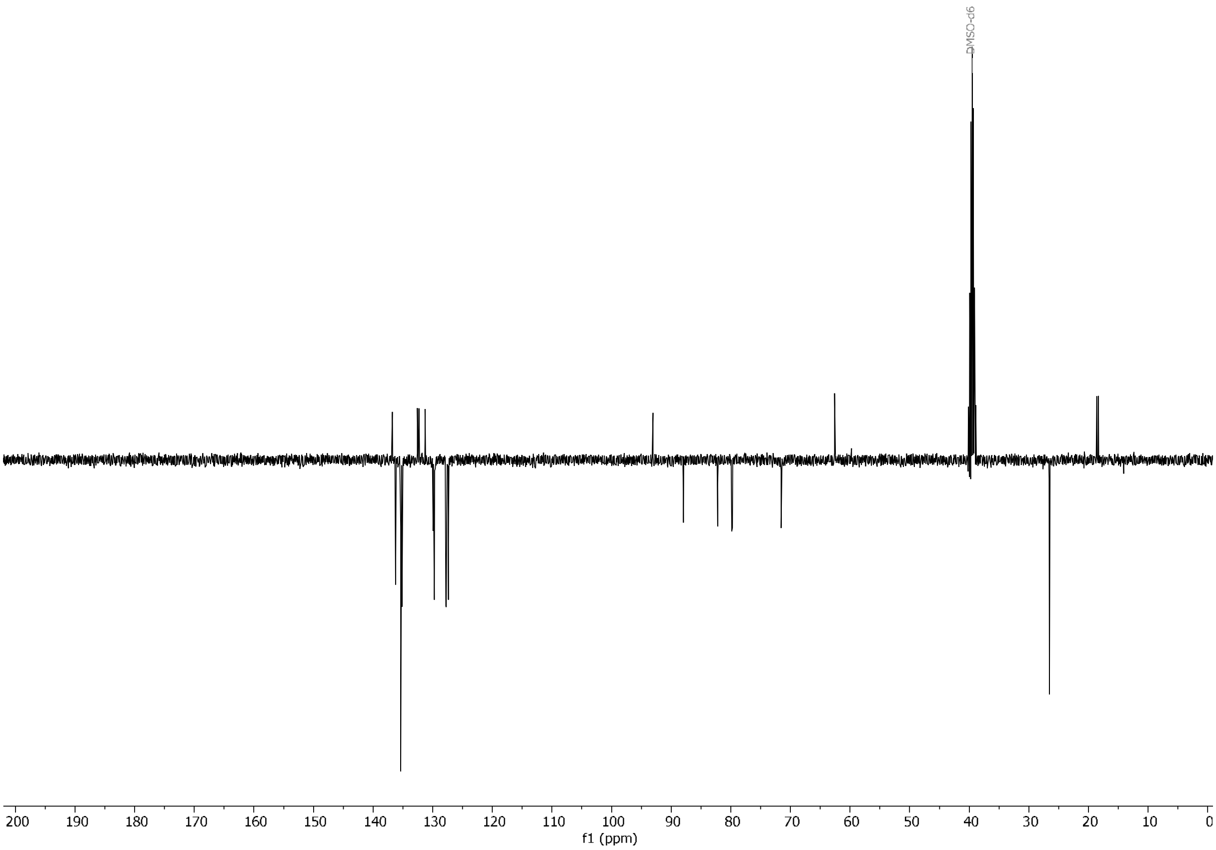

Compound **37**

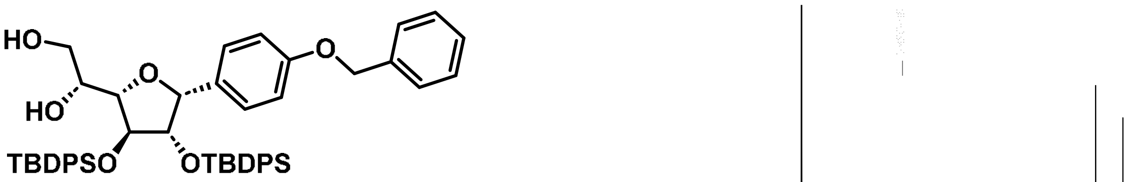

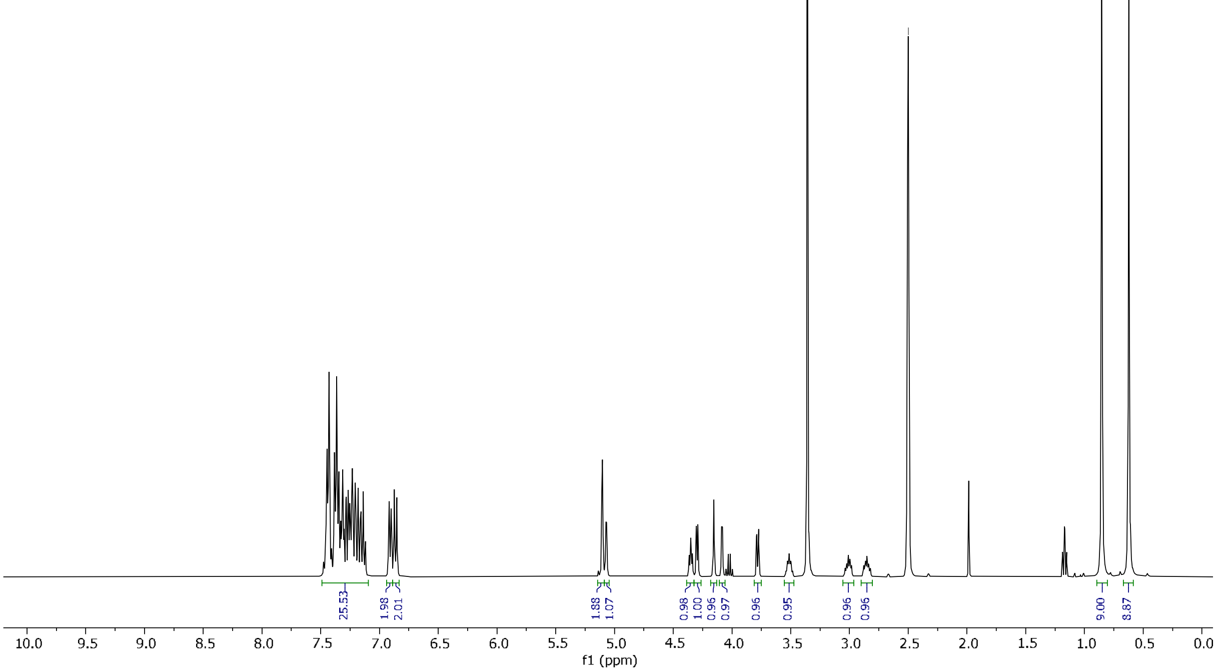

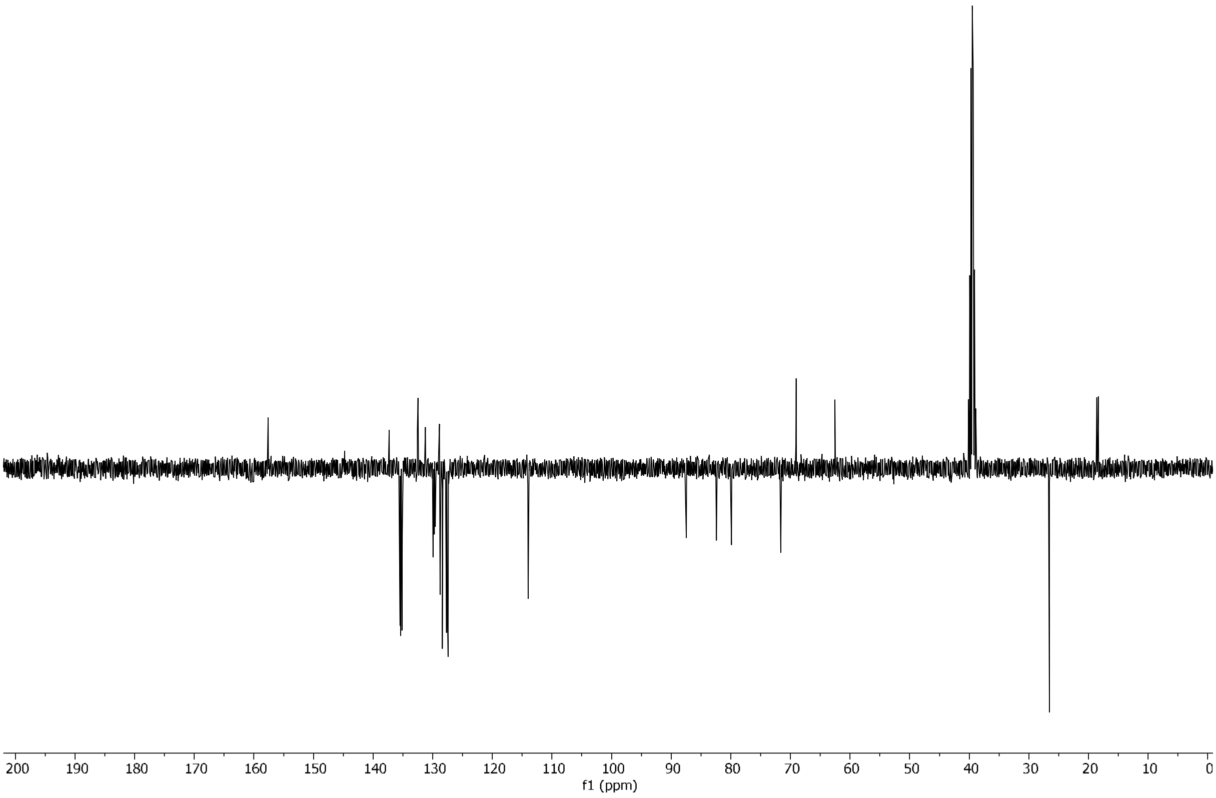

Compound **38**

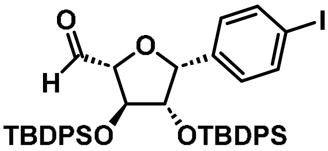
O
H
I
TBDPSO
OTBDPS

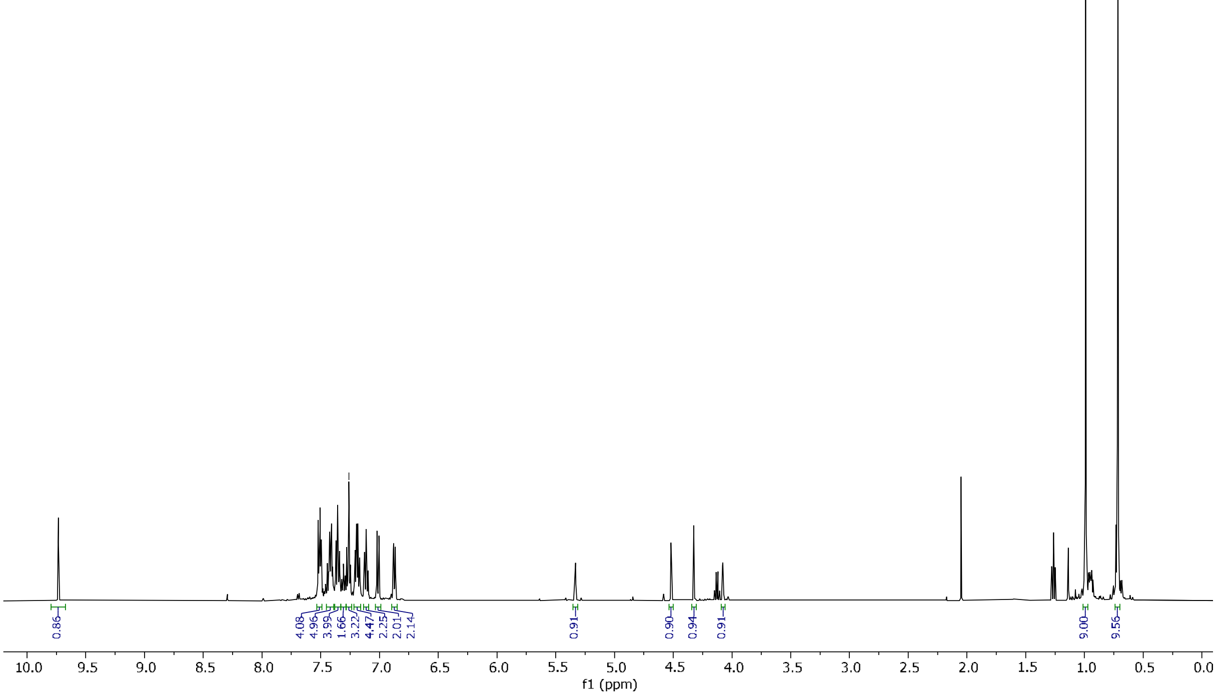
CDCl3
0.86
4.08
4.96
3.99
1.66
3.22
4.47
2.25
2.01
2.14
0.91
0.90
0.94
0.91
9.00
9.56
10.0 9.5 9.0 8.5 8.0 7.5 7.0 6.5 6.0 5.5 5.0 4.5 4.0 3.5 3.0 2.5 2.0 1.5 1.0 0.5 0.0
f1 (ppm)

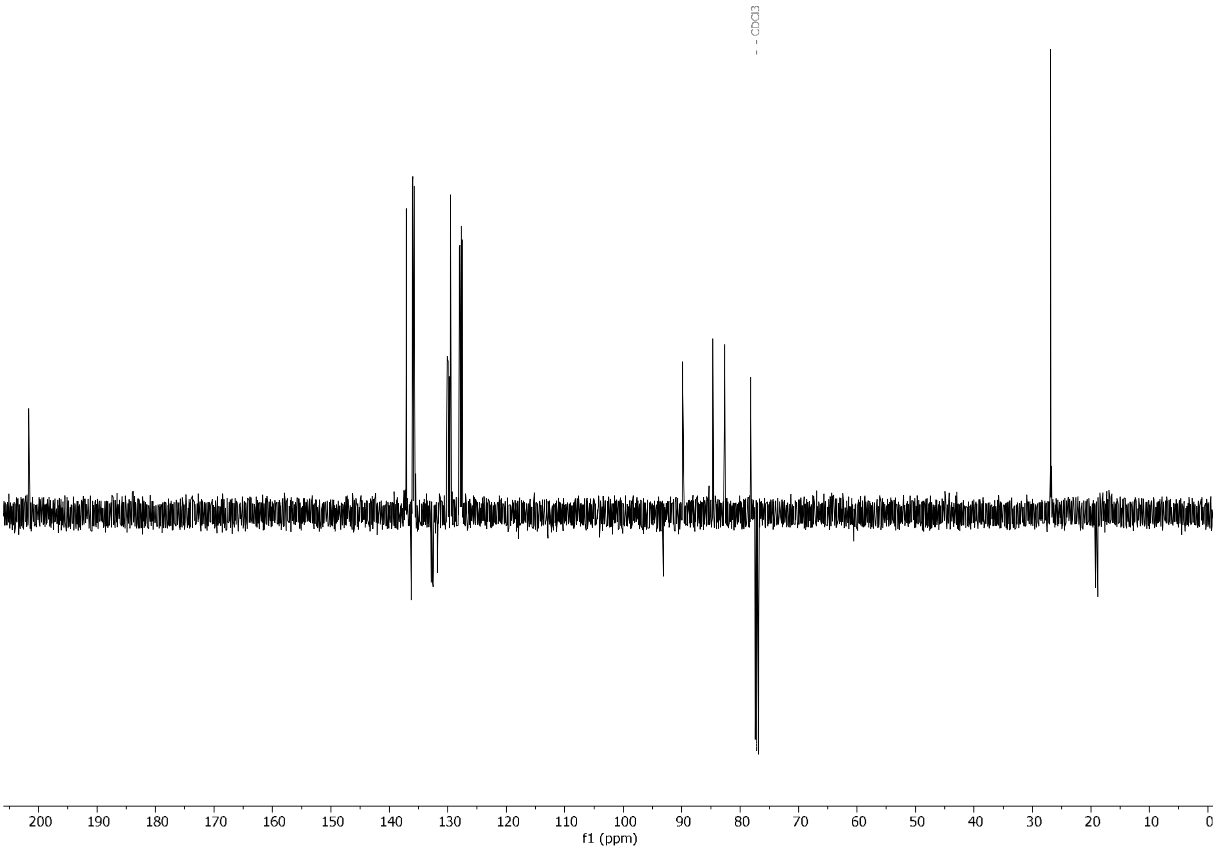
CDCl3
200 190 180 170 160 150 140 130 120 110 100 90 80 70 60 50 40 30 20 10 0
f1 (ppm)

Compound **39**

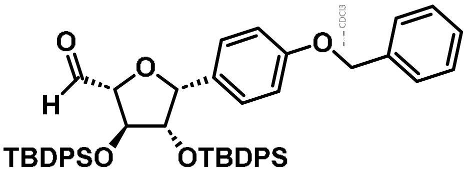
O
H
O
O
TBDPSO
OTBDPS

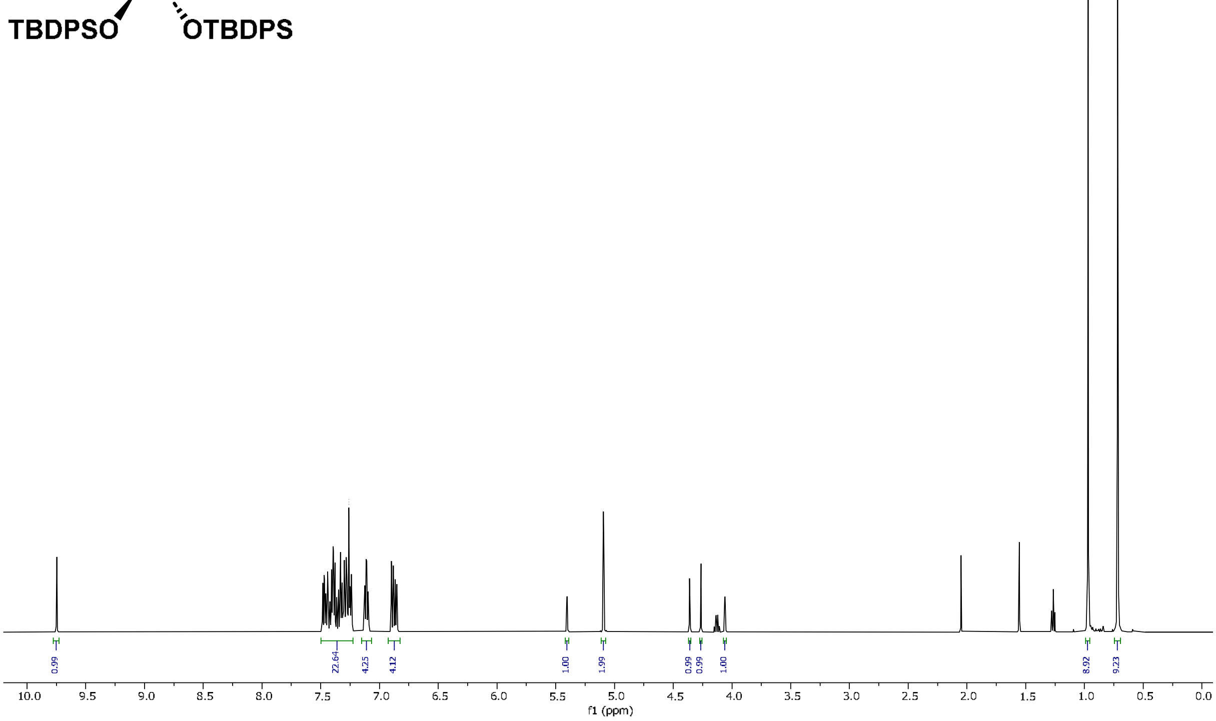
0.99
22.64
4.25
4.12
1.00
1.99
0.99
0.99
1.00
8.92
9.23
10.0 9.5 9.0 8.5 8.0 7.5 7.0 6.5 6.0 5.5 5.0 4.5 4.0 3.5 3.0 2.5 2.0 1.5 1.0 0.5 0.0
f1 (ppm)

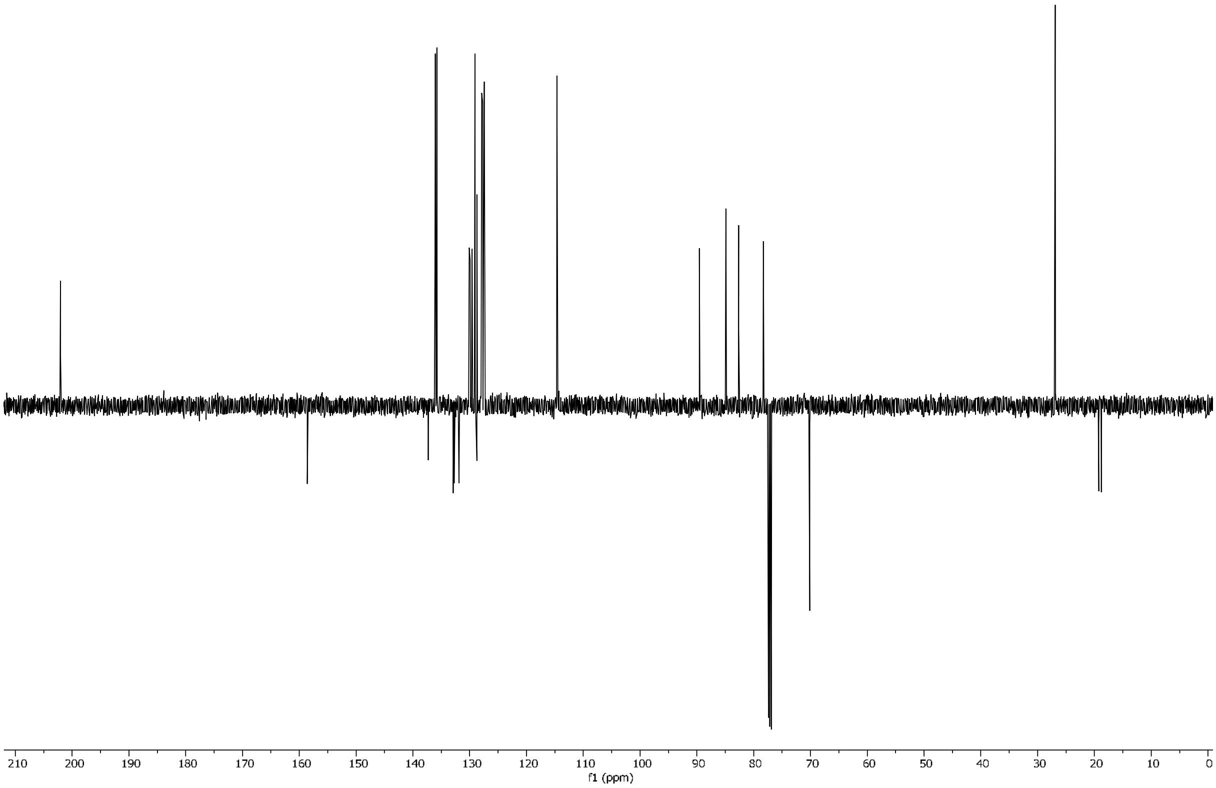
210 200 190 180 170 160 150 140 130 120 110 100 90 80 70 60 50 40 30 20 10 0
f1 (ppm)

Compound **40**

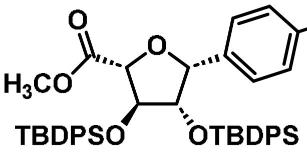
O
O
H3CO
TBDPSO
OTBDPS
I

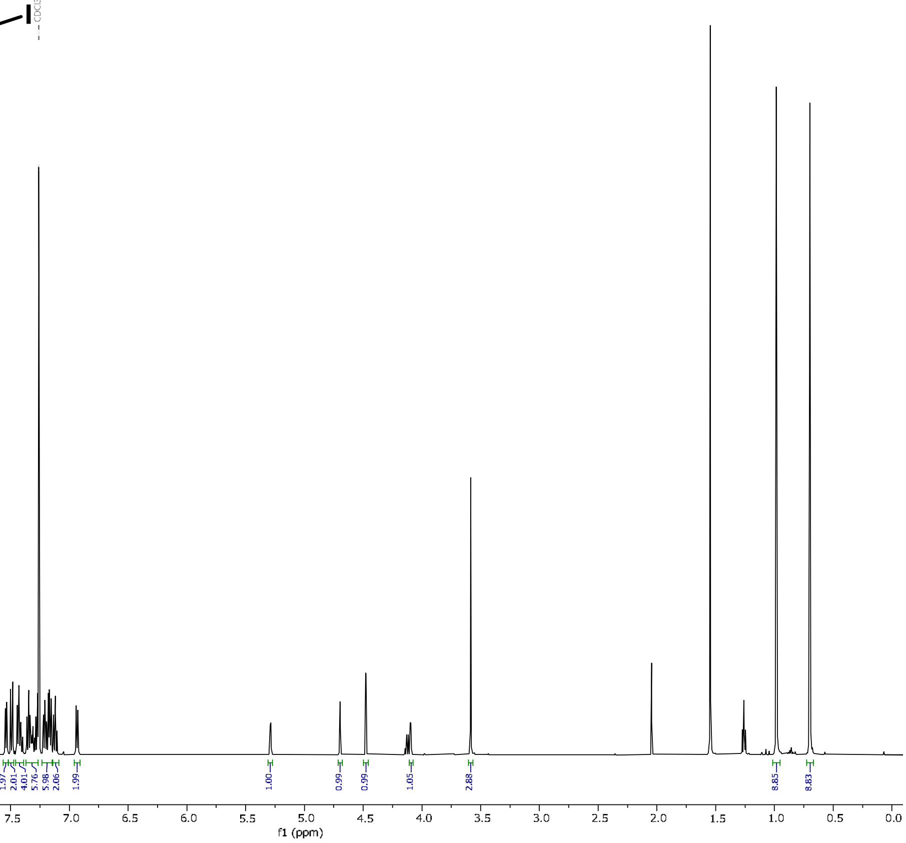
CDCl3
1.97
2.01
4.01
5.76
5.98
2.06
1.99
1.00
0.99
0.99
1.05
2.88
8.85
8.83
10.0 9.5 9.0 8.5 8.0 7.5 7.0 6.5 6.0 5.5 5.0 4.5 4.0 3.5 3.0 2.5 2.0 1.5 1.0 0.5 0.0
f1 (ppm)

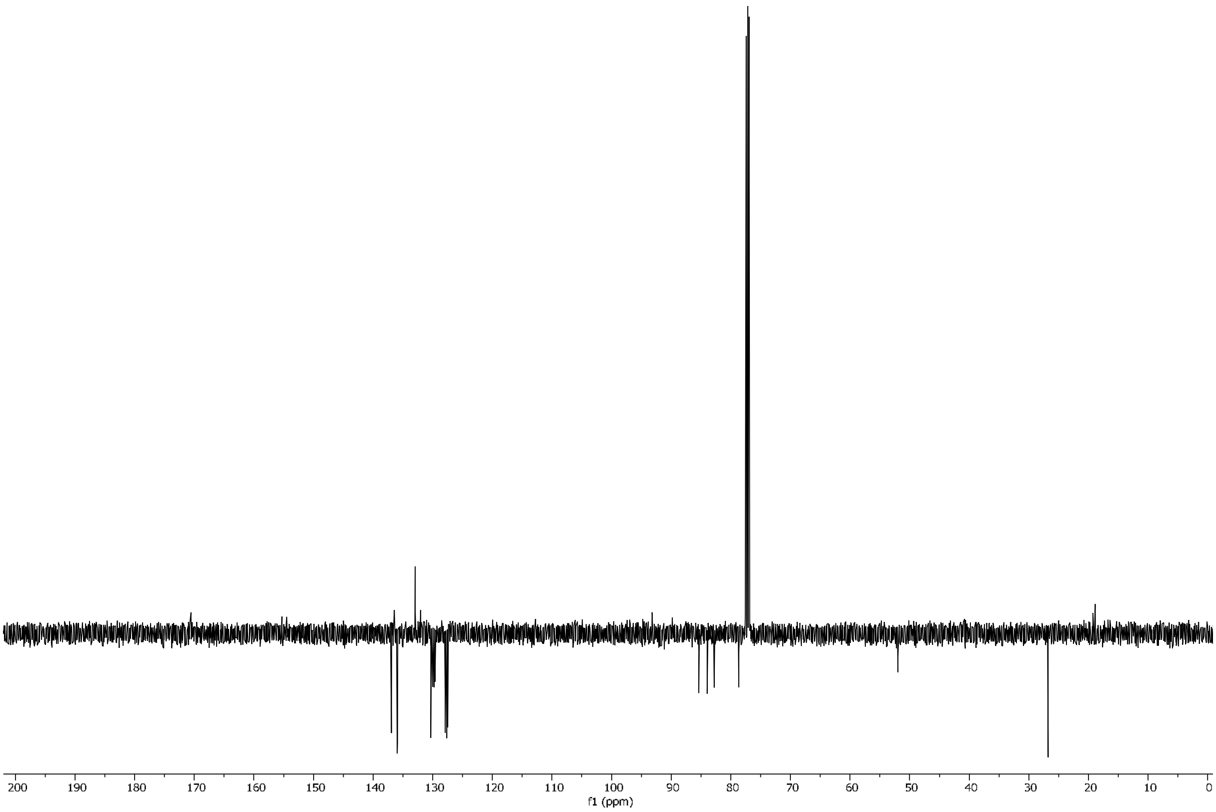
200 190 180 170 160 150 140 130 120 110 100 90 80 70 60 50 40 30 20 10 0
f1 (ppm)

Compound **41**

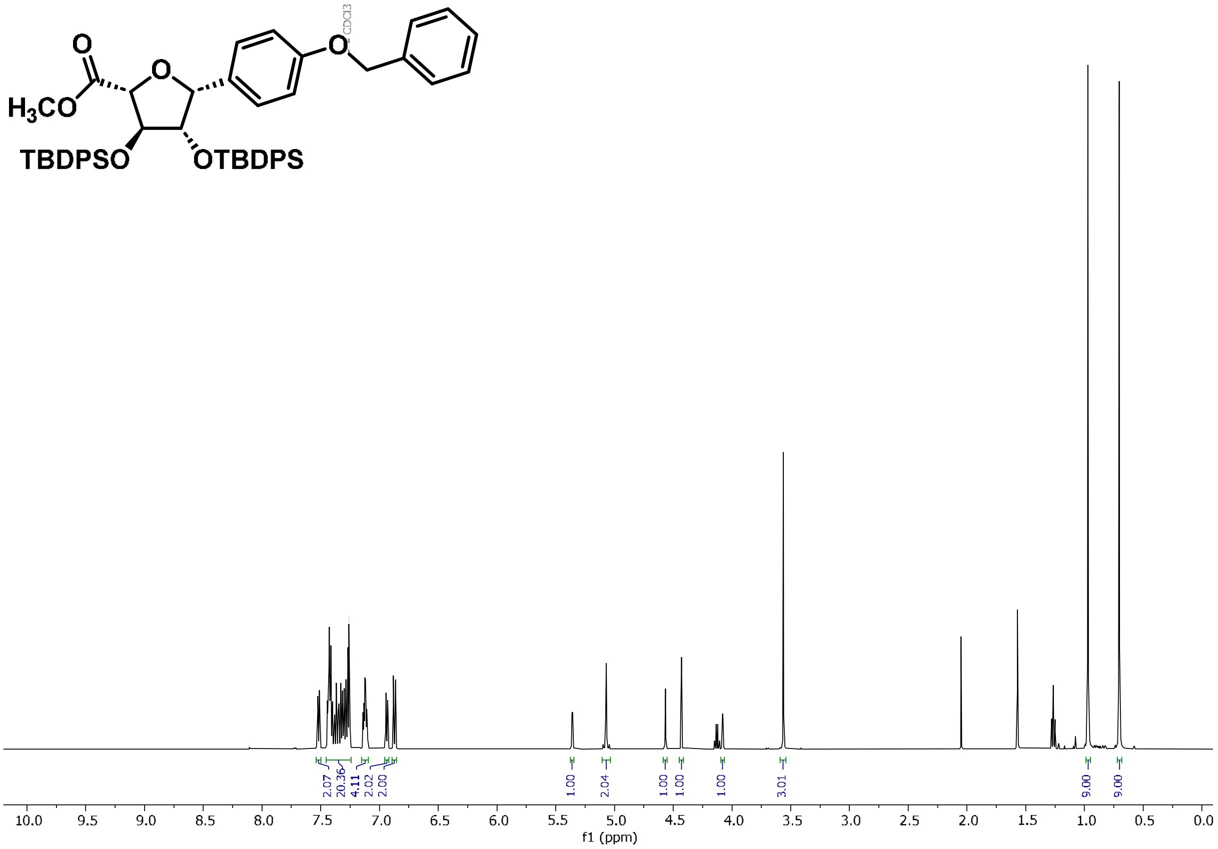
O
H3CO
O
TBDPSO
OTBDPS
O
CDCl3
2.07
20.36
4.11
2.02
2.00
1.00
2.04
1.00
1.00
1.00
3.01
9.00
9.00
10.0 9.5 9.0 8.5 8.0 7.5 7.0 6.5 6.0 5.5 5.0 4.5 4.0 3.5 3.0 2.5 2.0 1.5 1.0 0.5 0.0
f1 (ppm)

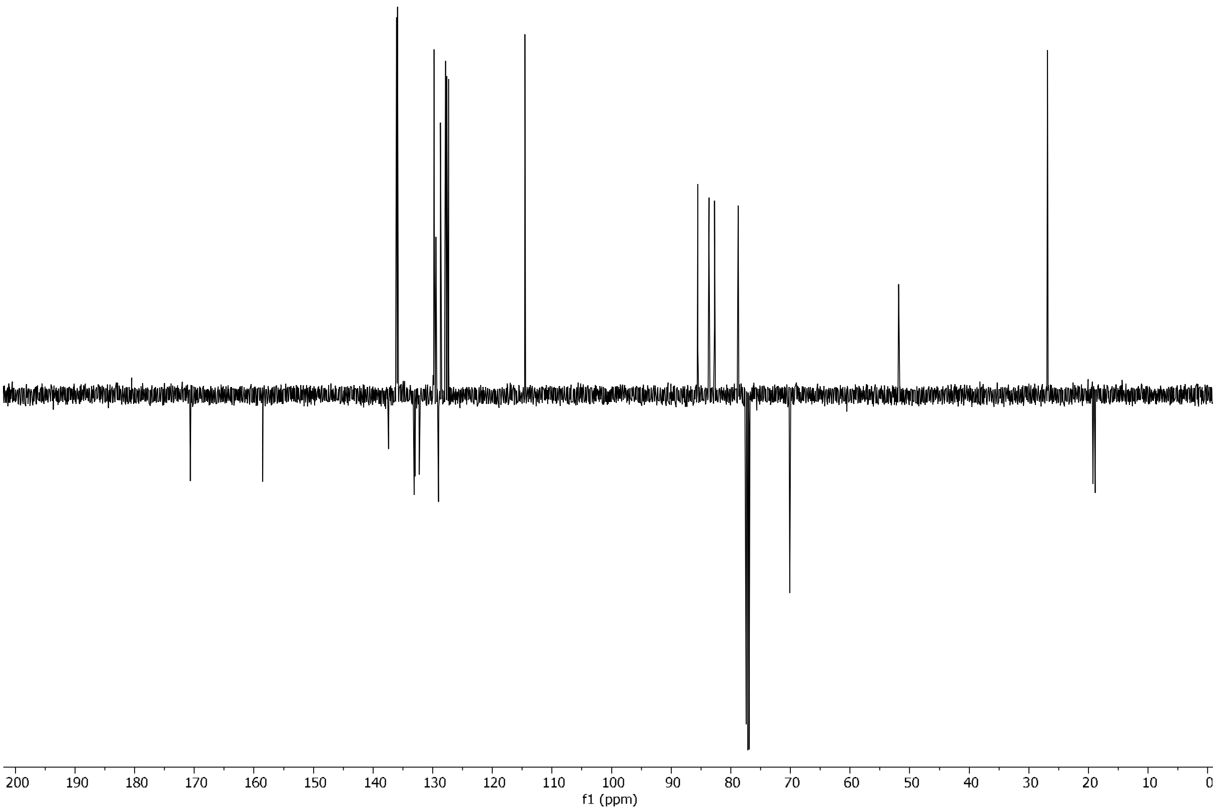
200 190 180 170 160 150 140 130 120 110 100 90 80 70 60 50 40 30 20 10 0
f1 (ppm)

Compound **42**

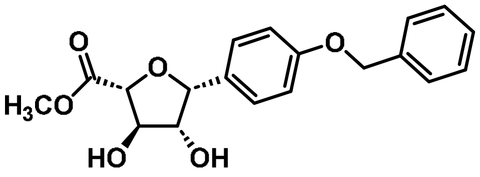
O
H3CO
O
HO
OH
O

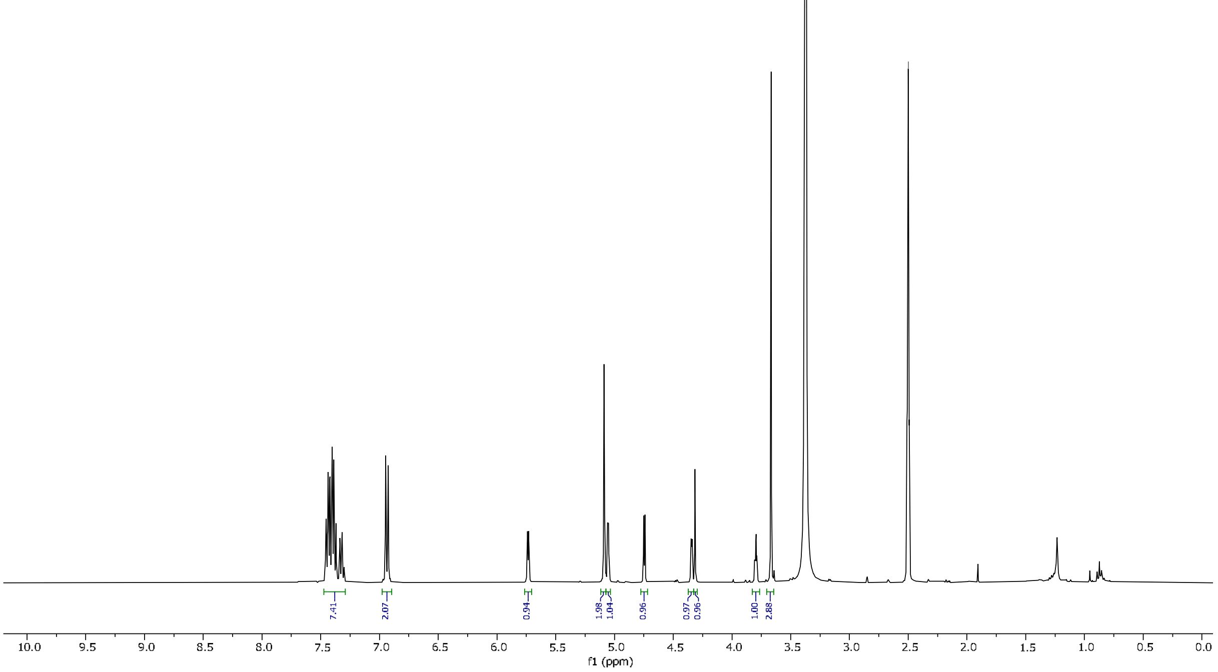
7.41
2.07
0.94
1.98
1.04
0.96
0.97
0.96
1.00
2.88
10.0 9.5 9.0 8.5 8.0 7.5 7.0 6.5 6.0 5.5 5.0 4.5 4.0 3.5 3.0 2.5 2.0 1.5 1.0 0.5 0.0
f1 (ppm)

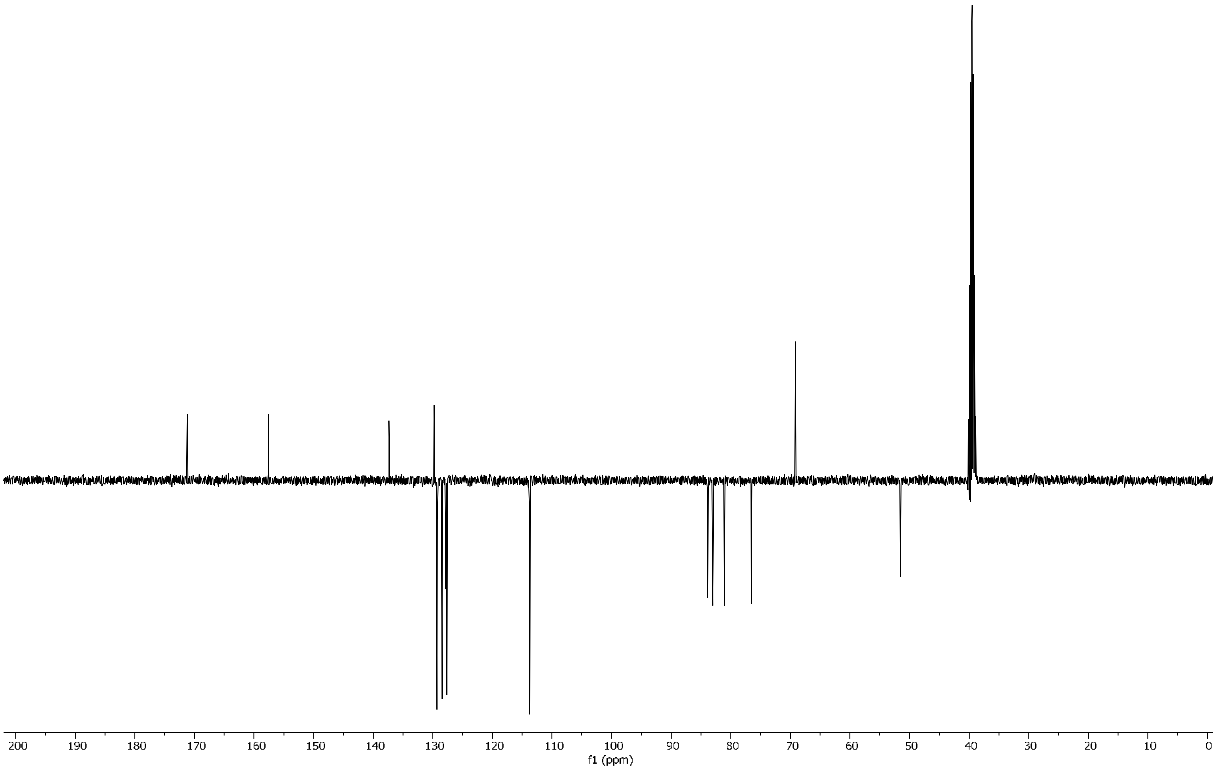
200 190 180 170 160 150 140 130 120 110 100 90 80 70 60 50 40 30 20 10 0
f1 (ppm)

Compound **43**

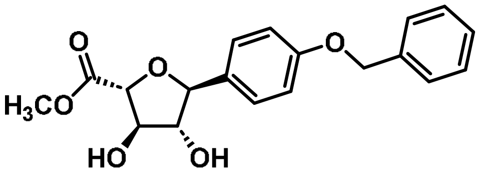

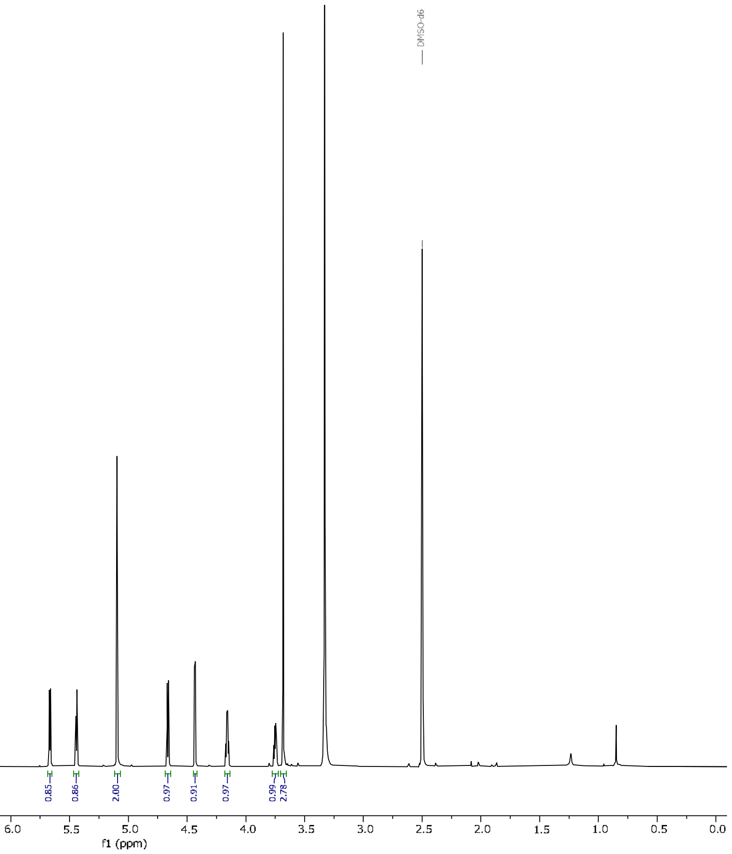
DMSO-d6
1.92
2.00
2.93
1.89
0.85
0.86
2.00
0.97
0.91
0.97
0.99
2.78
10.0
9.5
9.0
8.5
8.0
7.5
7.0
6.5
6.0
5.5
5.0
4.5
4.0
3.5
3.0
2.5
2.0
1.5
1.0
0.5
0.0
f1 (ppm)

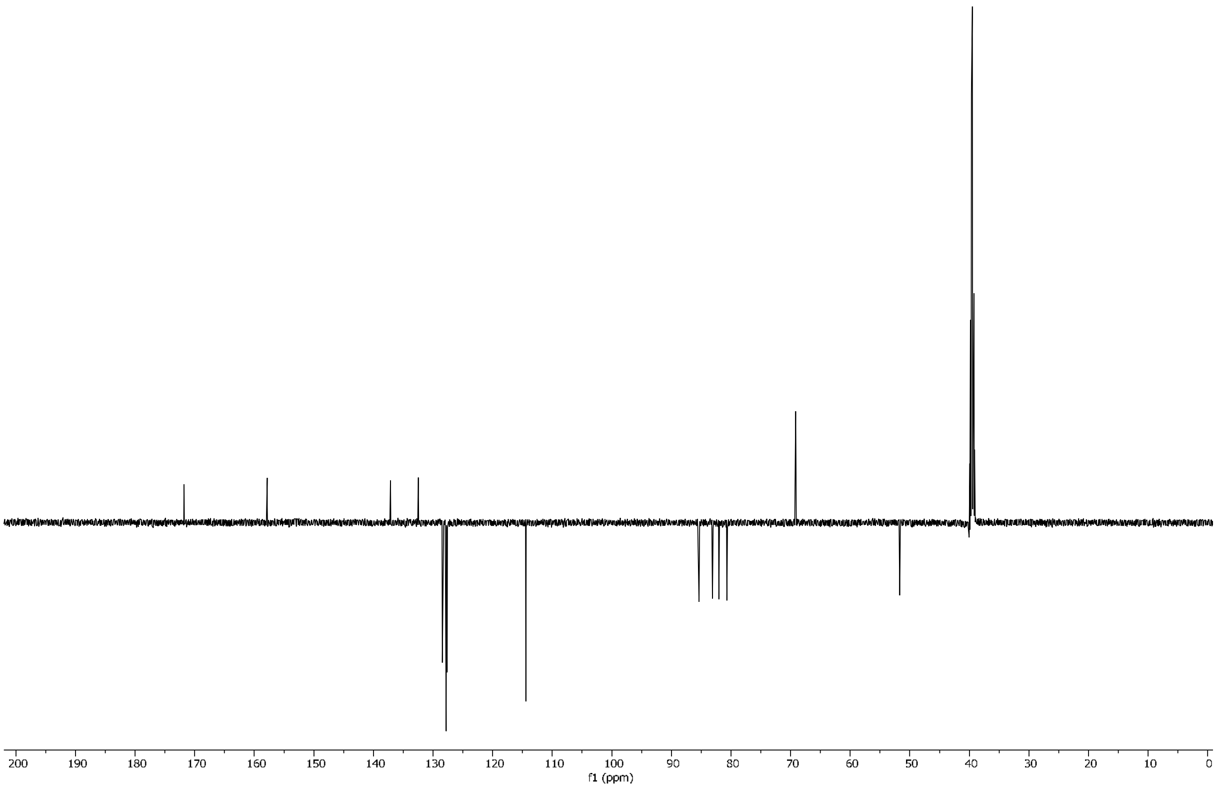
200
190
180
170
160
150
140
130
120
110
100
90
80
70
60
50
40
30
20
10
0
f1 (ppm)

Compound **44**

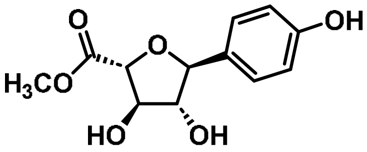

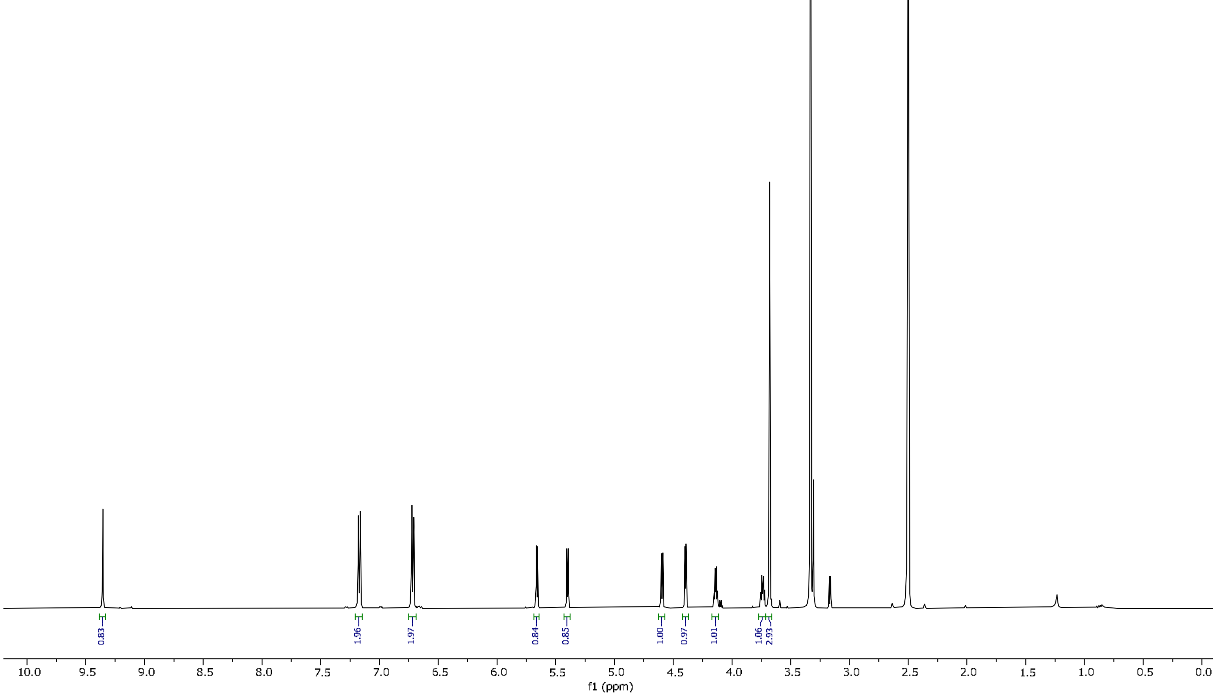

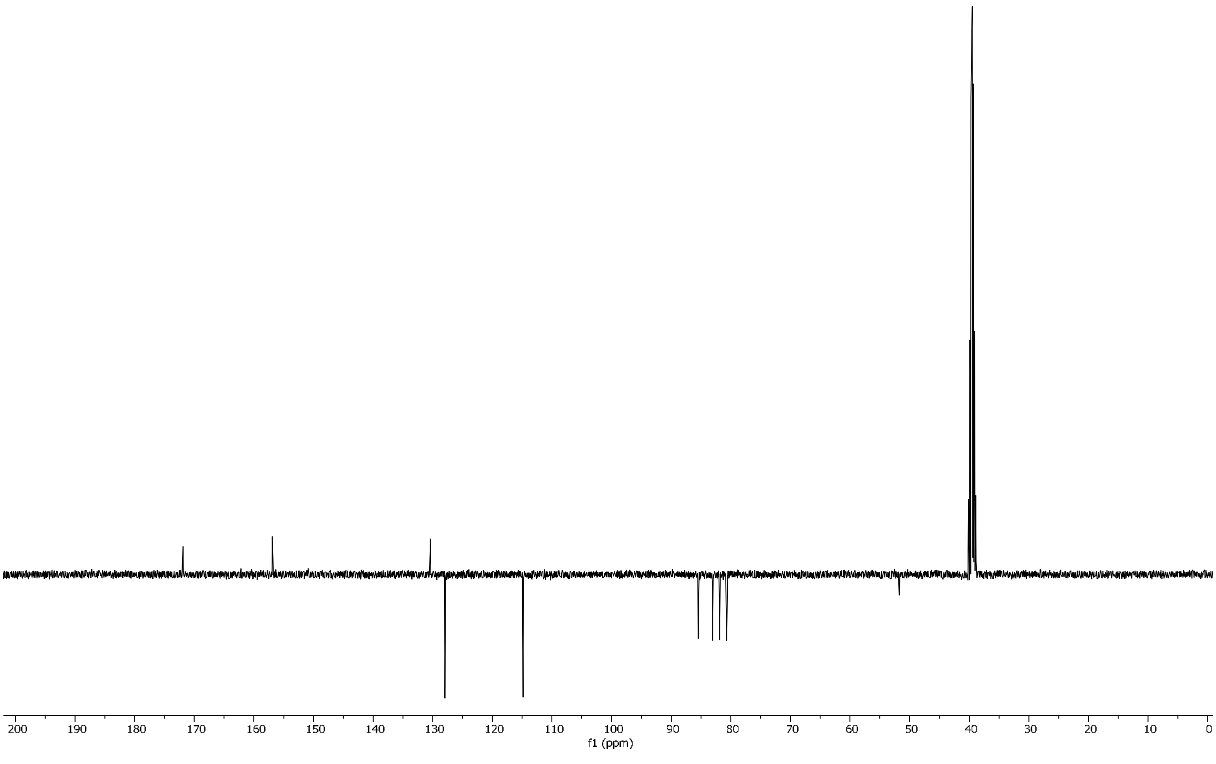

Compound **45**

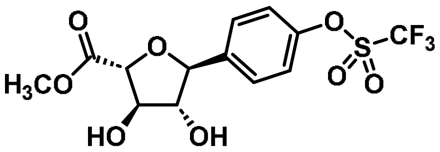
O
O
H3CO
HO
OH
O
S
CF3
O
O

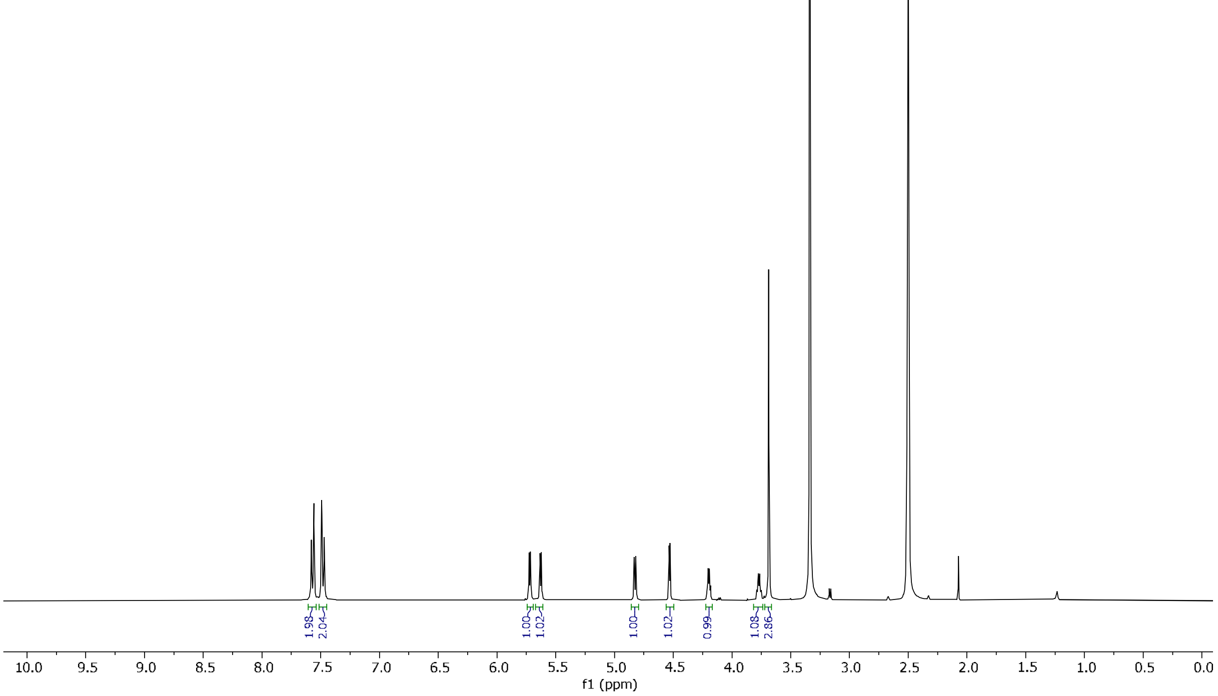
DMSO-d6
1.98
2.04
1.00
1.02
1.00
1.02
0.99
1.08
2.86
10.0 9.5 9.0 8.5 8.0 7.5 7.0 6.5 6.0 5.5 5.0 4.5 4.0 3.5 3.0 2.5 2.0 1.5 1.0 0.5 0.0
f1 (ppm)

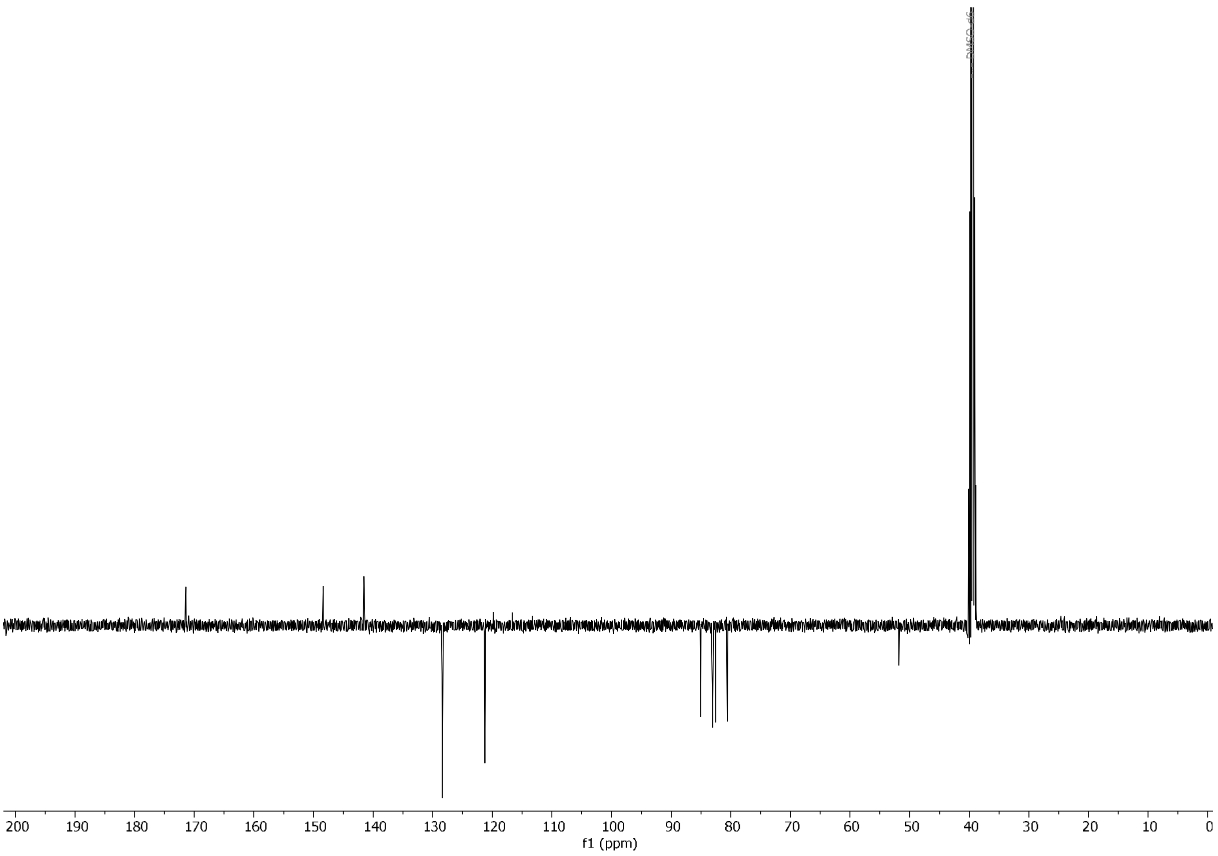
200 190 180 170 160 150 140 130 120 110 100 90 80 70 60 50 40 30 20 10 0
f1 (ppm)

HPLC traces of the synthesized compounds

15

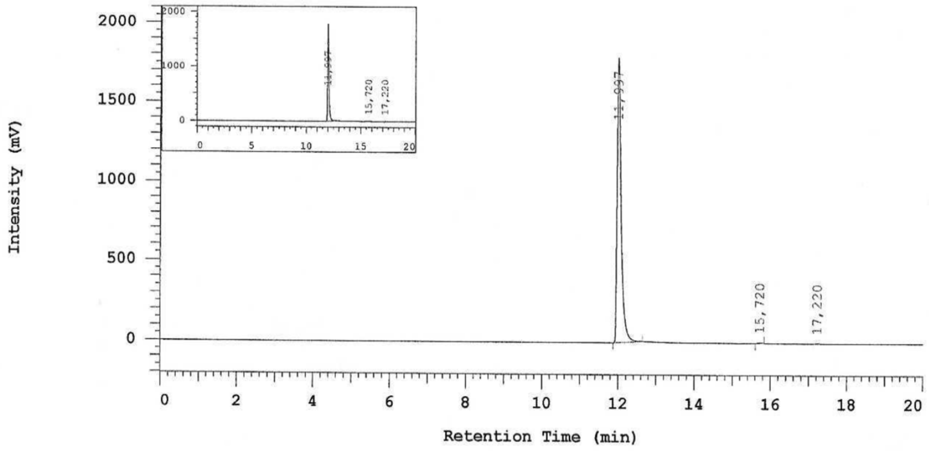

No.	RT	Area	Conc 1	BC
1	11,997	12853388	99,669	BB
2	15,720	36250	0,281	BB
3	17,220	6381	0,049	BB
		12896019	100,000	

ent-**15**

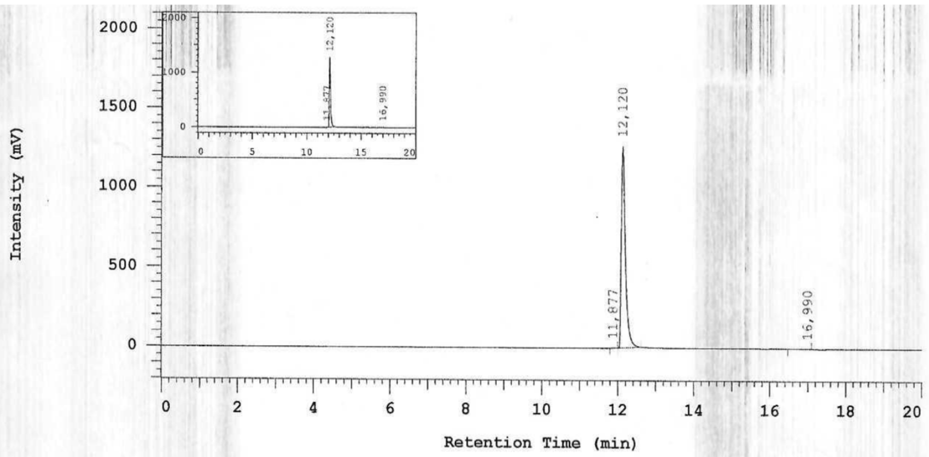

No.	RT	Area	Conc 1	BC
1	11,877	19112	0,207	BB
2	12,120	9185130	99,672	BB
3	16,990	11077	0,120	BB
		9215319	100,000	

16

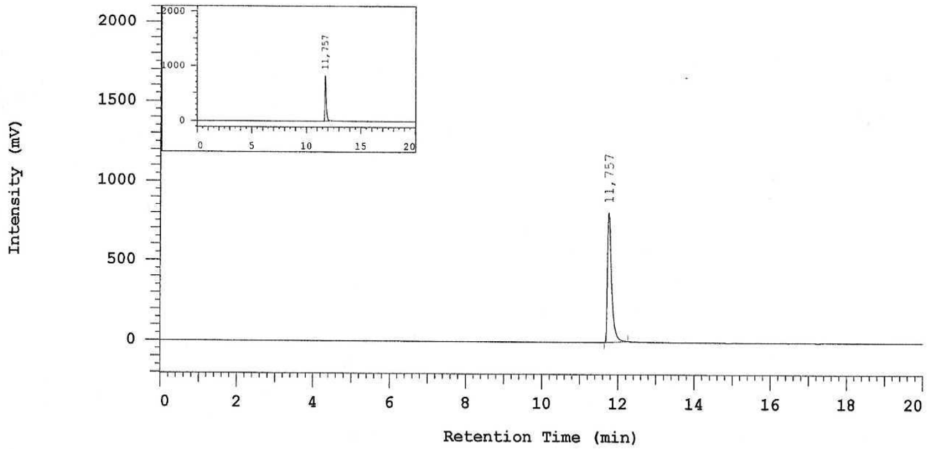

No.	RT	Area	Conc 1	BC
1	11,757	5956134	100,000	BB
		5956134	100,000	

ent-**16**

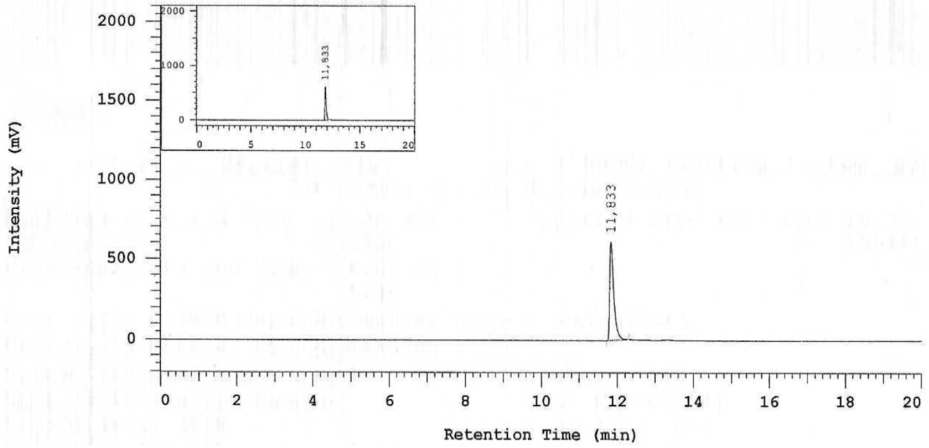

No.	RT	Area	Conc 1	BC
1	11,833	4369621	100,000	VB
		4369621	100,000	

19

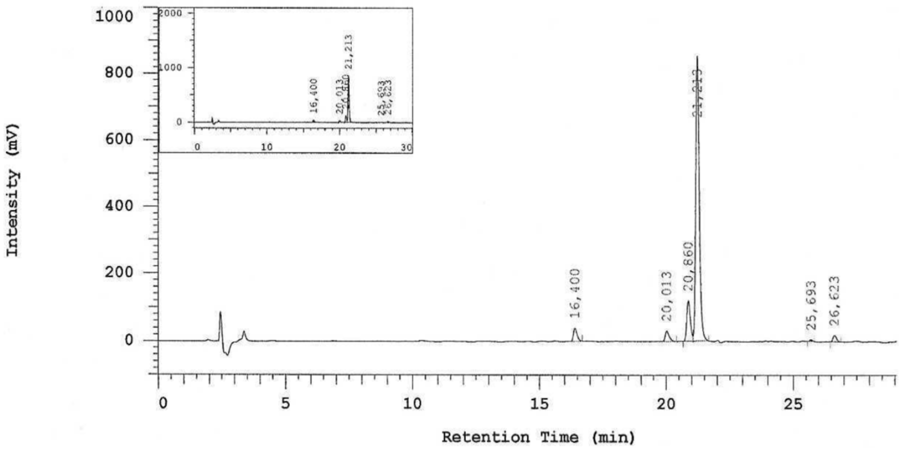

No.	RT	Area	Conc 1
1	16,400	374636	3,760
2	20,013	335260	3,364
3	20,860	1116453	11,204
4	21,213	7949983	79,781
5	25,693	21912	0,220
6	26,623	166453	1,670
		9964697	100,000

21

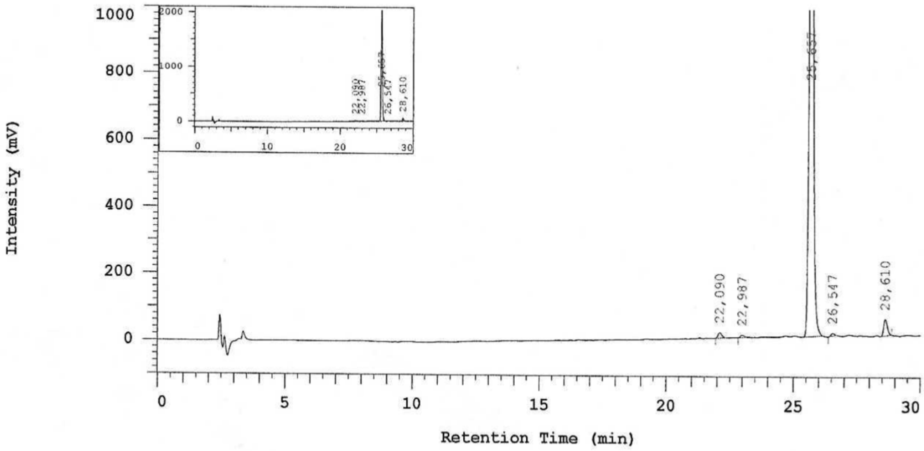

No.	RT	Area	Conc 1	BC
1	22,090	164526	0,761	BB
2	22,987	64130	0,297	BB
3	25,657	20827024	96,380	BB
4	26,547	74212	0,343	BB
5	28,610	479444	2,219	BB
		21609336	100,000	

22

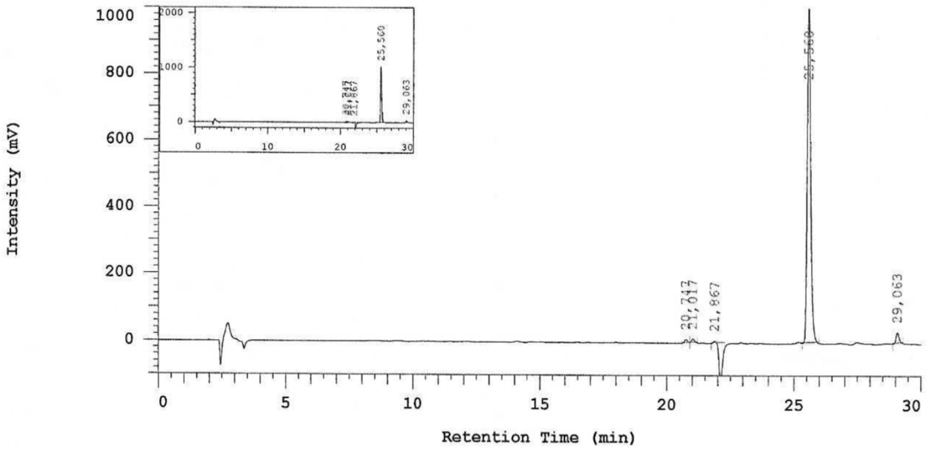

No.	RT	Area	Conc 1	BC
1	20,747	36772	0,396	VV
2	21,017	51091	0,550	VV
3	21,867	4597	0,049	VV
4	25,560	9001961	96,915	VV
5	29,063	194062	2,089	VV
		9288483	100,000	

23

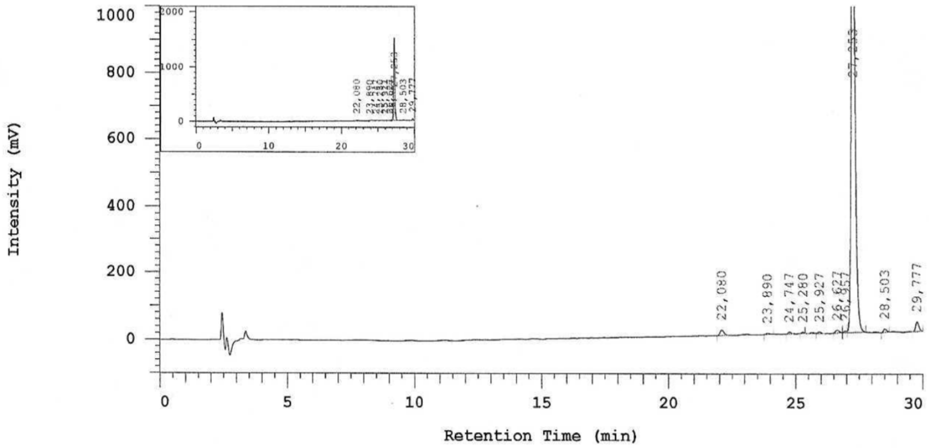

No.	RT	Area	Conc 1	BC
1	22,080	163558	1,039	BB
2	23,890	33597	0,214	BB
3	24,747	38365	0,244	BB
4	25,280	17647	0,112	BB
5	25,927	47412	0,301	VB
6	26,627	82632	0,525	BB
7	26,957	37586	0,239	BV
8	27,253	14983443	95,215	VB
9	28,503	76346	0,485	BB
10	29,777	255789	1,625	BB
		15736375	100,000	

24

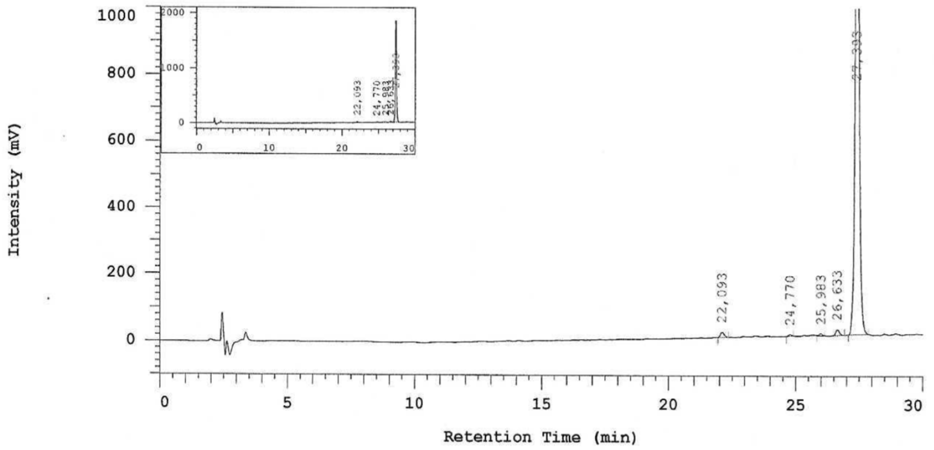

No.	RT	Area	Conc 1	BC
1	22,093	156768	0,840	BB
2	24,770	37500	0,201	BB
3	25,983	53938	0,289	VB
4	26,633	171512	0,919	BB
5	27,393	18247936	97,752	BB
		18667654	100,000	

25

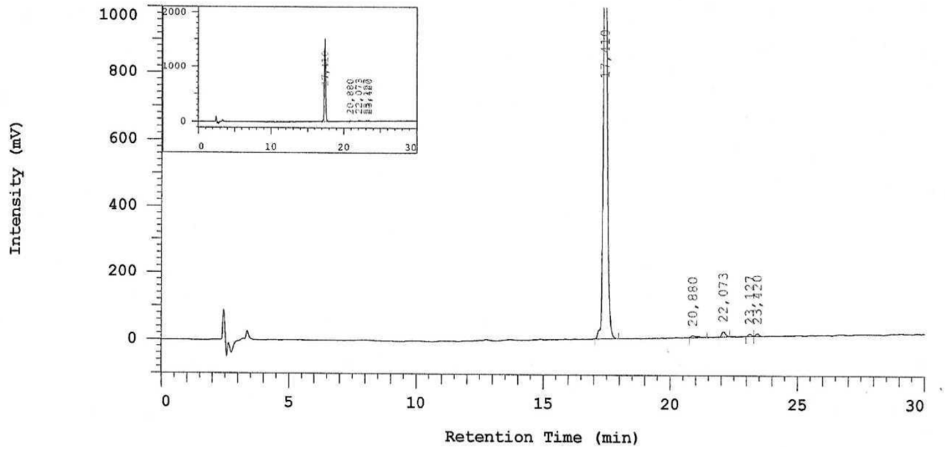

No.	RT	Area	Conc 1	BC
1	17,410	14441685	97,389	BB
2	20,880	87716	0,592	BB
3	22,073	159488	1,076	BB
4	23,127	62396	0,421	BV
5	23,420	77569	0,523	VB
		14828854	100,000	

ent-**25**

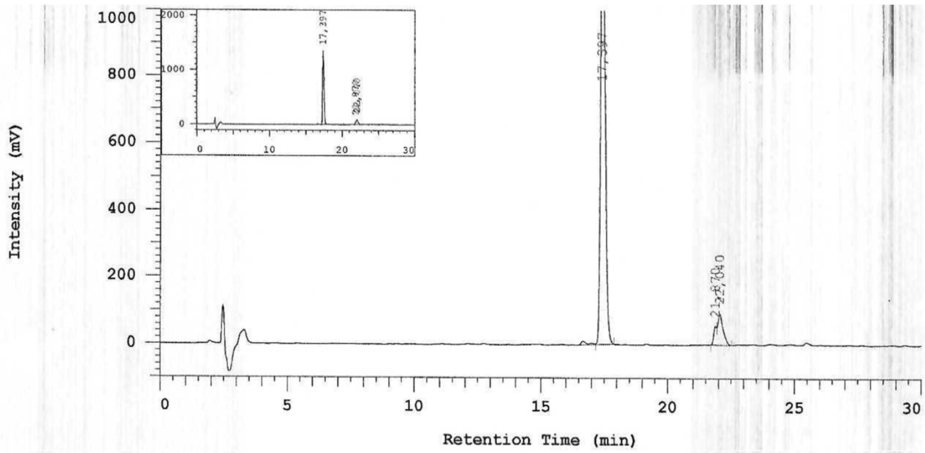

No.	RT	Area	Conc 1	BC
1	17,397	16236597	90,219	VB
2	21,870	408683	2,271	BV
3	22,040	1351617	7,510	VB
		17996897	100,000	

26

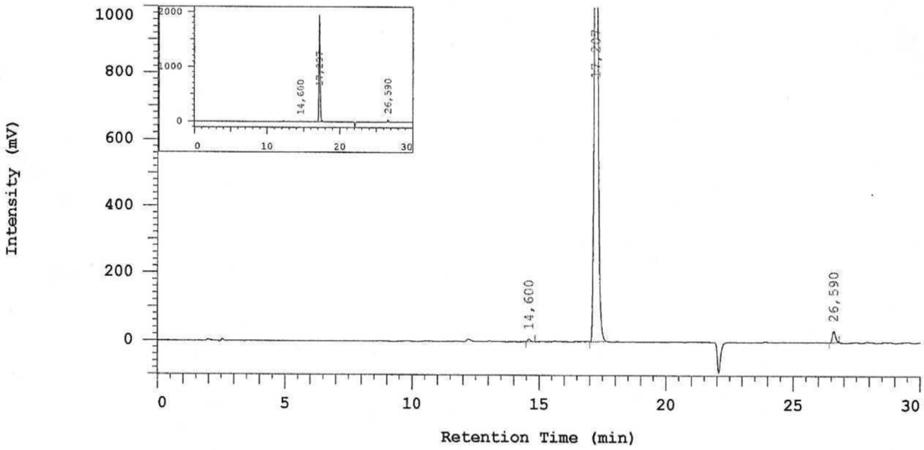

No.	RT	Area	Conc 1	BC
1	14,600	56179	0,314	BB
2	17,207	17518160	98,054	BB
3	26,590	291565	1,632	BB
		17865904	100,000	

27

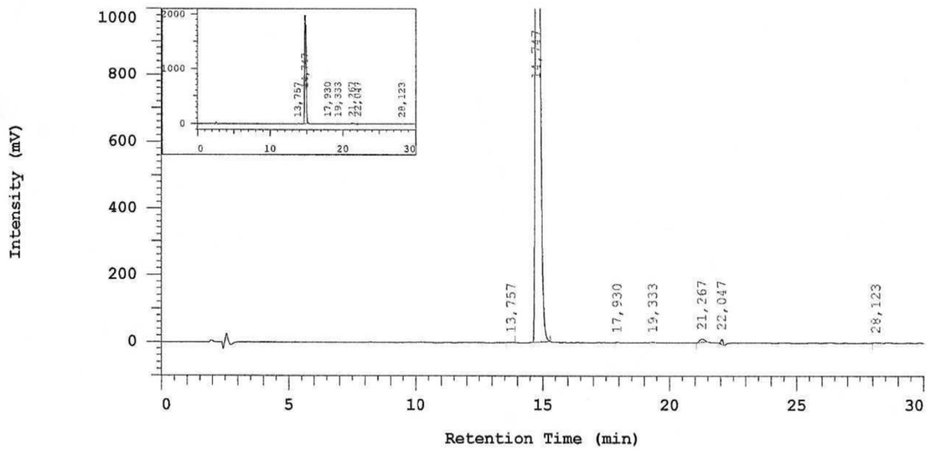

No.	RT	Area	Conc 1	BC
1	13,757	11566	0,046	BB
2	14,747	24938723	98,973	BB
3	17,930	8279	0,033	BB
4	19,333	8373	0,033	BB
5	21,267	119604	0,475	BB
6	22,047	87485	0,347	BB
7	28,123	23462	0,093	BB
		25197492	100,000	

ent-**27**

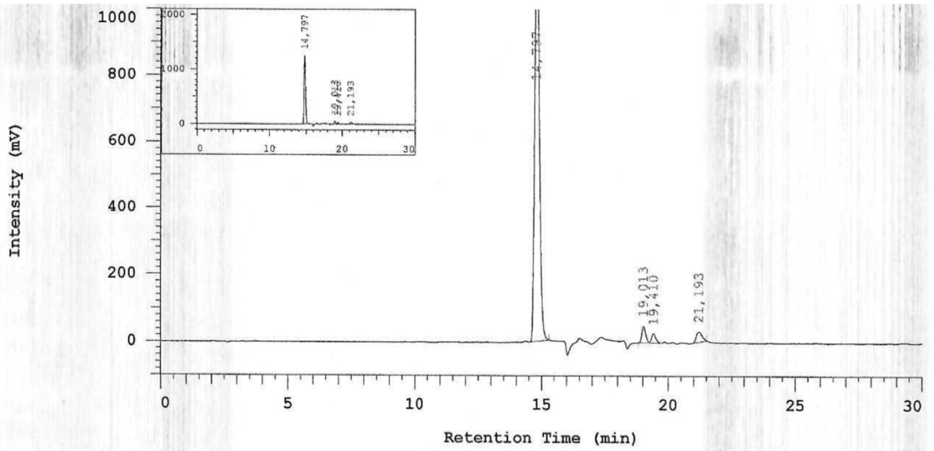

No.	RT	Area	Conc 1	BC
1	14,797	15326188	92,209	BB
2	19,013	511270	3,076	BV
3	19,410	302188	1,818	VB
4	21,193	481473	2,897	BB
		16621119	100,000	

28

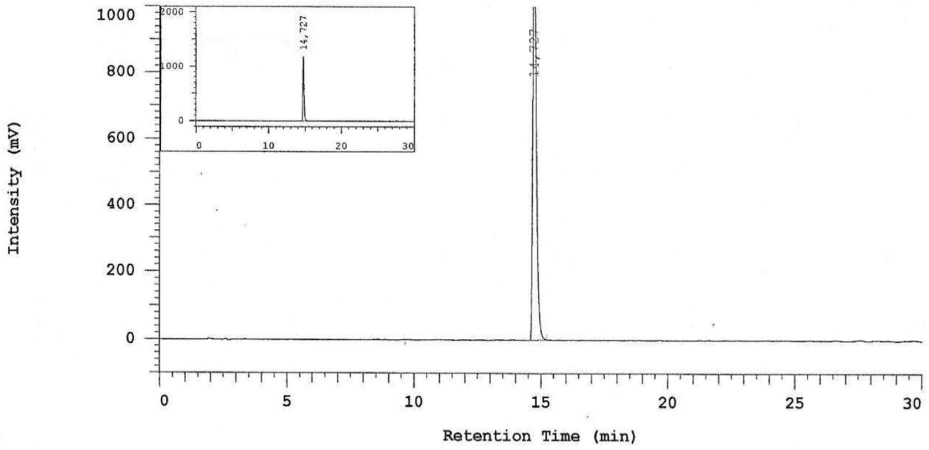

No.	RT	Area	Conc 1	BC
1	14,727	11230830	100,000	BB
		11230830	100,000	

ent-**28**

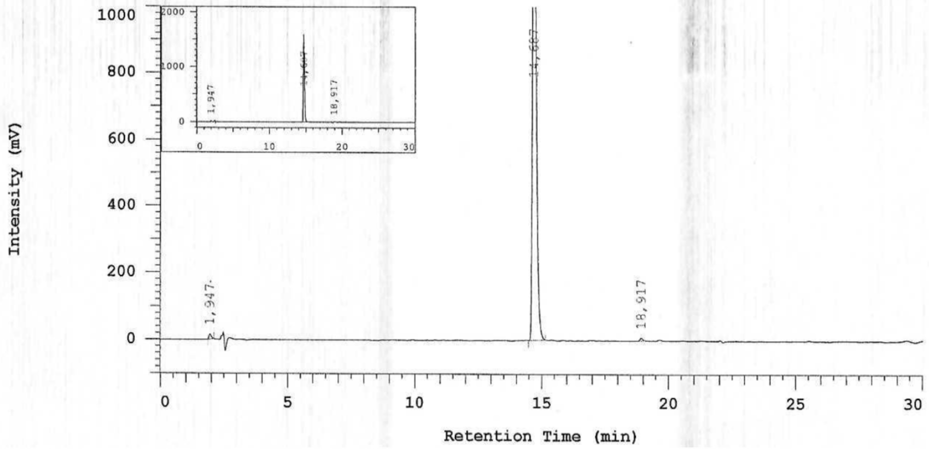

No.	RT	Area	Conc 1	BC
1	1,947	88640	0,569	BB
2	14,687	15445980	99,078	BB
3	18,917	55172	0,354	BB
		15589792	100,000	

31

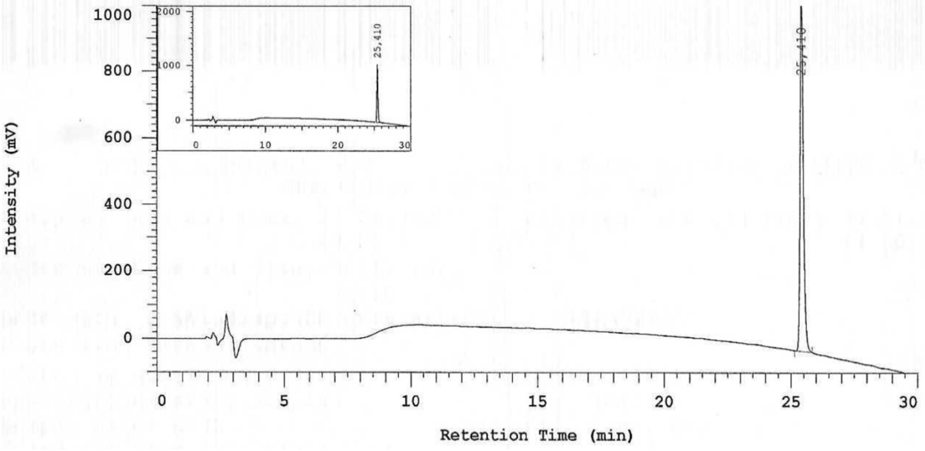

No.	RT	Area	Conc 1	BC
1	25,410	9119122	100,000	MC
		9119122	100,000	

33

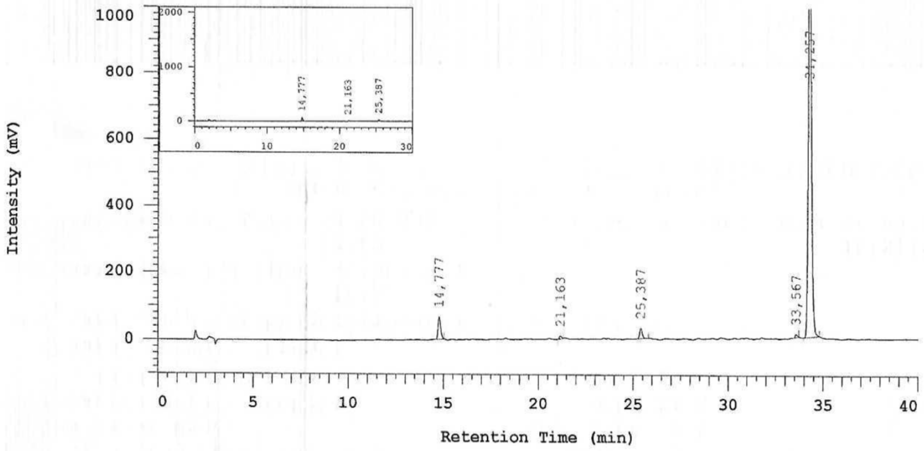

No.	RT	Area	Conc 1	BC
1	14,777	591813	4,091	BB
2	21,163	64911	0,449	BB
3	25,387	275437	1,904	BB
4	33,567	75049	0,519	BB
5	34,257	13459692	93,038	BB
		14466902	100,000	

35

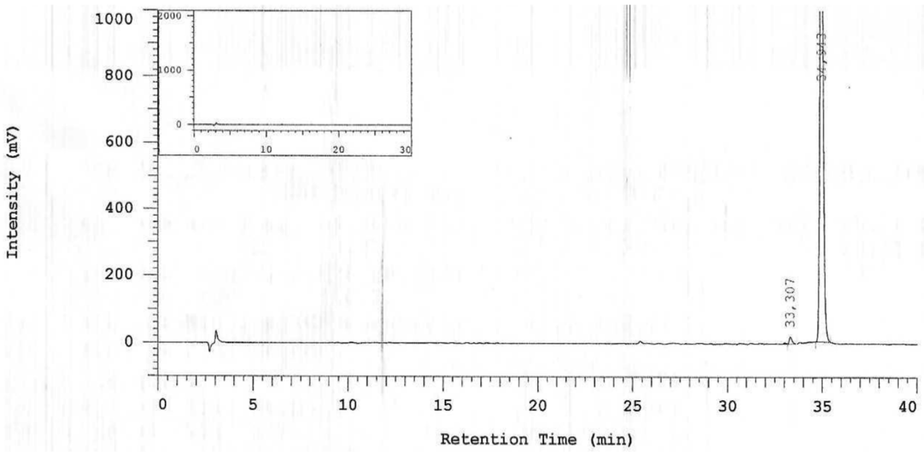

No.	RT	Area	Conc 1	BC
1	33,307	200894	1,082	VB
2	34,943	18364321	98,918	BB
		18565215	100,000	

37

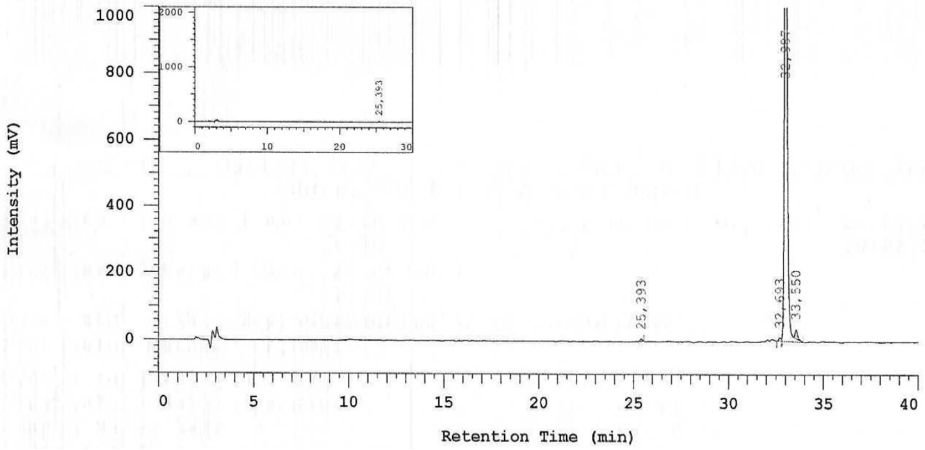

No.	RT	Area	Conc 1	BC
1	25,393	52071	0,313	BB
2	32,693	80232	0,482	BV
3	32,987	16293342	97,804	VV
4	33,550	233574	1,402	TBB
		16659219	100,000	

41

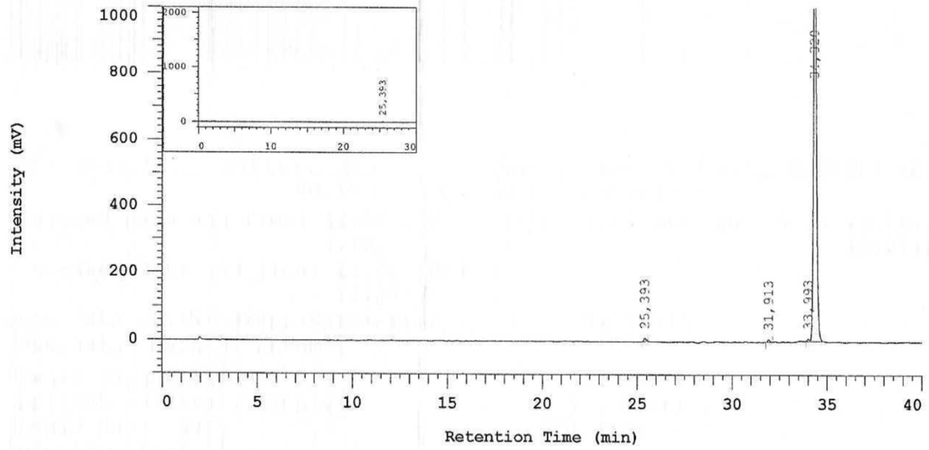

No.	RT	Area	Conc 1	BC
1	25,393	73001	0,558	BB
2	31,913	61136	0,468	BB
3	33,993	52661	0,403	BV
4	34,320	12890423	98,572	VB
		13077221	100,000	

42

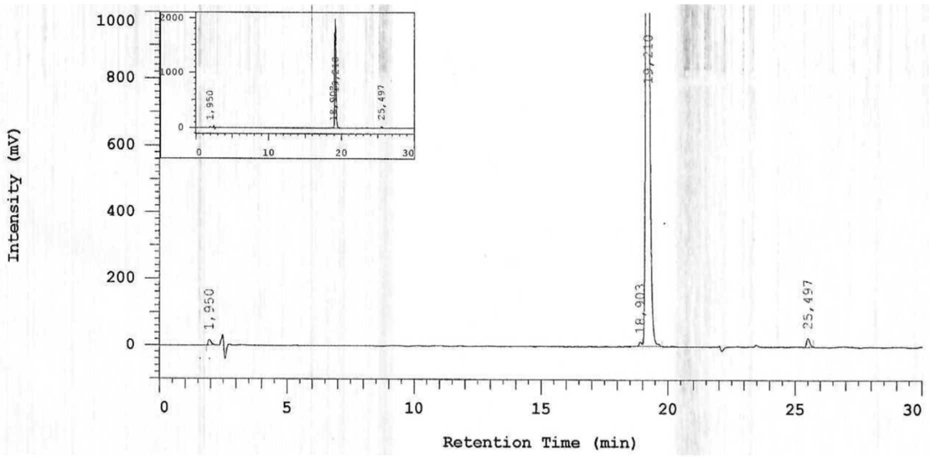

No.	RT	Area	Conc 1	BC
1	1,950	119757	0,546	BB
2	18,903	89599	0,409	BV
3	19,210	21475435	97,994	VB
4	25,497	230210	1,050	BB
		21915001	100,000	

43

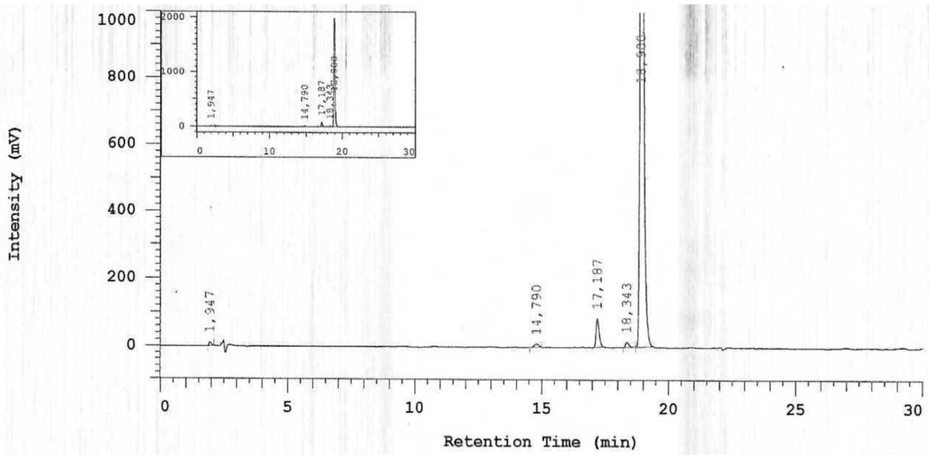

No.	RT	Area	Conc 1	BC
1	1,947	63402	0,315	BB
2	14,790	107941	0,536	BB
3	17,187	671981	3,336	BB
4	18,343	145955	0,725	BB
5	18,900	19154206	95,089	BB
		20143485	100,000	

44

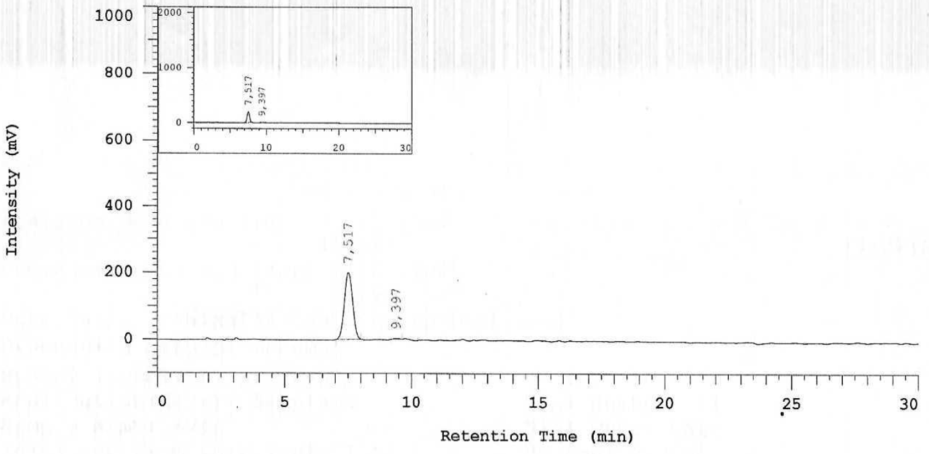

No.	RT	Area	Conc 1	BC
1	7,517	4433538	99,237	BB
2	9,397	34085	0,763	BB
		4467623	100,000	

45

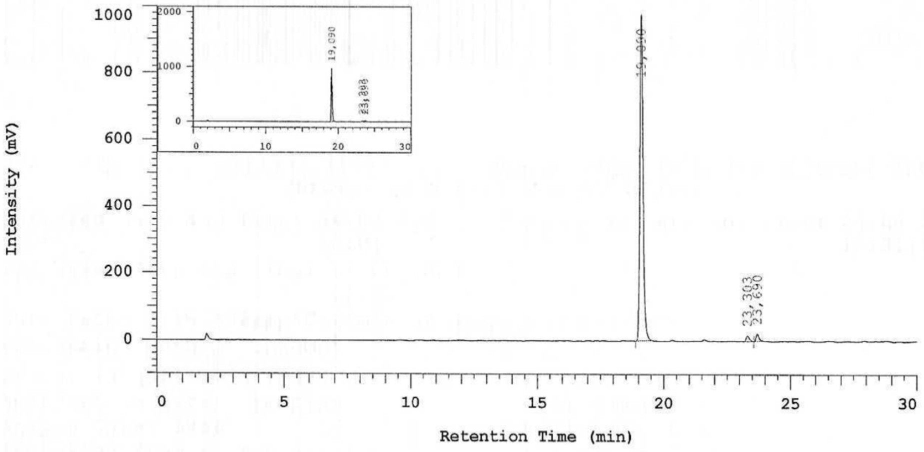

No.	RT	Area	Conc 1	BC
1	19,090	8290713	96,367	BB
2	23,303	143559	1,669	BB
3	23,690	168961	1,964	BB
		8603233	100,000	

HRMS spectra of the synthesized compounds

15

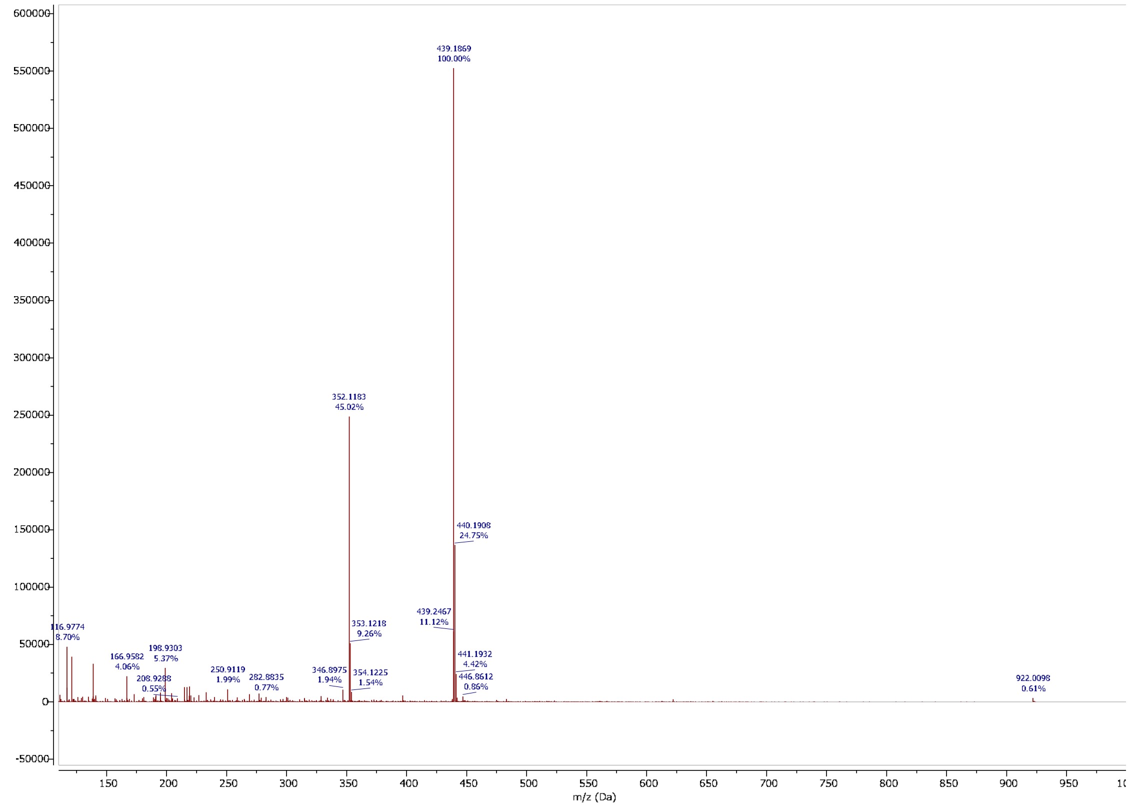

ent-**15**

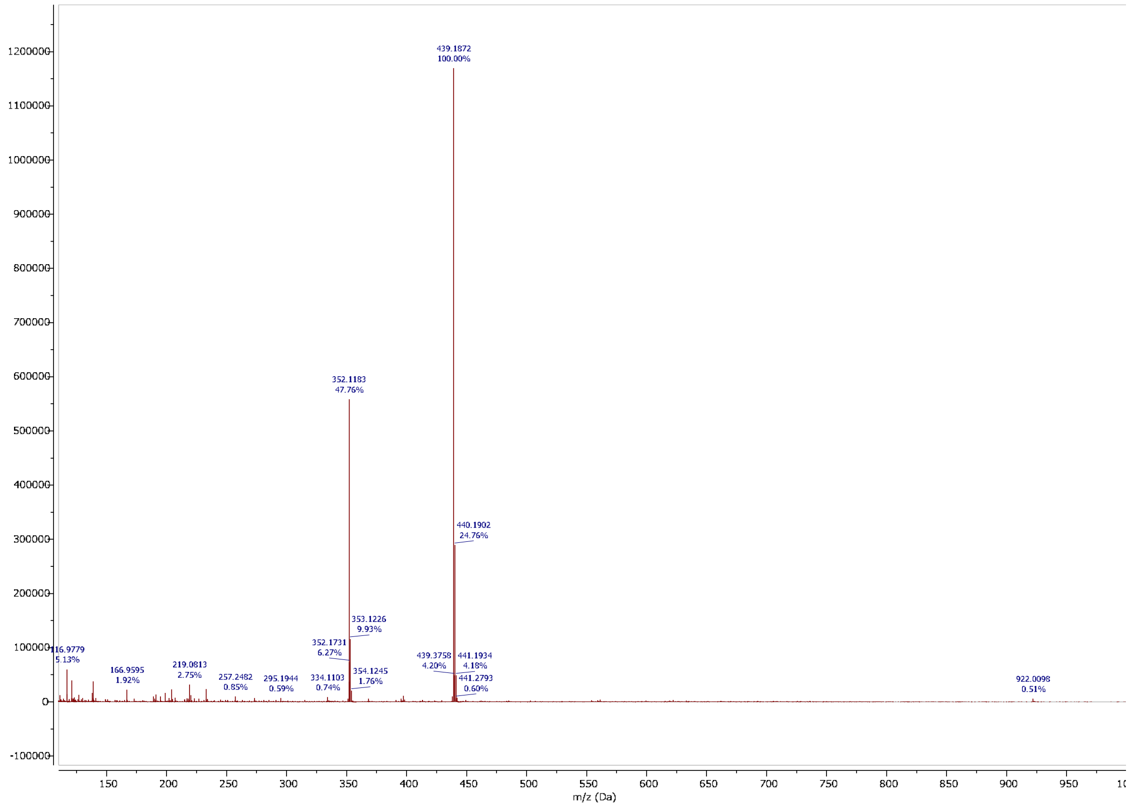

16

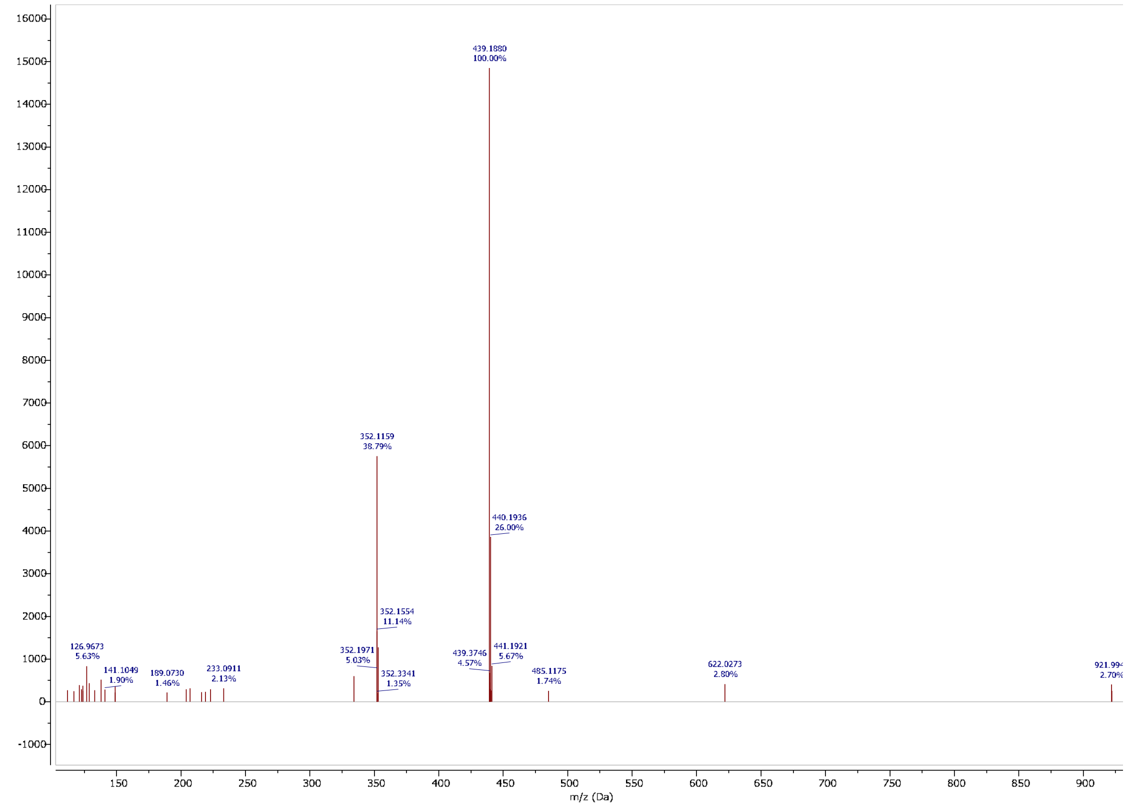
439.1880
100.00%
352.1159
38.79%
440.1936
26.00%
352.1554
11.14%
126.9673
5.63%
141.1049
1.90%
189.0730
1.46%
233.0911
2.13%
352.1971
5.03%
352.3341
1.35%
439.3746
4.57%
441.1921
5.67%
485.1175
1.74%
622.0273
2.80%
m/z (Da)

ent-**16**

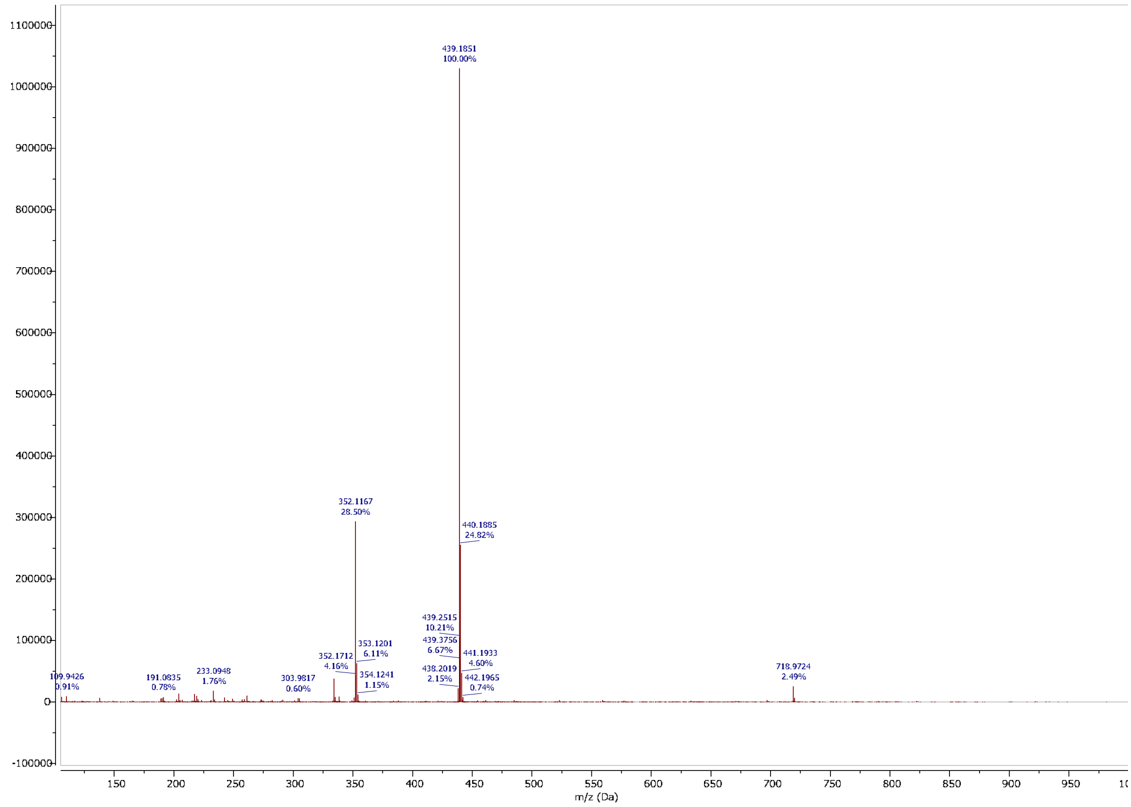
439.1851
100.00%
352.1167
28.50%
440.1885
24.82%
439.2515
10.21%
439.3756
6.67%
441.1933
4.60%
438.2019
2.15%
442.1965
0.74%
352.1712
4.16%
353.1201
6.11%
354.1241
1.15%
109.9426
0.91%
191.0835
0.78%
233.0948
1.76%
303.9817
0.60%
718.9724
2.49%
m/z (Da)

19

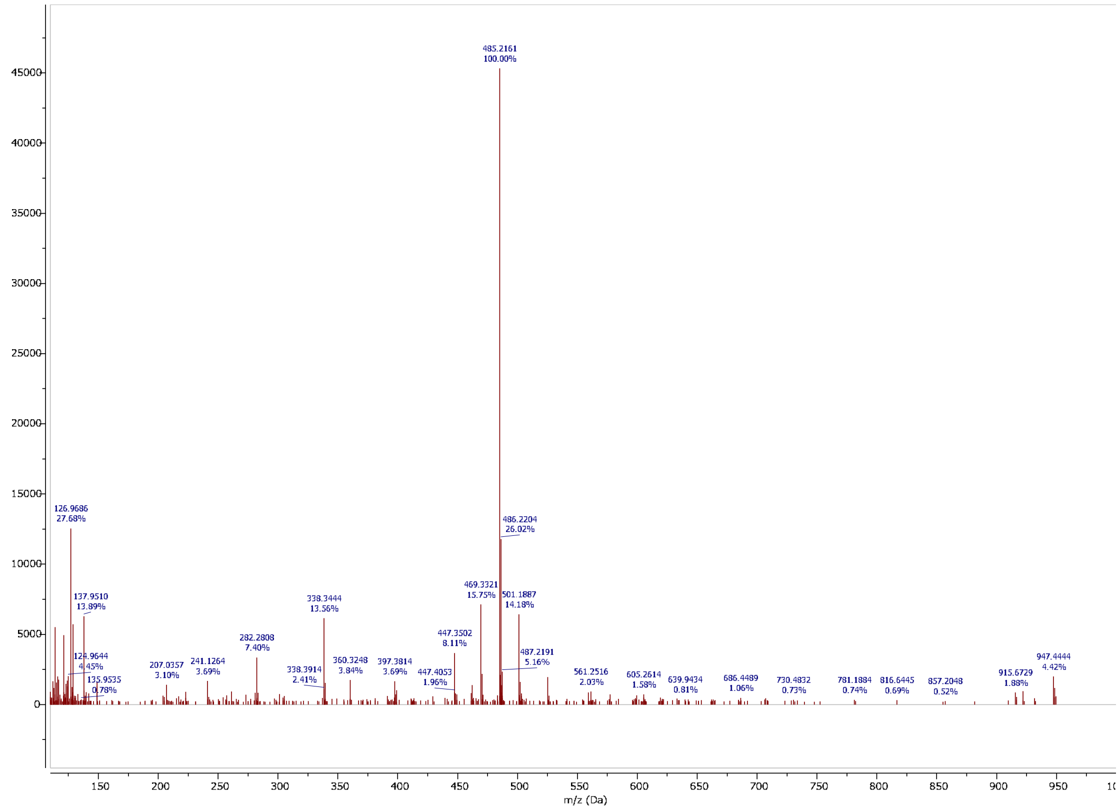
485.2161 100.00%
126.9686 27.68%
486.2204 26.02%
137.9510 13.89%
338.3444 13.56%
469.3321 15.75%
501.1887 14.18%
447.3502 8.11%
282.2808 7.40%
487.2191 5.16%
947.4444 4.42%
m/z (Da)

20

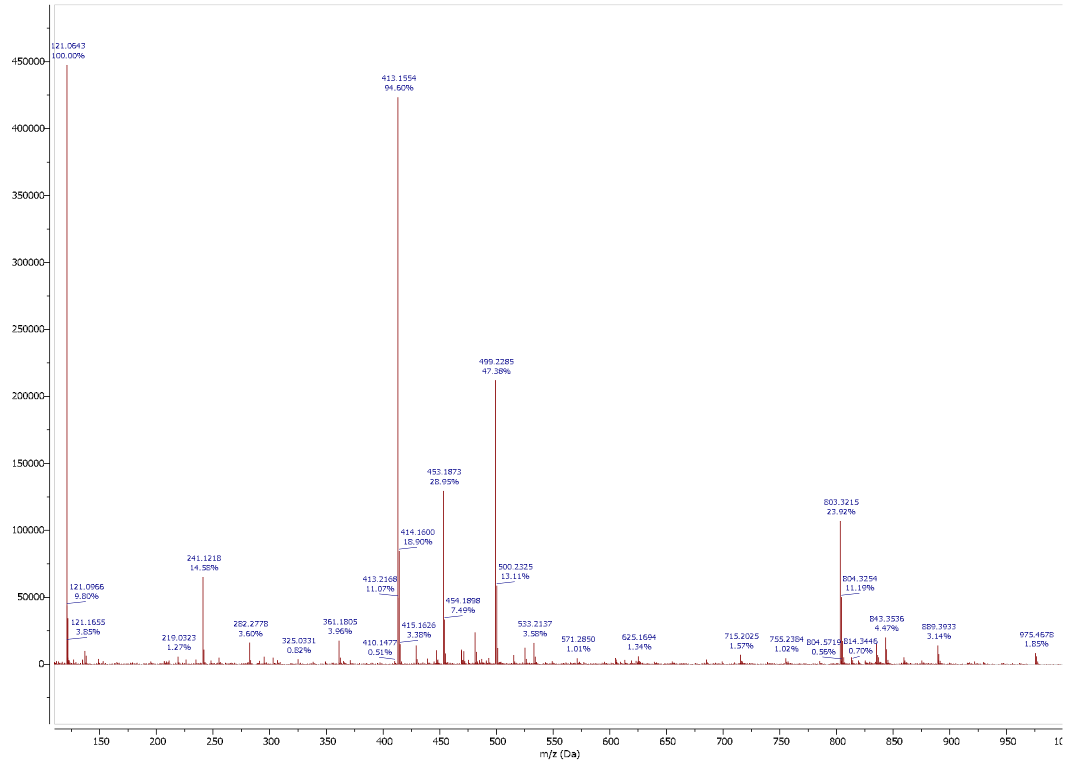
121.0643 100.00%
413.1554 94.60%
499.2285 47.38%
453.1873 28.95%
803.3215 23.92%
414.1600 18.90%
241.1218 14.58%
500.2325 13.11%
413.2168 11.07%
804.3254 11.19%
121.0966 9.80%
454.1898 7.49%
m/z (Da)

21

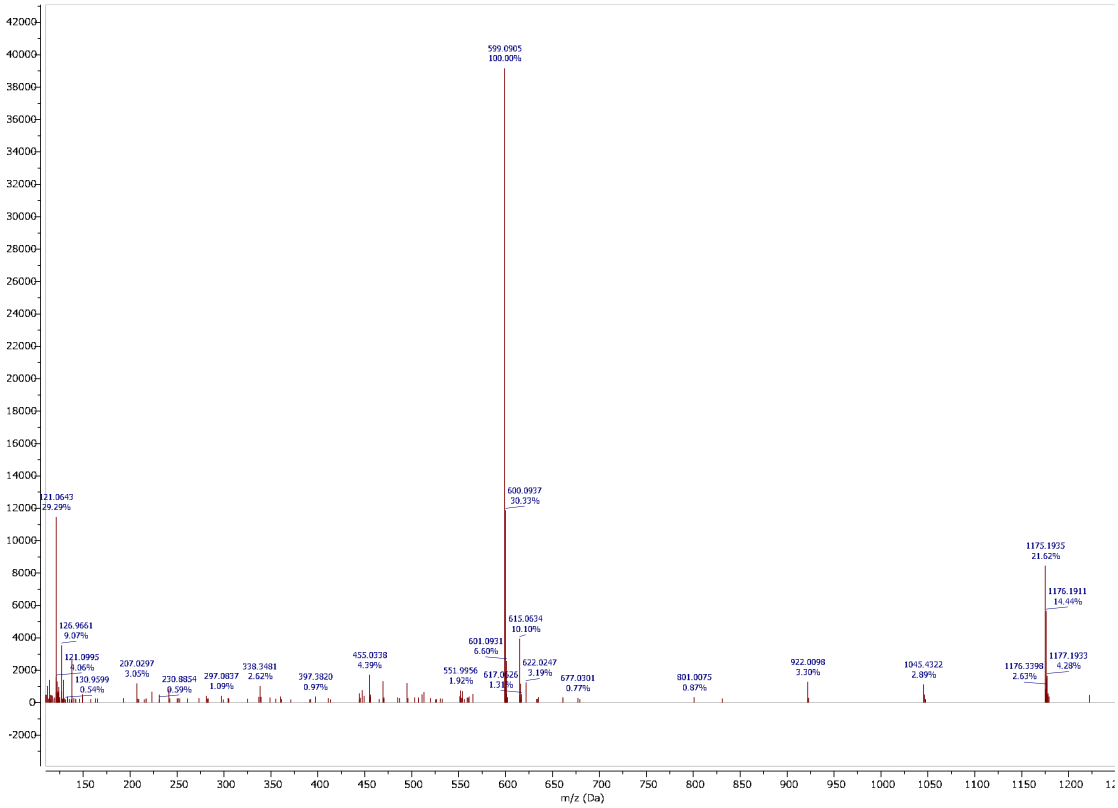
m/z (Da)
599.0905
100.00%
121.0643
29.29%
600.0937
30.33%
1175.1935
21.62%
1176.1911
14.44%
126.9661
9.07%
615.0634
10.10%
601.0931
6.60%
121.0995
4.06%
130.9599
0.54%
207.0297
3.05%
230.8854
0.59%
297.0837
1.09%
338.3481
2.62%
397.3820
0.97%
455.0338
4.39%
551.9956
1.92%
617.0626
1.31%
622.0247
3.19%
677.0301
0.77%
801.0075
0.87%
922.0098
3.30%
1045.4322
2.89%
1176.3398
2.63%
1177.1933
4.28%

22

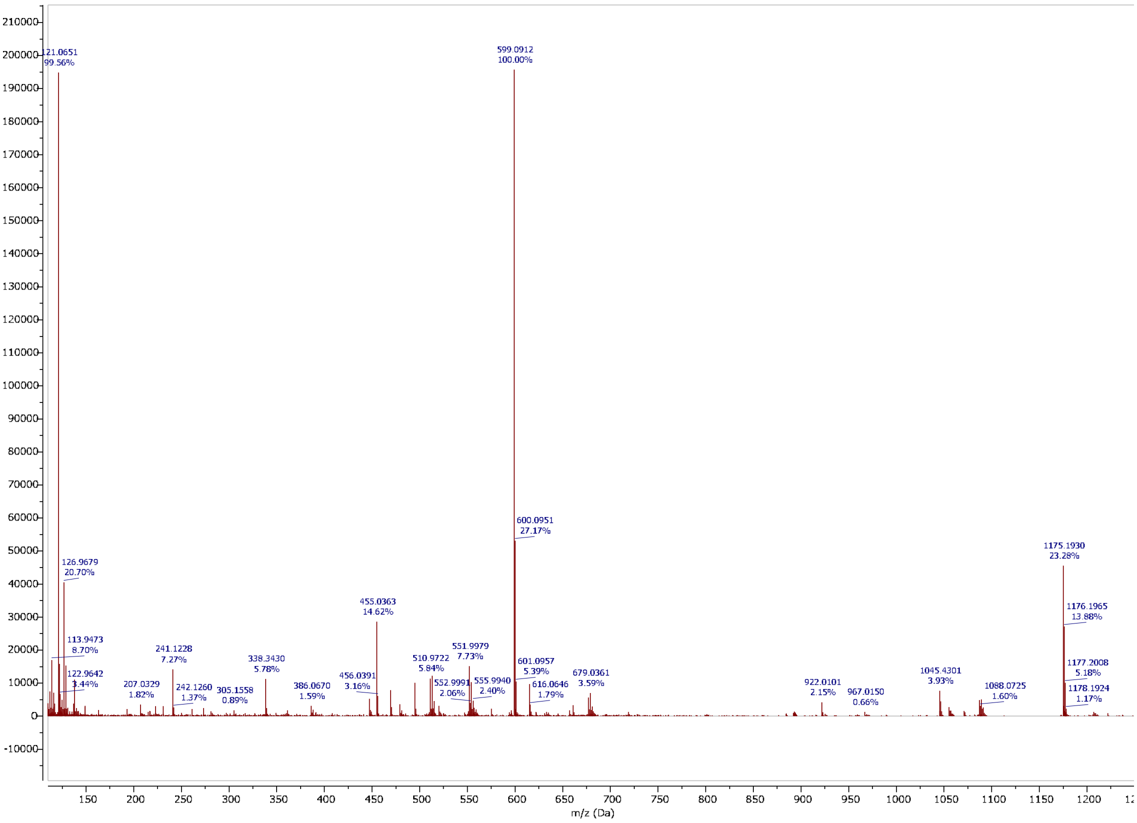
m/z (Da)
121.0651
99.56%
599.0912
100.00%
600.0951
27.17%
1175.1930
23.28%
126.9679
20.70%
1176.1965
13.88%
455.0363
14.62%
113.9473
8.70%
241.1228
7.27%
122.9642
3.44%
338.3430
5.78%
551.9979
7.73%
510.9722
5.84%
601.0957
5.39%
679.0361
3.59%
1045.4301
3.93%
1177.2008
5.18%
207.0329
1.82%
242.1260
1.37%
305.1558
0.89%
386.0670
1.59%
456.0391
3.16%
552.9991
2.06%
555.9940
2.40%
616.0646
1.79%
922.0101
2.15%
967.0150
0.66%
1088.0725
1.60%
1178.1924
1.17%

23

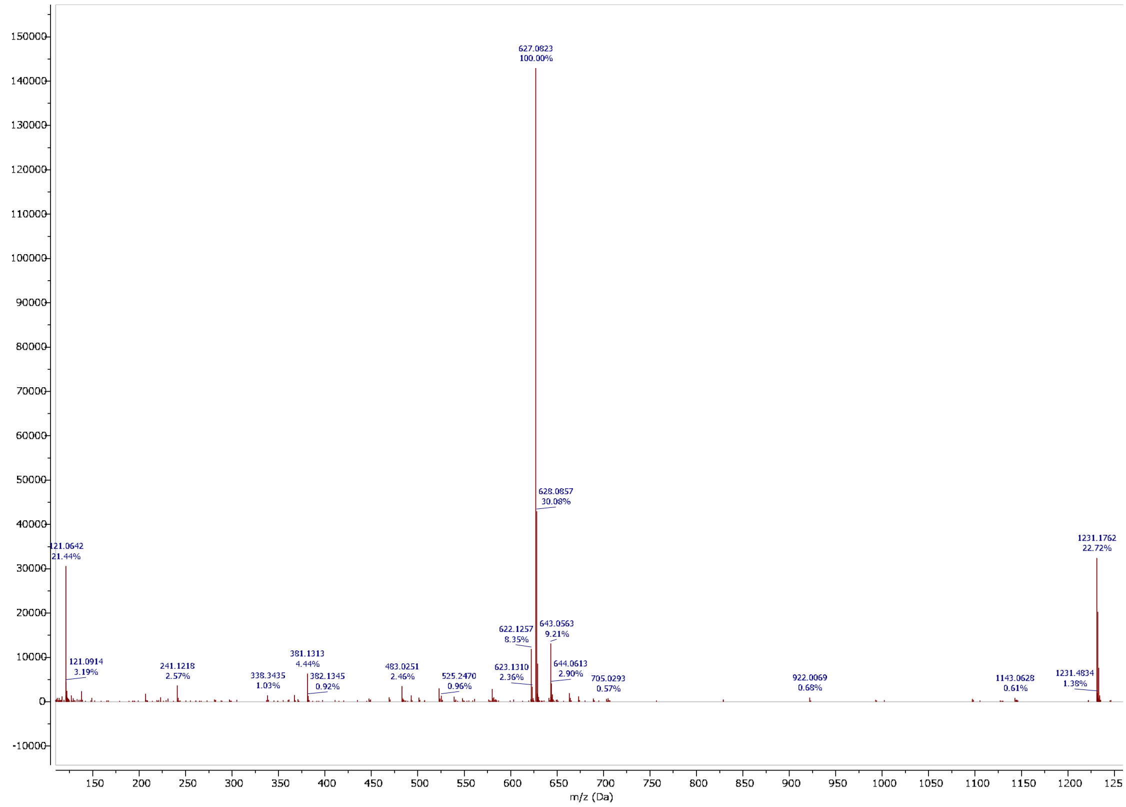
627.0823
100.00%
628.0857
30.08%
121.0642
21.44%
1231.1762
22.72%
643.0563
9.21%
622.1257
8.35%
381.1313
4.44%
121.0914
3.19%
241.1218
2.57%
483.0251
2.46%
623.1310
2.36%
644.0613
2.90%
338.3435
1.03%
382.1345
0.92%
525.2470
0.96%
705.0293
0.57%
922.0069
0.68%
1143.0628
0.61%
1231.4834
1.38%
m/z (Da)

24

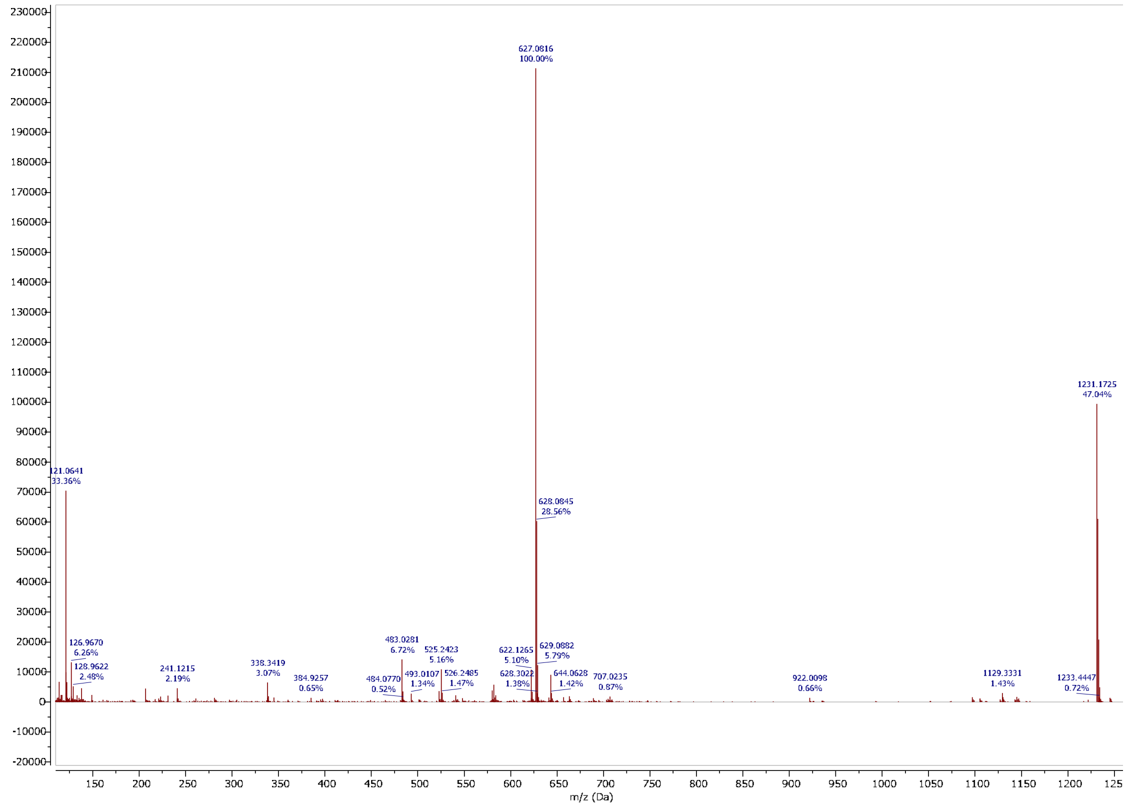
627.0816
100.00%
1231.1725
47.04%
121.0641
33.36%
628.0845
28.56%
126.9670
6.26%
128.9622
2.48%
241.1215
2.19%
338.3419
3.07%
384.9257
0.65%
483.0281
6.72%
484.0770
0.52%
493.0107
1.34%
525.2423
5.16%
526.2485
1.47%
622.1265
5.10%
628.3022
1.38%
629.0882
5.79%
644.0628
1.42%
707.0235
0.87%
922.0098
0.66%
1129.3331
1.43%
1233.4447
0.72%
m/z (Da)

25

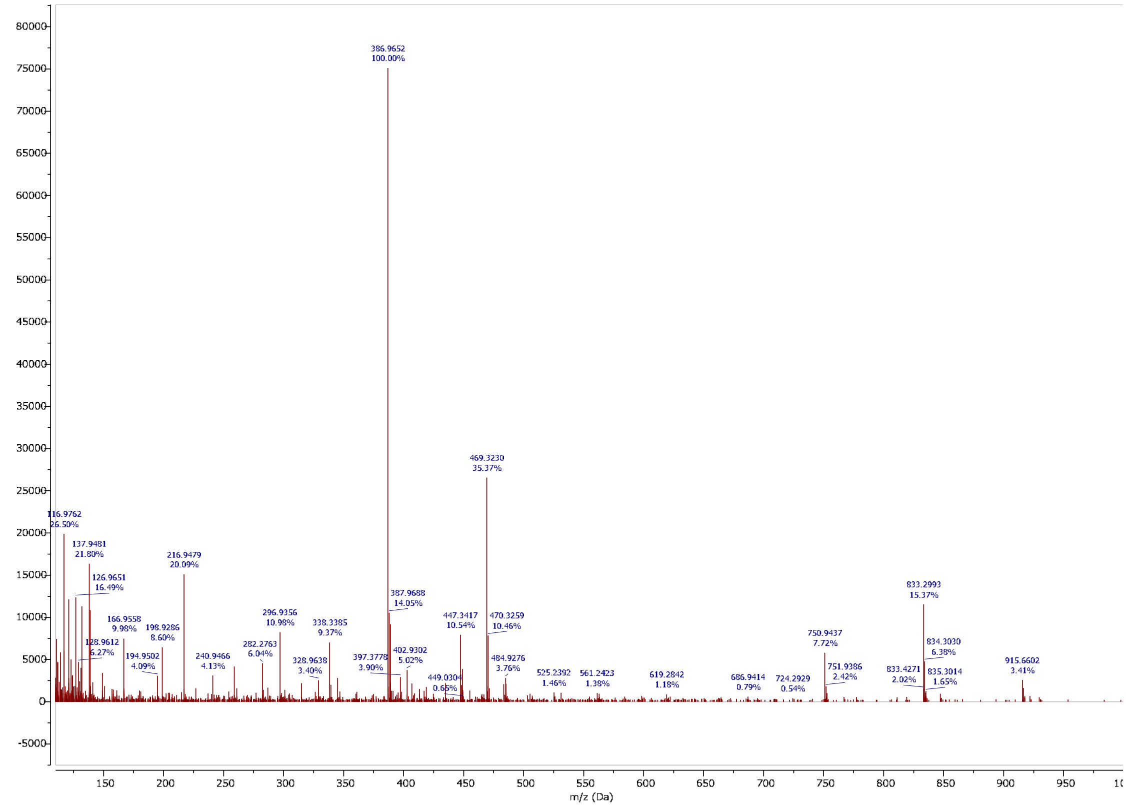

386.9652
100.00%
469.3230
35.37%
116.9762
26.50%
137.9481
21.80%
216.9479
20.09%
126.9651
16.49%
833.2993
15.37%
387.9688
14.05%
296.9356
10.98%
447.3417
10.54%
470.3259
10.46%
166.9558
9.98%
338.3385
9.37%
198.9286
8.60%
750.9437
7.72%
128.9612
6.27%
282.2763
6.04%
834.3030
6.38%
194.9502
4.09%
240.9466
4.13%
328.9638
3.40%
397.3778
3.90%
402.9302
5.02%
449.0304
0.65%
484.9276
3.76%
525.2392
1.46%
561.2423
1.38%
619.2842
1.18%
686.9414
0.79%
724.2929
0.54%
751.9386
2.42%
833.4271
2.02%
835.3014
1.65%
915.6602
3.41%
m/z (Da)

ent-**25**

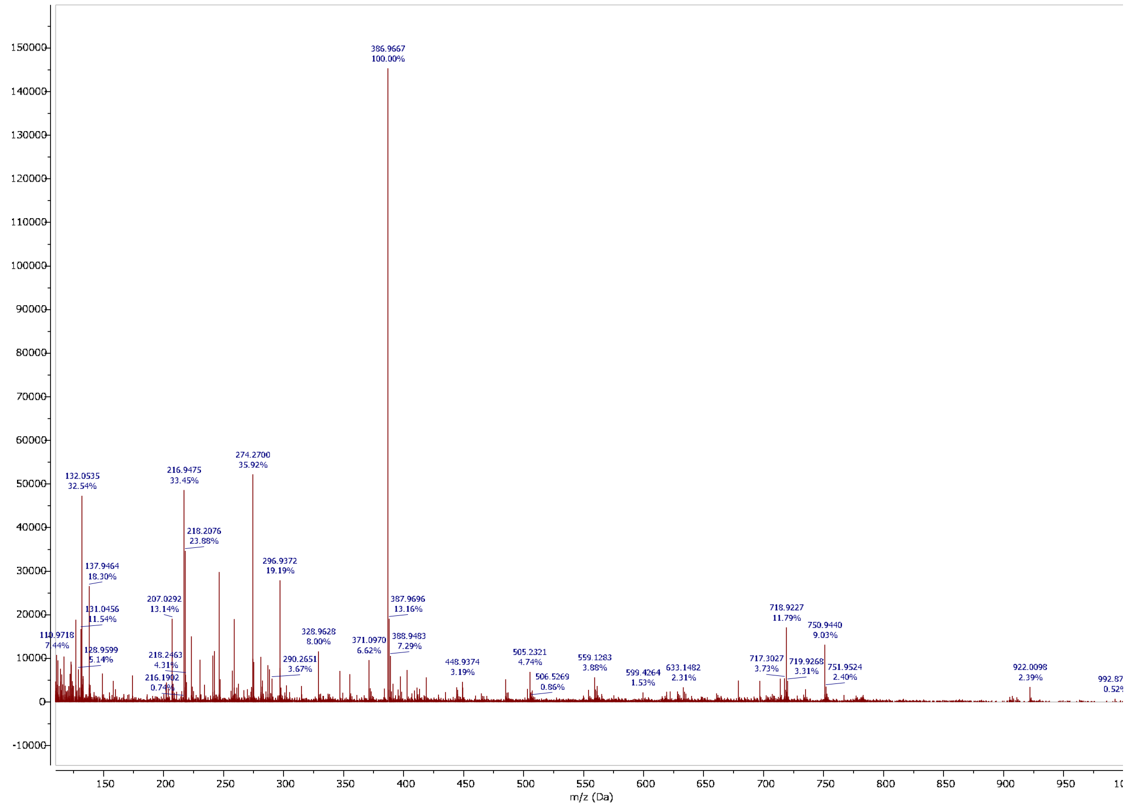

386.9667
100.00%
274.2700
35.92%
216.9475
33.45%
132.0535
32.54%
218.2076
23.88%
296.9372
19.19%
137.9464
18.30%
207.0292
13.14%
387.9696
13.16%
131.0456
11.54%
718.9227
11.79%
750.9440
9.03%
328.9628
8.00%
110.9718
7.44%
388.9483
7.29%
371.0970
6.62%
128.9599
5.14%
218.2463
4.31%
216.1902
0.74%
290.2651
3.67%
448.9374
3.19%
505.2321
4.74%
506.5269
0.86%
559.1283
3.88%
599.4264
1.53%
633.1482
2.31%
717.3027
3.73%
719.9268
3.31%
751.9524
2.40%
922.0098
2.39%
m/z (Da)

26

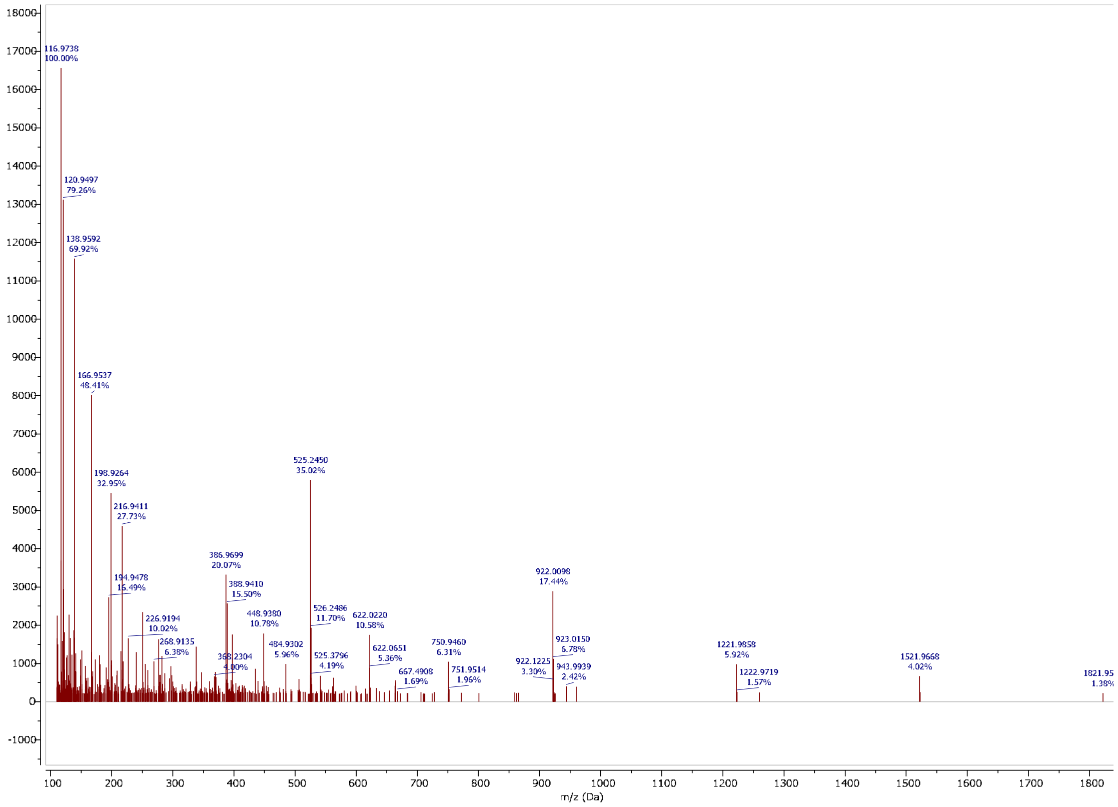
116.9738
100.00%
120.9497
79.26%
138.9592
69.92%
166.9537
48.41%
198.9264
32.95%
216.9411
27.73%
194.9478
16.49%
226.9194
10.02%
268.9135
6.38%
386.9699
20.07%
388.9410
15.50%
368.2304
4.00%
448.9380
10.78%
484.9302
5.96%
525.2450
35.02%
526.2486
11.70%
525.3796
4.19%
622.0220
10.58%
622.0651
5.36%
667.4908
1.69%
750.9460
6.31%
751.9514
1.96%
922.0098
17.44%
922.1225
3.30%
923.0150
6.78%
943.9939
2.42%
1221.9858
5.92%
1222.9719
1.57%
1521.9668
4.02%
1821.95
1.38%
m/z (Da)

27

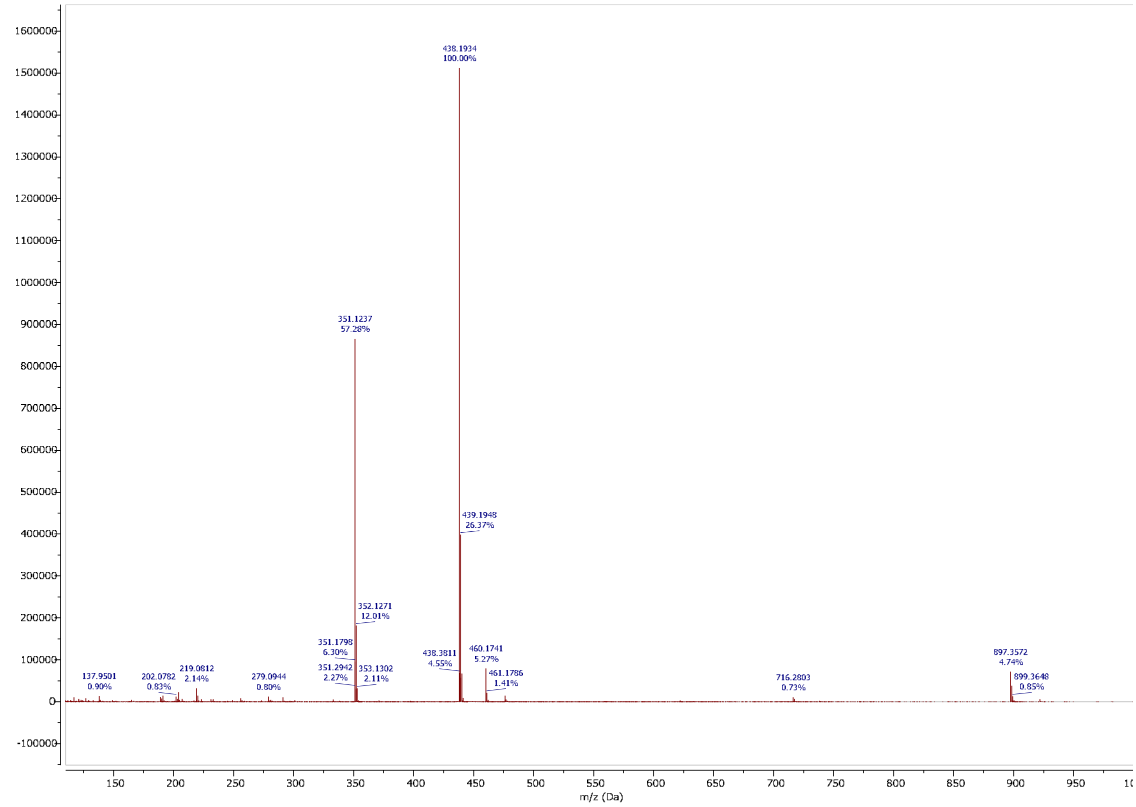
438.1934
100.00%
351.1237
57.28%
439.1948
26.37%
352.1271
12.01%
351.1798
6.30%
351.2942
2.27%
353.1302
2.11%
438.3811
4.55%
460.1741
5.27%
461.1786
1.41%
137.9501
0.90%
202.0782
0.83%
219.0812
2.14%
279.0944
0.80%
716.2803
0.73%
897.3572
4.74%
899.3648
0.85%
m/z (Da)

ent-**27**

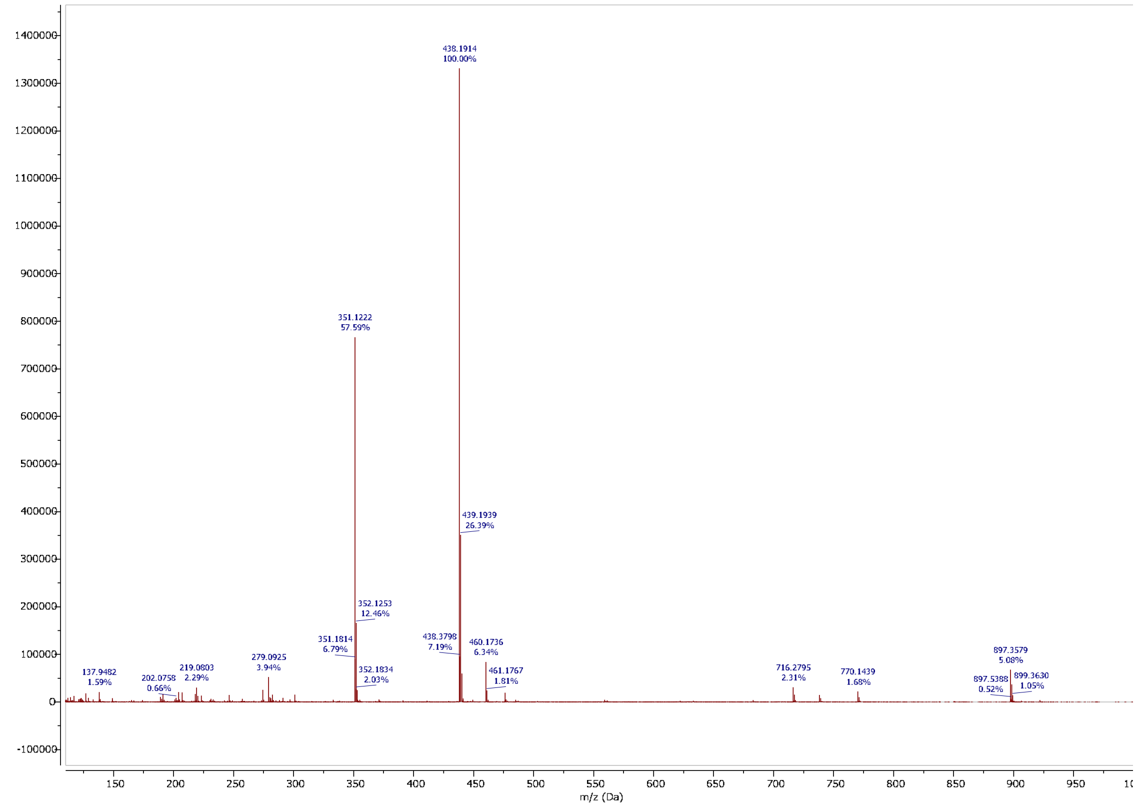
438.1914
100.00%
351.1222
57.59%
439.1939
26.39%
352.1253
12.46%
351.1814
6.79%
352.1834
2.03%
438.3798
7.19%
460.1736
6.34%
461.1767
1.81%
137.9482
1.59%
202.0758
0.66%
219.0803
2.29%
279.0925
3.94%
716.2795
2.31%
770.1439
1.68%
897.3579
5.08%
897.5388
0.52%
899.3630
1.05%
m/z (Da)

28

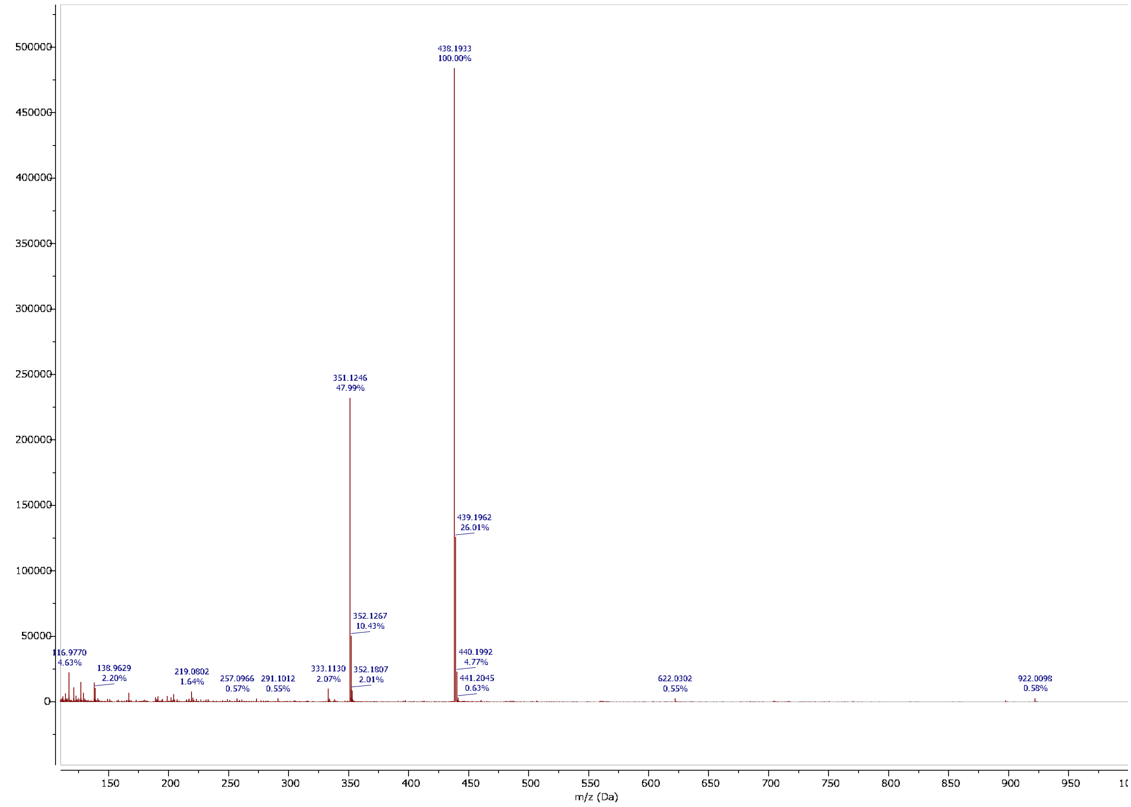
438.1933
100.00%
351.1246
47.99%
439.1962
26.01%
352.1267
10.43%
116.9770
4.63%
138.9629
2.20%
219.0802
1.64%
257.0966
0.57%
291.1012
0.55%
333.1130
2.07%
352.1807
2.01%
440.1992
4.77%
441.2045
0.63%
622.0302
0.55%
922.0098
0.58%
m/z (Da)

ent-**28**

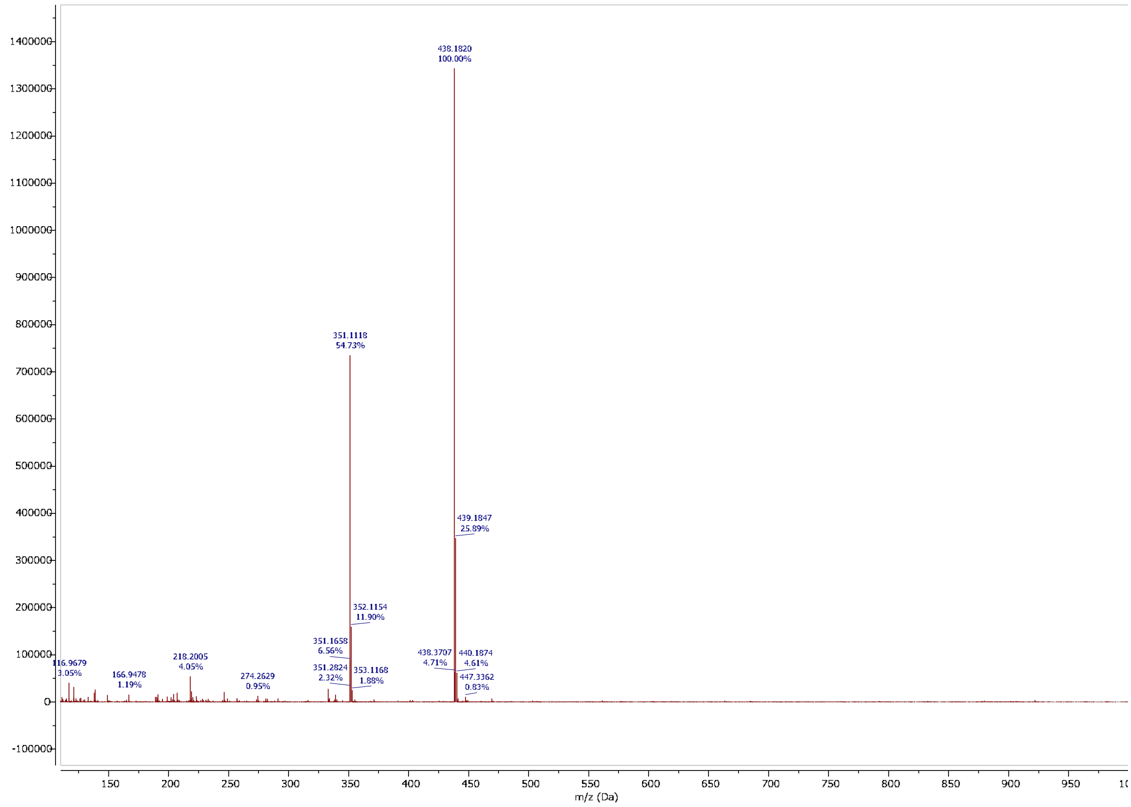
438.1820
100.00%
351.1118
54.73%
439.1847
25.89%
352.1154
11.90%
351.1658
6.56%
438.3707
4.71%
440.1874
4.61%
351.2824
2.32%
353.1168
1.88%
447.3362
0.83%
116.9679
3.05%
166.9478
1.19%
218.2005
4.05%
274.2629
0.95%
m/z (Da)

31

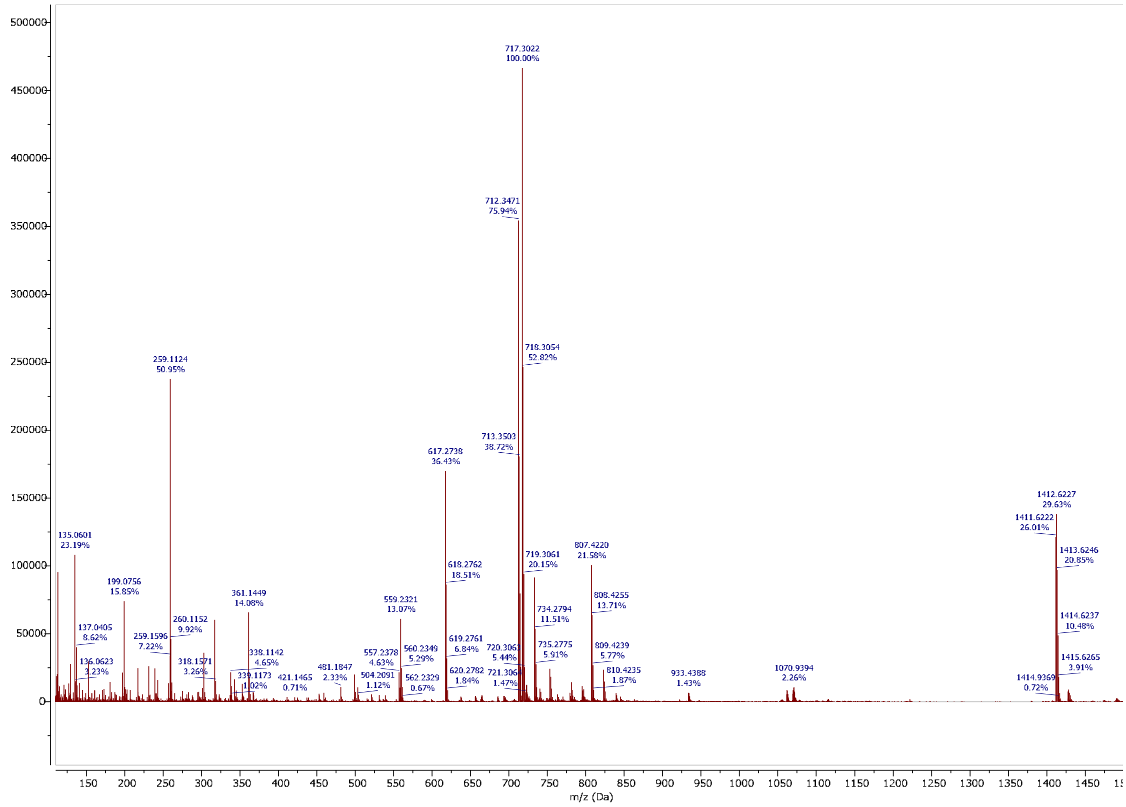
717.3022
100.00%
712.3471
75.94%
718.3054
52.82%
259.1124
50.95%
713.3503
38.72%
617.2738
36.43%
1412.6227
29.63%
1411.6222
26.01%
135.0601
23.19%
807.4220
21.58%
1413.6246
20.85%
719.3061
20.15%
618.2762
18.51%
199.0756
15.85%
361.1449
14.08%
808.4255
13.71%
559.2321
13.07%
734.2794
11.51%
1414.6237
10.48%
260.1152
9.92%
m/z (Da)

32

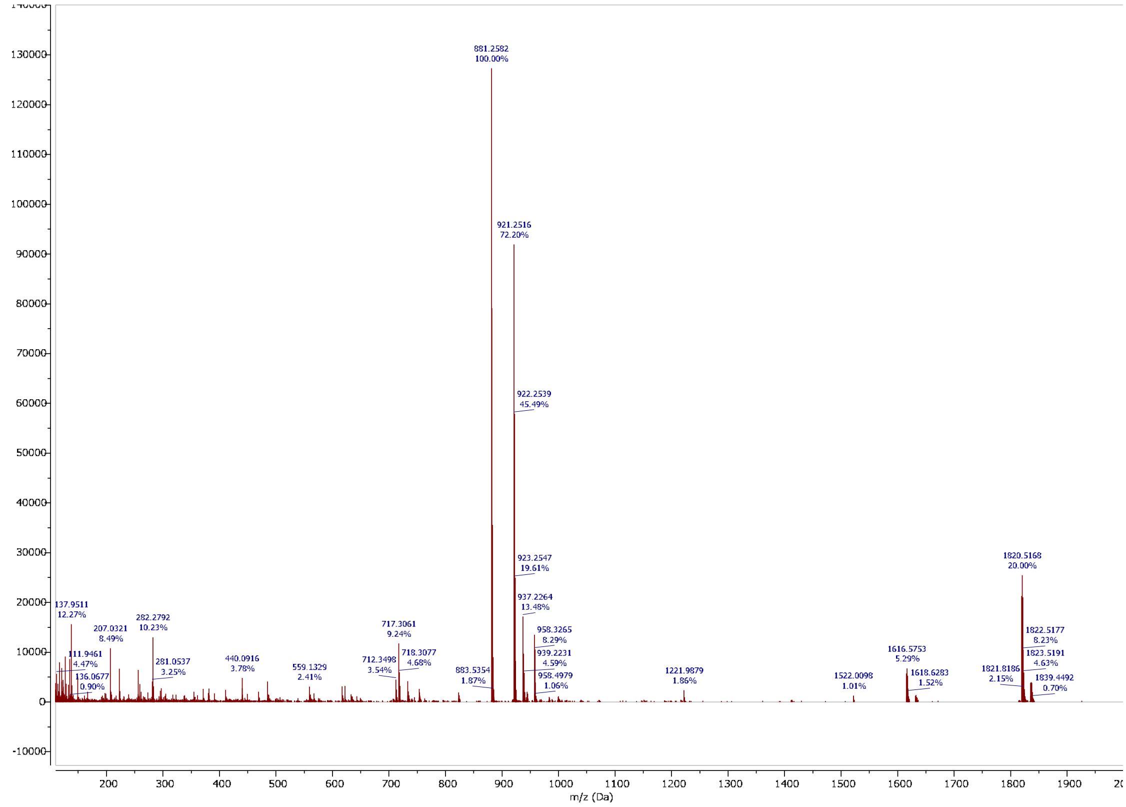
881.2582
100.00%
921.2516
72.20%
922.2539
45.49%
1820.5168
20.00%
923.2547
19.61%
937.2264
13.48%
137.9511
12.27%
282.2792
10.23%
717.3061
9.24%
207.0321
8.49%
958.3265
8.29%
1822.5177
8.23%
m/z (Da)

33

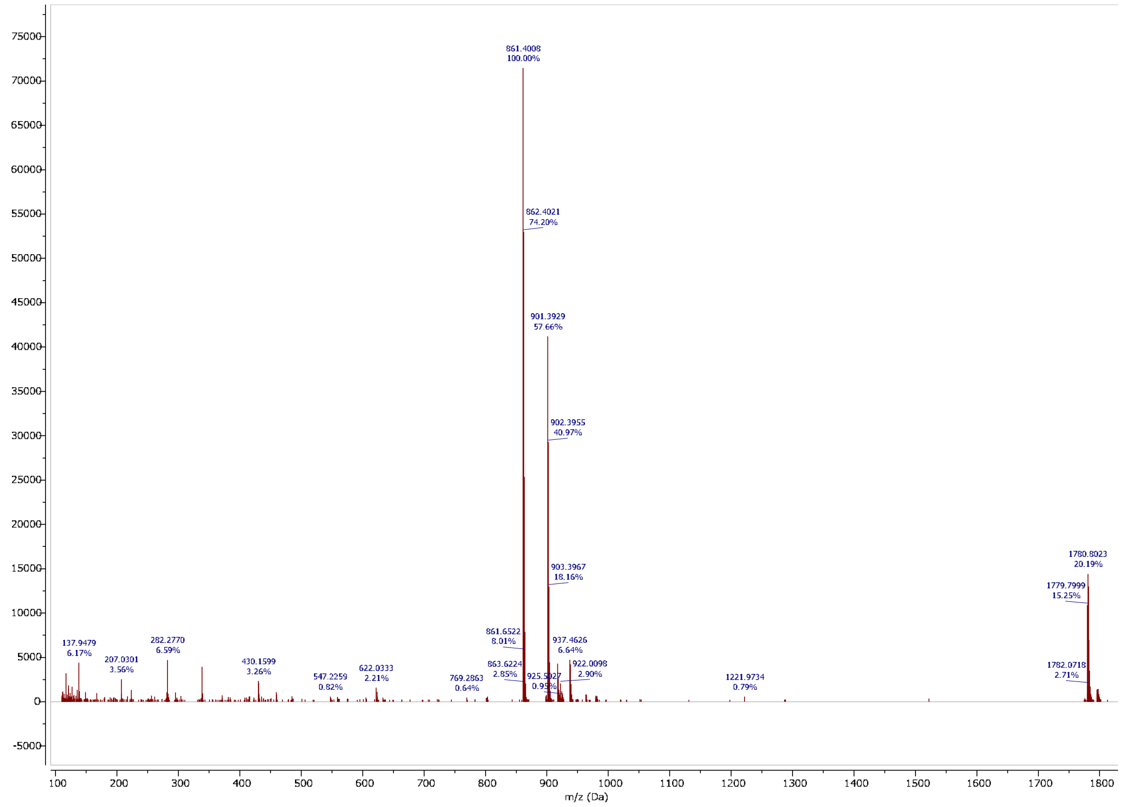
861.4008
100.00%
862.4021
74.20%
901.3929
57.66%
902.3955
40.97%
903.3967
18.16%
1780.8023
20.19%
1779.7999
15.25%
1782.0718
2.71%
861.6522
8.01%
863.6224
2.85%
925.5027
0.95%
937.4626
6.64%
922.0098
2.90%
1221.9734
0.79%
137.9479
6.17%
207.0301
3.56%
282.2770
6.59%
430.1599
3.26%
547.2259
0.82%
622.0333
2.21%
769.2863
0.64%
m/z (Da)

34

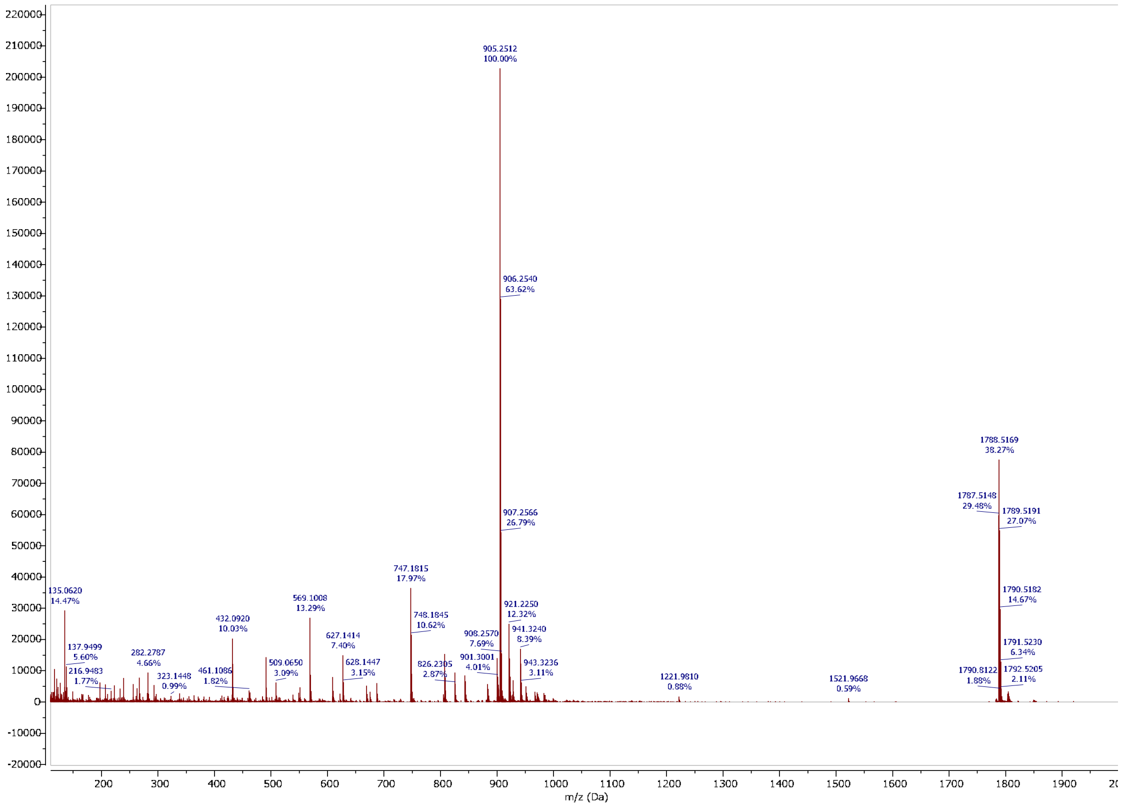
905.2512
100.00%
906.2540
63.62%
907.2566
26.79%
1788.5169
38.27%
1787.5148
29.48%
1789.5191
27.07%
1790.5182
14.67%
1791.5230
6.34%
1790.8122
1.88%
1792.5205
2.11%
747.1815
17.97%
748.1845
10.62%
921.2250
12.32%
941.3240
8.39%
908.2570
7.69%
901.3001
4.01%
943.3236
3.11%
826.2305
2.87%
569.1008
13.29%
627.1414
7.40%
628.1447
3.15%
509.0650
3.09%
432.0920
10.03%
461.1086
1.82%
135.0620
14.47%
137.9499
5.60%
216.9483
1.77%
282.2787
4.66%
323.1448
0.99%
1221.9810
0.88%
1521.9668
0.59%
m/z (Da)

35

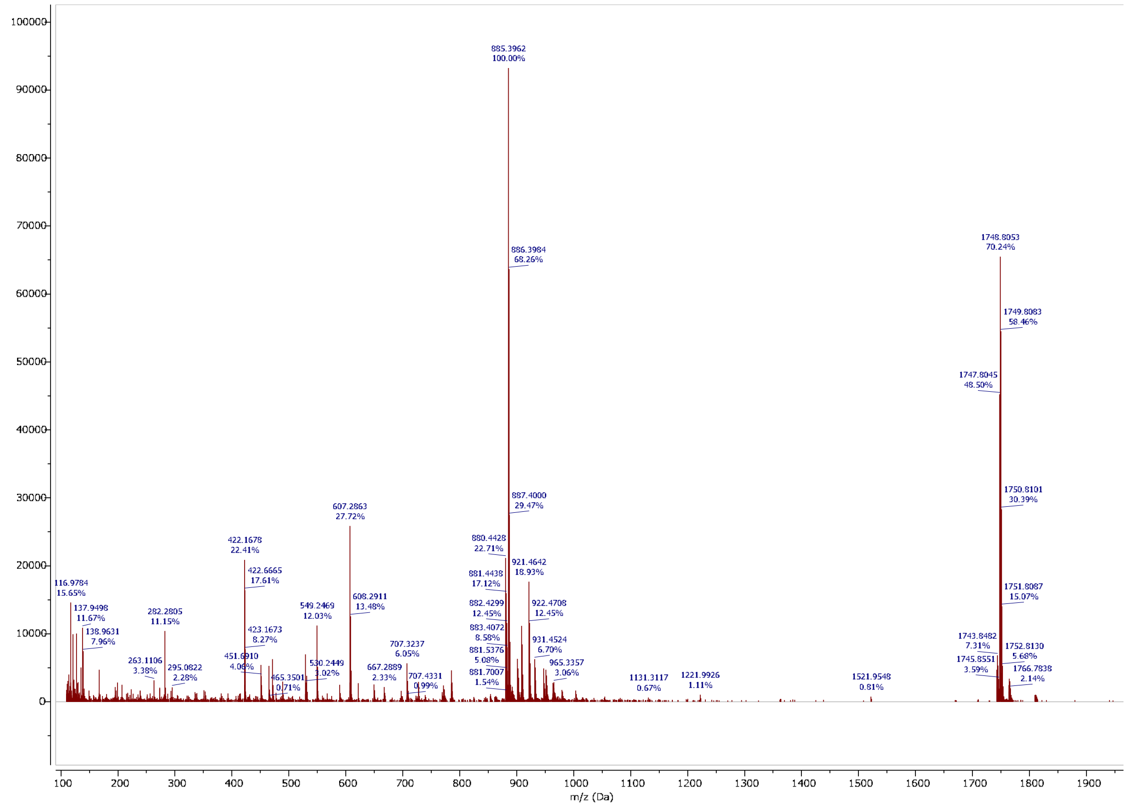
885.3962
100.00%
1748.8053
70.24%
886.3984
68.26%
1749.8083
58.46%
1747.8045
48.50%
607.2863
27.72%
422.1678
22.41%
m/z (Da)

36

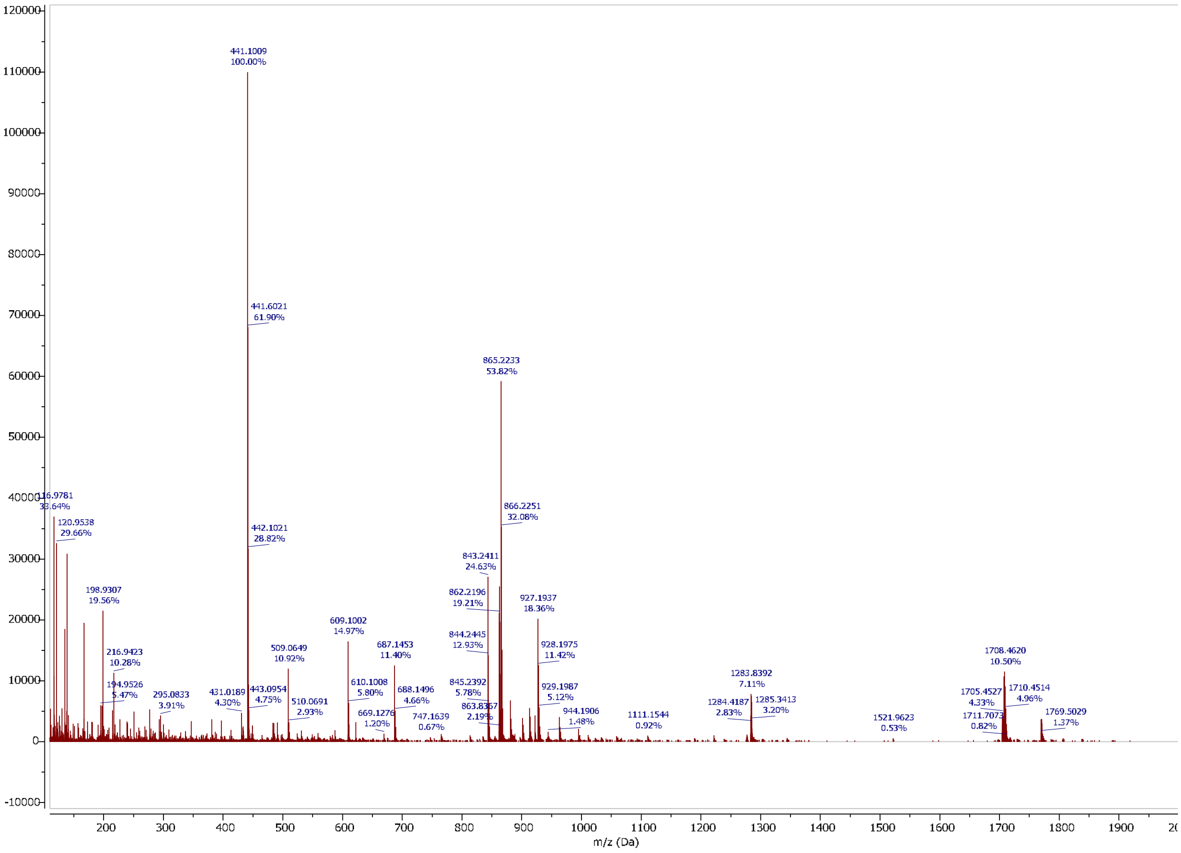
441.1009
100.00%
441.6021
61.90%
865.2233
53.82%
116.9781
33.64%
866.2251
32.08%
1708.4620
10.50%
m/z (Da)

37

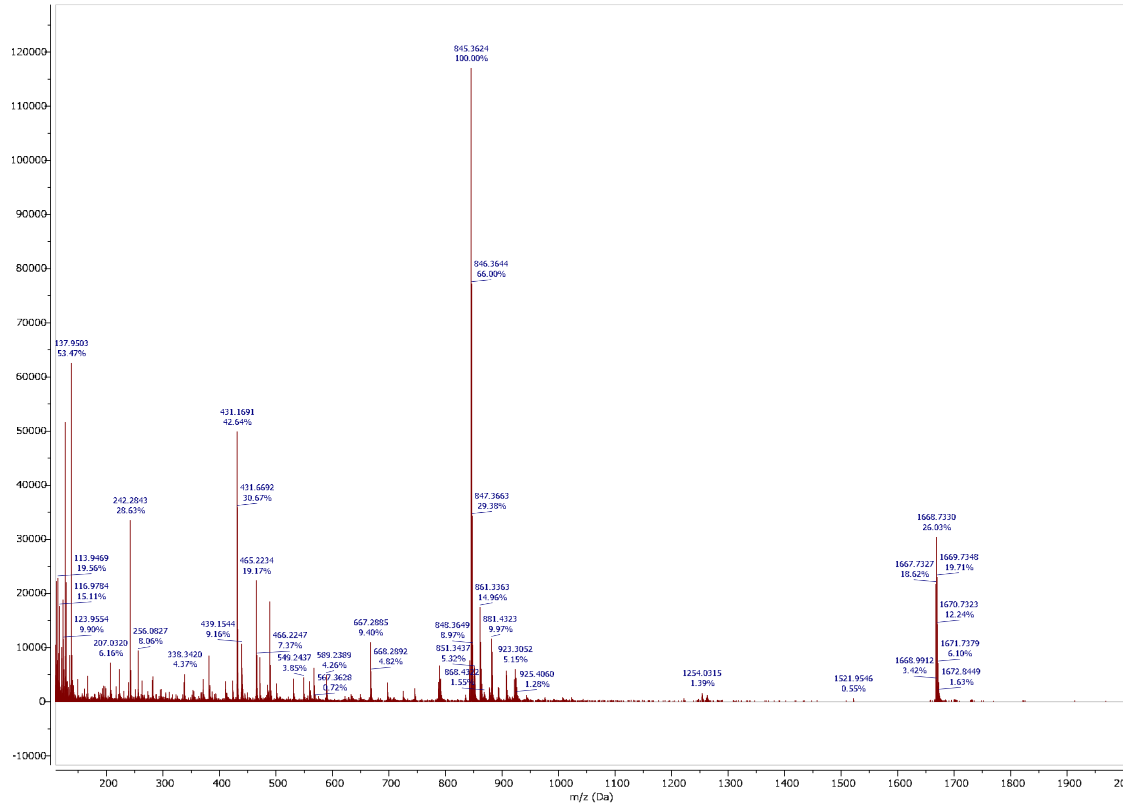
845.3624
100.00%
846.3644
66.00%
137.9503
53.47%
431.1691
42.64%
431.6692
30.67%
242.2843
28.63%
847.3663
29.38%
1668.7330
26.03%
1667.7327
18.62%
1669.7348
19.71%
113.9469
19.56%
116.9784
15.11%
465.2234
19.17%
861.3363
14.96%
1670.7323
12.24%
123.9554
9.90%
207.0320
6.16%
256.0827
8.06%
338.3420
4.37%
439.1544
9.16%
466.2247
7.37%
549.2437
3.85%
589.2389
4.26%
567.3628
0.72%
667.2885
9.40%
668.2892
4.82%
848.3649
8.97%
851.3437
5.32%
868.4362
1.55%
881.4323
9.97%
923.3052
5.15%
925.4060
1.28%
1254.0315
1.39%
1521.9546
0.55%
1668.9912
3.42%
1671.7379
6.10%
1672.8449
1.63%
m/z (Da)

38

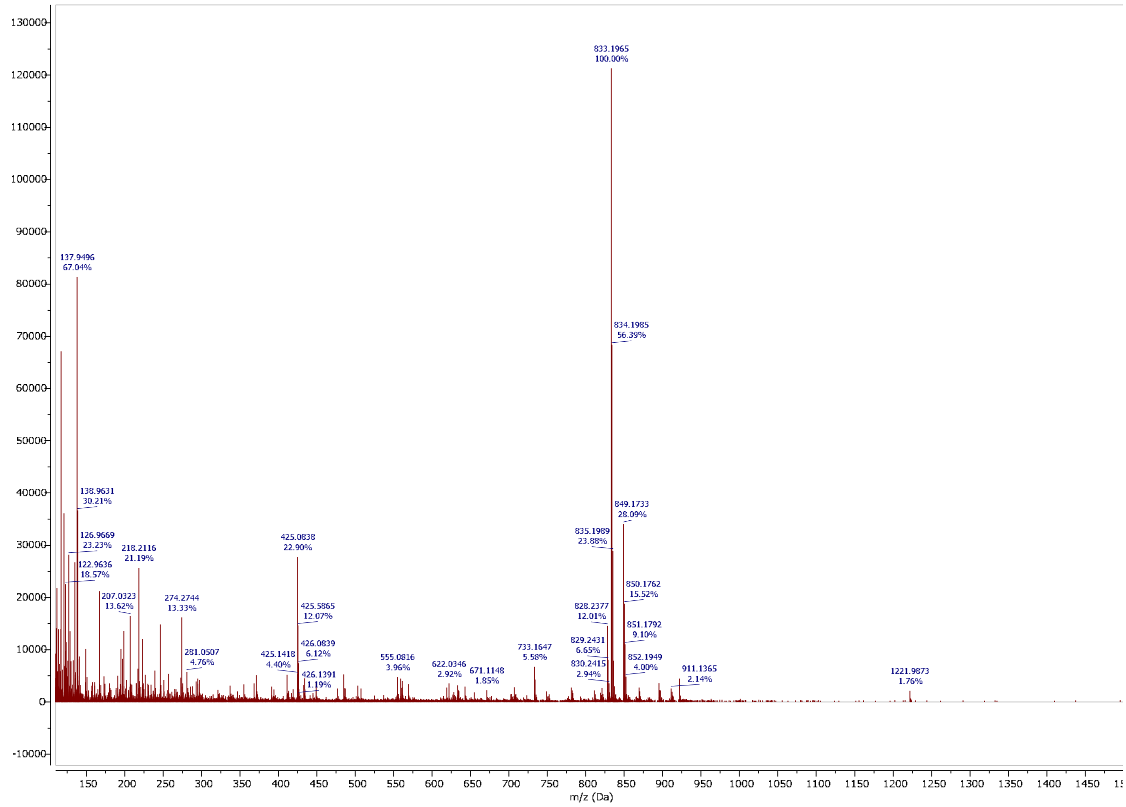
833.1965
100.00%
137.9496
67.04%
834.1985
56.39%
138.9631
30.21%
126.9669
23.23%
122.9636
18.57%
218.2116
21.19%
207.0323
13.62%
274.2744
13.33%
281.0507
4.76%
425.0838
22.90%
425.5865
12.07%
426.0839
6.12%
425.1418
4.40%
426.1391
1.19%
555.0816
3.96%
622.0346
2.92%
671.1148
1.85%
733.1647
5.58%
835.1989
23.88%
828.2377
12.01%
829.2431
6.65%
830.2415
2.94%
849.1733
28.09%
850.1762
15.52%
851.1792
9.10%
852.1949
4.00%
911.1365
2.14%
1221.9873
1.76%
m/z (Da)

39

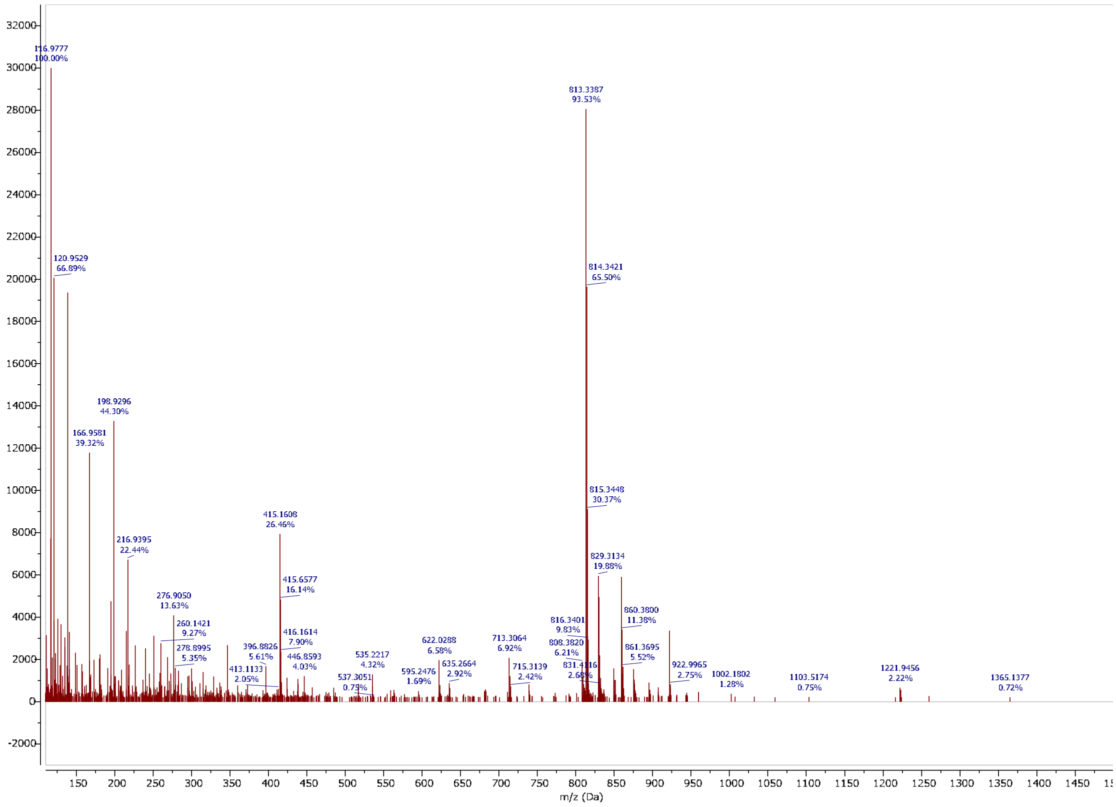

40

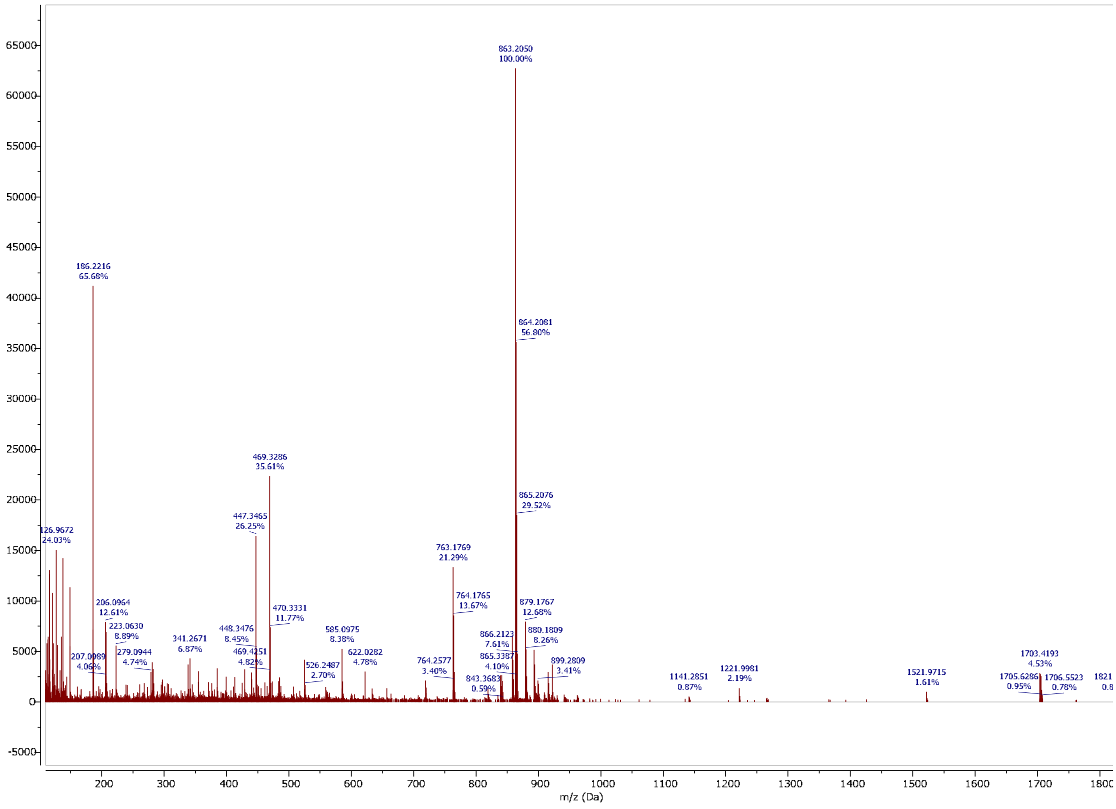

41

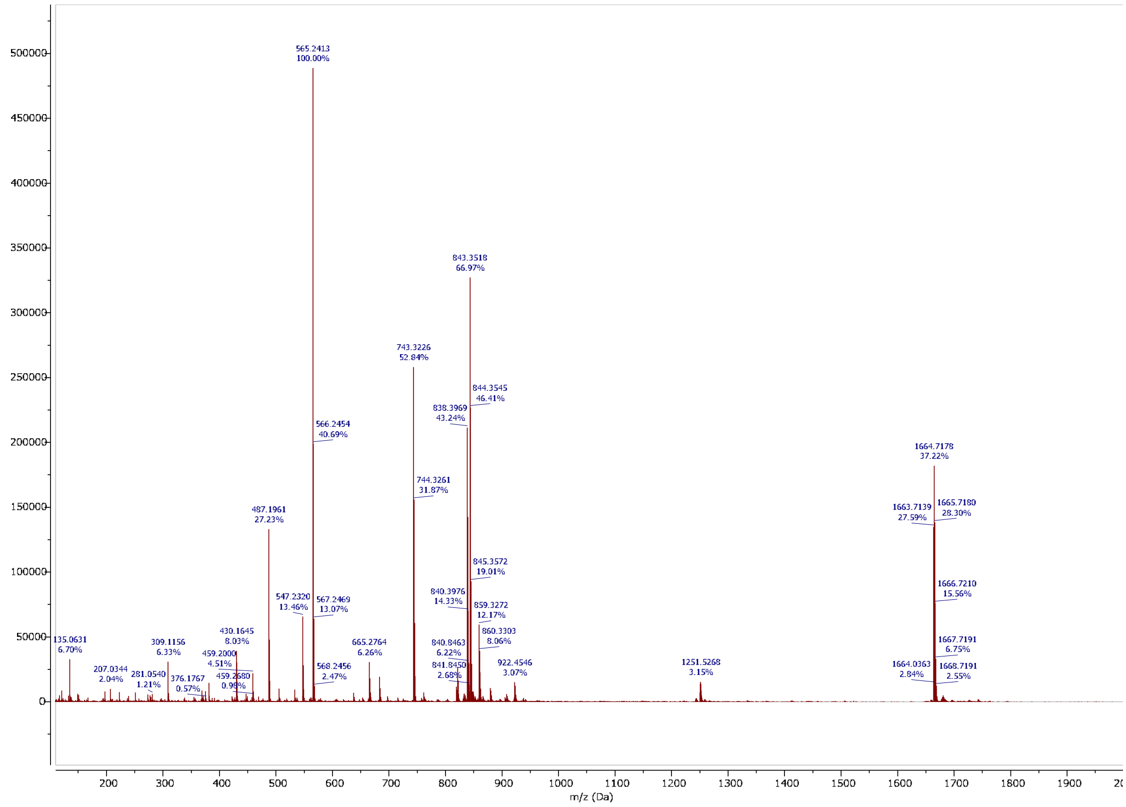

565.2413
100.00%
843.3518
66.97%
743.3226
52.84%
844.3545
46.41%
838.3969
43.24%
566.2454
40.69%
1664.7178
37.22%
744.3261
31.87%
1663.7139
27.59%
1665.7180
28.30%
487.1961
27.23%
845.3572
19.01%
1666.7210
15.56%
840.3976
14.33%
547.2320
13.46%
567.2469
13.07%
859.3272
12.17%
860.3303
8.06%
430.1645
8.03%
135.0631
6.70%
309.1156
6.33%
665.2764
6.26%
840.8463
6.22%
1667.7191
6.75%
459.2000
4.51%
922.4546
3.07%
1251.5268
3.15%
1664.0363
2.84%
1668.7191
2.55%
207.0344
2.04%
281.0540
1.21%
376.1767
0.57%
459.2680
0.98%
568.2456
2.47%
841.8450
2.68%
m/z (Da)

42

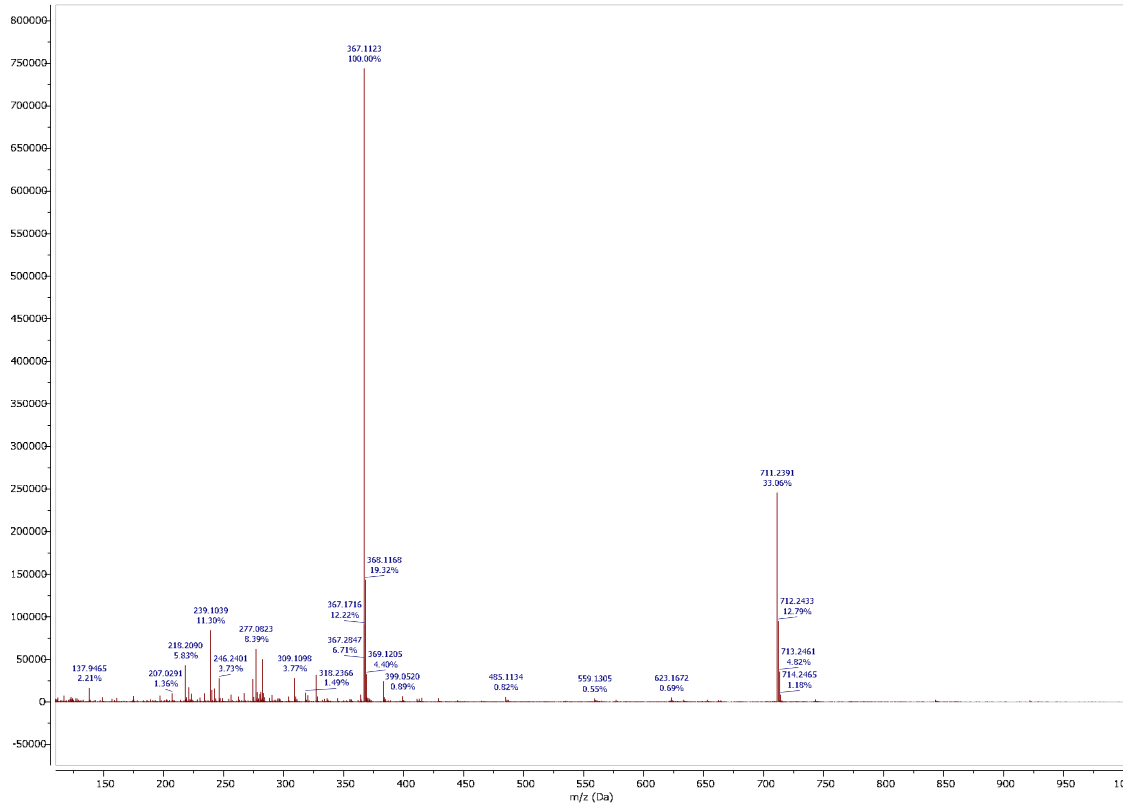

367.1123
100.00%
711.2391
33.06%
368.1168
19.32%
367.1716
12.22%
712.2433
12.79%
239.1039
11.30%
277.0823
8.39%
218.2090
5.83%
367.2847
6.71%
369.1205
4.40%
713.2461
4.82%
246.2401
3.73%
309.1098
3.77%
137.9465
2.21%
207.0291
1.36%
318.2366
1.49%
399.0520
0.89%
485.1134
0.82%
559.1305
0.55%
623.1672
0.69%
714.2465
1.18%
m/z (Da)

43

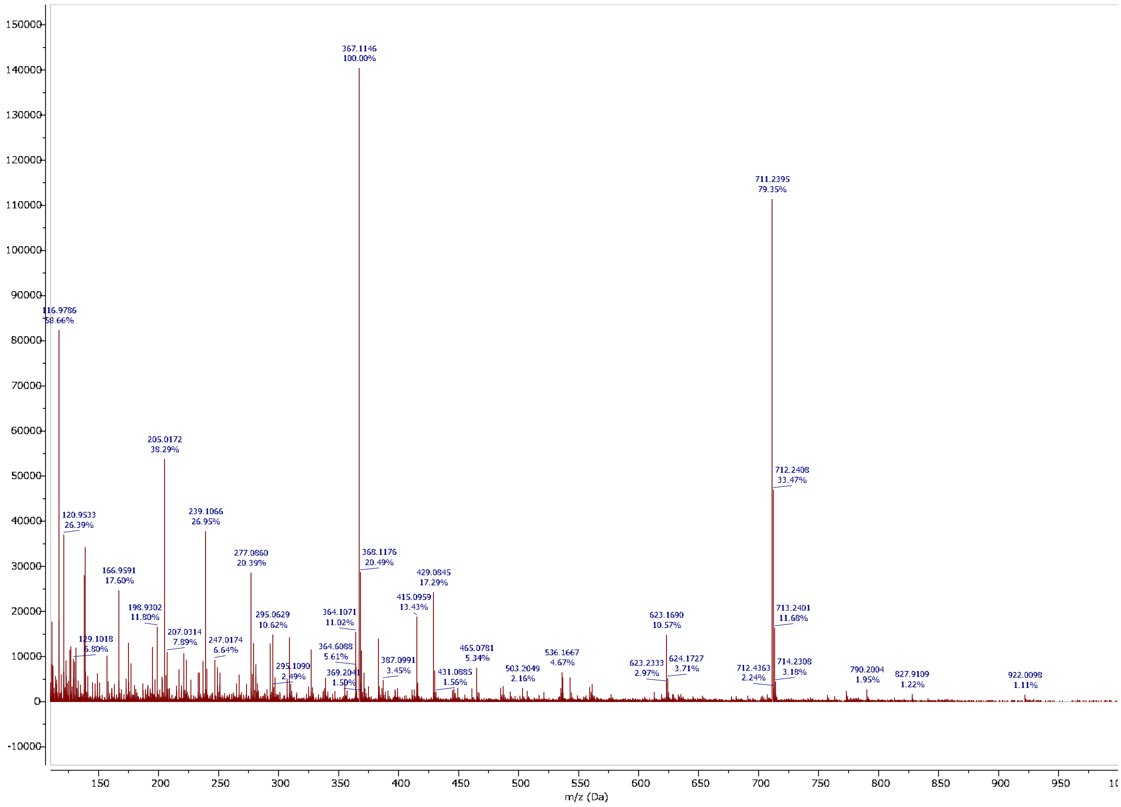

367.1146
100.00%
711.2395
79.35%
116.9786
58.66%
205.0172
38.29%
712.2408
33.47%
120.9533
26.39%
239.1066
26.95%
277.0860
20.39%
368.1176
20.49%
429.0845
17.29%
166.9591
17.60%
415.0959
13.43%
198.9302
11.80%
295.0629
10.62%
364.1071
11.02%
623.1690
10.57%
713.2401
11.68%
129.1018
6.80%
207.0314
7.89%
247.0174
6.64%
364.6088
5.61%
465.0781
5.34%
536.1667
4.67%
387.0991
3.45%
295.1090
2.49%
369.2041
1.50%
431.0885
1.56%
503.2049
2.16%
623.2333
2.97%
624.1727
3.71%
712.4363
2.24%
714.2308
3.18%
790.2004
1.95%
827.9109
1.22%
922.0098
1.11%
m/z (Da)

44

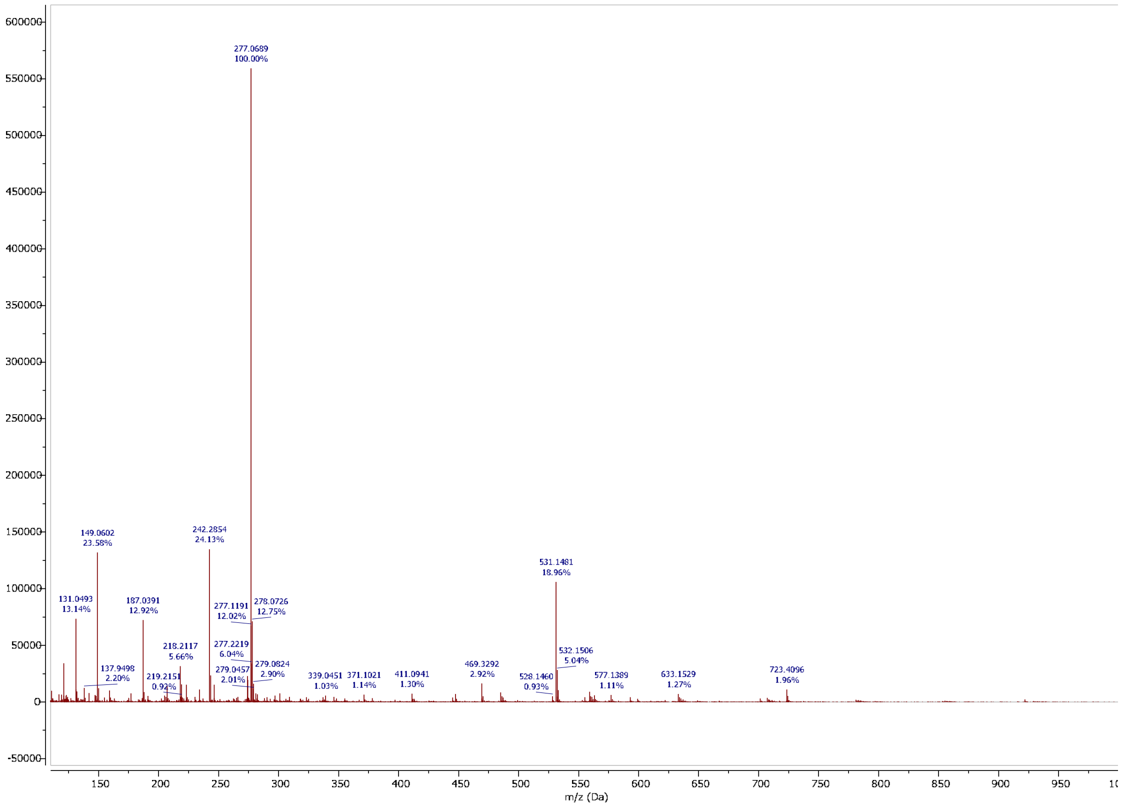

277.0689
100.00%
149.0602
23.58%
242.2854
24.13%
531.1481
18.96%
131.0493
13.14%
187.0391
12.92%
277.1191
12.02%
278.0726
12.75%
218.2117
5.66%
277.2219
6.04%
532.1506
5.04%
137.9498
2.20%
219.2151
0.92%
279.0457
2.01%
279.0824
2.90%
339.0451
1.03%
371.1021
1.14%
411.0941
1.30%
469.3292
2.92%
528.1460
0.93%
577.1389
1.11%
633.1529
1.27%
723.4096
1.96%
m/z (Da)

45

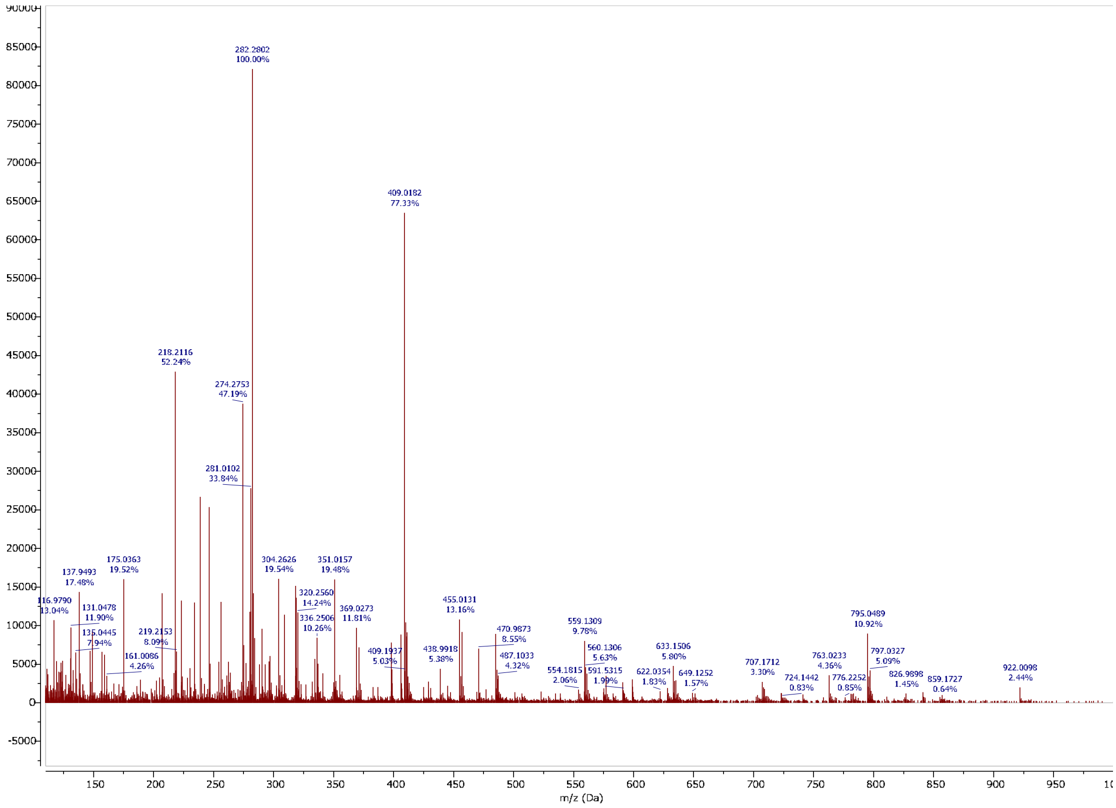
282.2802
100.00%
409.0182
77.33%
218.2116
52.24%
274.2753
47.19%
281.0102
33.84%
175.0363
19.52%
137.9493
17.48%
304.2626
19.54%
351.0157
19.48%
116.9790
13.04%
131.0478
11.90%
135.0445
7.94%
161.0086
4.26%
219.2153
8.09%
320.2560
14.24%
336.2506
10.26%
369.0273
11.81%
409.1937
5.03%
438.9918
5.38%
455.0131
13.16%
470.9873
8.55%
487.1033
4.32%
554.1815
2.06%
559.1309
9.78%
560.1306
5.63%
591.5315
1.99%
622.0354
1.83%
633.1506
5.80%
649.1252
1.57%
707.1712
3.30%
724.1442
0.83%
763.0233
4.36%
776.2252
0.85%
795.0489
10.92%
797.0327
5.09%
826.9898
1.45%
859.1727
0.64%
922.0098
2.44%
m/z (Da)
