# Supplementary material for: Synthesis, Biological Evaluation, and Molecular Docking Studies of 2r,3t,4c‐Configured C‐Furanosidic LpxC Inhibitors
Source: Arch Pharm (Weinheim). 2026 Jul 7;359(6):e70270. doi: 10.1002/ardp.70270 (PMC13342928; doi:10.1002/ardp.70270)
Supplement: Supplementary file 2 — Supporting File 2 [file ARDP-359-e70270-s001.doc]

**Supplemental Material: Novel Compounds and Biological Screening Results**

**Synthesis, biological evaluation, and molecular docking studies of 2*r*,3*t*,4*c*-configured *C*-furanosidic LpxC inhibitors**

André Behnk,^a,b^ Fabian Lüttchens,^a,b^ Frederick Wichter,^a,b^ Katharina Hoff,^a,b^ Elisa Venanzi,^a^ Stefan Wimmer,^a^ Christopher Vorreiter,^c^ Katharina Rox,^d,e^ Wolfgang Sippl,^c^ and Ralph Holl^a,b^,*

^a^Institute of Organic Chemistry, Universität Hamburg, Martin-Luther-King-Platz 6, 20146 Hamburg, Germany

^b^German Center for Infection Research (DZIF), partner site Hamburg-Lübeck-Borstel-Riems

^c^Institute of Pharmacy, Martin-Luther-University of Halle-Wittenberg, Kurt-Mothes-Str. 3, 06120 Halle (Saale), Germany

^d^Department of Chemical Biology, Helmholtz Centre for Infection Research (HZI), Inhoffenstr. 7, 38124 Braunschweig, Germany

^e^German Center for Infection Research (DZIF), partner site Hannover-Braunschweig

*To whom correspondence should be addressed. Tel.: +49-40-2395-22825; E-mail: [ralph.holl@uni-hamburg.de](mailto:ralph.holl@uni-hamburg.de)

| **Compound No.** | **InChI** | **Biological Activity (*K*_i_)^a^** |
| --- | --- | --- |
| 15 | InChI=1S/C24H26N2O6/c27-20-21(28)23(24(29)25-30)32-22(20)19-9-7-17(8-10-19)2-1-16-3-5-18(6-4-16)15-26-11-13-31-14-12-26/h3-10,20-23,27-28,30H,11-15H2,(H,25,29)/t20-,21-,22-,23-/m0/s1 | 3.55 µM |
| ent-15 | InChI=1S/C24H26N2O6/c27-20-21(28)23(24(29)25-30)32-22(20)19-9-7-17(8-10-19)2-1-16-3-5-18(6-4-16)15-26-11-13-31-14-12-26/h3-10,20-23,27-28,30H,11-15H2,(H,25,29)/t20-,21-,22-,23-/m1/s1 | 2.27 µM |
| 16 | InChI=1S/C24H26N2O6/c27-20-21(28)23(24(29)25-30)32-22(20)19-9-7-17(8-10-19)2-1-16-3-5-18(6-4-16)15-26-11-13-31-14-12-26/h3-10,20-23,27-28,30H,11-15H2,(H,25,29)/t20-,21-,22+,23-/m0/s1 | 9.94 µM |
| ent-16 | InChI=1S/C24H26N2O6/c27-20-21(28)23(24(29)25-30)32-22(20)19-9-7-17(8-10-19)2-1-16-3-5-18(6-4-16)15-26-11-13-31-14-12-26/h3-10,20-23,27-28,30H,11-15H2,(H,25,29)/t20-,21-,22+,23-/m1/s1 | 2.50 µM |
| 19 | InChI=1S/C25H34O8/c1-25(2)32-16-22(33-25)24(31-15-18-7-11-20(29-4)12-8-18)23(21(27)13-26)30-14-17-5-9-19(28-3)10-6-17/h5-12,21-24,26-27H,13-16H2,1-4H3/t21-,22+,23-,24-/m1/s1 |  |
| 20 | InChI=1S/C24H30O7/c1-24(2)30-16-22(31-24)23(29-15-18-7-11-20(27-4)12-8-18)21(13-25)28-14-17-5-9-19(26-3)10-6-17/h5-13,21-23H,14-16H2,1-4H3/t21-,22+,23+/m1/s1 |  |
| 21 | InChI=1S/C27H29IO6/c1-30-22-11-3-18(4-12-22)16-32-26-24(15-29)34-25(20-7-9-21(28)10-8-20)27(26)33-17-19-5-13-23(31-2)14-6-19/h3-14,24-27,29H,15-17H2,1-2H3/t24-,25+,26-,27-/m1/s1 |  |
| 22 | InChI=1S/C27H29IO6/c1-30-22-11-3-18(4-12-22)16-32-26-24(15-29)34-25(20-7-9-21(28)10-8-20)27(26)33-17-19-5-13-23(31-2)14-6-19/h3-14,24-27,29H,15-17H2,1-2H3/t24-,25-,26-,27-/m1/s1 |  |
| 23 | InChI=1S/C28H29IO7/c1-31-22-12-4-18(5-13-22)16-34-25-24(20-8-10-21(29)11-9-20)36-27(28(30)33-3)26(25)35-17-19-6-14-23(32-2)15-7-19/h4-15,24-27H,16-17H2,1-3H3/t24-,25+,26-,27-/m0/s1 |  |
| 24 | InChI=1S/C28H29IO7/c1-31-22-12-4-18(5-13-22)16-34-25-24(20-8-10-21(29)11-9-20)36-27(28(30)33-3)26(25)35-17-19-6-14-23(32-2)15-7-19/h4-15,24-27H,16-17H2,1-3H3/t24-,25-,26+,27+/m1/s1 |  |
| 25 | InChI=1S/C12H13IO5/c1-17-12(16)11-9(15)8(14)10(18-11)6-2-4-7(13)5-3-6/h2-5,8-11,14-15H,1H3/t8-,9-,10-,11-/m0/s1 |  |
| ent-25 | InChI=1S/C12H13IO5/c1-17-12(16)11-9(15)8(14)10(18-11)6-2-4-7(13)5-3-6/h2-5,8-11,14-15H,1H3/t8-,9-,10-,11-/m1/s1 |  |
| 26 | InChI=1S/C12H13IO5/c1-17-12(16)11-9(15)8(14)10(18-11)6-2-4-7(13)5-3-6/h2-5,8-11,14-15H,1H3/t8-,9-,10+,11-/m0/s1 |  |
| 27 | InChI=1S/C25H27NO6/c1-30-25(29)24-22(28)21(27)23(32-24)20-10-8-18(9-11-20)3-2-17-4-6-19(7-5-17)16-26-12-14-31-15-13-26/h4-11,21-24,27-28H,12-16H2,1H3/t21-,22-,23-,24-/m0/s1 |  |
| ent-27 | InChI=1S/C25H27NO6/c1-30-25(29)24-22(28)21(27)23(32-24)20-10-8-18(9-11-20)3-2-17-4-6-19(7-5-17)16-26-12-14-31-15-13-26/h4-11,21-24,27-28H,12-16H2,1H3/t21-,22-,23-,24-/m1/s1 |  |
| 28 | InChI=1S/C25H27NO6/c1-30-25(29)24-22(28)21(27)23(32-24)20-10-8-18(9-11-20)3-2-17-4-6-19(7-5-17)16-26-12-14-31-15-13-26/h4-11,21-24,27-28H,12-16H2,1H3/t21-,22-,23+,24-/m0/s1 |  |
| ent-28 | InChI=1S/C25H27NO6/c1-30-25(29)24-22(28)21(27)23(32-24)20-10-8-18(9-11-20)3-2-17-4-6-19(7-5-17)16-26-12-14-31-15-13-26/h4-11,21-24,27-28H,12-16H2,1H3/t21-,22-,23+,24-/m1/s1 |  |
| 31 | InChI=1S/C41H50O6Si2/c1-39(2,3)48(30-21-13-9-14-22-30,31-23-15-10-16-24-31)46-36-35(34-29-43-41(7,8)45-34)44-38(42)37(36)47-49(40(4,5)6,32-25-17-11-18-26-32)33-27-19-12-20-28-33/h9-28,34-37H,29H2,1-8H3/t34-,35+,36+,37-/m1/s1 |  |
| 32 | InChI=1S/C47H55IO6Si2/c1-44(2,3)55(36-21-13-9-14-22-36,37-23-15-10-16-24-37)53-42-41(40-33-50-46(7,8)51-40)52-47(49,34-29-31-35(48)32-30-34)43(42)54-56(45(4,5)6,38-25-17-11-18-26-38)39-27-19-12-20-28-39/h9-32,40-43,49H,33H2,1-8H3/t40-,41-,42+,43-,47?/m1/s1 |  |
| 33 | InChI=1S/C54H62O7Si2/c1-51(2,3)62(43-26-16-10-17-27-43,44-28-18-11-19-29-44)60-49-48(47-39-57-53(7,8)58-47)59-54(55,41-34-36-42(37-35-41)56-38-40-24-14-9-15-25-40)50(49)61-63(52(4,5)6,45-30-20-12-21-31-45)46-32-22-13-23-33-46/h9-37,47-50,55H,38-39H2,1-8H3/t47-,48-,49-,50+,54?/m0/s1 |  |
| 34 | InChI=1S/C47H55IO5Si2/c1-45(2,3)54(36-21-13-9-14-22-36,37-23-15-10-16-24-37)52-43-41(34-29-31-35(48)32-30-34)50-42(40-33-49-47(7,8)51-40)44(43)53-55(46(4,5)6,38-25-17-11-18-26-38)39-27-19-12-20-28-39/h9-32,40-44H,33H2,1-8H3/t40-,41+,42-,43+,44+/m1/s1 |  |
| 35 | InChI=1S/C54H62O6Si2/c1-52(2,3)61(43-26-16-10-17-27-43,44-28-18-11-19-29-44)59-50-48(41-34-36-42(37-35-41)55-38-40-24-14-9-15-25-40)57-49(47-39-56-54(7,8)58-47)51(50)60-62(53(4,5)6,45-30-20-12-21-31-45)46-32-22-13-23-33-46/h9-37,47-51H,38-39H2,1-8H3/t47-,48+,49-,50+,51+/m1/s1 |  |
| 36 | InChI=1S/C44H51IO5Si2/c1-43(2,3)51(34-19-11-7-12-20-34,35-21-13-8-14-22-35)49-41-39(32-27-29-33(45)30-28-32)48-40(38(47)31-46)42(41)50-52(44(4,5)6,36-23-15-9-16-24-36)37-25-17-10-18-26-37/h7-30,38-42,46-47H,31H2,1-6H3/t38-,39-,40+,41+,42+/m1/s1 |  |
| 37 | InChI=1S/C51H58O6Si2/c1-50(2,3)58(41-24-14-8-15-25-41,42-26-16-9-17-27-42)56-48-46(39-32-34-40(35-33-39)54-37-38-22-12-7-13-23-38)55-47(45(53)36-52)49(48)57-59(51(4,5)6,43-28-18-10-19-29-43)44-30-20-11-21-31-44/h7-35,45-49,52-53H,36-37H2,1-6H3/t45-,46-,47+,48+,49+/m1/s1 |  |
| 38 | InChI=1S/C43H47IO4Si2/c1-42(2,3)49(34-19-11-7-12-20-34,35-21-13-8-14-22-35)47-40-38(31-45)46-39(32-27-29-33(44)30-28-32)41(40)48-50(43(4,5)6,36-23-15-9-16-24-36)37-25-17-10-18-26-37/h7-31,38-41H,1-6H3/t38-,39+,40-,41-/m0/s1 |  |
| 39 | InChI=1S/C50H54O5Si2/c1-49(2,3)56(41-24-14-8-15-25-41,42-26-16-9-17-27-42)54-47-45(36-51)53-46(39-32-34-40(35-33-39)52-37-38-22-12-7-13-23-38)48(47)55-57(50(4,5)6,43-28-18-10-19-29-43)44-30-20-11-21-31-44/h7-36,45-48H,37H2,1-6H3/t45-,46+,47-,48-/m0/s1 |  |
| 40 | InChI=1S/C44H49IO5Si2/c1-43(2,3)51(34-20-12-8-13-21-34,35-22-14-9-15-23-35)49-39-38(32-28-30-33(45)31-29-32)48-41(42(46)47-7)40(39)50-52(44(4,5)6,36-24-16-10-17-25-36)37-26-18-11-19-27-37/h8-31,38-41H,1-7H3/t38-,39+,40-,41-/m1/s1 |  |
| 41 | InChI=1S/C51H56O6Si2/c1-50(2,3)58(41-25-15-9-16-26-41,42-27-17-10-18-28-42)56-46-45(39-33-35-40(36-34-39)54-37-38-23-13-8-14-24-38)55-48(49(52)53-7)47(46)57-59(51(4,5)6,43-29-19-11-20-30-43)44-31-21-12-22-32-44/h8-36,45-48H,37H2,1-7H3/t45-,46+,47-,48-/m1/s1 |  |
| 42 | InChI=1S/C19H20O6/c1-23-19(22)18-16(21)15(20)17(25-18)13-7-9-14(10-8-13)24-11-12-5-3-2-4-6-12/h2-10,15-18,20-21H,11H2,1H3/t15-,16-,17-,18-/m1/s1 |  |
| 43 | InChI=1S/C19H20O6/c1-23-19(22)18-16(21)15(20)17(25-18)13-7-9-14(10-8-13)24-11-12-5-3-2-4-6-12/h2-10,15-18,20-21H,11H2,1H3/t15-,16-,17+,18-/m1/s1 |  |
| 44 | InChI=1S/C12H14O6/c1-17-12(16)11-9(15)8(14)10(18-11)6-2-4-7(13)5-3-6/h2-5,8-11,13-15H,1H3/t8-,9-,10+,11-/m1/s1 |  |
| 45 | InChI=1S/C13H13F3O8S/c1-22-12(19)11-9(18)8(17)10(23-11)6-2-4-7(5-3-6)24-25(20,21)13(14,15)16/h2-5,8-11,17-18H,1H3/t8-,9-,10+,11-/m1/s1 |  |

^a^ *E. coli LpxC* C63A enzyme inhibition assay: The wells in a black, non-binding, 96-well fluorescence microplate (Greiner Bio One, Frickenhausen) were filled with 93 µL of 26.9 µM UDP-3-*O*-[(*R*)-3-hydroxymyristoyl]-*N*-acetylglucosamine in assay buffer (40 mM sodium morpholinoethanesulfonic acid (pH 6.0), 80 µM dithiothreitol, 0.02% Brij 35). In order to assay the inhibitors at final concentrations from 20 nM up to 20 µM, 2 µL of a respective dilution of the compounds in DMSO were added. The addition of 5 µL of a solution of purified LpxC (10 µg · mL^‑1^) in assay buffer led to final concentrations of 25 µM UDP-3-*O*-[(*R*)-3-hydroxymyristoyl]-*N*-acetylglucosamine, 15 nM *E. coli* LpxC C63A, 2% DMSO, and from 20 nM up to 20 µM inhibitor. The microplate was incubated for 30 min at 37 °C in a plate shaker. Then, the biochemical reaction was stopped by adding 40 µL of 0.625 M sodium hydroxide. The reaction mixture was further incubated for 10 min and neutralized by adding 40 µL of 0.625 M acetic acid. The deacetylated product UDP-3-*O*-[(*R*)-3-hydroxymyristoyl]glucosamine was converted into a fluorescing isoindole by adding 120 µL of a *o*-phthaldialdehyde-2-mercaptoethanol solution, which was prepared by dissolving 10 mg *o*-phthaldialdehyde in 1 mL methanol, diluting the mixture with 24 mL sodium borate buffer (0.1 M), and finally adding 2.5 µL 2-mercaptoethanol. Fluorescence was measured with a Tristar^2^ plate reader (Berthold, Bad Wildbad) at 340 nm excitation and 460 nm emission wavelengths. Each assay was performed at least three times on separate days. The *IC*_50_ values were calculated *via* Probit-log concentration graphs with the aid of the software Origin and were subsequently converted into *K*_i_ values using the Cheng–Prusoff equation. Mielniczuk, S.; Hoff, K.; Baselious, F.; Li, Y.; Haupenthal, J.; Kany, A. M.; Riedner, M.; Rohde, H.; Rox, K.; Hirsch, A. K. H.; Krimm, I.; Sippl, W.; Holl, R. Development of Fragment-Based Inhibitors of the Bacterial Deacetylase LpxC with Low Nanomolar Activity. *Journal of Medicinal Chemistry* **2024**, *67* (19), 17363-17391. DOI: 10.1021/acs.jmedchem.4c01262
